# 2-aminobenzimidazoles for leishmaniasis: From initial hit discovery to *in vivo* profiling

**DOI:** 10.1371/journal.pntd.0009196

**Published:** 2021-02-22

**Authors:** Rafael Augusto Alves Ferreira, Celso de Oliveira Rezende Junior, Pablo David Grigol Martinez, Paul John Koovits, Bruna Miranda Soares, Leonardo L. G. Ferreira, Simone Michelan-Duarte, Rafael Consolin Chelucci, Adriano D. Andricopulo, Mariana K. Galuppo, Silvia R. B. Uliana, An Matheeussen, Guy Caljon, Louis Maes, Simon Campbell, Jadel M. Kratz, Charles E. Mowbray, Luiz Carlos Dias

**Affiliations:** 1 Institute of Chemistry, University of Campinas (UNICAMP), Campinas-SP, Brazil; 2 Laboratory of Medicinal and Computational Chemistry, Physics Institute of São Carlos, University of São Paulo (USP), São Carlos-SP, Brazil; 3 Department of Parasitology, Biomedical Sciences Institute, University of São Paulo (USP), São Paulo-SP, Brazil; 4 Laboratory of Microbiology, Parasitology and Hygiene (LMPH), Antwerpen, Belgium; 5 Drugs for Neglected Diseases *initiative* (DND*i*), Geneva, Switzerland; London School of Hygiene and Tropical Medicine, UNITED KINGDOM

## Abstract

Leishmaniasis is a major infectious disease with hundreds of thousands of new cases and over 20,000 deaths each year. The current drugs to treat this life-threatening infection have several drawbacks such as toxicity and long treatment regimens. A library of 1.8 million compounds, from which the hits reported here are publicly available, was screened against *Leishmania infantum* as part of an optimization program; a compound was found with a 2-aminobenzimidazole functionality presenting moderate potency, low metabolic stability and high lipophilicity. Several rounds of synthesis were performed to incorporate chemical groups capable of reducing lipophilicity and clearance, leading to the identification of compounds that are active against different parasite strains and have improved *in vitro* properties. As a result of this optimization program, a group of compounds was further tested in anticipation of *in vivo* evaluation. *In vivo* tests were carried out with compounds **29** (*L*. *infantum* IC_50_: 4.1 μM) and **39** (*L*. *infantum* IC_50_: 0.5 μM) in an acute *L*. *infantum* VL mouse model, which showed problems of poor exposure and lack of efficacy, despite the good *in vitro* potency.

## Author summary

Leishmaniasis is a neglected tropical disease affecting millions of people worldwide and, in the case of visceral leishmaniasis (VL), is potentially fatal if untreated. Protozoan parasites of the genus *Leishmania spp*. are the causative agents of leishmaniasis, which has different clinical manifestations, including the visceral form and a cutaneous form that causes disfiguring skin lesions. The current treatment options are limited either by the length of treatment or toxic side effects. Starting from a promising hit in an *in vitro* phenotypic screen, hundreds of analogues were synthesized with the aim of finding a molecule capable of killing the parasite whilst causing little or no harm to the patient. The program led to several active compounds with good *in vitro* activity against *L*. *infantum* intracellular amastigotes, however, *in vivo* data showed low parasiticidal efficacy.

## Introduction

According to World Health Organization’s estimates, neglected tropical diseases (NTDs) are common in some 150 countries and affect over one billion people,[1] usually living in poor and vulnerable communities. As one of the most common vector-borne parasitic diseases, leishmaniasis is a classic example of an NTD. Caused by kinetoplastid protozoans of the genus *Leishmania spp*., which comprises over 20 species,[2,3] the disease is widespread and endemic in 98 countries.[4] It presents as diverse clinical manifestations including cutaneous leishmaniasis (CL, self-healing skin ulcers with an estimated incidence of 600,000 to 1,000,000 new cases every year[2,5]), mucosal leishmaniasis (ML)[5] and the potentially fatal VL, (also known as kala-azar)[6] caused by *L*. *donovani* or *L*. *infantum* which infect 200,000–400,000 people annually, causing more than 20,000 deaths.[7,8] Co-infection with HIV is a common complication and is a significant additional threat to patients health.[9]

The *Leishmania* life cycle involves phlebotomine insects (sand flies) as vectors with mammalians, including humans, as the vertebrate host. Upon inoculation of promastigotes, parasites are phagocytosed by macrophages and transform into amastigotes, an appropriate stage for compound screening in phenotypic drug discovery.[3] In the vertebrate host, parasites are located inside parasitophorous vacuoles where the pH is ~5.5, whereas the pH of the cytosol and the interstitial fluid is ~7.4.[8] Thus potential drugs have to cross three membranes to reach the target of action, which is an additional complication that makes drug discovery for leishmaniasis particularly challenging.

Current treatment options for VL are limited due to the different clinical manifestations. They have many drawbacks, such as extensive toxicity, long treatment times, high cost, decreasing efficacy, low compliance and increasing cases of treatment failure or resistance observed for different parasite strains in different regions in the world.[10–12] It is evident that the current drugs (antimonial compounds, amphotericin B, pentamidine, paromomycin and miltefosine) are not sufficient to meet the unmet medical needs of patients with leishmaniasis.

The drug discovery scenario for leishmaniasis has changed significantly over recent years, with the progression of new chemical entities into preclinical and early clinical development as the result of extensive efforts from international consortia and collaborations largely funded by governments and charities.[8,13,14] The research in this manuscript was conducted within the Lead Optimization Latin America (LOLA) consortium in partnership with the Drugs for Neglected Diseases *initiative* (DND*i*).[15] In collaboration with its partners, DND*i* has published a target product profile (TPP) defining minimal and optimal requirements for the development of new treatments for leishmaniasis. Ideally, a treatment for VL should be oral, safe and well tolerated for all ages, during pregnancy and by immune-deficient patients, achieve >95% parasite clearance through a short-course (≤14 days) treatment and be accessible and affordable.[16,17]

**Fig 1:**
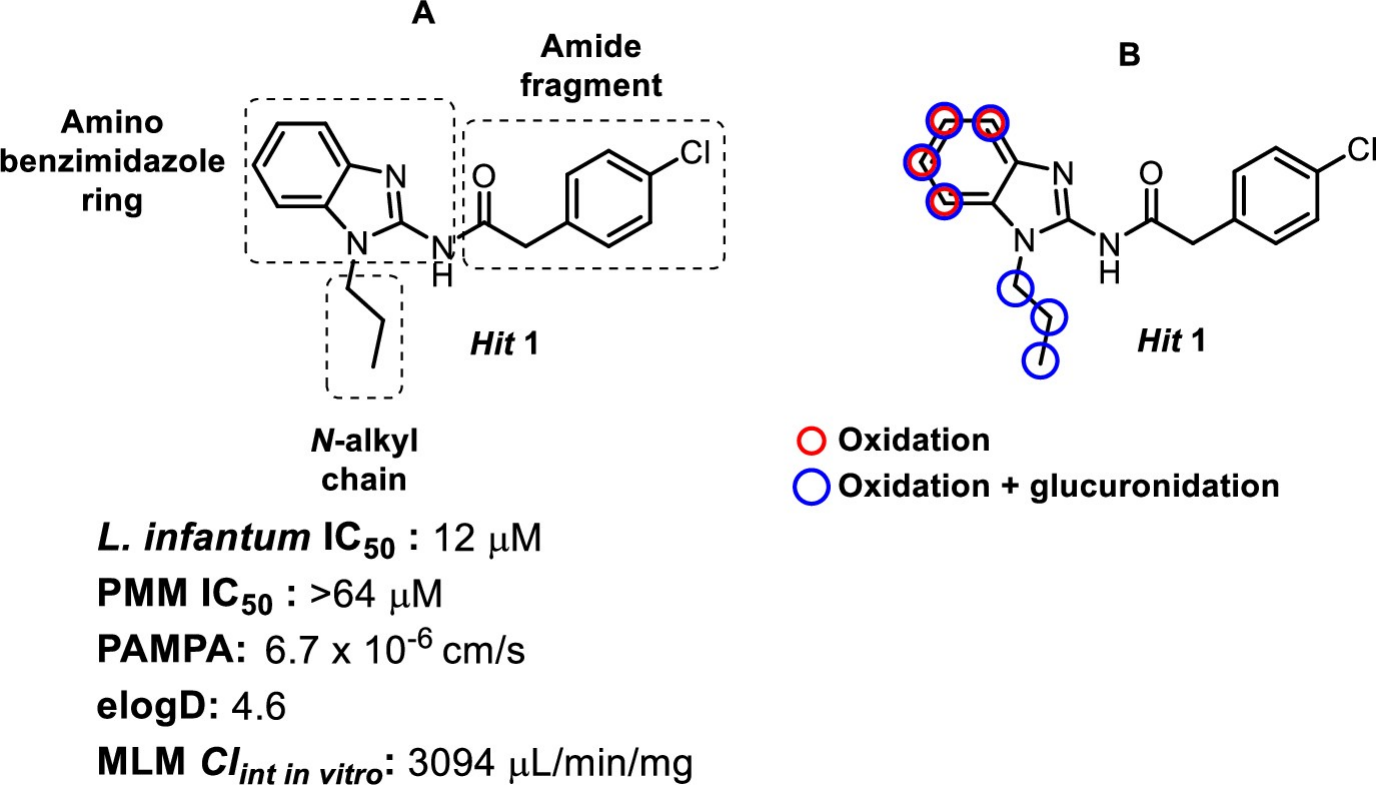
Chemical structure, initial hit assessment data and metabolic soft spots for compound 1.

Following whole-cell, phenotypic screening of GlaxoSmithKline’s diversity set of 1.8 million compounds,[18] the 2-aminobenzimidazole hit **1** (**Fig 1**) was selected for resynthesis and follow-up based on promising activity against the kinetoplastid parasites *L*. *donovani*, *T*. *cruzi* and *T*. *brucei*. Moderate potency was confirmed against intracellular amastigotes of *L*. *infantum* with good selectivity over mammalian cell lines.

The mode of action for this series is still under investigation, but there is recent evidence that acyl aminobenzimidazoles may have the kinetochore CLK1 protein kinase as their primary target.[19]. In recently published work[20], the same 2-aminobenzimidazole scaffold was explored in an early hit-to-lead program where similar structure-activity relationships were observed against *T*. *cruzi* and *T*. *brucei*, suggesting broad-spectrum antiparasitic activity.

Physicochemical and ADME profiling showed good permeability for compound **1**, but with high lipophilicity and low metabolic stability in mouse liver microsomes (MLM). Metabolite identification (MetID) was conducted in mouse S9 microsomal fractions to identify possible soft spots and to direct medicinal chemistry efforts to improve metabolic stability. Oxidation and subsequent glucuronidation on the propyl chain and the benzimidazole ring were identified as the main routes of metabolism (**Fig 1**).

The present study details hit-to-lead optimization for this 2-aminobenzimidazole series to improve the balance between antileishmanial potency, selectivity, and oral bioavailability. This effort led to the profiling of several analogues *in vivo* and to the identification of leads **29** and **39** that were progressed to a mouse model of acute VL.

## Results and discussion

### Synthetic chemistry

**Fig 2:**
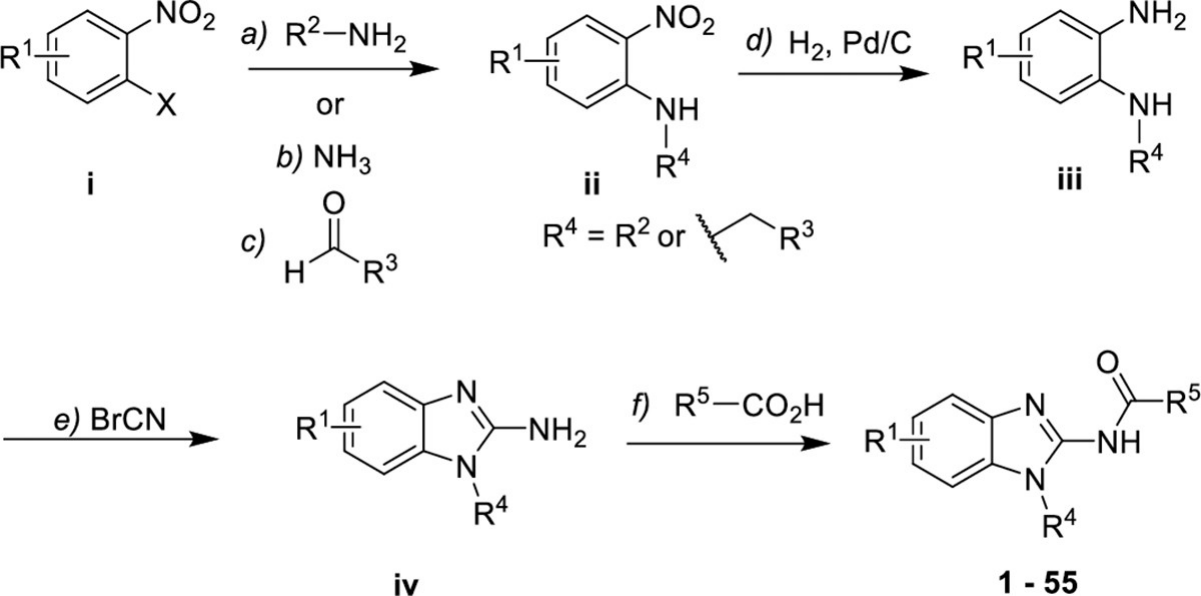
Synthesis of 2-aminobenzimidazoles. Reagents and conditions: **a)** alkylamine, K_2_CO_3_, KF, DMF, 0°C—r.t.; **b)** NH_3_ (7.0 mol.L^-1^ in MeOH), 80°C, 1h, microwave; **c)** Aldehyde, NaBH(OAc)_3_, TFA or DCM, 0°C-r.t, 3h; **d)** H_2_ (1 bar), Pd/C, EtOAc/MeOH, r.t.; **e)** BrCN (1M in DCM), MeOH, 60°C; **f)** EDC, HOBt, DMF, r.t.. Detailed synthetic schemes are shown in S1 Information.

Synthesis was strategically planned to make wide changes to each of the hydrophobic fragments of compound **1**: the benzimidazole ring, *N*-alkyl chain and amide as highlighted above. The aniline intermediate **ii** was prepared by aromatic nucleophilic substitution reaction (S_N_Ar)[21] of compound **i** (X = F or Cl) with primary *N*-alkylamines or with ammonia followed by reductive amination (**Fig 2**). The syntheses of the final compounds (**1–55**) were performed from **ii** by reduction of the nitro group followed by cyclization using cyanogen bromide solution,[22] then coupling of the 2-aminobenzimidazoles **iv** with selected carboxylic acids (**Fig 2**).

**Fig 3:**
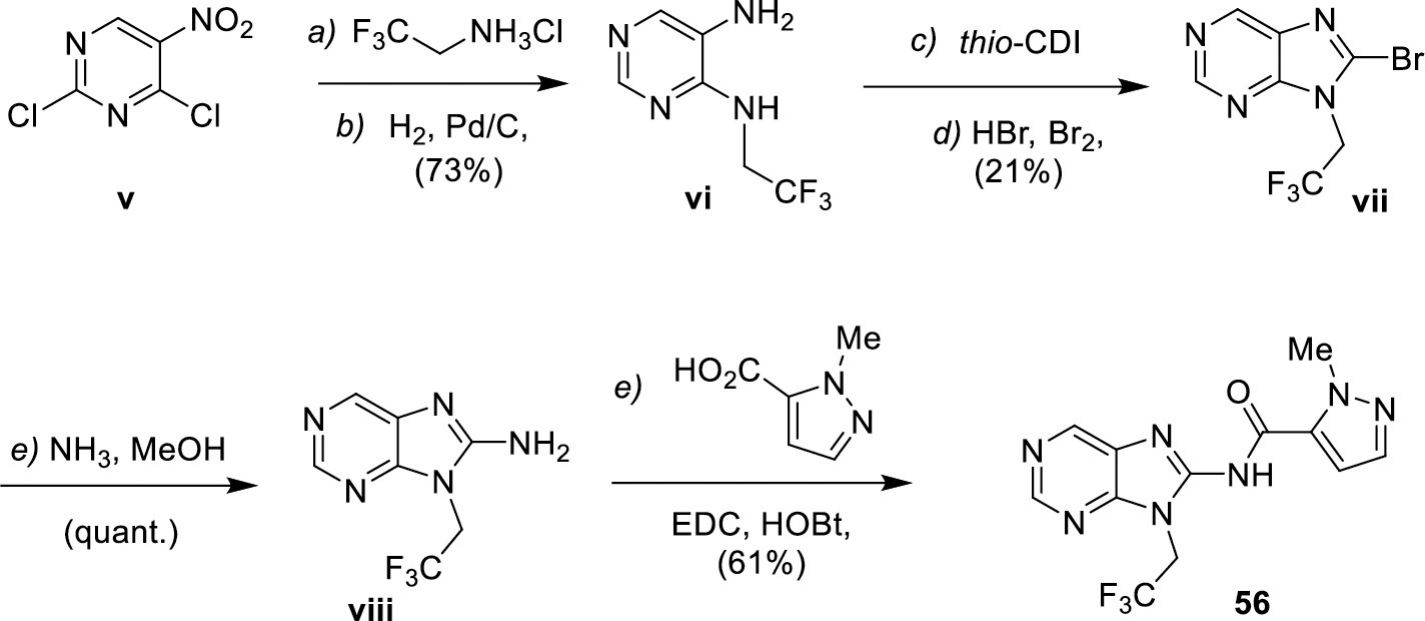
Synthesis of purine 56. Reagents and conditions: **a)** trifluoroethylamonium chloride, DCM, DIPEA, -78°C-r.t.; **b)** H_2_ (1 bar), Pd/C, MeOH, r.t.; **c)** thio-CDI, THF, 70°C, 14h; **d)** HBr (48%), bromine, AcOH, 0°C-r.t., 15h; **e)** NH_3_ (7 mol.L^-1^ in MeOH), 120°C, 2h, microwave; **e)** EDC, HOBt, DMF, r.t.

For examples where cyclization with cyanogen bromide was unsuccessful, such as with strongly electron withdrawing aminopyrimidines, an alternative route was used (**Fig 3**). The nitropyrimidine derivative **v** was transformed into the diaminopyrimidine **vi** by regioselective aromatic nucleophilic substitution with trifluoroethylamonium chloride,[23] followed by reduction of the nitro group concomitant to removal of the chloro in position 2. Diaminopyrimidine **vi** was then cyclized with 1,1’-thiocarbonyldiimidazole followed by reaction with bromine under acidic conditions. The resultant bromo compound **vii** was then transformed into the aminopurine **viii** in quantitative yield by reaction with ammonia under microwave conditions. Finally, **56** was obtained by amide coupling of aminopurine **viii** with *N*-methylpyrazolecarboxylic acid.

### Early hit-to-lead development

Based on the preliminary assessment of compound **1**, the initial medicinal chemistry focus was to reduce lipophilicity (elogD ≤3.5), improve metabolic stability (MLM Clint <25 μL/min/mg) and increase *in vitro* potency (IC_50_ <5 μM), whilst maintaining selectivity (SI >10), with the aim of identifying analogues suitable for testing in a mouse VL efficacy model.

**Fig 4:**
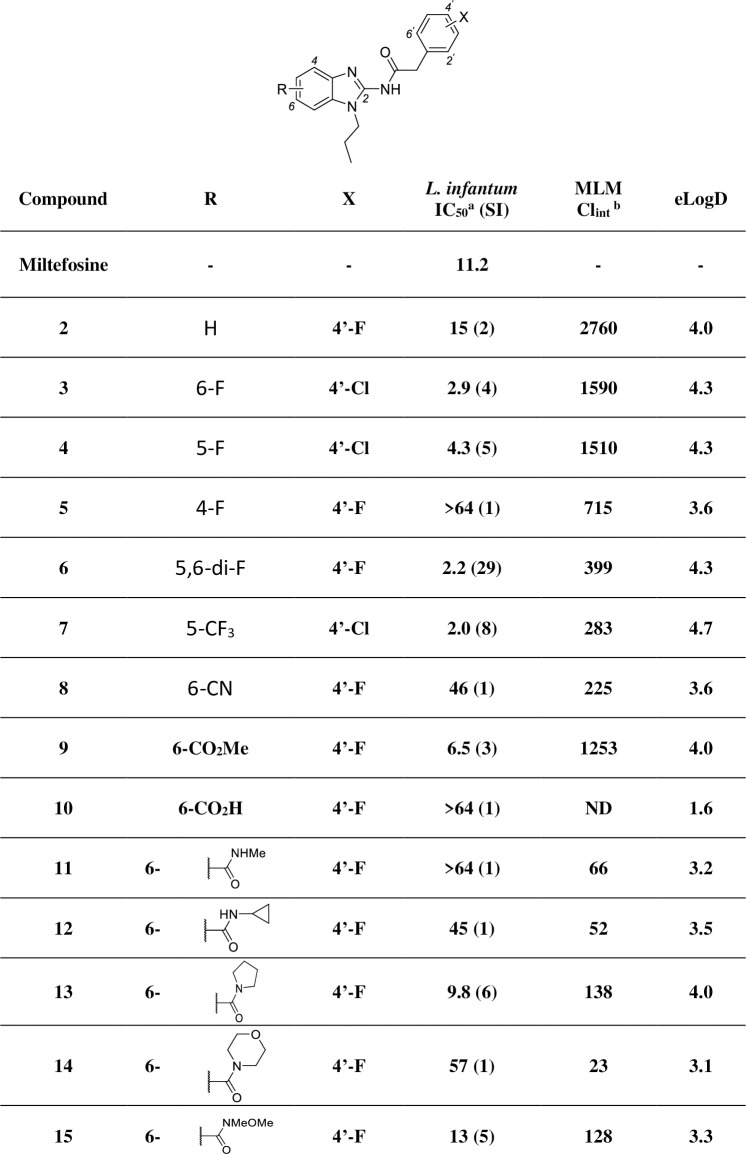
In vitro profiling of analogues with modifications in the benzimidazole ring. SI: selectivity index (CC_50_ / IC_50_); ^a^ IC_50_ represented in μM; MLM: mouse liver microsome; ^b^ intrinsic clearance represented in μL/min/mg.

The results in **Fig 4** show that exchange of chlorine for fluorine on the right-hand side of compound **1** maintained potency and lowered lipophilicity (**2**), so both compounds were used as benchmarks for subsequent SAR and SPR developments.

According to MetID studies (**Fig 1**), the left-hand side of the molecule was prone to oxidation and possibly subsequent glucuronidation, so the initial strategy was to block these soft spots by attaching electron withdrawing groups (EWGs) to the benzimidazole ring which generally reduced MLM intrinsic clearance (Cl_int_). The position of fluorine-substituents on the benzimidazole ring plays an important role in activity. Compounds **3**–**5** show that whilst the 4-position of the benzimidazole ring did not tolerate substitution, fluorine incorporation at the 5- and 6-postions led to a small (3 to 4-fold) improvement in potency compared to the initial hit **1**. Combination of these observations in the 5,6-di-fluorinated analogue **6** maintained the increase in potency, but with much improved clearance (399 vs. 2760 μL/min/mg).

Further SAR development showed that the 6-methyl ester **9** was also potent, but due to the inherent instability of esters in biological systems, alternative isosteric groups were explored. Unfortunately, the parent carboxylic acid (**10**) was inactive. Nitrile (**8**), amido (**11**–**14**) and hydroxamic ester (**15**) groups also had a detrimental impact on *in vitro* potency, despite improving metabolic stability and reducing lipophilicity. A trifluoromethyl group at the 5-position (**7**) was 6-fold more potent than **1** with improved metabolic stability, but at the expense of a still high lipophilicity. Generally, less hydrophobic EWGs improved metabolic stability but reduced potency.

**Fig 5:**
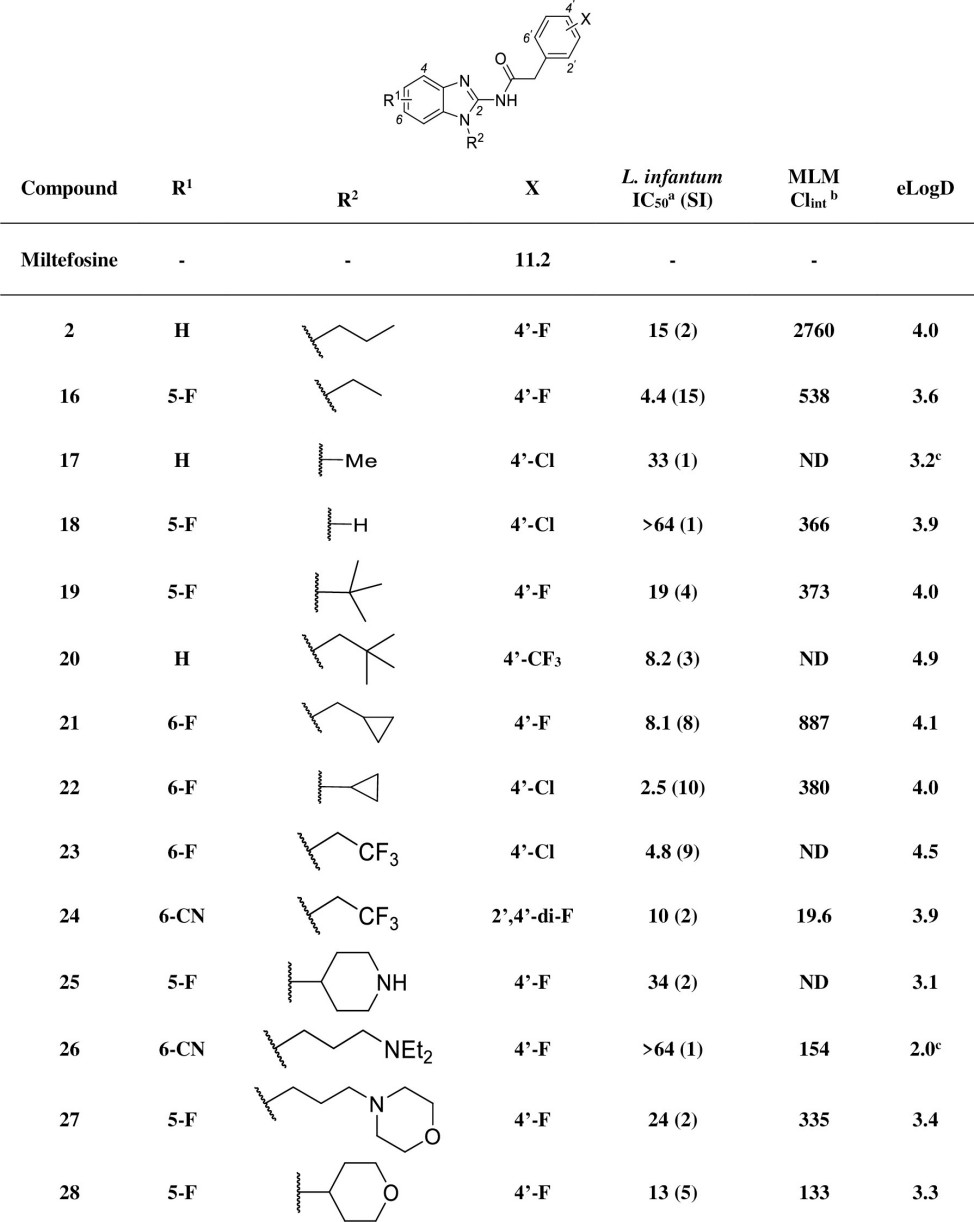
In vitro profiling of analogues with modifications at the N-alkyl chain. ND: not determined; SI: selectivity index (CC_50_ / IC_50_); ^a^ IC_50_ represented in μM; MLM: mouse liver microsome; ^b^ intrinsic clearance represented in μL/min/mg; ^c^ calculated logD.

Following the identification of preferred substitutions (cyano, fluoro and trifluoromethyl groups) on the benzimidazole ring, modifications at other soft spots were explored. MetID suggested that optimization of the *N*-alkyl side chain would be necessary to improve metabolic stability (**Fig 5**). Compounds **2** and **16**–**18** show the importance of the length of the alkyl chain, where a decrease in the number of linear carbon atoms improved metabolic stability but was accompanied by a reduction in potency (except for **16**, which might be also influenced by the EWG attached to the aminobenzimidazole ring). This was further highlighted by removal of the carbon chain in compound **18,** which showed poor activity, underlining the need for hydrophobic groups in this fragment.

To investigate the effect of more elaborate hydrophobic fragments, bulky substituents (**19**–**22**) were introduced. Except for **22**, potency was roughly like the initial hit **1,** however, intrinsic clearance was improved despite high lipophilicity, which may suggest steric impedance of metabolic oxidation.

An alternative approach to reducing lipophilicity was to introduce polar functionalities, such as amine and ether (**25**–**28**), but activity was usually compromised, although the tetrahydropyran derivative **28** showed a similar level of potency as the initial hit.

Installation of a trifluoroethyl moiety as the alkyl chain combined with an EWG cyano function in the benzimidazole ring (**24**) enabled high metabolic stability while unexpectedly maintaining potency (*L*. *infantum* IC_50_ 10 μM), considering the low activity of compound **8** (**Fig 4**, *L*. *infantum* IC_50_ 46 μM). Based on the balance of properties, cyclopropyl, trifluoroethyl and tetrahydropyran groups (**22**–**24** and **28**, respectively) were identified as the preferred N-substitutions on the benzimidazole core.

Modifications of the amide fragment were next investigated by introduction of heteroaromatic rings and removal of the methylene linker using a common 6-cyanobenzimidazole core bearing preferred N-substituents from **Fig 5**.

**Fig 6:**
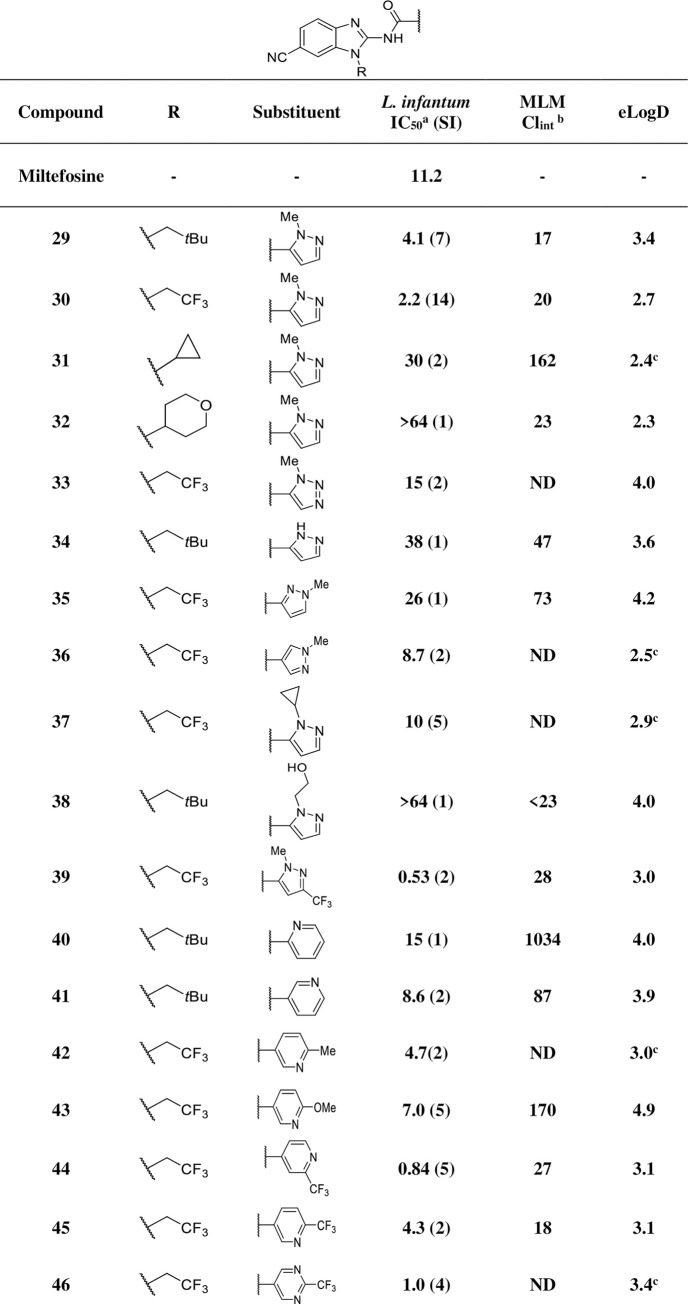
In vitro profiling of analogues with modifications at the amide group. ND: not determined; SI: selectivity index (CC_50_ / IC_50_); ^a^ IC_50_ represented in μM; MLM: mouse liver microsome; ^b^ intrinsic clearance represented in μL/min/mg; ^c^ calculated logD.

Incorporation of an *N*-methylpyrazole group (**29**–**32**, **Fig 6**) produced greater metabolic stability, accompanied by improved *in vitro* potency for **29** and **30** (4.1 and 2.2 μM respectively) with reasonable selectivity. However, combination of the *N*-methylpyrazole group with cyclopropyl or 4-tetrahydropyran as N-substituents led to a reduction in activity (**31** and **32**). Similarly, removal of the pyrazole methyl group (**34**) or replacement by bulkier (cyclopropyl, **37**) or polar (hydroxyethyl, **38**) groups also led to significant reductions in activity.

Further exploration included variation in the connection point of the pyrazole ring (**35** and **36**) and the inclusion of an additional nitrogen atom (triazole, **33**), which showed similar levels of potency but were slightly more cytotoxic to host cell lines.

Addition of a trifluoromethyl group to **30** generated **39** which was 4-fold more potent than the parent compound (**30**), with comparable metabolic stability and lipophilicity. However, this compound was cytotoxic with a low selectivity index.

Other heteroaromatic groups were investigated, such as pyridine (**40**–**45**) and pyrimidine (**46**), with differing degrees of success. Similarly, addition of a trifluoromethyl group to the pyridine ring further improved metabolic stability generating compound **44** with sub-micromolar potency. The most active analogues were the 6-cyano CF_3_-substituted heterocycles **39**, **44** and **46** (IC_50_ = 0.53, 0.84, 1.0 μM, respectively) which were about 10 to 20-fold more potent than initial hit **1**.

**Fig 7:**
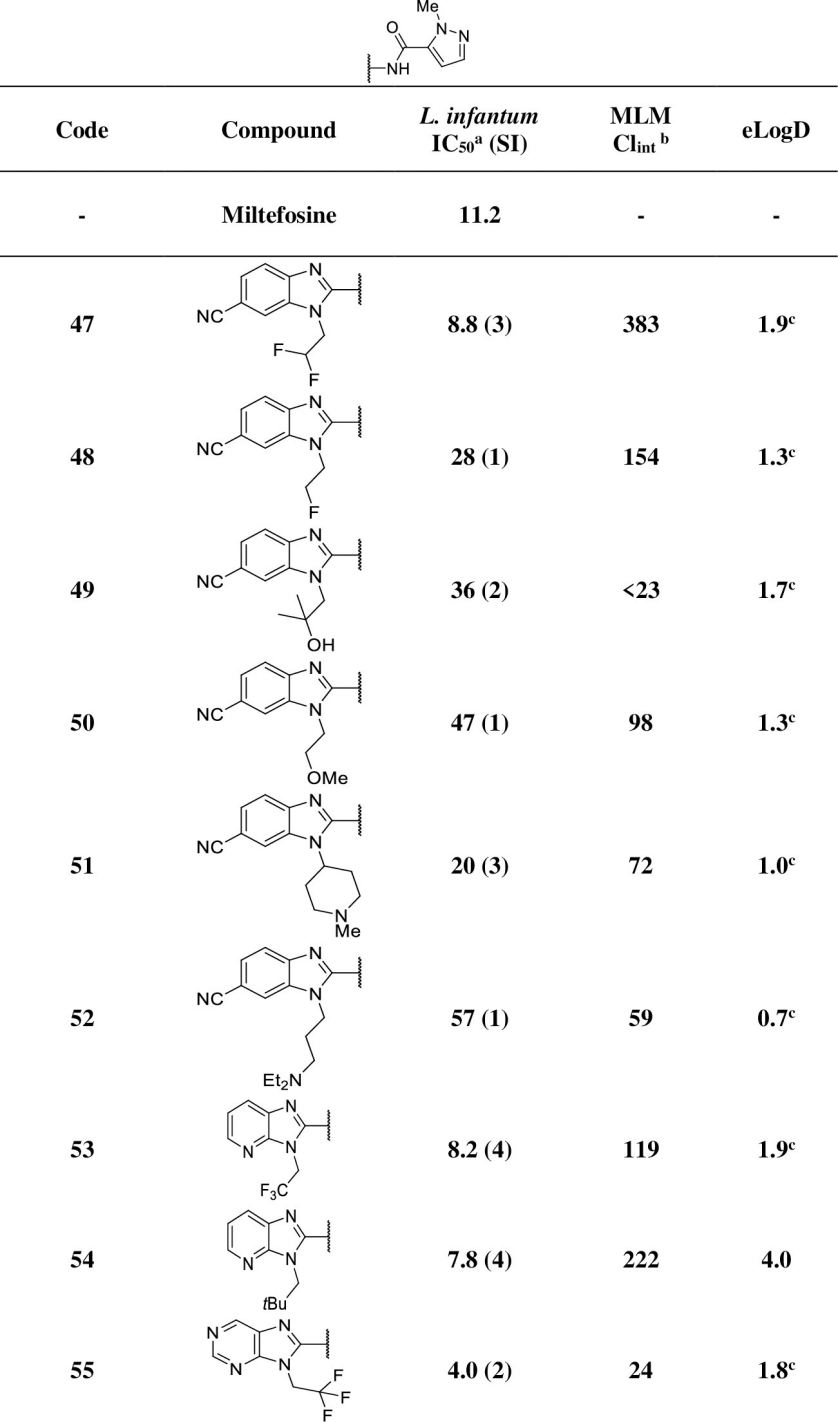
In vitro profiling of analogues with additional modifications at the benzimidazole core and side chain. SI: selectivity index (CC_50_ / IC_50_); ^a^ IC_50_ represented in μM; MLM: mouse liver microsome; ^b^ intrinsic clearance represented in μL/min/mg; ^c^ calculated logD.

With the significant improvement of potency, metabolic stability and lipophilicity generated by these pyrazinamides, additional structural modifications were explored in an attempt to further improve physicochemical properties and selectivity, such as the inclusion of nitrogen atoms (not very basic) in the benzimidazole ring and less hydrophobic side chains (**Fig 7**).

Difluoroethyl and fluoroethyl, and groups with alcohol, ether, and amine functionalities (**47**–**52**) were also evaluated but usually resulted in a loss of potency.

The pyridoimidazole derivatives **53** and **54** displayed similar potency to their benzimidazole counterparts **30** and **29**, but clearance values were much poorer, so they were not pursued further. The purine derivative **55** retained similar potency compared to **30** (**Fig 6**) but was less selective.

**Fig 8:**
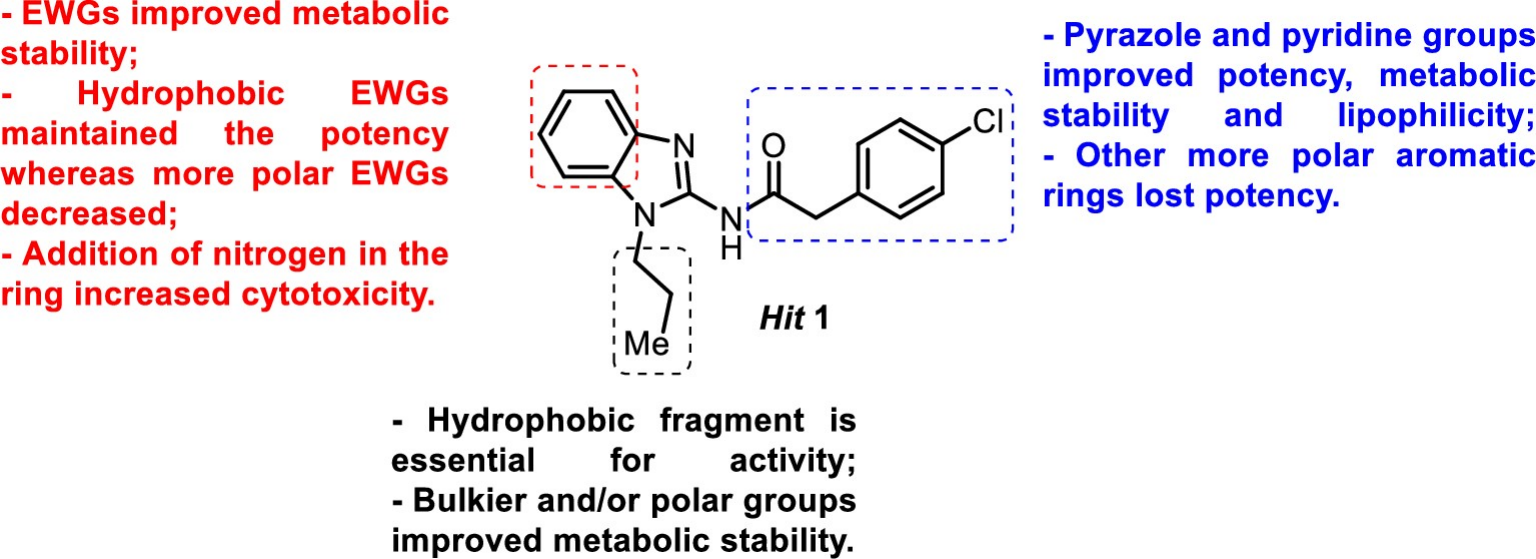
Summary of SARs and SPRs for 2-aminobenzimidazole derivatives.

Analysis of the impact of the structural modifications in the three hydrophobic fragments of the initial hit provided important information for SAR and SPR development (**Fig 8**). In summary, addition of hydrophobic EWGs at the benzimidazole ring retained potency whereas polar EWGs reduced it. A hydrophobic group is essential for activity at the *N*-alkyl chain and bulkier groups improved metabolic stability. Introduction of pyrazole and pyridine derivatives at the amide fragment improved potency, metabolic stability, and lipophilicity.

### Secondary profiling of selected leads

Initial rounds of optimization identified improved analogues such as **29**, **30**, **39** and **44** that showed good *in vitro* potency in combination with excellent metabolic stability; these were progressed for further profiling in anticipation of subsequent *in vivo* evaluation.

Selected compounds (**29** and **39**) were screened against a panel of VL and CL strains (intracellular amastigotes) and showed acceptable IC_50_ values ranging from 0.11–5.9 μM. The only two clear exceptions were *L*. *major* (MHOM/SA/85/JISH118) and *L*. *braziliensis* (MHOM/BR/94/H3227), where a potency drop was evident (see S1 Data for the complete dataset).

**Table 1** shows *in vitro* ADME data for these four compounds. All analogues had comparable microsomal stability (mouse and human) and good passive permeability, as expected for lipophilic compounds that are non-ionized at physiological pH conditions. Additionally, plasma stability and protein binding apparently do not represent significant obstacles for the series. On the other hand, compounds **39** and **40** showed poor aqueous kinetic solubility which could limit oral bioavailability.

**Table 1:** Summary of in vitro ADME and in vivo PK data for selected compounds.

Compound	*L*. *infantum* IC_50_ (SI)	*In vitro* ADME							Single dose mouse PK				
		eLogD	MLM / HLM	KS pH 7.4	PAMPA	Mouse plasma stability	Mouse PPB	Medium binding	Oral dose	C_max_	t_1/2_	AUC last	F
**29**	4.1 (7)	3.4	17 / 25	9.2	4.7	99.8	97.1	71.0	10	1.0	7.9	8.16	72
									50	2.0	ND	35.4	37
**30**	2.2 (14)	2.7	20 / 17	26	7.9	107	95.8	29.2	50	2.0	6.8	25.2	ND
**39**	0.53 (2)	3.0	28 / 20	<1.0	10	111	99.4	88.3	50	7.3	8.0	63.7	ND
**44**	0.84 (5)	3.1	27 / 30	<1.0	2.3	111	99.2	88.7	ND	ND	ND	ND	ND

ND: not determined. SI: selectivity index (CC_50_ / IC_50_). Units: MLM/HLM (mouse liver microsome/human liver microsome): μL/min/mg; KS (kinetic solubility): μM; PAMPA (parallel artificial membrane permeability assay): 10^−6^ cm/s; mouse plasma stability: % remaining after 6h; mouse PPB: % bound; medium binding: % bound; oral dose: mg/kg; C_max_: μg/mL; t_1/2_: h; AUC (area under curve) last: h*μg/mL; F: %.

In parallel, an early *in vitro* safety assessment was conducted in which all compounds showed low inhibition of the hERG channel (IC_50_ >30 μM) and the main cytochrome P450 family enzymes (IC_50_ >20 μM). Screening against an off-target panel covering key human enzymes, kinases and receptors showed little cause for concern. The full dataset is presented in S1 Data. It is important to note that poor aqueous solubility could lead to false-negative results, therefore these results should be confirmed with more soluble analogues.

### *In vivo* pharmacokinetics and tolerability studies

Based on overall *in vitro* profiles, **29**, **30** and **39** were progressed to single-dose pharmacokinetic studies in mice (**Table 1**).

When tested at three dosages (2 mg/kg IV and 10 and 50 mg/kg oral), compound **29** showed promising bioavailability (72 and 37%, respectively) and low clearance of 9 mL/min/kg and had an encouraging plasma half-life (7.9h). However, the free plasma concentration (corrected by the PPB) achieved after single oral dosing was below the *in vitro* IC_50_ (corrected for medium binding) for both doses. Direct comparison of the two oral regimens showed a 2-fold increase in the C_max_ and an approximately 4-fold higher AUC_last_ value, indicative of some lack of dose-linearity (possibly linked to poor solubility). When compared to **29**, compound **39** showed a 2-fold higher exposure (as measured by the AUC_last_) at the same dose. After a single 50 mg/kg dose, a total C_max_ of 7.3 μg/mL (~17 μM) was achieved. Free plasma concentrations (corrected for PPB) of up to four times above the free IC_50_ (corrected for medium binding) were maintained for about 4h, which is a good indicator of exposure over *in vitro* potency at this stage (see supplementary information for the PK plots). Although compound **30** is more soluble, it presented lower AUC, indicating a worse PK profile when compared to the other two analogues.

Based on *in vivo* pharmacokinetic profiling, compounds **29** and **39** were progressed to a 5-day tolerability study in healthy mice. Multiple oral doses (25, 50 and 100 mg/kg/day) were evaluated to identify dosing regimens for the follow-up *in vivo* proof-of-concept study. Both compounds were well tolerated at 25mg/kg/day. In the 50 mg/kg/day group, animals presented clinical signs from day 3 of treatment, such as piloerection, change in behavior and visible irritability. After euthanasia on day 6, no changes were observed in the internal organs. The studies were terminated by day 3 or 4 of treatment at the top dose due to body weight reduction and severe toxic signs, including mortality, with both compounds.

### *In vivo* acute visceral leishmaniasis mouse model

Based on their overall *in vitro* and *in vivo* profiles, **29** and **39** were progressed to a proof-of-concept study employing a bioluminescent acute VL mouse model with a *L*. *infantum* (MHOM/BR/2005/NLC) strain expressing luciferase. The 5-day oral treatment started on day 14 post-infection and animals were imaged at 14 and 19-days post-infection (dpi). Imaging was repeated after an additional 5-day washout period at 24 dpi. Treatment groups included: vehicle, miltefosine 40 mg/kg/day (reference control) and compounds **29, 39** at 25 mg/kg/day.

Animals treated with vehicle showed some toxicity signs from the fourth day of administration, such as piloerection, reduced activity, and irregular breathing. As expected, no decrease in parasite load was detected after treatment. Polyethylene glycol is considered a safe vehicle for oral administration[24] and the side effects observed in this group were unexpected. It must also be noted that vehicle toxicity was not observed when cremophor or saline were used in a CL mouse model.[25,26]

**Fig 9:**
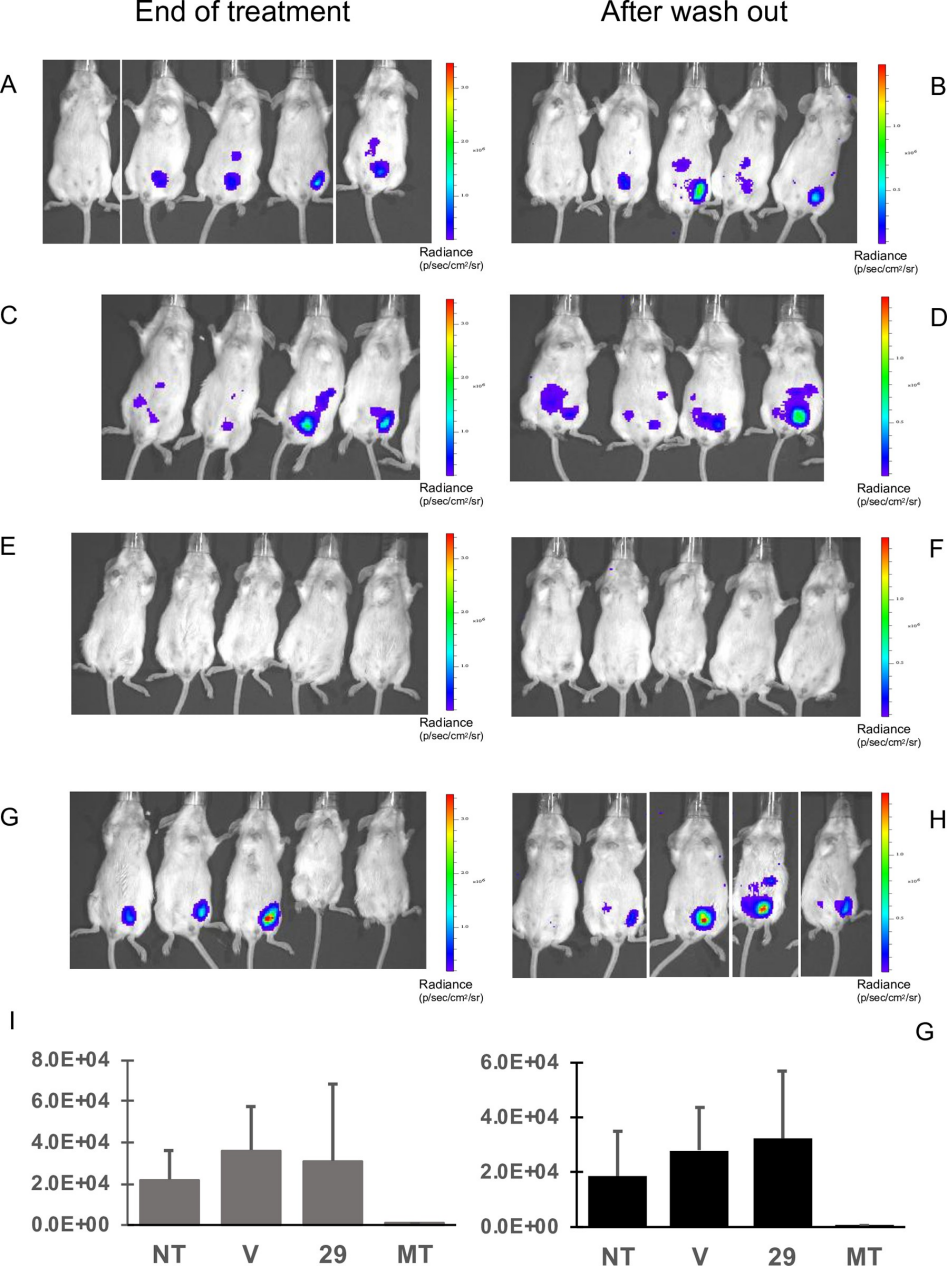
Proof of concept in vivo activity in a VL acute model. BALB/c mice were inoculated with L. infantum and a 5-day treatment was initiated 14 days post-infection. The parasite load was evaluated by bioluminescence at the end of treatment (A, C, E, G) and 5 days later (B, D, F, H). Groups of animals: untreated (A, B), treated with the vehicle (C, D), with 40 mg/kg/day miltefosine (E, F) and 25 mg/kg/day compound **29** (G, H). The bars on the right show a pseudo-colour scale representing light intensities. (I) Percentage variation of parasite burden in treated animals shown as the average bioluminescence in each group at the end of the experiment.

Treatment with miltefosine reduced parasite load, as measured by total ventral bioluminescence, to undetectable levels after 5 days of treatment and remained undetectable throughout the washout period (**Fig 9**).

After 4 days of treatment with compound **29**, one animal was euthanized due to severe clinical signs of distress. The remaining 5 animals survived until the end of the treatment but presented piloerection and diarrhea. Disappointingly, no decrease in parasite load was observed at the end of the treatment or washout period (**Fig 9**). It is possible that appropriate free compound concentrations in plasma and tissues were not attained with the 25 mg/kg/day dose regimen (see supplementary information).

The study with compound **39** had to be stopped on day 2 when animals were humanely euthanized due to severe toxic effects, even though this dose level was well tolerated by healthy animals.

## Conclusion

A 2-aminobenzimidazole derivative identified by HTS, displayed moderate activity against *L*. *infantum*, but with poor physicochemical properties and very rapid metabolism *in vitro*. A focused SAR program improved potency to sub-μM levels and reduced metabolic clearance by over 100-fold. PK profiles *in vivo* were also acceptable with both good bioavailability and plasma half-lives. However, progression of two prototypes to a mouse proof of concept model showed lack of efficacy, possibly linked with low systemic exposure, and a poor safety profile. Despite considerable efforts, our team was not able to further optimize the candidates’ properties and this prompted our decision to interrupt further work with this series. Our results highlight the difficulty within the series to find compounds with better safety and pharmacokinetics profile. Nevertheless, the results presented in this manuscript (and complete dataset available in S1 Data) may be a valuable resource for the drug discovery community involved in the development of new chemical entities for NTDs.

## Materials and methods

### Ethics statement

All animal experiments were performed according to institutional ethical guidelines for animal care. Mouse tolerability and pharmacokinetic studies (CIEnP, Florianópolis, Brazil) were approved by the internal animal ethical committee (217/01 and 264/00) and proof-of-concept acute VL studies (ICB/USP) were approved by the ethical committee of the Institute of Biomedical Sciences from University of São Paulo (178/2012/CEUA).

### Parasite and cell cultures

*Leishmania infantum* MHOM/MA(BE)/67/ITMAP263 amastigotes were collected from the spleen of infected donor Golden hamsters. Primary peritoneal mouse macrophages (PMM) were used as host cells for *in vitro* assays. PMM were collected from Swiss mice after 2 days of peritoneal stimulation with a 2% suspension of potato starch and maintained in RPMI-1640 medium supplemented with 200 mM L-glutamine, antibiotics and 5% FCS at 37°C and 5% CO_2_. Promastigotes of *L*. *infantum chagasi* (luciferase) (MHOM/BR/2005/NLC) obtained as described in Reimão et al.,[27] were grown in M199 medium supplemented with 10% FCS, 0.25% hemin, 32 μg/ml hygromycin and 2% sterile human urine at 25°C and 5% CO_2_.

### Compound solutions/dilutions

Stock solutions of the compounds at 20 mM were prepared in 100% DMSO. Next, the compounds were serially pre-diluted in DMSO followed by a further dilution in demineralized water to assure a final in-test DMSO concentration below 1%. The compounds were tested at 4-fold dilutions starting at 64 μM.

### *In vitro* parasitology assays

For the antileishmania *in vitro* assays, 3×10^4^ PMM/well were seeded in 96-well plates. After 24h, 5×10^5^ amastigotes/well were added and incubated for 2h at 37°C. Next, the compounds in 4-fold serial dilutions were added and the plates were further incubated for 5 days (37°C, 5% CO_2_). Parasite burdens were assessed using an inverted microscope after Giemsa staining. The results were expressed as percent reduction in parasite burdens compared to negative control wells (100% growth) and IC_50_ values were determined. Values represent the geometric mean of at least two independent experiments. Miltefosine was used as the positive control in all plates. Additional *in vitro* assays using a panel of VL and CL strains and clinical isolates were performed using comparable protocols.

### Experimental determination of distribution coefficient (eLogD)

To determine the lipophilicity of the compounds, a methodology based on the retention time of molecules in reverse stationary phase (Ascentis RP-Amide HPLC column) was used. The chromatogram was obtained using LC-MS/MS. Test compounds were prepared at 1.0 μg/mL by adding the stock solution at (1:1) mobile phases A:B + internal standard at 200nM (A: 5% methanol in 10mM ammonium acetate pH 7.4, B: 100% methanol), DMSO concentration must be lower than 2%. The lipophilicity of compounds was assessed by injecting individual test compounds and a series of eight commercial drugs for which LogD values has already been determined, covering a LogD range of -1.86 to 6.1. The retention time (in minutes) of each of the eight standards was plotted against their LogD values. The resulting equation for the calibration curve (y = mx + b) was used to calculate the LogD values for the test compounds.

### Human and mouse liver microsomal stability assay

The metabolic stability of the compounds was evaluated in human (pool of 200 donors, XenoTech) and mouse liver microsomes (CD1 mouse, GIBCO). Test compounds were prepared at a concentration of 0.5 μM and incubated with 0.25 mg/mL liver microsomes at pH 7.4 and 37°C. The reaction was started by addition of NADPH at 0.5 μM. Samples were taken at 0, 5, 10, 15, 20 and 30 minutes. The reaction was stopped by the addition of acetonitrile:methanol (1:1) containing an internal standard (tolbutamide at 50 nM). Compounds were quantified by liquid chromatography triple quadrupole mass spectrometry (LC-MS/MS). Peak area ratios (analyte/internal standard) were converted to % remaining using the area ratio at time 0 as 100%. Half-life (t_1/2_ = ln(2)/k) in minutes and intrinsic clearance (Cl_int_ = k x 1000/(0.25)) in μL/min/mg were calculated using a non-linear regression from % remaining *versus* incubation time. From this plot, the slope (k) was determined. The analysis conditions were: analytical column (Gemini C18, 5062.0 mm, 3 mm, Phenomenex), electrospray ionization source (ESI) in positive and negative mode, mobile phase A (water + 0.1% formic acid) and B (acetonitrile + 0.1% formic acid), flow rate of 0.7 mL/min.

### Parallel artificial membrane permeability assays (PAMPA)

To determine the passive permeability of the compounds, a 96-well plate containing membranes pre-coated with lipids (Corning Gentest # 353015) was used. The solutions of the compounds were prepared by diluting the stock solutions (10mM) in phosphate buffered saline (PBS) pH 6.5 at a final concentration of 10μM. The solutions diluted in PBS pH 6.5 were then added to the donor portion of the plate (300 μl/well), while PBS pH 7.4 (200 μl/well) was added to the acceptor portion. The two portions of the plate were then coupled, and the system was incubated for 5 h at 37°C. At the end of the incubation, samples were collected from the donor and acceptor plates, and then added to plates containing quench solution (10% water and 90% methanol: acetonitrile (50:50) + 50 nM tolbutamide). The final concentrations of compounds in the donor, acceptor, and initial solution (T0) wells were analyzed using LC-MS/MS. The results were used to calculate an effective permeability (Pe) value. The PAMPA assay was performed in triplicate (n = 3).

### Kinetic solubility

To determine kinetic solubility, 10 mM samples of each compound were transferred to a 96-well plate (incubation plate) in duplicate; for each sample on the plate, 195 μL of PBS buffer pH 7.4 or pH 2.0 (final concentration of 250 μM) was added; the plate was sealed and shaken for 24 ± 1 hour (200 rpm, r.t.). The precipitates on the incubation plate were removed by centrifugation (15 min, 3000 rpm, r.t.); the compound concentration in the resulting supernatant was determined by LC-MS/MS. Results were calculated as the concentration of remaining compound in solution after incubation.

### Plasma and medium protein binding

For plasma protein binding, pooled plasma was thawed in a water bath at 37°C and centrifuged at 4,000 rpm for 5 min to remove any clots. The pH value was measured and adjusted to 7.4, if necessary. RPMI medium (pH 7.4) supplemented with 10% fetal calf serum was used. Working solutions (400 μM) of test compounds were prepared by diluting appropriate volume of the stock solutions with DMSO. Working solutions (400 μM) were spiked into plasma to achieve 2 μM final concentrations as loading solutions. The concentration of organic solvent in the final solutions was 0.5% DMSO. The dialysis instrument was assembled following manufacturer’s instructions. Aliquots of loading solution were loaded in triplicate to the donor side of each dialysis well and dialyzed against an equal volume of dialysis buffer. The plate was shaken and incubated for 6 hours in a humidified incubator with 5% CO_2_ at 37°C. At the end of dialysis, aliquots of dialysate and retentate were removed into sample collection plates. Each sample was matched with an opposite blank buffer or plasma, with a ratio of matrix:dialysis buffer of 50:50 (v:v) in each well and participated with 500μL of stop solution (50% acetonitrile/methanol; internal standards—200 ng/mL tolbutamide, labetalol and 50 ng/mL metformin). All sample collection plates were rotated at 800 rpm for 5 min to mix samples and centrifuged at 20°C, 4000 rpm for 20 min. Aliquots of supernatant of all the samples were removed and injected into LC-MS/MS. Results were calculated as the percentage of remaining parent compound in plasma or medium after incubation.

### Plasma stability

Pooled plasma was thawed in a water bath at 37°C and centrifuged at 4,000 rpm for 5 min to remove any clots. Plasma solution was prepared by adding PBS (pH 7.4) in a 1:1 ratio. It was not necessary to adjust the pH of the final plasma solution. Plasma:PBS and test compound solutions were added to the incubation plate at a final concentration of 2 μM. The plate was incubated in a shaker at 37°C and 50 rpm. Samples were removed (25 μL) from each well of the incubation plate at multiple timepoints and added to a plate containing 100 μL of quench solution (200 ng/mL tolbutamide in 50% acetonitrile/methanol) to precipitate protein. The quenched aliquots were centrifuged at 4000 rpm for 10 min at 5°C. An aliquot of supernatant was transferred from each well to a plate for analysis by LC-MS/MS. Results were calculated as the percentage of remaining parent compound in plasma after incubation.

### hERG assay

This was adapted from the literature method;[28] briefly, hERG functional activity was measured in an inducible hERG T-RExTM-CHO Cell line (ThermoFisher #K1237) using thallium influx as a surrogate indicator of potassium ion channel activity. Thallium enhances the fluorescent signal of BTC-AM dye (ThermoFisher #B6791). 384 well plates were seeded at 15,000 cells/well with doxycycline hyclate, included to induce expression of the hERG channel, and grown for 48 hours. Media was removed and cells were loaded with 4 μM dye for 90 mins in a low potassium buffer, dye was then removed and compound added to the cells in a high potassium buffer in a 6 point 1:3 dilution dose series (maximum final concentration of 30 μM). After 30 mins of compound incubation, channel activity was recorded upon addition of thallium buffer using a Tetra plate reader. The slope of the kinetic read was used to calculate channel activity.

### CYP inhibition assay

These assays measure the ability of test compounds to inhibit cytochrome P450 activity for four isoforms CYP1A2, CYP2C9, CYP2D6 and CYP3A4, with a time dependent inhibition component for 3A4 only, in human liver microsomes, using luminescent and/or fluorescent assay kits. The test compound’s ability to inhibit the enzyme activity (IC_50_) was assessed by determining the extent of inhibition of metabolite formation relative to vehicle control (DMSO).

### Off-target panel

The compounds were evaluated across a panel of 20 liability targets (37 functional assays) which included functional cell-based GPCRs and ion channels in both agonist and antagonist readout, measuring calcium flux and biochemical functional assays for nuclear hormone receptors and phosphodiesterases using TR-FRET format, in a 6 point 1:3 dilution dose series (maximum final concentration of 10 μM).

### Mouse single-dose pharmacokinetics

Compounds were administered to groups (n = 6) of female BALB/c mice via single intravenous dosing (at 2 mg/kg) or single oral dosing (at 50 mg/kg) employing a solution/suspension vehicle comprising of 10% ethanol, 40% PEG_400_ and 0.4% Tween_80_ in saline buffer (pH adjusted to 9). Samples derived from plasma (at 0.083 for iv only, 0.25, 1, 2, 4, 8 and 24 h) were quantified by LC−MS/MS. Data analysis and calculation of pharmacokinetic parameters was performed using Phoenix WinNonlin version 7.0.

### Mouse 5-day tolerability

Compounds were administered to groups (n = 3) of female BALB/c mice via oral gavage in a 5-day exploratory repeat-dose study at doses of 25, 50 and 100 mg/kg/day, employing a solution/suspension vehicle comprising 10% ethanol, 40% PEG_400_ and 0.4% Tween_80_ in saline buffer (pH adjusted to 9). Repeat-dose toxicity was evaluated based on mortality, clinical observations, body weight and macroscopic organ pathologies.

### Acute visceral leishmaniasis mouse model

Groups of female BALB/c mice (n = 5) were infected with 10^8^ stationary phase promastigotes of *L*. *infantum chagasi*-LUC in a final volume of 100 μL by the intraperitoneal route. At day 14 post-infection, groups were orally treated for 5 days with either drug vehicle only (10% ethanol, 40% PEG_400_ and 0.4% Tween_80_ in saline buffer—pH adjusted to 9), miltefosine (40 mg/kg QD) or with compounds **29** and **39** (25 mg/kg QD). Light emission in the ventral abdominal cavity of infected animals was recorded by bioimaging (IVIS Spectrum, Caliper Life Sciences) on 14, 19- and 24-days post-infection. Before imaging, mice received 75 mg/kg VivoGlo™ Luciferin (Promega Corporation) (i.p.) and were anesthetized in a 2% isoflurane atmosphere (Cristália). Animals were then transferred to the imaging chamber and kept in a 2% isoflurane atmosphere. Emitted photons were collected using the high resolution (medium binning) mode. Total photon emission from a defined region of interest (ROI) corresponding to the whole abdominal area was registered. The same ROI was applied to all animals in any given group. Images were acquired 15 min after luciferin injection. Total photon emission from each mouse was quantified with Living Image software version 4.3.1 (Caliper Life Sciences), and results were expressed as the number of photons/s/ROI subtracted from the corresponding ROI in uninfected animals. The detected signal was presented as a pseudocolor image representing light intensity (red = most intense and blue = least intense) and superimposed on the gray scale reference image.

## Supporting information

S1 Data Complete biological dataset. (XLSX)

S1 Information Detailed synthetic and spectral information. (PDF)
