# Supplementary material for: 2-aminobenzimidazoles for leishmaniasis: From initial hit discovery to in vivo profiling
Source: PLoS Negl Trop Dis. 2021 Feb 22;15(2):e0009196. doi: 10.1371/journal.pntd.0009196 (PMC7932521; doi:10.1371/journal.pntd.0009196)
Supplement: S1 Information — (PDF) [file pntd.0009196.s002.pdf]

2-aminobenzimidazoles for leishmaniasis: from initial hit discovery to in vivo profiling

Rafael Augusto Alves Ferreira‡[1], Celso de Oliveira Rezende Junior‡[1], Pablo David Grigol Martinez‡[1], Paul John Koovits[1], Bruna Miranda Soares[1], Leonardo L. G. Ferreira[2], Simone Michelan-Duarte[2], Rafael Consolin Chelucci[2], Adriano D. Andricopulo[2], Mariana K. Galuppo[3], Silvia R. B. Uliana[3], An Matheeussen[4], Guy Caljon[4], Louis Maes[4], Simon Campbell[5], Jadel M. Kratz[5], Charles E. Mowbray[5], Luiz Carlos Dias[1]

[1]Institute of Chemistry, University of Campinas (UNICAMP), Campinas-SP, 13083-861, Brazil

[2]Laboratory of Medicinal and Computational Chemistry, Physics Institute of São Carlos, University of São Paulo (USP), São Carlos-SP, 13563-120, Brazil

[3]Department of Parasitology, Biomedical Sciences Institute, University of São Paulo (USP), São Paulo-SP, 05508-000, Brazil

[4]Laboratory of Microbiology, Parasitology and Hygiene (LMPH), Universiteitsplein 1, 2610 Antwerpen, Belgium

[5]Drugs for Neglected Diseases *initiative* (DND*i*), 15 Chemin Louis-Dunant, 1202 Geneva, Switzerland

‡ These authors contributed equally

*To whom correspondence should be addressed. L.C.D.: telephone: + 55 19 3521 3097; email: ldias@unicamp.br

SUPPORTING INFORMATION

Experimental

Unless noted, all reactions were performed under an atmosphere of argon with dry solvents and magnetic stirring. Dichloromethane (DCM) and triethylamine (Et_3N) were distilled from CaH_2. Tetrahydrofuran (THF) was distilled from sodium/benzophenone. Dimethyl formamide (DMF) was purchased from Aldrich (anhydrous) and used without further purification. Yields refer to homogeneous materials obtained after purification of reaction products by flash column chromatography using silica gel (200-400 mesh), liquid-liquid extraction or recrystallization. Analytical thin-layer chromatography was performed on silica-gel 60 and GF (5-40 μm thickness) plates, and visualization was accomplished using UV light, basic potassium permanganate staining or ninhydride solution followed by heating. ^{1}H and proton-decoupled ^{13}C NMR spectra were acquired in $CDCl_3$, CD_3OD or d_6-DMSO at 250 MHz (^{1}H) and 62.5 MHz (^{13}C) (Bruker DPX250), at 400 MHz (^{1}H) and 100 MHz (^{13}C) (Bruker Avance 400), at 500 MHz (^{1}H) and 125 MHz (^{13}C) (Varian Inova 500), or at 600 MHz (^{1}H) and 150 MHz (^{13}C) (Bruker Avance 600). Chemical shifts (δ) are reported in ppm using residual undeuterated solvent as an internal standard ($CDCl_3$ at 7.26 ppm, CD_3OD at 3.31 ppm, d_6-DMSO at 2.50 ppm, and TMS at 0.00 ppm for ^{1}H NMR spectra and $CDCl_3$ at 77.16 ppm, CD_3OD at 49.0 ppm, d_6-DMSO at 39.52 ppm for ^{13}C NMR spectra). Multiplicity data are reported as follows: s = singlet, d = doublet, t = triplet, q = quartet, br s = broad singlet, dd = doublet of doublets, dt = doublet of triplets, ddd = doublet of doublet of doublets, tt = triplet of triplets, app d = apparent doublet, app t = apparent triplet, m = multiplet, and br m = broad multiplet. The multiplicity is followed by the coupling constant(s) in Hz and integration. High resolution mass spectrometry (HRMS) was measured using electrospray ionization (ESI)

(waters xevo Q-tof, thermo LTQ-FT ultra, or thermos Q exactive) or using electron ionization (EI) (GCT premier waters).

File named Biological Dataset_09_09_2020.xlsx file should be included as a Supporting File for this manuscript

Methods and conditions

Method A: Nucleophilic substitution reactions with amines

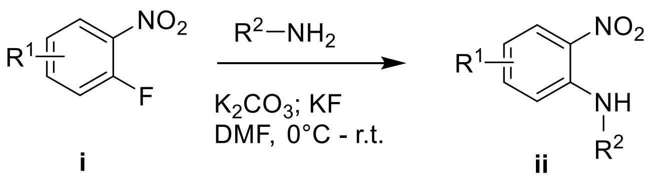

To a stirring solution of 2-fluoronitrobenzene derivatives (i) in DMF (concentration of 0.5 mol.L^{-1}) was added K_2CO_3 (1.0 equiv.), KF (1.0 equiv.) and corresponding primary amines (x equiv.) at 0°C. The reaction was stirred at room temperature until the consumption of the 2-fluoronitrobenzene. Water (15-fold DMF amount) was added at room temperature and the formed solid was filtered and washed with water. The solid was dried in the high vacuum, generating the desired aniline. When a solid was not formed after water addition, the solution was extracted with diethyl ether. The organic layer was dried with magnesium sulfate, filtered e evaporated, giving the aniline product.[1]

Method B: Reduction reaction of nitro groups

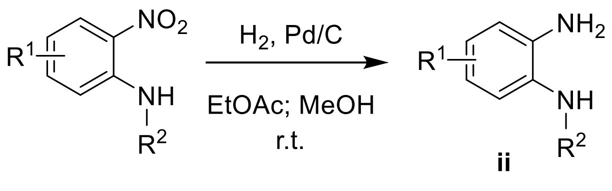

To a stirring solution of nitrobenzene derivatives in mixture of EtOAc and MeOH (1:1, concentration of 0.2 mol.L^{-1}) was added 10% Pd/C (10 mol %) at room temperature.

Hydrogen gas (1 bar) was added until the consumption of the nitrobenzene derivative. The solution was filtered through celite and evaporated under reduced pressure, generating the phenylenediamine derivatives (ii).

Method C: Construction of aminobenzimidazole fragment

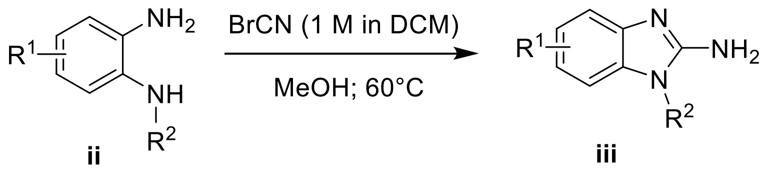

To a stirring solution of phenylenediamine derivatives (ii) in methanol (concentration of 1.0 $mol.L^{-1}$) in a sealed tube, was added a 1.0 $mol.L^{-1}$ fresh solution of cyanogen bromine (1.5 equiv.) at room temperature. The reaction was stirred at 60°C until the consumption of phenylenediamine derivative. The reaction was quenched at room temperature with a solution of NaOH (2.0 $mol.L^{-1}$). The organic layer was separated and washed with brine. The DCM layer was dried with magnesium sulfate, filtered and evaporated under reduced pressure, giving the desired aminobenzimidazole fragment (iii).[2]

Method D: Amidation reaction

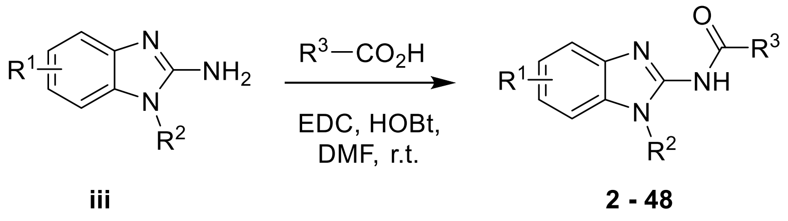

To a stirring solution of aminobenzimidazole (iii) in a minimal possible amount of DMF was added EDC (1.2 equiv.), HOBt (1.0 equiv.) and the corresponding carboxylic acid (1.1 equiv.) at room temperature. The reaction was stirred until the consumption of aminobenzimidazole (iii). Excess of water was added and the mixture was stirred for 15 minutes at room temperature. The solid was filtered and washed with water and dried in

the high vacuum, generating the desired acyl aminobenzimidazole **2-48**. A flesh chromatography column was performed for some final compounds.

Method E: Nucleophilic substitution reactions with ammonium chloride salts.

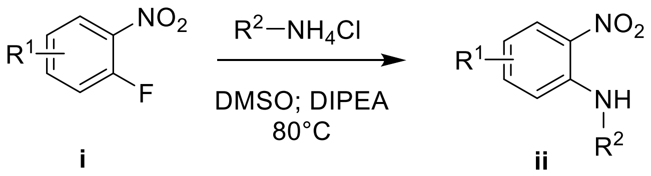

To a stirring solution of 2-fluoronitrobenzene derivatives in DMSO (0.1 mol.L^{-1}) was added the ammonium chloride salt (2.0 equiv.) and DIPEA (4.0 equiv.) in a sealed tube. The reaction was stirred at 80°C until the consumption of the 2-fluoronitrobenzene. Water (15-fold DMSO amount) was added at room temperature and the formed solid was filtered and washed with water. The solid was dried in the high vacuum, generating the desired aniline. giving the aniline product.

$$KCN + Br_2 \xrightarrow[r.t.]{H_2O} Br{-}C{\equiv}N$$

The solution of cyanogen bromine was prepared as reported in the literature.[3]

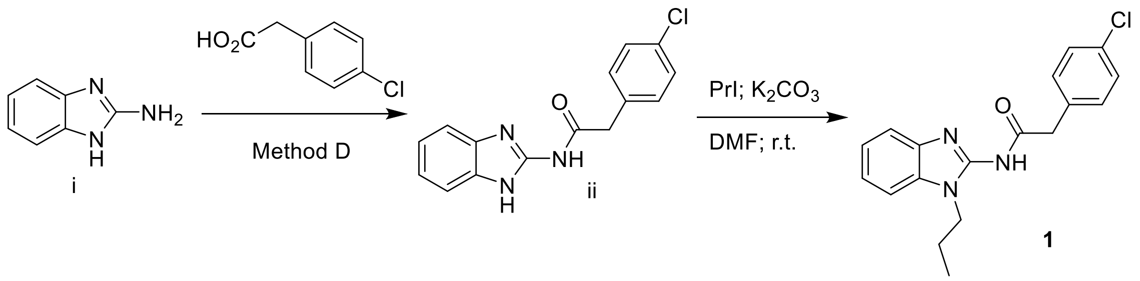

The compound ii was prepared in 73% yield using the Method D. To a solution of ii (80 mg; 0.28 mmol) in DMF (2 mL) was added potassium carbonate (58 mg; 0.42 mmol) and *n*-propyl iodide (41 μL; 0.42 mmol) at room temperature. After 19 h at room temperature was added water (6 mL). The mixture was extracted with EtOAc (3 x 5 mL), the organic

layer was dried with magnesium sulfate, filtered and evaporated under reduced pressure. The residue was purified by flash chromatographic column (1 EtOAc : 1 Hex) giving **1** in 78% yield.

^{1}H NMR (400 MHz, $CDCl_3$) δ 12.10 (s, 1H), 7.57 – 7.13 (m, 8H), 4.28 – 3.89 (m, 2H), 3.77 (s, 2H), 1.90 – 1.77 (m, 2H), 0.97 (t, *J* = 7.4 Hz, 3H). **^{13}C NMR (101 MHz, $CDCl_3$)** δ 183.05, 153.51, 136.14, 132.02, 130.91, 129.53, 128.22, 128.07, 123.00, 122.89, 111.16, 109.35, 46.86, 43.66, 21.69, 11.30.

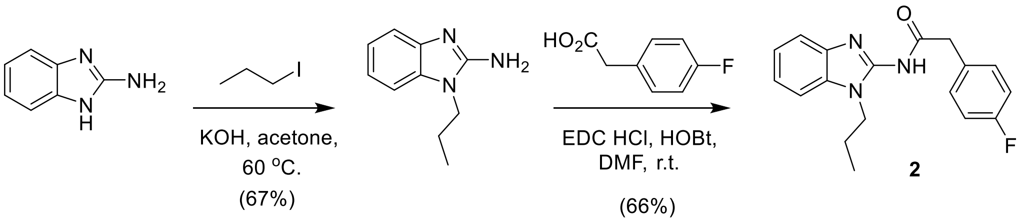

To a stirred solution of 2-aminobenzimidazole (531 mg; 4 mmol) in acetone PA (20 mL), KOH (450 mg; 8 mmol) and then Pr-I (0.39 mL; 4 mmol) were added. After 3 h at 60 °C (oil bath temperature) the volatiles were removed and water (1 mL) was added. The product was extracted with AcOEt (4 x 8 mL), dried over Na_2SO_4 and purified by flash column chromatography (DCM:MeOH 9:1) to yield 468 mg (67%) of the desired alkylated compound. Followed by method D.

^{1}H NMR (250 MHz, $CDCl_3$) δ 7.35 (dd, *J* = 8.7, 5.5 Hz, 2H), 7.28 – 7.16 (m, 4H), 6.99 (t, *J* = 8.8 Hz, 2H), 4.17 – 3.94 (m, 2H), 3.75 (s, 2H), 1.81 (sext, *J* = 7.4 Hz, 2H), 0.95 (t, *J* = 7.4 Hz, 3H). **^{13}C NMR (126 MHz, $CDCl_3$)** δ 183.04, 161.67 (d, *J* = 243.7 Hz), 153.30, 133.42, 131.01 (d, *J* = 7.7 Hz), 129.59, 128.44, 122.92 (d, *J* = 13.5 Hz), 114.91 (d, *J* = 21.2 Hz), 111.36, 109.34, 46.72, 43.69, 21.74, 11.35. **HRMS** m/z calculated for $C_{18}H_{19}FN_3O$ $[M+H]^+$ 312.1507, found 312.1503.

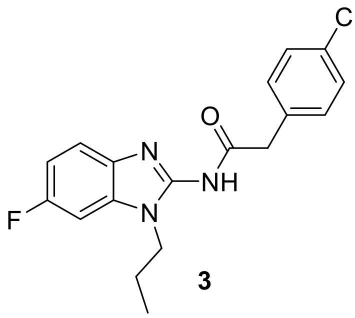

3

Method A, followed by B, C and D.

69% overall yield. **^{1}H NMR (400 MHz, $CDCl_3$)** δ 12.08 (s, 1H), 7.35-7.28 (m, 4H), 7.17 (dd, *J* = 8.7, 4.4 Hz, 1H), 6.99-6.91 (m, 2H), 4.03 (t, *J* = 7.3 Hz, 2H), 3.76 (s, 2H), 1.88-1.76 (m, 2H), 0.97 (t, *J* = 7.4 Hz, 3H). **^{13}C NMR (101 MHz, $CDCl_3$)** δ 160.71, 158.32, 135.94, 132.10, 130.89, 128.26, 124.46, 124.38, 111.81, 111.68, 110.24, 109.99, 97.33, 97.04, 46.80, 43.90, 21.55, 11.26. **HRMS** m/z calculated for $C_{18}H_{18}ClFN_3O$ $[M+H]^+$ 346.1117, found 346.1137.

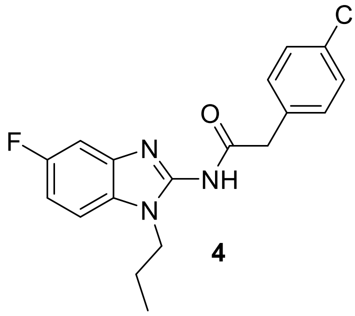

4

Method A, followed by B, C and D.

62% overall yield. **^{1}H NMR (400 MHz, $CDCl_3$)** δ 12.07 (s, 1H), 7.36 – 7.24 (m, 4H), 7.14 (dd, *J* = 8.7, 4.3 Hz, 1H), 7.05 – 6.92 (m, 2H), 4.05 (t, *J* = 7.3 Hz, 2H), 3.73 (s, 2H), 1.95 – 1.71 (m, 2H), 0.95 (t, *J* = 7.4 Hz, 3H); **^{13}C NMR (126 MHz, $CDCl_3$)** δ 160.31, 158.39, 153.94, 135.85, 130.87, 128.28, 126.19, 110.42, 110.22, 109.81, 109.73, 99.47, 46.65, 43.96, 21.74, 11.30. **HRMS** m/z calculated for $C_{18}H_{18}ClFN_3O$ $[M+H]^+$ 346.1117, found 346.1137.

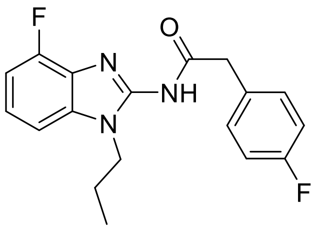

5

Method E, followed by B, C and D.

37% overall yield. **^{1}H NMR (250 MHz, CDCl$_3$)** δ 7.34 (dd, *J* = 8.6, 5.5 Hz, 2H), 7.16 (td, *J* = 8.2, 4.8 Hz, 1H), 7.07 – 6.88 (m, 4H), 4.09 – 3.99 (m, 2H), 3.76 (s, 2H), 1.81 (sext, *J* = 7.3 Hz, 2H), 0.94 (t, *J* = 7.4 Hz, 3H). **^{13}C NMR (101 MHz, CDCl$_3$)** δ 180.65 , 161.91 (d, *J* = 244.3 Hz), 151.72 , 149.54 (d, *J* = 246.9 Hz), 133.63 , 132.37 , 131.07 (d, *J* = 7.9 Hz), 123.52 (d, *J* = 6.9 Hz), 119.67, 115.21 (d, *J* = 21.2 Hz), 109.17 (d, *J* = 16.9 Hz), 105.71 (d, *J* = 3.0 Hz), 45.59 , 44.94 , 21.85 , 11.38 . **HRMS** m/z calculated for $C_{18}H_{17}F_2N_3NaO^+$ $[M+Na]^+$ 352.1232, found 352.1238.

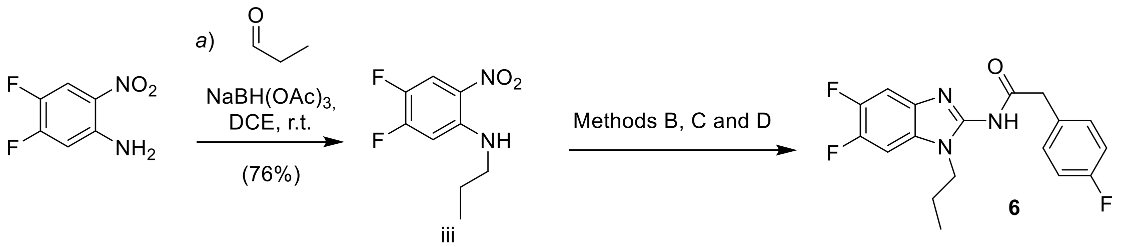

To a solution of nitroaniline (350 mg; 2 mmol) in DCE (2.4 mL), propanal (0.30 mL; 4.2 mmol) was added. After 10 min at rt, NaBH(OAc)3 (1189 mg; 5.6 mmol) was added in five portions for 10 min. After stiring for 24 h at rt, DCE (4 mL), propanal (0.30 mL; 4.2 mmol) and NaBH(OAc)3 (1165 mg; 5.5 mmol) were added again. The reaction mixture was allowed to stir additional 36 h and then quenched with NaOH 2 M (5 mL). The product was extracted with DCM, dried over $MgSO_4$, concentrated and purified by flash column chromatography (Hexanes:AcOEt 9:1) to yield 329 mg (76%) of the desired compound. Followed by methods B, C and D.

24% overall yield. **^{1}H NMR (600 MHz, $CDCl_3$)** δ 12.19 (bs, 1H), 7.33 (dd, *J* = 8.3, 5.6 Hz, 2H), 7.08 (t, *J* = 7.4 Hz, 1H), 7.03 (dd, *J* = 9.5, 6.6 Hz, 1H), 6.99 (t, *J* = 8.7 Hz, 2H), 4.00 (t, *J* = 7.2 Hz, 2H), 3.74 (s, 2H), 1.83 – 1.74 (m, 2H), 0.94 (t, *J* = 7.4 Hz, 3H). **^{13}C NMR (151 MHz, $CDCl_3$)** δ 161.85 (d, *J* = 244.3 Hz), 147.79 (dd, *J* = 243.2, 14.3 Hz), 147.50 (dd, *J* = 243.3, 14.2 Hz), 131.06 (d, *J* = 7.7 Hz), 115.14 (d, *J* = 21.1 Hz), 100.69 (bs), 98.67 (d, *J* = 23.8 Hz), 46.59, 44.24, 21.71, 11.38. **HRMS** m/z calculated for $C_{18}H_{16}F_3N_3ONa^+$ $[M+Na]^+$ 370.1143, found 370.1134.

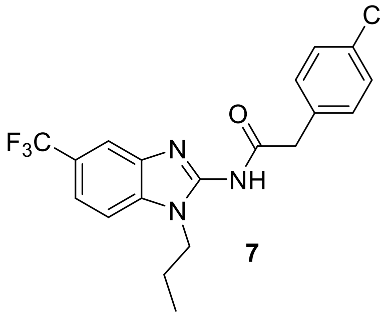

7

Method A, followed by B, C and D. 40% overall yield.

29% overall yield. **^{1}H NMR (500 MHz, $CDCl_3$)** δ 7.66 – 7.40 (m, 2H), 7.46 – 7.15 (m, 5H), 4.10 (t, *J* = 7.2 Hz, 2H), 3.79 (s, 2H), 1.89 – 1.83 (m, 2H), 1.03 – 0.97 (m, 3H). **^{13}C NMR (126 MHz, $CDCl_3$)** δ 154.06, 135.60, 132.25, 130.91, 129.59, 128.73, 128.33, 125.75, 125.49, 125.29, 125.23, 120.32, 120.19, 111.40, 109.36, 108.69, 106.64, 46.76, 44.05, 21.66, 11.27. **HRMS** m/z calculated for $C_{19}H_{18}ClF_3N_3O+$ $[M+H]^+$ 396.1085, found 396.1088.

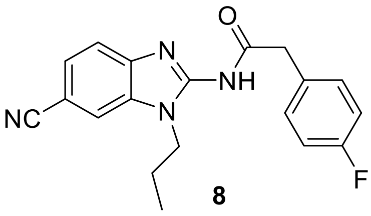

8

Method E, followed by B, C and D.

62% overall yield. **^{1}H NMR (250 MHz, $CDCl_3$)** δ 12.28 (s, 1H), 7.51 (s, 1H), 7.47 (s, 1H), 7.37-7.26 (m, 3H), 7.00 (t, *J* = 8.7 Hz, 1H), 4.05 (t, *J* = 7.2 Hz, 2H), 3.76 (s, 2H), 1.92 – 1.71 (m, 2H), 0.96 (t, *J* = 7.4 Hz, 3H). **^{13}C NMR (126 MHz, DMSO-d_6)** δ 170.89, 161.09 (d, *J* = 240.4 Hz), 148.24, 143.90, 133.77, 131.10 (d, *J* = 7.9 Hz), 126.64, 125.27, 119.80, 119.47, 115.46, 115.04, 103.71, 44.85, 41.21, 21.86, 10.78. **HRMS** m/z calculated for $C_{19}H_{17}FN_4ONa^+$ $[M+Na]^+$ 359.1279, found 359.1268.

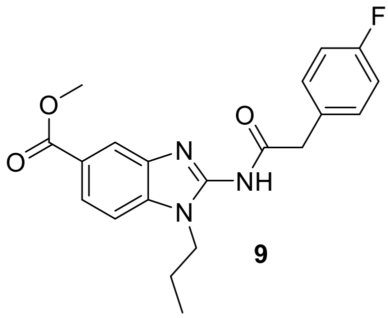

9

Method A, followed by B, C and D.

73% overall yield. **^{1}H NMR (500 MHz, $CDCl_3$)** δ 8.05 – 7.89 (m, 2H), 7.37 (dd, *J* = 8.5, 5.5 Hz, 2H), 7.33 – 7.24 (m, 1H), 7.04 (dt, *J* = 17.2, 8.7 Hz, 2H), 4.16 – 4.09 (m, 2H), 3.98 (s, 3H), 3.79 (s, 2H), 1.89 – 1.85 (m, 2H), 1.01 – 0.98 (m, 3H). **^{13}C NMR (126 MHz, $CDCl_3$)** δ 183.57, 166.64, 162.67, 160.73, 154.20, 132.93, 130.99, 125.16, 124.96, 115.04, 114.87, 110.88, 108.78, 52.33, 46.64, 43.88, 21.67, 11.27.

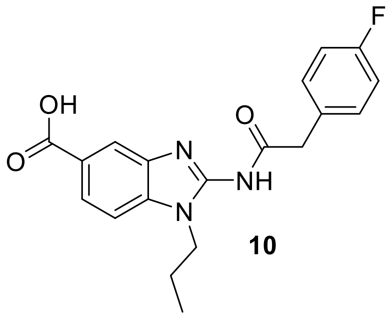

10

To a stirring solution of methyl ester **9** (1.3 g; 3,52 mmol) in MeOH and water (5:20 mL) was added a solution of NaOH (4.0 mol.L^{-1}, 6,0 equiv.) at 0° C. The reaction was stirred

at room temperature for 2h. The solution was acidified with HCl (2.0 mol.L^{-1}) until pH ~2-3 at 0°C. The formed solid was filtered, washed with cold water and dried in the high vacuum, giving the carboxilic acid **10** in quantitative yield.

^{1}H NMR (400 MHz, DMSO) δ 13.11 (s, 1H), 8.29 (s, 1H), 7.99 (d, *J* = 8.4 Hz, 1H), 7.72 (d, *J* = 8.5 Hz, 1H), 7.47 (dd, *J* = 8.5, 5.7 Hz, 2H), 7.19 (t, *J* = 8.9 Hz, 2H), 4.56 (s, 2H), 4.12 (s, 2H), 1.75 (dt, *J* = 14.6, 7.3 Hz, 2H), 0.92 (t, *J* = 7.3 Hz, 3H**)**. **^{13}C NMR (101 MHz, DMSO)** δ 167.39, 162.88, 160.55, 131.89, 131.43, 130.22, 127.26, 126.01, 115.61, 115.40, 114.74, 113.22, 45.39, 22.16, 11.08.

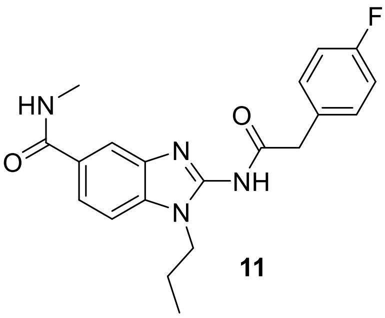

11

Method D, using the compound **10** as carboxilic acid. Methylammonium hydrochloride and triethylamine were used as amine source.

38% yield. **^{1}H NMR (500 MHz, $CDCl_3$)** δ 7.81 (s, 1H), 7.52 (dd, *J* = 8.3, 1.3 Hz, 1H), 7.35 (dd, *J* = 8.4, 5.6 Hz, 2H), 7.24 (d, *J* = 8.2 Hz, 1H), 7.01 (t, *J* = 8.7 Hz, 2H), 6.29 (d, *J* = 3.8 Hz, 1H), 4.13 – 4.03 (m, 2H), 3.77 (s, 2H), 3.05 (d, *J* = 4.8 Hz, 3H), 1.83 (dd, *J* = 14.7, 7.4 Hz, 2H), 0.96 (t, *J* = 7.4 Hz, 3H). **^{13}C NMR (126 MHz, $CDCl_3$)** δ 183.34, 167.65, 162.66, 160.72, 153.97, 132.94, 130.97, 130.91, 130.39, 129.95, 129.87, 120.95, 115.03, 114.86, 110.76, 109.28, 46.57, 43.89, 27.04, 21.66, 11.21. **HRMS** m/z calculated for $C_{20}H_{22}FN_4O_2$ $[M+H]^+$ 369.1721, found 369.1726.

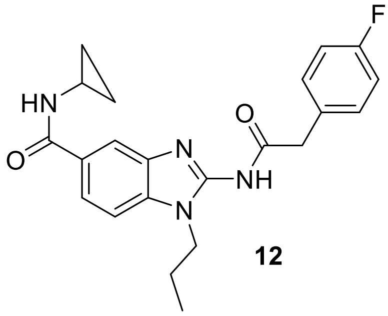

12

Method D, using the compound **10** as carboxilic acid.

58% yield. **^{1}H NMR (500 MHz, DMSO)** δ 10.48 (s, 1H), 8.11 (s, 1H), 7.89 (s, 1H), 7.69 (d, *J* = 8.3 Hz, 1H), 7.51 (d, *J* = 6.4 Hz, 1H), 7.45 – 7.36 (m, 2H), 7.10 (t, *J* = 8.1 Hz, 2H), 4.03 (t, *J* = 6.8 Hz, 2), 2.89 (dd, *J* = 13.0, 9.1 Hz, 1H), 1.73 (d, *J* = 6.6 Hz, 2H), 0.83 (s, 3H), 0.73 (d, *J* = 4.9 Hz, 2H), 0.63 (d, *J* = 2.5 Hz, 2H). **HRMS** m/z calculated for $C_{22}H_{24}FN_4O_2$ $[M+H]^+$ 395.1878, found 395.1884.

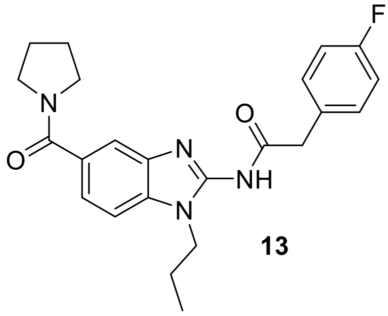

13

Method D, using the compound **10** as carboxilic acid.

44% yield. **^{1}H NMR (500 MHz, $CDCl_3$)** δ 12.17 (s, 1H), 7.48 (s, 1H), 7.41 – 7.34 (m, 3H), 7.22 (d, *J* = 8.2 Hz, 1H), 7.01 (t, *J* = 8.7 Hz, 2H), 4.07 (t, *J* = 7.3 Hz, 2H), 3.77 (s, 2H), 3.68 (t, *J* = 6.7 Hz, 2H), 3.48 (t, *J* = 6.4 Hz, 2H), 2.05 – 1.94 (m, 2H), 1.94 – 1.88 (m, 2H), 1.82 (dt, *J* = 14.7, 7.4 Hz, 2H), 1.00 – 0.90 (m, 3H). **^{13}C NMR (126 MHz, $CDCl_3$)** δ 183.40, 169.16, 162.63, 160.69, 153.97, 133.09, 132.19, 130.98, 130.92, 129.52, 122.09, 114.99, 114.82, 110.45, 109.08, 49.96, 46.64, 46.51, 43.80, 26.50, 24.45,

21.66, 11.26. **HRMS** m/z calculated for $C_{23}H_{26}FN_4O_2$ $[M+H]^+$ 409.2034, found 409.2044.

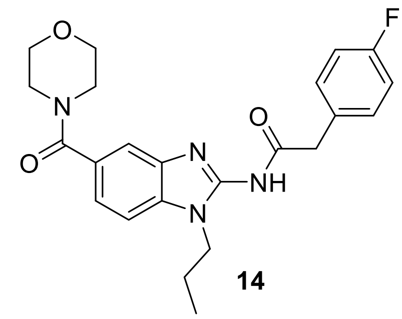

14

Method D, using the compound **10** as carboxilic acid.

50% yield. ^{1}**H NMR (500 MHz, DMSO)** δ 12.55 (s, 0.5H), 10.81 (s, 0.5H), 7.58 (s, 2H), 7.39 (s, 2H), 7.23 (dd, *J* = 8.2, 1.3 Hz, 1H), 7.16 (s, 2H), 4.02 (m, 2H), 3.80 – 3.35 (m, 8H), 1.60 (s, 2H), 0.91 – 0.68 (m, 3H). **HRMS** m/z calculated for $C_{23}H_{26}FN_4O_3$ $[M+H]^+$ 425.1983, found 395.1884.

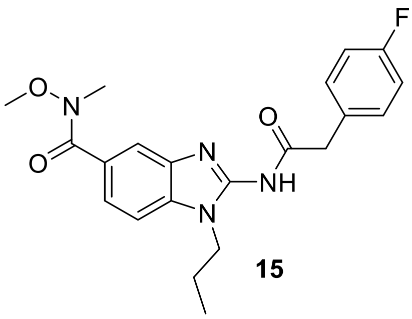

15

Method D, using the compound **10** as carboxilic acid. *N*,*O*-dimethylhydroxylammonium hydrochloride and triethylamine were used as amine source.

71% yield. ^{1}**H NMR (500 MHz, $CDCl_3$)** δ 7.66 (d, *J* = 0.7 Hz, 1H), 7.64 (dd, *J* = 8.3, 1.4 Hz, 1H), 7.36 (dd, *J* = 8.6, 5.5 Hz, 2H), 7.25 (d, *J* = 8.3 Hz, 1H), 7.01 (t, *J* = 8.8 Hz, 2H), 4.16 – 4.05 (m, 2H), 3.78 (s, 2H), 3.56 (s, 3H), 3.41 (s, 3H), 1.91 – 1.79 (m, 2H), 0.97 (t, *J* = 7.4 Hz, 3H). ^{13}**C NMR (126 MHz, $CDCl_3$)** δ 183.31, 169.11, 162.65, 160.71, 153.94, 133.04, 130.98, 130.91, 130.25, 129.24, 128.70, 125.52, 123.72, 115.00, 114.84, 110.46,

110.21, 61.06, 46.59, 43.83, 33.84, 21.68, 11.26. **HRMS** m/z calculated for $C_{21}H_{24}FN_4O_3$ $[M+H]^+$ 399.1827, found 399.1830.

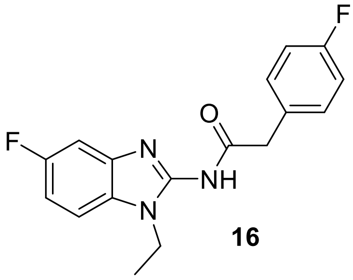

16

Method A, followed by B, C and D.

57% overall yield. **^{1}H NMR (500 MHz, CDCl$_3$)** δ 7.34 (m, 2H), 7.14 (dd, *J* = 8.7, 4.1 Hz, 1H), 6.99 (m, 4H), 4.14 (q, *J* = 7.2 Hz, 2H), 3.75 (s, 2H), 1.36 (t, *J* = 7.2 Hz, 3H). **^{13}C NMR (126 MHz, CDCl$_3$)** δ 182.9, 162.6, 160.7, 160.3, 158.4, 153.3, 130.9, 115.0, 110.3, 109.6, 99.2, 46.4, 37.3, 13.5. **HRMS** m/z calculated for $C_{17}H_{15}F_2N_3O$ $[M+H]^+$ 316.1256, found 316.1262.

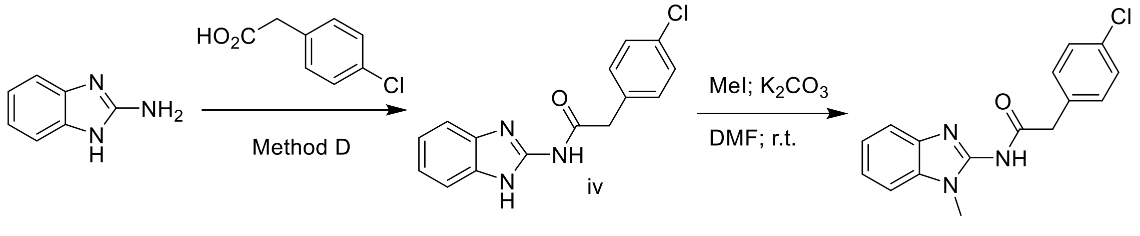

17

The compound iv was prepared in 73% yield using the Method D. To a solution of iv (100 mg; 0.35 mmol) in DMF (2 mL) was added potassium carbonate (96,6 mg; 0.7 mmol) and methyl iodide (64.6 mg; 0.46 mmol) at room temperature. After 16 h at room temperature was added water (6 mL). The mixture was extracted with EtOAc (3 x 5 mL), the organic layer was dried with magnesium sulfate, filtered and evaporated under reduced pressure. The residue was purified by flash chromatographic column (2 EtOAc : 1 Hex) giving **17** in 72% yield.

62% overall yield. **^{1}H NMR (400 MHz, $CDCl_3$)** δ 12.04 (s, 1H), δ 7.44 – 7.03 (m, 8H), 3.77 (s, 2H), 3.63 (s, 3H). **^{13}C NMR (101 MHz, $CDCl_3$)** δ 182.99, 153.71, 136.01, 132.26, 131.01, 130.17, 128.45, 128.27, 123.29, 111.31, 109.21, 77.48, 77.16, 76.84, 46.85, 28.37, 0.13. **HRMS** m/z calculated for $C_{19}H_{18}F_2N_3O$ $[M+H]^+$ 300.0898, found 300.0908.

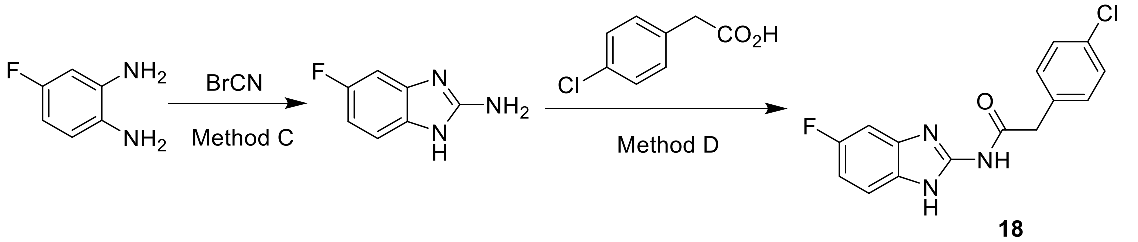

18

Method C, followed by D.

72% overall yield. **^{1}H NMR (500 MHz, DMSO)** δ 12.15 (s, 1H), 11.87 (s, 1H), 7.45 – 7.38 (m, 5H), 7.22 (dd, *J* = 9.6, 2.5 Hz, 1H), 6.96 – 6.90 (m, 1H), 3.81 (s, 2H). **^{13}C NMR (126 MHz, DMSO)** δ 170.52, 159.97, 157.99, 148.20, 141.77, 137.84, 134.56, 132.03, 131.64, 129.69, 128.80, 118.26, 112.61, 109.31, 103.14, 98.70, 41.88. **HRMS** m/z calculated for $C_{16}H_{15}ClN_3O$ $[M+H]^+$ 304.0647, found 304.0647.

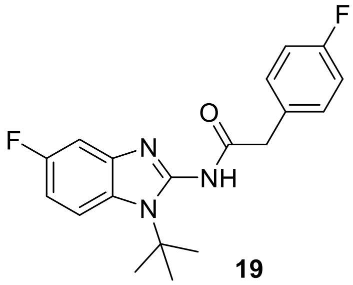

19

Method A, followed by B, C and D.

53% overall yield. **^{1}H NMR (500 MHz, $CDCl_3$)** δ 12.91 (s, 1H), 7.56 (dd, J = 9.2, 4.3 Hz, 1H), 7.30 (dd, J = 8.4, 5.6 Hz, 2H), 6.99 (m, 2H), 6.93 (dd, J = 7.9, 2.5 Hz, 1H), 6.85 (td, J = 9.2, 2.5 Hz, 1H), 3.72 (s, 2H), 1.80 (s, 9H). **^{13}C NMR (126 MHz, $CDCl_3$)**

δ 162.6, 160.6, 159.7, 157.8, 131.0, 114.9, 114.3, 109.6, 61.4, 47.1, 29.8. **HRMS** m/z calculated for $C_{19}H_{19}F_2N_3O$ $[M+H]^+$ 344.1569, found 344.1573.

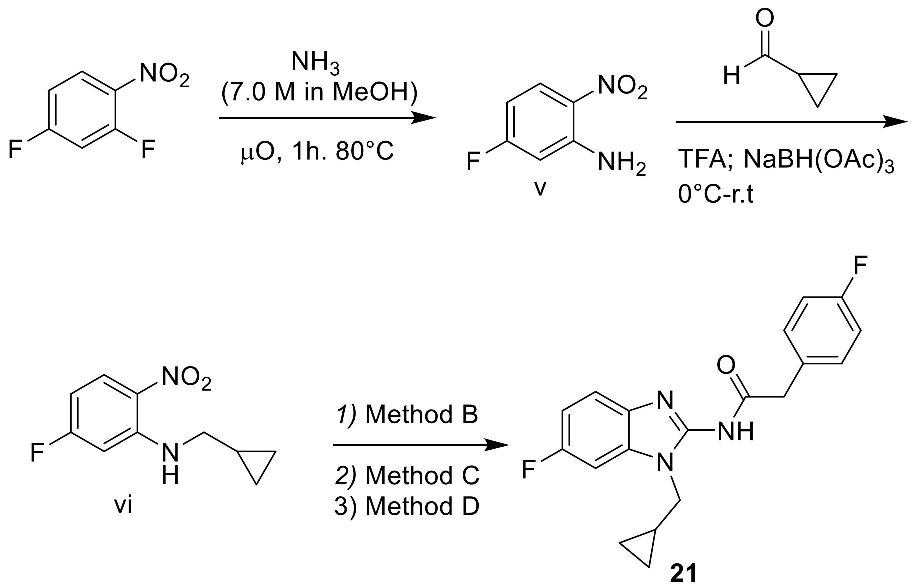

2,4-difluoronitrobenzene (1.45 g; 9.1 mmol) was added to a solution of ammonia (2 mL; 7.0 mol.L^{-1}). The reaction was stirred in a microwave reactor at 80°C for 1h. The solution was evaporated and the crude was purified by flash cromatographic column, generating the aniline v in 92% yield. To a solution of v (1.52 g; 9.74 mmol) in TFA (10 mL) was slowly added $NaBH(OAc)_3$ (4.13 g; 19.5 mmol) at 0°C. The mixture was stirred at 0°C for 15 minutes. Cyclopropanecarbaldehyde (0.73 mL; 9.74 mmol) was slowly added at 0°C. After 3h at room temperature, the solution was verted into an ice bath. The formed solid was filtered, washed with cold water and dried under high vacum, giving the compound vi in quantitaive yield.[4] **21** was prepared from vi using the Method B, followed for C and D in 23% yield over these three last steps.

^{1}H NMR (500 MHz, $CDCl_3$) δ 7.35 (dd, *J* = 8.5, 5.5 Hz, 2H), 7.20 (dd, *J* = 8.6, 4.4 Hz, 1H), 7.06 – 7.00 (m, 3H), 6.99 – 6.93 (m, 1H), 3.97 (d, *J* = 7.1 Hz, 2H), 3.76 (s, 2H), 1.31 – 1.16 (m, 1H), 0.61 – 0.51 (m, 2H), 0.45 (q, *J* = 4.8 Hz, 2H). **^{13}C NMR (126 MHz, $CDCl_3$)** δ 183.11, 162.65, 160.71, 160.50, 158.59, 154.60, 133.10, 133.08, 130.96,

130.90, 130.33, 130.23, 124.71, 115.05, 114.88, 111.90, 111.82, 110.27, 110.07, 97.44, 97.21, 46.73, 10.08, 4.07. **HRMS** m/z calculated for $C_{19}H_{18}F_2N_3O$ [M+H]$^+$ 342.1412, found 342.1416.

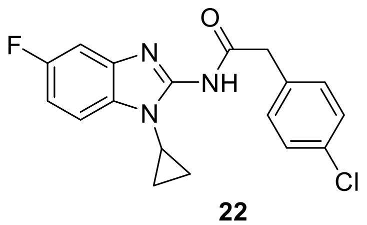

22

Method E, followed by B, C and D.

9% overall yield. **^{1}H NMR (400 MHz, CDCl$_3$)** δ 11.89 (bs, 1H), 7.35 (dd, *J* = 8.7, 4.4 Hz, 1H), 7.33 – 7.23 (m, 4H), 7.06 (d, *J* = 7.8 Hz, 1H), 6.98 (td, *J* = 9.3, 2.1 Hz, 1H), 3.79 (s, 2H), 3.16 (s, 1H), 1.08 (d, *J* = 6.2 Hz, 2H), 0.95 (s, 2H). **^{13}C NMR (126 MHz, CDCl$_3$)** δ 176.9 (bs), 159.62 (d, *J* = 239.5 Hz), 152.25 (bs), 134.52 (bs) , 132.81, 130.96, 128.90 (bs), 128.68, 111.14 (d, *J* = 9.7 Hz), 110.54 (d, *J* = 25.2 Hz), 101.46 (bs), 45.24, 24.90, 6.84. **HRMS** m/z calculated for $C_{18}H_{16}ClFN_3O^+$ [M+H]$^+$ 344.0960, found 344.0953.

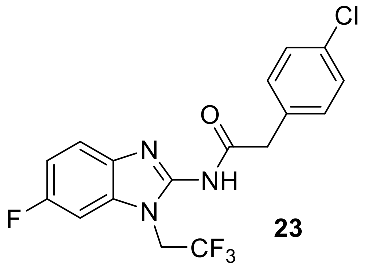

23

LOLA237 – crj368

Method E, followed by B, C and D.

52% overall yield. **^{1}H NMR (500 MHz, CDCl$_3$)** δ 12.00 (s, 1H), 7.28 (dd, *J* = 13.6, 9.6 Hz, 4H), 7.17 (dd, *J* = 8.0, 4.0 Hz, 1H), 7.04 – 6.90 (m, 2H), 4.69 (q, *J* = 8.5 Hz, 2H), 3.74 (s, 2H). **^{13}C NMR (126 MHz, CDCl$_3$)** δ 183.57, 160.86, 158.93, 154.81, 135.50, 132.49, 131.00, 128.51, 126.92, 124.69, 122.46, 120.23, 112.14, 111.49, 111.30, 98.08,

97.85, 46.90, 43.89, 43.60, 43.31, 43.02. **HRMS** m/z calculated for $C_{17}H_{13}ClF_4N_3O$ $[M+H]^+$ 386.0678, found 386.0683.

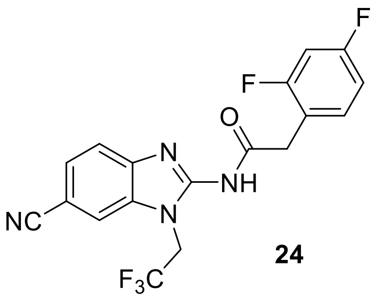

24

23% overall yield. **^{1}H NMR (500 MHz, DMSO, 90ºC)** δ 8.19-7.99 (m, 1H), 7,71-7,62 (m, 2H), 7.44 (dd, *J* = 7.5 Hz, 15 Hz, 1H), 7.09-7.00 (m, 2H), 5.24-4.97 (m, 2H), 3.94-3.75 (m, 2H). **^{13}C NMR (125 MHz, DMSO)** δ 179.77, 170.00, 152.83, 133.80, 133.15, 132.93, 132.73, 129.09, 127.80, 126.36, 119.82, 115.46, 113.94, 112.77, 110.85, 104.82, 104.48, 103.42, 103.21, 71.99, 35.16.

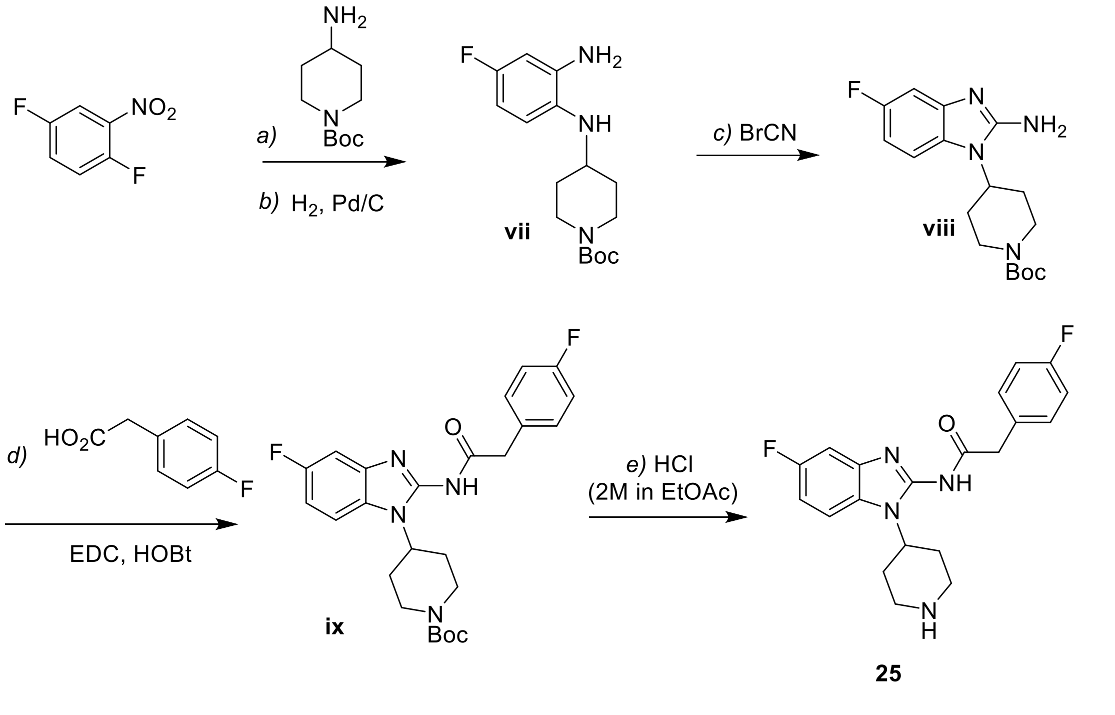

The compound ix was prepared by Method A, followed by B, C and D. To a solution of ix in DCM (0.1 mol.L^{-1}) was added HCl (2 mol.L^{-1} in EtOAc, 15 equiv.). The solution was stirred at room temperature. The solvents were removed under reduced pressure and

the residual solid was solubilized in a minimal amount of MeOH. To this solution was added NaOH (1.0 mol.L^{-1}) and the mixture was extracted with EtOAC. The organic layer was extracted with brine and dried with magnesium sulfate. The solution was filtered and evaporated under reduced pressure, generating **25** in 84% yield.

^{1}H NMR (500 MHz, MeOD) δ 7.93 (dd, *J* = 9.0, 4.0 Hz, 1H), 7.47 (dd, *J* = 8.2, 5.6 Hz, 2H), 7.41 (dd, *J* = 8.4, 2.2 Hz, 1H), 7.24 (td, *J* = 9.3, 2.3 Hz, 1H), 7.11 (t, *J* = 8.8 Hz, 2H), 5.13 (t, *J* = 12.3 Hz, 1H), 4.02 (s, 2H), 3.68 (d, *J* = 13.0 Hz, 2H), 3.29 (d, *J* = 10.9 Hz, 2H), 2.87 (qd, *J* = 13.3, 4.2 Hz, 2H), 2.26 (d, *J* = 13.2 Hz, 2H). **^{13}C NMR (126 MHz, MeOD)** δ 163.10, 161.16, 160.95, 159.03, 131.10, 131.03, 130.56, 125.15, 114.89, 114.72, 113.43, 113.36, 111.88, 111.68, 101.70, 101.40, 78.15, 77.84, 77.66, 67.88, 67.16, 63.12, 53.40, 51.34, 43.49, 25.73. **HRMS** m/z calculated for $C_{20}H_{21}F_2N_4O$ $[M+H]^+$ 371.1687, found 371.1681.

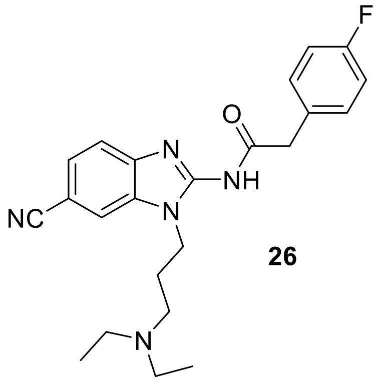

26

Method A, followed by B, C and D.

52% overall yield. **^{1}H NMR (500 MHz, $CDCl_3$)** δ 7.64 – 7.46 (m, 3H), 7.37 – 7.21 (m, 3H), 6.99 (t, *J* = 8.6 Hz, 3H), 4.12 (m, 4H), 2.65 (dd, *J* = 13.9, 6.8 Hz, 4H), 2.42 (s, 2H), 2.06 (s, 2H), 1.08 (t, *J* = 7.2 Hz, 6H). **^{13}C NMR (126 MHz, $CDCl_3$)** δ 188.74, 162.78, 160.83, 131.07, 131.01, 126.64, 119.66, 115.24, 115.07, 113.12, 105.41, 45.24, 40.31, 29.80, 25.34, 9.90. **HRMS** m/z calculated for $C_{23}H_{27}FN_5O$ $[M+H]^+$ 408.2194, found 408.2205.

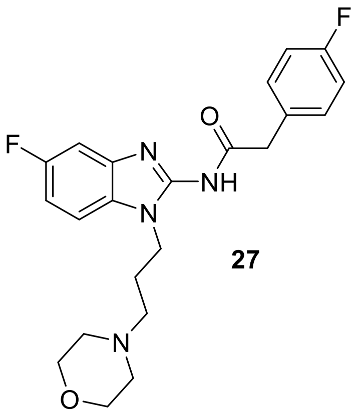

27

Method A, followed by B, C and D.

30% overall yield. **^{1}H NMR (500 MHz, CDCl$_3$)** δ 12.15 (s, 1H), 7.31 (s, 2H), 7.20 (m, 1H), 6.98 (m, 3H), 4.13 (s, 2H), 3.68 (m, 6H), 2.33 (m, 6H), 1.97 (s, 2H). **^{13}C NMR (126 MHz, CDCl$_3$)** δ 162.7, 160.7, 160.3, 158.4, 130.9, 115.0, 110.2, 66.7, 53.4, 40.1, 24.6. **HRMS** m/z calculated for $C_{22}H_{24}F_2N_4O_2$ $[M+H]^+$ 415.1940, found 415.1942.

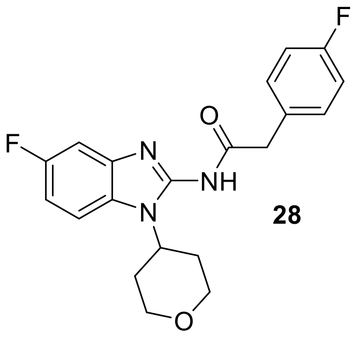

28

Method A, followed by B, C and D.

25% overall yield. **^{1}H NMR (500 MHz, CDCl$_3$)** δ 12.31 (s, 1H), 7.34 (m, 3H), 6.97 (m, 4H), 4.90 (t, J = 11.9 Hz, 1H), 4.16 (dd, J = 11.6, 4.6 Hz, 2H), 3.75 (s, 2H), 3.58 (t, J = 11.3 Hz, 2H), 2.53 (qd, J = 12.6, 4.6 Hz, 2H), 1.77 (dd, J = 12.5, 2.4 Hz, 2H). **^{13}C NMR (126 MHz, CDCl$_3$)** δ 162.6, 160.7, 160.1, 158.2, 132.9, 130.9, 124.6, 114.9, 111.5, 110.2, 67.4, 51.2, 46.5, 29.9. **HRMS** m/z calculated for $C_{20}H_{19}F_2N_3O_2$ $[M+H]^+$ 372.1518, found 372.1520.

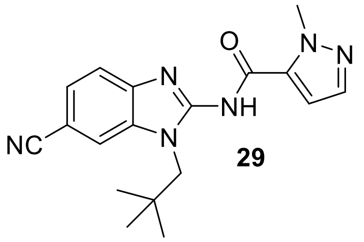

29

Method E, followed by B, C and D.

57% overall yield. **^{1}H NMR (500 MHz, DMSO d_6)** δ 13.06 (s, 1H), 8.14 (s, 1H), 7.69 – 7.61 (m, 2H), 7.44 (d, *J* = 1.8 Hz, 1H), 6.90 (d, *J* = 1.8 Hz, 1H), 4.21 (s, 3H), 4.06 (s, 2H), 1.02 (s, 9H). **^{13}C NMR (151 MHz, DMSO d_6)** δ 167.26, 153.88, 139.77, 136.95, 132.55, 130.82, 126.98, 119.43, 114.80, 112.67, 110.21, 104.49, 52.57, 34.53, 28.11. Obs: The pyrazine methyl's sign is under the DMSO sign (HSQC analysis). **HRMS** m/z calculated for $C_{18}H_{21}N_6O$ $[M+H]^+$ 337.1771, found 337.1775.

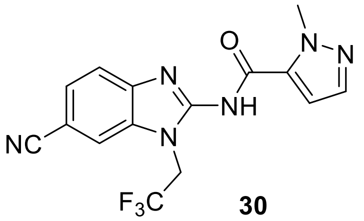

30

Method E, followed by B, C and D.

53% overall yield. **^{1}H NMR (500 MHz, DMSO)** δ 13.17 (s, 1H), 8.17 (s, 1H), 7.73 (dd, *J* = 8.3, 1.3 Hz, 1H), 7.65 (d, *J* = 8.3 Hz, 1H), 7.45 (d, *J* = 1.8 Hz, 1H), 6.98 (d, *J* = 1.8 Hz, 1H), 5.20 (q, *J* = 9.0 Hz, 2H), 4.21 (s, 3H). **^{13}C NMR (126 MHz, DMSO)** δ 167.85, 153.66, 139.88, 137.44, 133.29, 129.74, 128.39, 127.98, 125.74, 123.51, 121.27, 119.56, 114.57, 113.50, 111.27, 105.51, 43.60, 43.33, 43.05, 42.78.

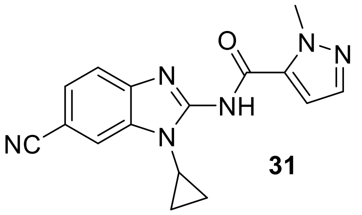

31

Method A, followed by B, C and D.

24% overall yield. **^{1}H NMR (400 MHz, DMSO)** δ 12.93 (s, 1H), 7.97 (s, 1H), 7.66 (m, 2H), 7.46 (s, 1H), 6.90 (s, 1H), 4.24 (s, 3H), 3.18 (m, 1H), 1.15 (m, 4H). **^{13}C NMR (101 MHz, DMSO)** δ 167.8, 137.5, 132.9, 131.1, 127.6, 119.9, 114.5, 113.2, 110.7, 105.0, 24.6, 7.0. **HRMS** m/z calculated for $C_{16}H_{14}N_6O_1$ $[M+Na]^+$ 329.1121, found 329.1164.

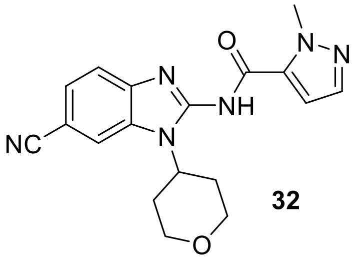

32

Method E, followed by B, C and D.

31% overall yield. **^{1}H NMR (500 MHz, DMSO d$_6$)** δ 13.08 (s, 1H), 8.25 (s, 1H), 7.70 – 7.63 (m, 2H), 7.47 (d, *J* = 1.3 Hz, 1H), 6.90 (d, J = 1.4 Hz, 1H), 4.94 (t, J = 12.0 Hz, 1H), 4.22 (s, 3H), 4.05 (dd, *J* = 11.2, 4.0 Hz, 2H), 3.54 (t, *J* = 11.3 Hz, 2H), 2.79 – 2.66 (m, 2H), 1.75 (d, *J* = 10.1 Hz, 2H). **^{13}C NMR (126 MHz, DMSO d$_6$)** δ 167.08, 152.46, 139.89, 136.99, 132.61, 128.72, 126.95, 119.35, 114.53, 112.85, 110.16, 104.69, 66.64, 51.82, 39.42, 29.29. **HRMS** m/z calculated for $C_{18}H_{19}N_6O_2$ $[M+H]^+$ 351.1564, found 351.1568.

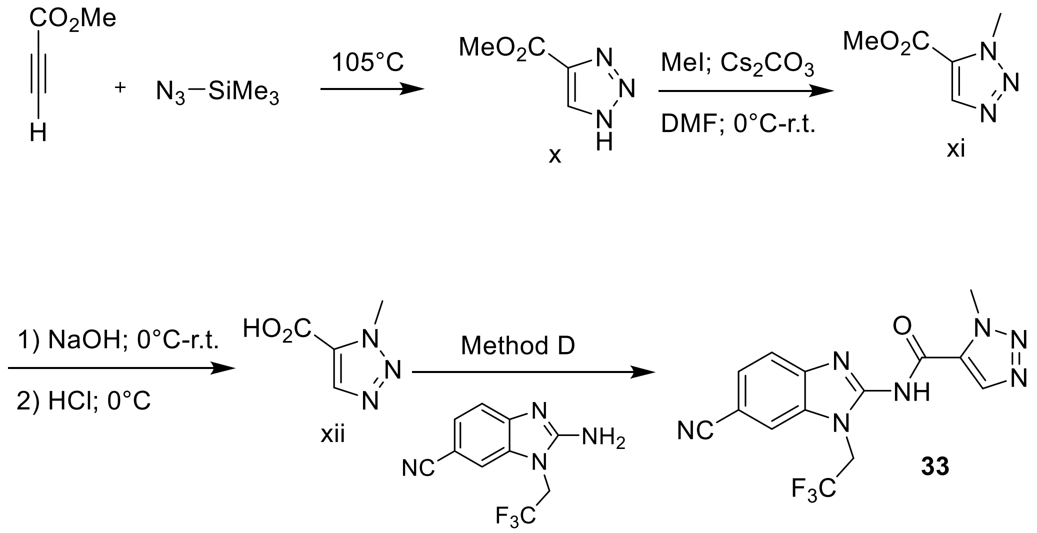

1-methyl-1*H*-1,2,3-triazole-5-carboxylic acid (xii) was prepared as reported in the references.[5,6] The aminobenzimidazole was prepared as reported in the synthesis of **30**. The coupling between xii and aminobenzimidazole was made using the Method D in 88% yield.

^{1}H NMR (500 MHz, DMSO) δ 13.27 (s, 1H), 8.31 (s, 1H), 8.21 (s, 1H), 7.76 (dd, *J* = 8.3, 1.2 Hz, 1H), 7.67 (d, *J* = 8.3 Hz, 1H), 5.26 (q, *J* = 8.9 Hz, 2H), 4.36 (s, 3H). **^{13}C NMR (126 MHz, DMSO)** δ 166.15, 153.56, 137.76, 135.38, 133.19, 129.73, 128.54, 127.97, 125.73, 123.49, 121.26, 119.50, 114.94, 113.78, 105.76, 43.65, 43.37, 43.09, 42.83, 37.70. **HRMS** m/z calculated for $C_{14}H_{11}F_3N_7O$ $[M+H]^+$ 350.0972, found 350.0973.

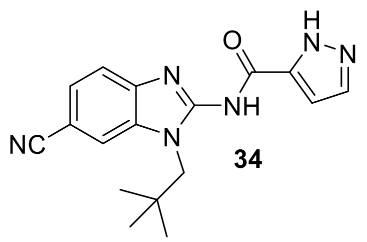

Method E, followed by B, C and D.

57% overall yield. **^{1}H NMR (600 MHz, DMSO d_6, 120 °C)** δ 7.98 (s, 1H), 7.68 (d, *J* = 8.2 Hz, 1H),7.65 – 7.49 (m, 1H), 7.57 (d, *J* = 8.2 Hz, 1H), 6.79 (s, 1H), 4.15 (s, 2H), 1.05 (s, 9H).

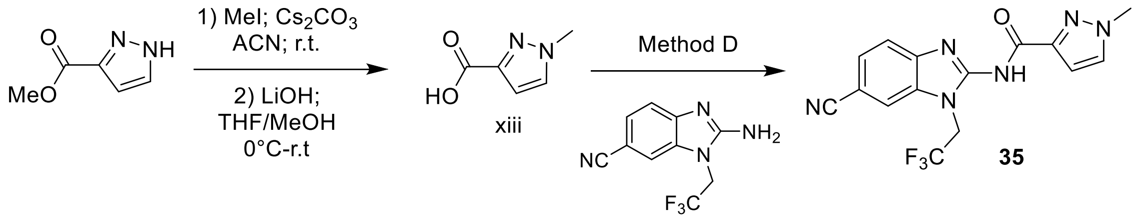

1-methyl-1*H*-pyrazole-3-carboxylic acid (xiii) was prepared as reported in the references.[7,8] The aminobenzimidazole was prepared as reported in the synthesis of **30**. The coupling between xiii and aminobenzimidazole was made using the Method D in 30% yield.

^{1}H NMR (400 MHz, DMSO) δ 8.11 (s, 1H), 7.69 (dd, *J* = 22.2, 8.7 Hz, 3H), 6.84 (s, 1H), 5.23 (s, 2H), 3.96 (s, 3H). **^{13}C NMR (151 MHz, DMSO)** δ 170.55, 153.73, 150.32, 133.80, 133.34, 132.00, 129.78, 128.99, 128.21, 119.73, 116.16, 114.27, 113.27, 109.06, 105.15, 70.02, 55.29, 43.10, 42.89. **HRMS** m/z calculated for $C_{15}H_{13}F_3N_6O$ $[M+H]^+$ 350.1092, found 350.1054.

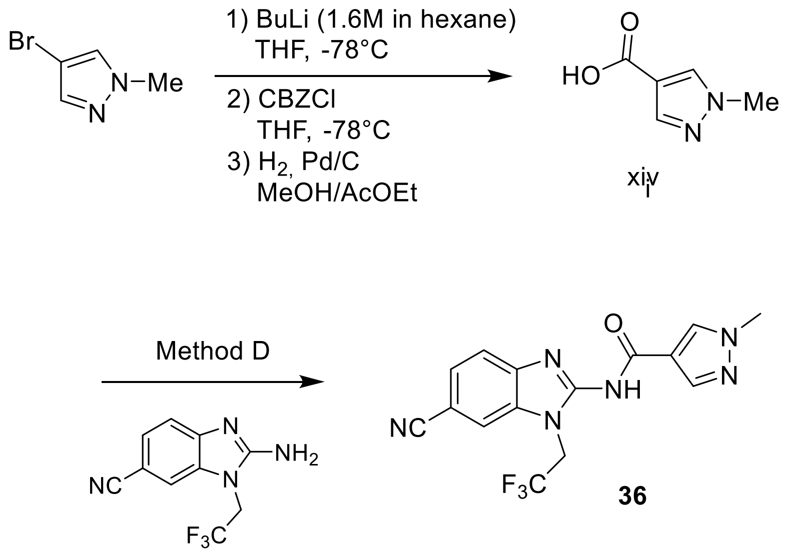

To a solution of the bromopyrazole derivative (321 µL, 3.1 mmol) in THF (8 mL) was added, dropwise, a solution of butyl lithium in hexane (1.6 M, 4.24 mL) at -78ºC. The mixture was stirred for 1 hour, after which CBZCl was slowly added (665 µL, 4.6 mmol). The temperature was, then allowed to reach room temperature and stirring was maintained for 16h. Aqueous sodium bicarbonate solution (10 mL) was added slowly to quench the reaction. The mixture was extracted with EtOAc (3 x 10 mL), the organic layer was dried over magnesium sulfate, filtered and evaporated under reduced pressure. The residue was purified by column chromatography eluting (1 Hex / 1 EtOAc). The pure product (205 mg, 0.95 mmol) was dissolved in a mixture of MeOH/EtOAc (2 mL, 1:1) and Pd/C (10 wt %, 1 mg, 0.01 mmol) was added. The mixture stirred under hydrogen atmosphere for 3 hours. The mixture was filtered on Celite and dried under vacuum to generate carboxylic acid xiv in 86 % yield. The aminobenzimidazole was prepared as reported in the synthesis of **30**. The coupling between xiv and aminobenzimidazole was performed using Method D in 55% yield.

50% overall yield. **^{1}H NMR (500 MHz, DMSO)** δ 12.82 (s, 1H), 8.27 (s, 1H), 8.10 (s, 1H), 7.91 (s, 1H), 7.69 (dd, *J* = 8.2, 1.1 Hz, 1H), 7.62 (d, *J* = 8.2 Hz, 1H), 5.19 (q, *J* =

8.9 Hz, 2H), 3.89 (s, 3H). **^{13}C NMR (126 MHz, DMSO)** δ 140.7, 134.1, 128.0, 125.8, 123.6, 119.7, 114.3, 105.0, 39.2. **HRMS** m/z calculated for $C_{15}H_{11}F_3N_6O$ [M+Na]+ 371.0839, found 371.0885.

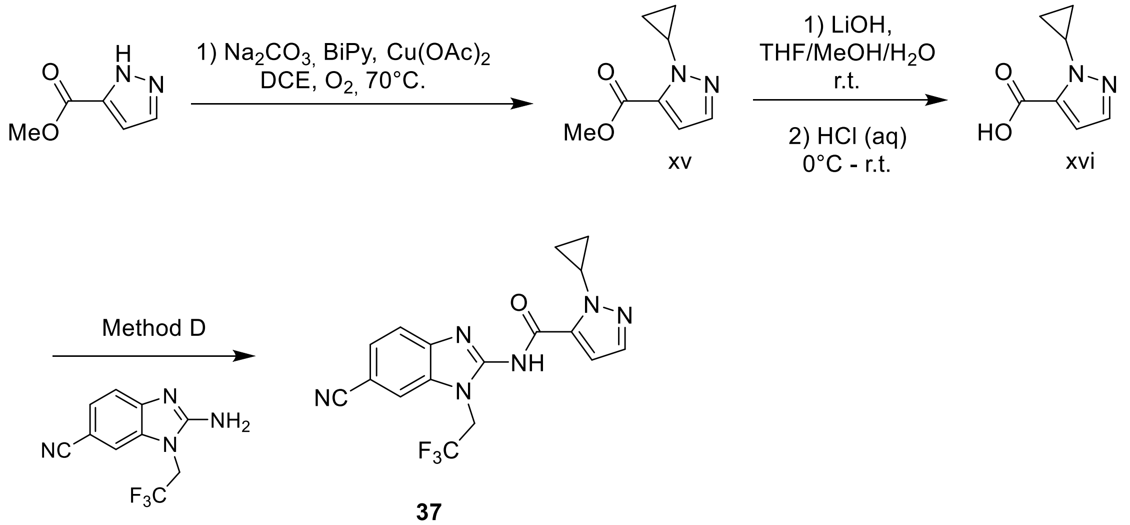

Methyl ester xv was prepared according to a literature procedure in a mixture of two isomers separable by column chromatography (1 Hex / 1 EtOAc).[9] To a solution of ester xv (90 mg, 0.54 mmol) in THF/MeOH (2 mL; 1:1) was added LiOH (1.08 mmol in 1 mL of water) at room temperature. After 2 h, an aqueous HCl solution (1 mol.L^{-1}) was added at 0°C until pH 2-3. The mixture was extracted with EtOAC (3 x 10 mL), the organic layer was dried with magnesium sulfate, filtered and evaporated under reduced pressure, to give the carboxylic acid xvi in 61% yield. The aminobenzimidazole was prepared as reported in the synthesis of **30**. The coupling between xvi and aminobenzimidazole was performed using Method D in 98% yield.

^{1}H NMR (400 MHz, DMSO) δ 13.18 (s, 1H), 8.18 (s, 1H), 7.73 (dd, *J* = 8.3, 1.4 Hz, 1H), 7.65 (d, *J* = 8.3 Hz, 1H), 7.41 (d, *J* = 1.8 Hz, 1H), 6.99 (d, *J* = 1.8 Hz, 1H), 5.20 (q, *J* = 9.0 Hz, 2H), 4.71 (dt, *J* = 7.6, 4.0 Hz, 1H), 1.16 (m, 2H), 0.99 (m, 2H). **^{13}C NMR**

(101 MHz, DMSO) δ 167.8, 153.6, 141.0, 137.0, 133.4, 129.8, 128.4, 126.0, 123.2, 119.6, 114.6, 113.6, 111.8, 110.1, 105.5, 33.4, 7.4. **HRMS** m/z calculated for $C_{17}H_{14}F_3N_6O$ $[M+Na]^+$ 397.0995, found 397.0974.

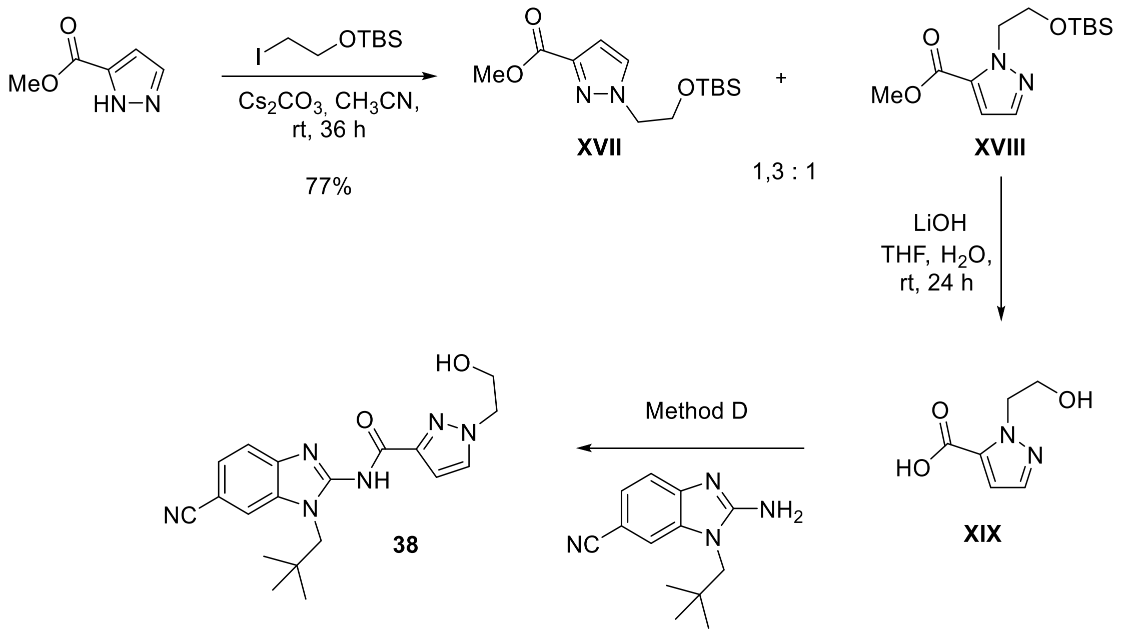

To a solution of ester (63 mg; 0.5 mmol) in CH_3CN (5.0 mL), Cs_2CO_3 was added. The heterogeneous mixture was allowed to stir 20 min before the alkyl iodide was added. Then, the reaction was stirred 36 h at rt, quenched with water (2 mL) and diluted with DCM (2 mL). The product was extracted with DCM, washed with brine and dried over $MgSO_4$ and concentrated. The residue was purified by flash column chromatography (Hexanes:AcOEt 20% to 40%) to yield 110 mg (77%) as a sum of separable regioisomers XVII and XVIII.

To a solution of ester XVIII (48 mg; 0.17 mmol) in THF (0.3 mL), was added an aqueous solution of LiOH (0.13 mL; 3.0 M). After 24h at rt, the reaction mixture was acidified with HCl (1.0 M, pH = 3). The volatiles were removed to yield a pale oil used in the next step without further purification. Followed by method D.

7% overall yield. **^{1}H NMR (400 MHz, CD_3OD)** δ 8.17 (s, 1H), 7.80 (bs, 1H), 7.79 (d, *J* = 8.5 Hz, 1H), 7.73 (dd, *J* = 8.3, 1.1 Hz, 1H), 6.97 (d, *J* = 1.6 Hz, 1H), 4.38 (t, *J* = 5.1 Hz, 2H), 4.35 (s, 2H), 3.98 (t, *J* = 5.1 Hz, 2H), 1.10 (s, 9H). **HRMS** m/z calculated for $C_{19}H_{23}N_6O_2^+$ $[M+H]^+$ 367.1877, found 367.1883.

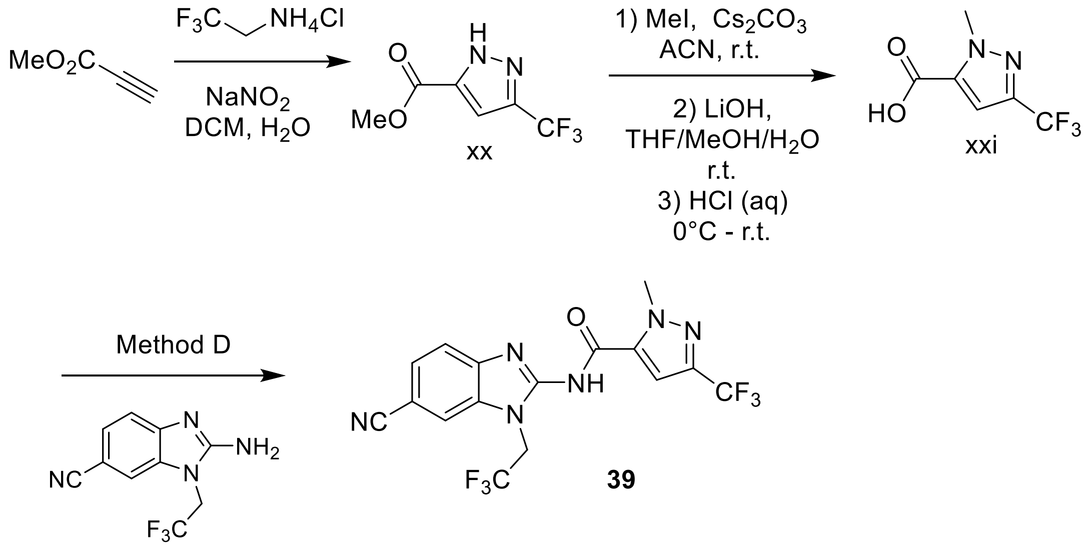

The methyl ester xvii was prepared steps using a literature procedure.[10] To a solution of methyl ester xx (284 mg; 1.58 mmol) in acetonitrile (10 mL) was added cesium carbonate (1.29 g; 3.95 mmol) and methyl iodide (0.12 mL; 1.89 mmol) at room temperature. After 3 h, acetone (20 mL) was added and the solid was filtered. The residual solution was evaporated in reduced pressure and purified by flash cromatographic column (9 Hex / 1 EtOAc), giving the methyl ester alkylated product in 27% yield. To a solution of this alkylated compound (90 mg; 0.43 mmol) in THF/MeOH (2 mL; 1:1) was added a solution of LiOH (0.87 mmol in 1 mL of water) at room temperature. After 2 h, a solution of HCl (1 mol.L^{-1}) was added at 0°C until pH 2-3. The mixture was extracted with EtOAC (2 x 15 mL), the organic layer was dried with magnesium sulfate, filtered and evaporated under reduced pressure, generating the carboxilic acid xxi in 84% yield. The aminobenzimidazole iii was prepared as reported in the synthesis of **30**. The coupling

between xviii and aminobenzimidazole was made using the Method D in quantitative yield.

^{1}H NMR (400 MHz, DMSO) δ 13.28 (s, 1H), 8.21 (s, 1H), 7.77 (dd, *J* = 8.3, 1.2 Hz, 1H), 7.68 (d, *J* = 8.3 Hz, 1H), 7.46 (s, 1H), 5.30 (q, *J* = 9.0 Hz, 2H), 4.30 (s, 3H). **^{13}C NMR (126 MHz, DMSO)** δ 166.55, 153.62, 141.71, 139.13, 138.83, 133.17, 129.72, 128.53, 125.77, 123.53, 122.82, 120.74, 119.51, 114.91, 113.74, 109.31, 105.74, 43.31, 43.04, 40.83, 40.57, 40.48, 40.40, 40.31, 40.24, 40.14, 39.98, 39.81, 39.64, 39.48. **HRMS** m/z calculated for $C_{16}H_{11}F_6N_6O$ $[M+H]^+$ 417.0893, found 417.0891.

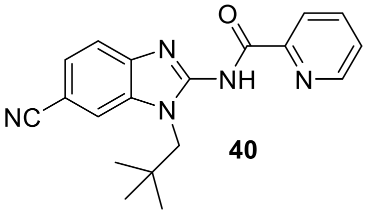

40

Method E, followed by B, C and D.

39% overall yield. **^{1}H NMR (400 MHz, CDCl$_3$)** δ 10.65 (s, 1H), 8.69 (s, 2H), 8.37 (d, *J* = 7.9 Hz, 1H), 7.94 (s, 1H), 7.77 (s, 1H), 7.63 (s, 1H), 7.53 (d, *J* = 7.8 Hz, 2H), 4.06 (s, 2H), 1.11 (s, 9H). **^{13}C NMR (101 MHz, CDCl$_3$)** δ 162.62, 161.62, 148.76, 145.07, 137.92, 134.50, 127.33, 126.32, 123.53, 120.24, 119.86, 114.72, 105.24, 55.19, 34.97, 28.52.

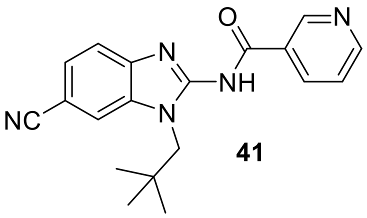

41

Method E, followed by B, C and D.

52% overall yield. **^{1}H NMR (250 MHz, CDCl$_3$)** δ 12.72 (s, 1H), 9.54 (s, 1H), 8.73 (d, *J* = 3.6 Hz, 1H), 8.51 (d, *J* = 8.0 Hz, 1H), 7.56 (d, *J* = 10.5 Hz, 2H), 7.40 (d, *J* = 8.2 Hz, 2H),

4.09 (s, 2H), 1.14 (s, 9H). **^{13}C NMR (126 MHz, $CDCl_3$)** δ 175.60, 156.05, 152.28, 151.40, 136.77, 132.91, 131.60, 131.31, 127.60, 123.24, 119.03, 114.48, 111.83, 106.68, 54.23, 35.09, 28.84. **HRMS** m/z calculated for $C_{19}H_{20}N_5O^+$ $[M+H]^+$ 334.1662, found 334.1659.

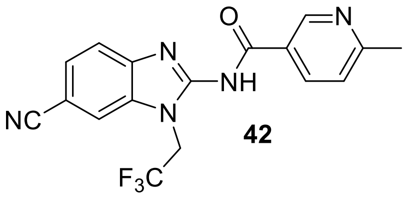

42

Method A, followed by B, C and D.

42% overall yield. **^{1}H NMR (500 MHz, DMSO)** δ 13.20 (s, 1H), 9.28 (s, 1H), 8.41 (dd, J = 8.0, 2.0 Hz, 1H), 8.19 (s, 1H), 7.74 (dd, J = 8.3, 1.2 Hz, 1H), 7.66 (d, J = 8.3 Hz, 1H), 7.38 (d, J = 8.0 Hz, 1H), 5.29 (q, J = 9.0 Hz, 2H), 2.55 (s, 3H). **^{13}C NMR (126 MHz, DMSO)** δ 161.63, 153.93, 150.49, 137.16, 130.36, 129.82, 128.35, 125.85, 123.15, 119.63, 114.59, 113.66, 107.00, 106.72, 105.42, 43.58, 43.32, 43.06, 42.75, 25.04. **HRMS** m/z calculated for $C_{17}H_{13}F_3N_5O$ $[M+H]^+$ 360.1067, found 360.1073.

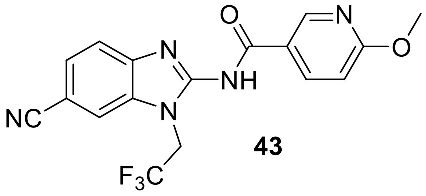

43

Method A, followed by B, C and D.

32% overall yield. **^{1}H NMR (500 MHz, DMSO)** δ 13.15 (s, 1H), 9.10 (d, J = 2.0 Hz, 1H), 8.42 (dd, J = 8.6, 2.3 Hz, 1H), 8.17 (s, 1H), 7.73 (dd, J = 8.2, 1.2 Hz, 1H), 7.65 (d, J = 8.2 Hz, 1H), 6.91 (d, J = 8.6 Hz, 1H), 5.27 (q, J = 9.0 Hz, 2H), 3.94 (s, 3H). **^{13}C NMR (126 MHz, DMSO)** δ 173.07, 166.05, 153.69, 149.92, 140.03, 133.35, 129.85, 128.33, 127.04, 126.02, 123.56, 119.64, 114.47, 113.39, 110.45, 105.34, 54.10, 43.61, 43.25,

42.98, 42.70. **HRMS** m/z calculated for $C_{17}H_{13}F_3N_5O_2$ $[M+H]^+$ 376.1016, found 376.1027.

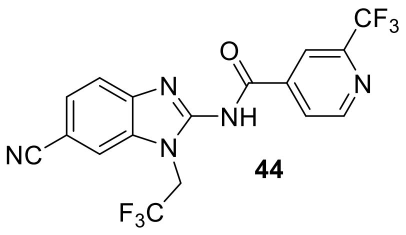

44

Method A, followed by B, C and D.

91% overall yield. **^{1}H NMR (500 MHz, DMSO)** δ 13.40 (s, 1H), 8.97 (d, *J* = 4.7 Hz, 1H), 8.43 (d, *J* = 5.2 Hz, 2H), 8.25 (s, 1H), 7.77 (d, *J* = 8.3 Hz, 1H), 7.71 (d, *J* = 8.3 Hz, 1H), 5.34 (q, *J* = 8.9 Hz, 2H). **^{13}C NMR (126 MHz, DMSO)** δ 171.15, 153.98, 151.71, 148.01, 147.74, 147.47, 147.20, 146.79, 133.09, 129.80, 128.59, 126.68, 125.75, 123.51, 123.20, 121.02, 119.46, 115.04, 113.94, 105.90, 43.76, 43.49, 43.22, 42.94. **HRMS** m/z calculated for $C_{17}H_{10}F_6N_5O$ $[M+H]^+$ 414.0784, found 414.0800.

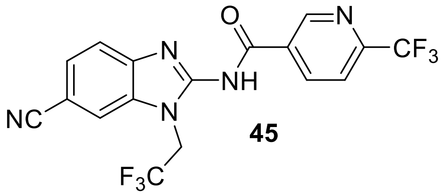

45

Method A, followed by B, C and D.

47% overall yield. **^{1}H NMR (500 MHz, DMSO)** δ 13.31 (s, 1H), 9.55 (s, 1H), 8.77 (d, *J* = 7.0 Hz, 1H), 8.22 (s, 1H), 8.03 (d, *J* = 7.3 Hz, 1H), 7.72 (dd, *J* = 30.5, 7.4 Hz, 2H), 5.34 (d, *J* = 8.0 Hz, 2H). **^{13}C NMR (126 MHz, DMSO)** δ 171.55, 153.80, 151.25, 149.10, 148.83, 148.56, 148.29, 139.03, 135.85, 133.17, 129.80, 128.52, 127.66, 125.79, 124.91, 123.68, 123.13, 121.04, 119.55, 114.92, 113.85, 110.12, 105.84, 43.71, 43.41, 43.13, 42.86. **HRMS** m/z calculated for $C_{17}H_{10}F_6N_5O$ $[M+H]^+$ 414.0784, found 414.0802.

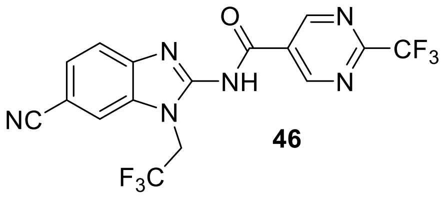

46

Method A, followed by B, C and D.

44% overall yield. **^{1}H NMR (500 MHz, DMSO)** δ 13.17 (s, 1H), 9.68 (s, 2H), 8.16 (s, 1H), 7.74 (s, 2H), 5.34 (q, *J* = 9.0 Hz, 2H). **^{13}C NMR (126 MHz, DMSO)** δ 149.42, 137.96, 127.33, 127.22, 125.55, 123.89, 123.32, 121.08, 119.61, 115.35, 105.66, 44.65, 44.39, 44.11, 43.83. **HRMS** m/z calculated for $C_{16}H_9F_6N_6O$ $[M+H]^+$ 415.0737, found 415.0354.

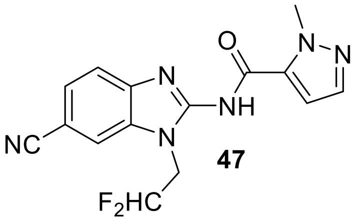

47

Method A, followed by B, C and D.

54% overall yield. **^{1}H NMR (500 MHz, DMSO)** δ 13.11 (s, 1H), 8.14 (s, 1H), 7.71 (dd, *J* = 8.3, 1.2 Hz, 1H), 7.65 (d, *J* = 8.3 Hz, 1H), 7.46 (d, *J* = 1.8 Hz, 1H), 6.95 (d, *J* = 1.7 Hz, 1H), 6.50 (tt, *J* = 54.8, 3.4 Hz, 1H), 4.76 (td, *J* = 15.1, 3.2 Hz, 2H), 3.18 (s, 3H). **HRMS** m/z calculated for $C_{15}H_{12}F_2N_6O$ $[M+Na]^+$ 353.0933, found 353.0935.

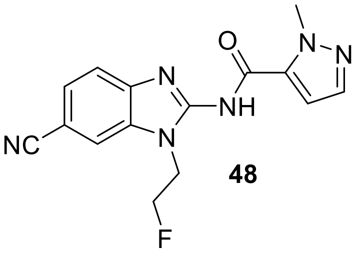

48

Method E, followed by B, C and D.

85% overall yield. **^{1}H NMR (500 MHz, DMSO)** δ 13.05 (s, 1H), 8.10 (s, 1H), 7.68 (d, *J* = 8.2 Hz, 1H), 7.64 (d, *J* = 8.2 Hz, 1H), 7.44 (d, *J* = 1.2 Hz, 1H), 6.92 (s, 1H), 4.83 (dt, *J* = 47.2, 4.5 Hz, 2H), 4.59 (dt, *J* = 26.4, 4.3 Hz, 2H), 4.21 (s, 3H). **^{13}C NMR (126 MHz, DMSO)** δ 167.82, 153.27, 140.22, 137.37, 133.22, 130.49, 127.62, 119.79, 114.58, 113.25, 110.85, 105.18, 82.86, 81.49, 43.23, 43.07, 42.72. **HRMS** m/z calculated for $C_{15}H_{14}FN_6O$ $[M+H]^+$ 313.1208, found 313.1218.

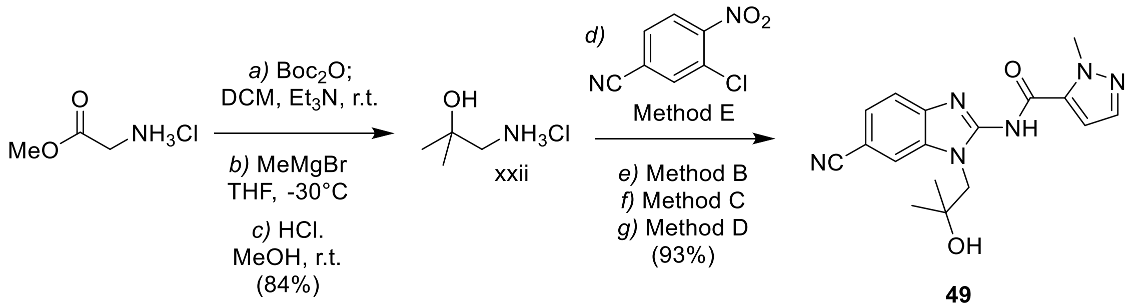

To a solution of methyl ester (2.1 g, 16.73 mmol) in DCM (40 mL) and Et_3N (7 mL, 50.2 mmol) was added Boc_2O (4.01 g, 18.4 mmol) at 0°C. After 4 h at room temperature, the mixture was extracted with saturated solution of ammonium chloride (3 x 30 mL). The organic layer was dried with magnesium sulfate, filtered and evaporated under reduced pressure. The residue was solubilized in anhydrous THF (20 mL) and a solution of methylmagnesium bromide (3 mol.L^{-1}, 13.82 mL, 41.48 mmol) was added at -78°C. The reaction stirred at room temperature for 14 h, and a saturated solution of ammonium chloride (10 mL) was added. The organic layer was dried with magnesium sulfate, filtered and evaporated under reduced pressure. The crude was solubilized in metanol (20 mL) and HCl (36.5%, 2 mL) was added at room temperature. The mixture was stirred at room temperature for 6h, and evaporated under reduced pressure, generating the ammonium chloride salt xxii in 84% yield overall steps.

49 was prepared in 93% yield from xix using the Method E, followed by B, C and D.

^{1}H NMR (500 MHz, DMSO) δ 13.06 (s, 1H), 8.09 (s, 1H), 7.75 – 7.55 (m, 1H), 7.44 (d, *J* = 1.7 Hz, 1H), 6.90 (d, *J* = 1.8 Hz, 1H), 4.93 (s, 1H), 4.22 (s, 3H), 4.17 (s, 2H), 1.22 (s, 6H). **^{13}C NMR (126 MHz, DMSO)** δ 167.67, 154.05, 140.19, 137.43, 133.00, 131.35, 127.18, 119.98, 116.38, 113.01, 110.69, 104.78, 79.65, 71.41, 53.35, 27.99. **HRMS** m/z calculated for $C_{17}H_{19}N_6O_2$ [M+H]$^+$ 339.1564, found 339.1566.

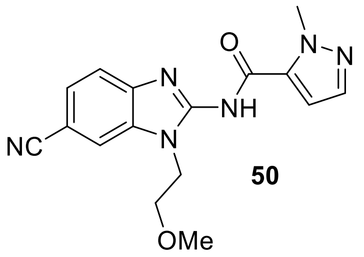

50

Method A, followed by B, C and D.

89% overall yield. **^{1}H NMR (500 MHz, DMSO)** δ 13.00 (s, 1H), 8.07 (s, 1H), 7.65 (m, 2H), 7.44 (d, *J* = 1.9 Hz, 1H), 6.90 (d, *J* = 1.4 Hz, 1H), 4.41 (t, *J* = 5.3 Hz, 2H), 4.21 (s, 3H), 3.74 (t, *J* = 5.3 Hz, 2H), 3.24 (s, 3H). **^{13}C NMR (126 MHz, DMSO)** δ 167.7, 153.2, 140.3, 137.4, 133.0, 130.5, 127.4, 119.9, 114.8, 113.2, 110.7, 105.1, 69.6, 58.6, 42.3, 40.6, 29.5. **HRMS** m/z calculated for $C_{16}H_{16}N_6O_2$ [M+Na]$^+$ 347.1227, found 347.1193.

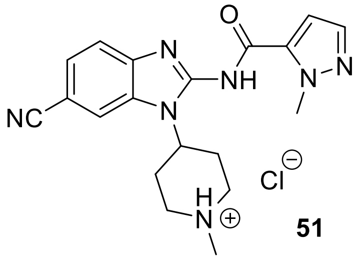

51

Method E, followed by B, C and D.

6% overall yield. **^{1}H NMR (500 MHz, DMSO d$_6$)** δ 13.09 (s, 1H), 10.46 (s, 1H), 8.31 (s, 1H), 7.70 (d, *J* = 8.1 Hz, 1H), 7.67 (d, *J* = 8.3 Hz, 1H), 7.47 (s, 1H), 7.14 (s, 1H), 5.08 (s, 1H), 4.21 (s, 3H), 3.61 (d, *J* = 11.7 Hz, 2H), 3.39 – 3.16 (m, 2H), 2.97 – 2.77 (m, 5H),

2.04 (d, *J* = 12.7 Hz, 2H). **^{13}C NMR (126 MHz, DMSO d_6)** δ 167.43, 152.51, 139.58, 136.87, 132.95, 128.11, 127.13, 119.30, 114.44, 113.07, 110.83, 104.58, 54.33, 52.92, 48.83, 42.49, 25.55. **HRMS** m/z calculated for $C_{19}H_{22}N_7O^+$ $[M+H]^+$ 364.1880, found 364.1876.

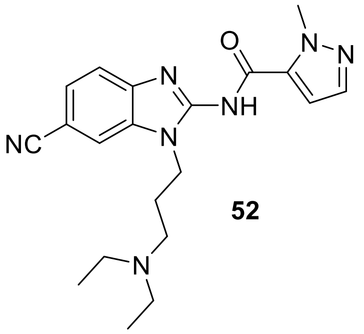

52

Method A, followed by B, C and D.

43% overall yield. **^{1}H NMR (500 MHz, DMSO)** δ 12.61 (s, 1H), 8.13 (s, 1H), 7.66 (dd, *J* = 8.2, 1.2 Hz, 1H), 7.62 (d, *J* = 8.2 Hz, 1H), 7.44 (d, *J* = 1.9 Hz, 1H), 6.90 (d, *J* = 1.9 Hz, 1H), 4.38 – 4.08 (m, 5H), 2.79 – 2.41 (m, 8H), 1.93 (dt, *J* = 12.7, 6.3 Hz, 2H), 0.94 (t, *J* = 7.1 Hz, 6H). **^{13}C NMR (126 MHz, DMSO)** δ 167.52, 153.09, 140.23, 137.33, 133.37, 130.17, 127.37, 119.88, 114.34, 113.36, 110.57, 104.99, 49.69, 46.38, 40.93, 40.48, 25.16, 11.33. **HRMS** m/z calculated for $C_{20}H_{26}N_7O$ $[M+H]^+$ 380.2193, found 380.2198.

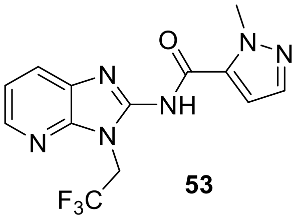

53

Method A, followed by B, C and D.

72% overall yield. **^{1}H NMR (500 MHz, $CDCl_3$)** δ 12.19 (s, 1H), 8.32 (dd, *J* = 5.1, 1.1 Hz, 1H), 7.59 (dd, *J* = 7.9, 1.1 Hz, 1H), 7.48 (d, *J* = 1.9 Hz, 1H), 7.27 (dd, *J* = 12.5, 4.6

Hz, 1H), 7.04 (d, *J* = 1.9 Hz, 1H), 5.30 – 4.92 (m, 2H), 4.31 (s, 3H). **^{13}C NMR (126 MHz, CDCl$_3$)** δ 170.09, 153.97, 143.84, 142.54, 139.48, 137.49, 124.38, 122.14, 121.98, 119.61, 118.21, 111.43, 42.29, 42.00, 41.71, 41.41, 39.93. **HRMS** m/z calculated for $C_{13}H_{12}F_3N_6O$ [M+H]$^+$ 325.1019, found 325.1030.

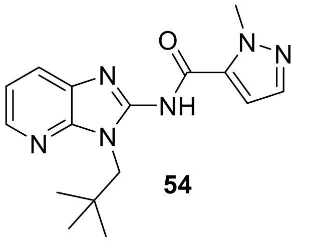

54

Method E, followed by B, C and D.

45% overall yield. **^{1}H NMR (500 MHz, CDCl$_3$)** δ 12.32 (s, 1H), 8.25 (d, *J* = 5.0 Hz, 1H), 7.48 (d, *J* = 7.8 Hz, 1H), 7.44 (d, *J* = 1.9 Hz, 1H), 7.14 (d, *J* = 2.8 Hz, 1H), 6.98 (d, *J* = 1.9 Hz, 1H), 4.29 (s, 3H), 4.14 (s, 2H), 1.08 (s, 9H). **^{13}C NMR (126 MHz, CDCl$_3$)** δ 170.08, 155.10, 144.35, 143.35, 140.15, 137.49, 121.80, 118.64, 117.56, 111.00, 52.19, 39.95, 34.52, 28.73.

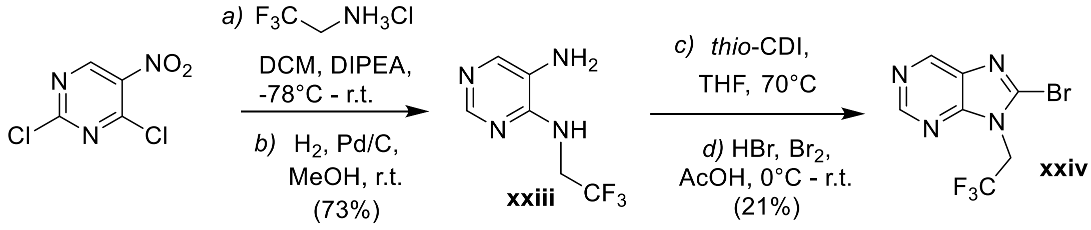

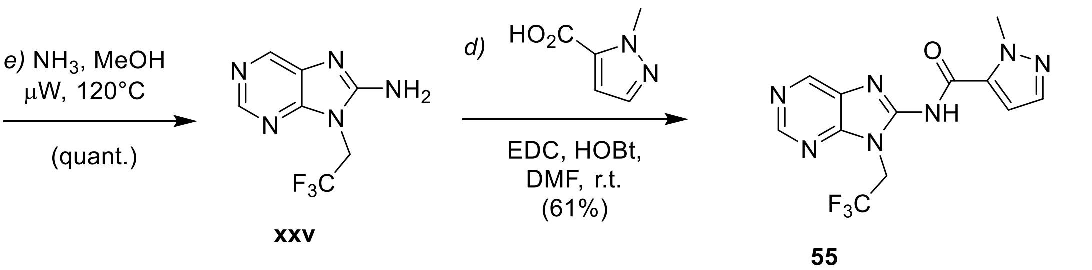

To a solution of 2,4-dichloro-5-nitropyrimidine (0.9 g; 4.64 mmol) in DCM (30 mL) and DIPEA (1.17 mL; 6.7 mmol) was addded trifluoroethylamonium chloride (0.75 g; 5.57

mmol) at -78°C. The reaction was spontaneously warmed to room temperature. The mixture was washed with saturated ammonium chloride solution (3 x 20 mL). The organic layer was dried with magnesium sulfate, filtered and evaporated under reduced pressure, giving the product of aromatic substitution reaction in 77% yield.[11] To a solution of this compound (0.49 g; 1.91 mmol) in methanol (20 mL) was added 10% Pd/C (50 mg) and hydrogen gas (1 bar) until the consumption of the nitroaromatic derivative. The solution was filtered through celite and evaporated under reduced pressure, generating the reduced product xxiii in 95% yield. To a suspension of xxiii (1.2 g; 6.25 mmol) in THF (50 mL) was added *thio*-CDI (1.22 g; 6.87 mmol) and the mixture was stirred at 70°C for 14 h. The mixture was evaporated under reduced pressure and purified by flash chromatographic column (9 DCM / 1 MeOH), giving the thiourea derivative in 75% yield. To a solution of this thiourea derivative (1.06 g; 4.53 mmol) in acetic acid (20 mL) was added HBr (48%, 1.03 mL; 6.11 mmol) and bromine (0.84 mL; 16.31 mmol) at 0°C. After 15 h, a solution of NaOH (2.5 mol.L^{-1}) was added at 0° C until pH 5. The mixture was extracted with EtOAc (2 x 50 mL). The organic layer was deried with magnesium sulfate, filtered and evaporated under reduced pressure. The crude was purified by flash cromatographic column (1 Hex/ 1 EtOAc), giving the bromine xxiv in 28% yield. The bromine xxiv (62 mg; 0.22 mmol) was solubilized in an ammonia solution (7 mol.L^{-1} in metanol; 3 mL) and heated for 2 h at 120°C in a microwave reactor. The reaction solution was evaporated under reduced pressure, generating the aminobenzimidazole analogue xxv in a quantitative yield.

55 was prepared in 61% yield, using the Method D from aminobenzimidazole xxii.

^{1}H NMR (500 MHz, $CDCl_3$) δ 12.09 (s, 1H), 8.95 (s, 1H), 8.64 (s, 1H), 7.50 (d, *J* = 2.0 Hz, 1H), 7.28 (s, 1H), 7.07 (d, *J* = 2.0 Hz, 1H), 4.91 (q, *J* = 8.2 Hz, 2H), 4.31 (s, 3H). **HRMS** m/z calculated for $C_{12}H_{11}F_3N_7O$ $[M+H]^+$ 326.0972, found 326.0973.

References

1- Scott B. Hoyt, Min K. Park, Clare London, Yusheng Xiong, Jim Tata, D. Jonathan Bennett, Andrew Cooke, Jiaqiang Cai, Emma Carswell, John Robinson, John MacLean, Lindsay Brown, Simone Belshaw, Thomas R. Clarkson, Kun Liu, Gui-Bai Liang, Mary Struthers, Doris Cully, Tom Wisniewski, Ning Ren, Charlene Bopp, Andrea Sok, Tian-Quan Cai, Sloan Stribling, Lee-Yuh Pai, Xiuying Ma, Joe Metzger, Andreas Verras, Daniel McMasters, Qing Chen, Elaine Tung, Wei Tang, Gino Salituro, Nicole Buist, Jeff Kuethe, Nelo Rivera, Joe Clemas, Gaochao Zhou, Jack Gibson, Carrie Ann Maxwell, Mike Lassman, Theresa McLaughlin, Jose Castro-Perez, Daphne Szeto, Gail Forrest, Richard Hajdu, Mark Rosenbach, and Amjad Ali. Discovery of Benzimidazole CYP11B2 Inhibitors with in Vivo Activity in Rhesus Monkeys. *ACS Medicinal Chemistry Letters* **2015** *6* (5), 573-578. DOI: 10.1021/acsmedchemlett.5b00054.

2- Mallesh Beesu, Subbalakshmi S. Malladi, Lauren M. Fox, Cassandra D. Jones, Anshuman Dixit, and Sunil A. David. Human Toll-Like Receptor 8-Selective Agonistic Activities in 1-Alkyl-1H-benzimidazol-2-amines. *Journal of Medicinal Chemistry* **2014** 57 (17), 7325-7341. DOI: 10.1021/jm500701q.

3- Jean-Pierre Falgueyret, Renata M. Oballa, Osamu Okamoto, Gregg Wesolowski, Yves Aubin, Robert M. Rydzewski, Peppi Prasit, Denis Riendeau, Sevgi B. Rodan, and M. David Percival. Novel, Nonpeptidic Cyanamides as Potent and

Reversible Inhibitors of Human Cathepsins K and L. *Journal of Medicinal Chemistry* **2001** *44* (1), 94-104. DOI: 10.1021/jm0003440.

4- Michael J. HaddMichael D. HockerMark W. HolladayGang LiuMartin W. RowbottomShimin Xu. Heterocyclic compounds and methods of use thereof. US20130096113A1.

5- Francine Sternfeld, Robert W. Carling, Richard A. Jelley, Tamara Ladduwahetty, Kevin J. Merchant, Kevin W. Moore, Austin J. Reeve, Leslie J. Street, Desmond O'Connor, Bindi Sohal, John R. Atack, Susan Cook, Guy Seabrook, Keith Wafford, F. David Tattersall, Neil Collinson, Gerard R. Dawson, José L. Castro and Angus M. MacLeod. Selective, Orally Active γ-Aminobutyric AcidA α5 Receptor Inverse Agonists as Cognition Enhancers. *Journal of Medicinal Chemistry* **2004** *47* (9), 2176-2179. DOI: 10.1021/jm031076j

6- Benoît Moreau, Jeff A. O'Meara, Josée Bordeleau, Michel Garneau, Cedrickx Godbout, Vida Gorys, Mélissa Leblanc, Elisia Villemure, Peter W. White, and Montse Llinàs-Brunet. Discovery of Hepatitis C Virus NS3-4A Protease Inhibitors with Improved Barrier to Resistance and Favorable Liver Distribution. *Journal of Medicinal Chemistry* **2014** *57* (5), 1770-1776. DOI: 10.1021/jm400121t

7- Sonja Nordhoff, Sebastian Wachten, Achim Kless, Felix Voss, Stefanie Ritter. Pyrazolyl-Based Carboxamides I. US20140194443A1.

8- Felix Voss, Stefanie Ritter, Soja Nordhoff, Sebastian Wachten, Stefan Oberborsch, Achim Kless. Annelated pyrroles and their use as crac inhibitors. WO2015022073A1.

9- Vladimir Ladziata, Peter W. Glunz, Zilun Hu, Yufeng Wang. Spirocycloheptanes as inhibitors of rock. WO2016010950.

10- Evgeniy Y. Slobodyanyuk, Olexiy S. Artamonov, Oleg V. Shishkin, Pavel K. Mykhailiuk. One-Pot Synthesis of CF_3-Substituted Pyrazolines/Pyrazoles from Electron-Deficient Alkenes/Alkynes and CF_3CHN_2 Generated in situ: Optimized Synthesis of Tris(trifluoromethyl)pyrazole. *European Journal of Organic Chemistry* **2014**, *12*, 2487-2495. DOI: 10.1002/ejoc.201301852.

11- Jiao Yang, Li-Jiao Wang, Jing-Jing Liu, Lei Zhong, Ren-Lin Zheng, Yong Xu, Pan Ji, Chun-Hui Zhang, Wen-Jing Wang, Xing-Dong Lin, Lin-Li Li, Yu-Quan Wei, Sheng-Yong Yang. Structural Optimization and Structure–Activity Relationships of N^2-(4-(4-Methylpiperazin-1-yl)phenyl)-N^8-phenyl-9H-purine-2,8-diamine Derivatives, a New Class of Reversible Kinase Inhibitors Targeting both EGFR-Activating and Resistance Mutations. Journal of Medicinal Chemistry **2012**, *55* (23), 10685–10699. DOI: 10.1021/jm301365e.

NMR spectra

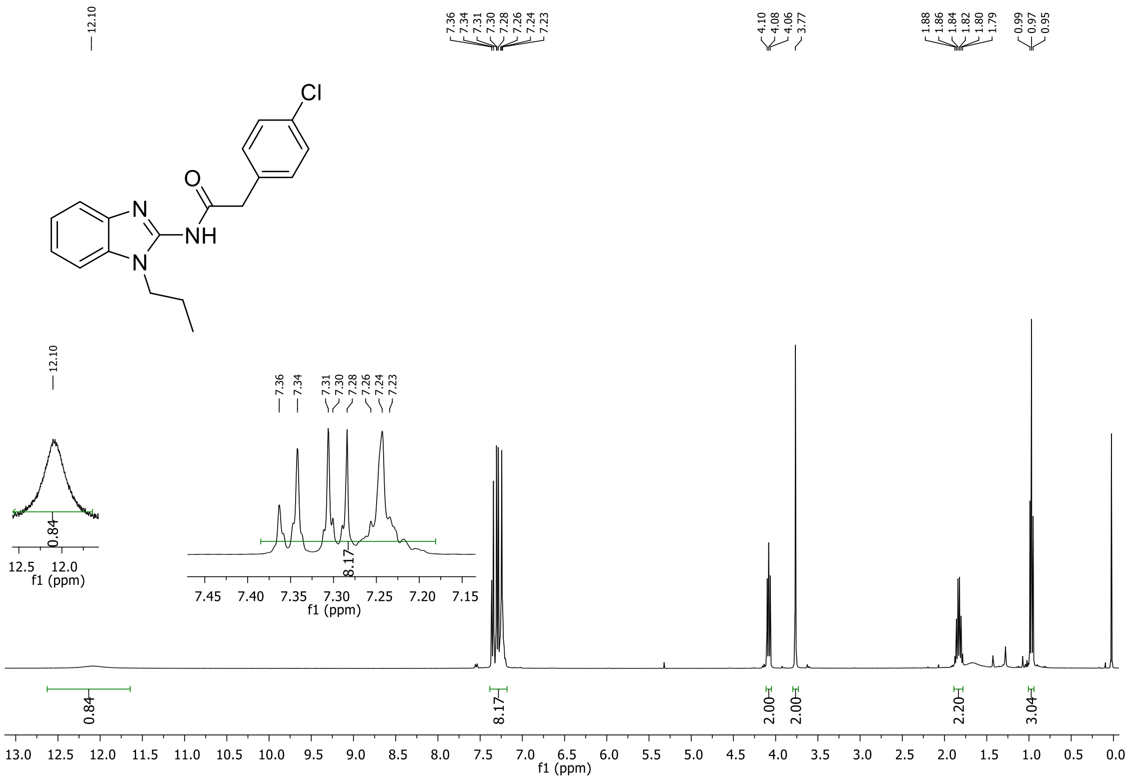

^{1}H NMR of Hit 1 (400 MHz, $CDCl_3$)

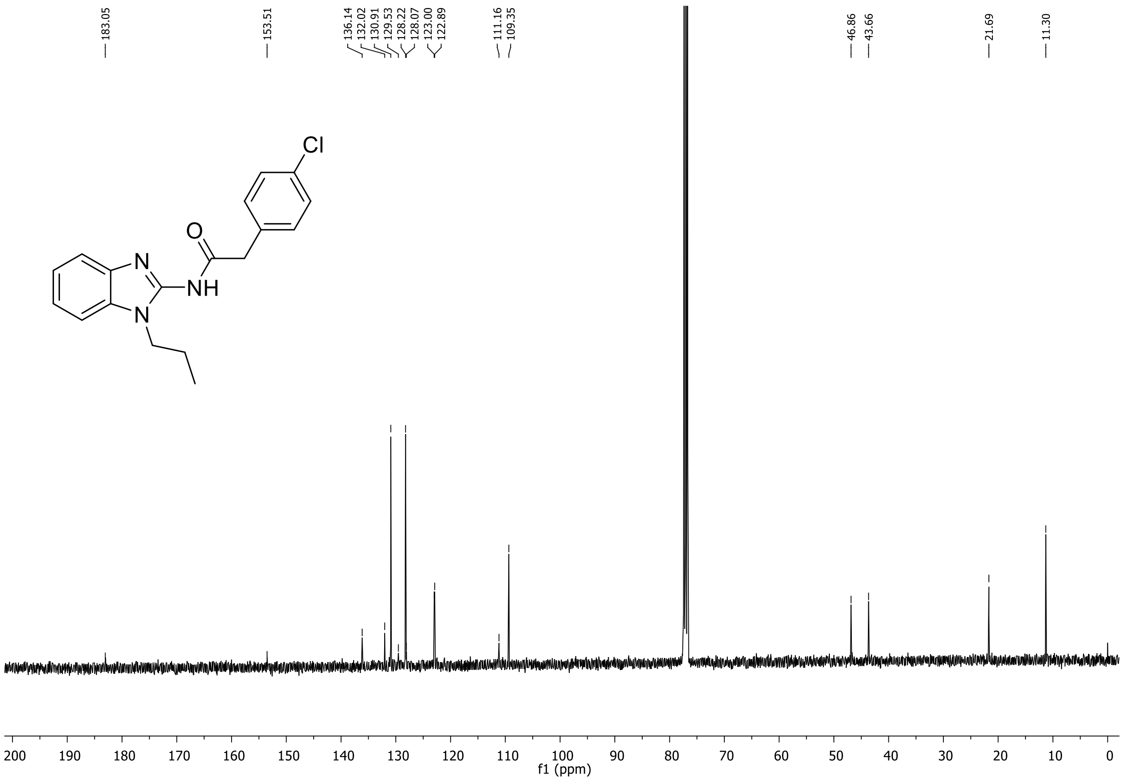

^{13}C NMR of Hit 1 (101 MHz, $CDCl_3$)

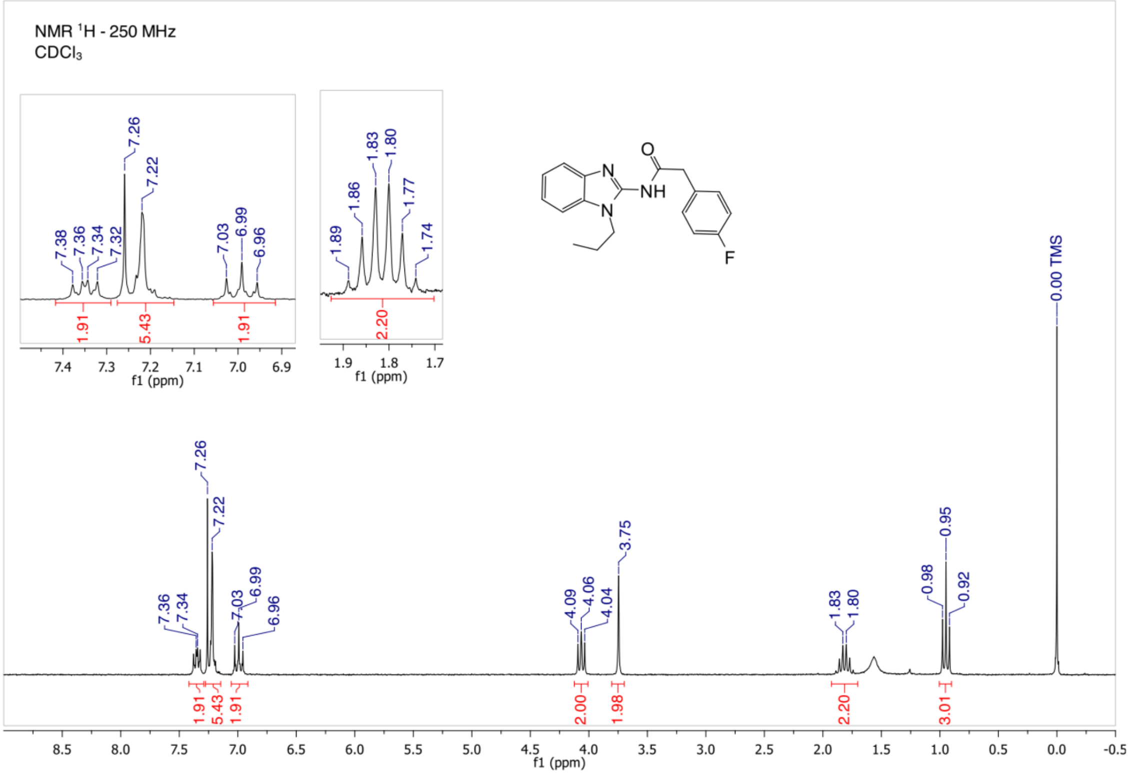

^{1}H NMR of 2 (250 MHz, $CDCl_3$)

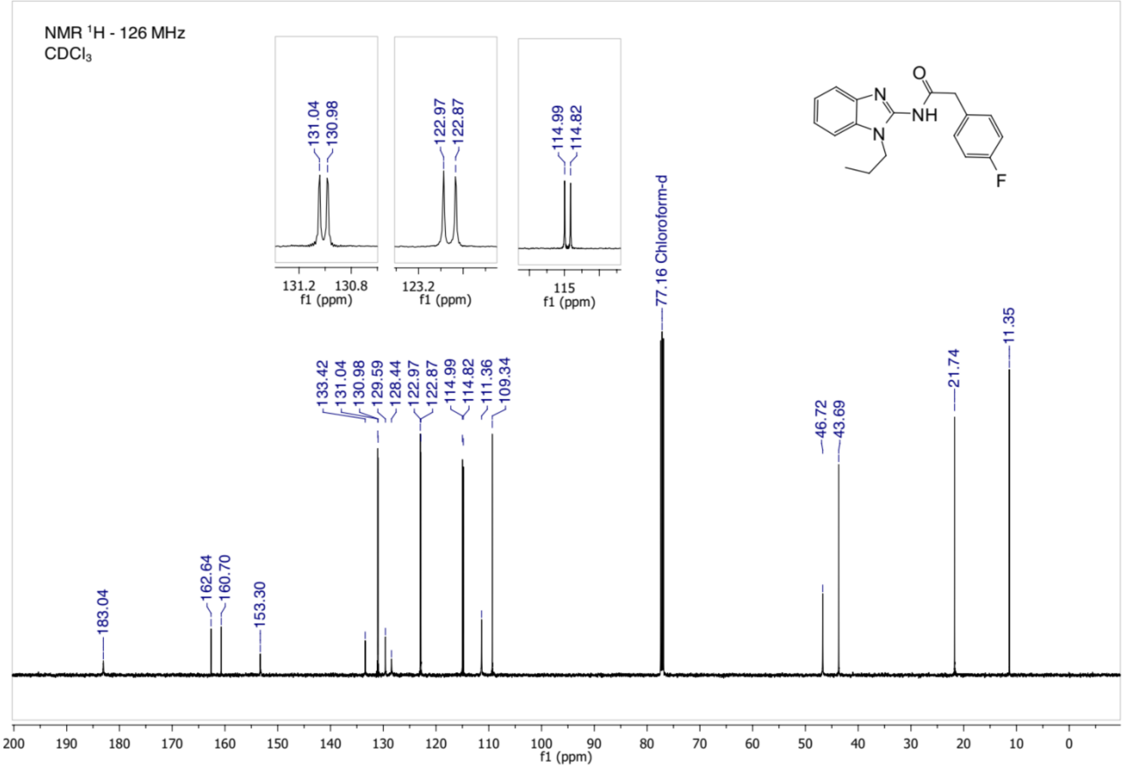

^{13}C NMR of 2 (125 MHz, $CDCl_3$)

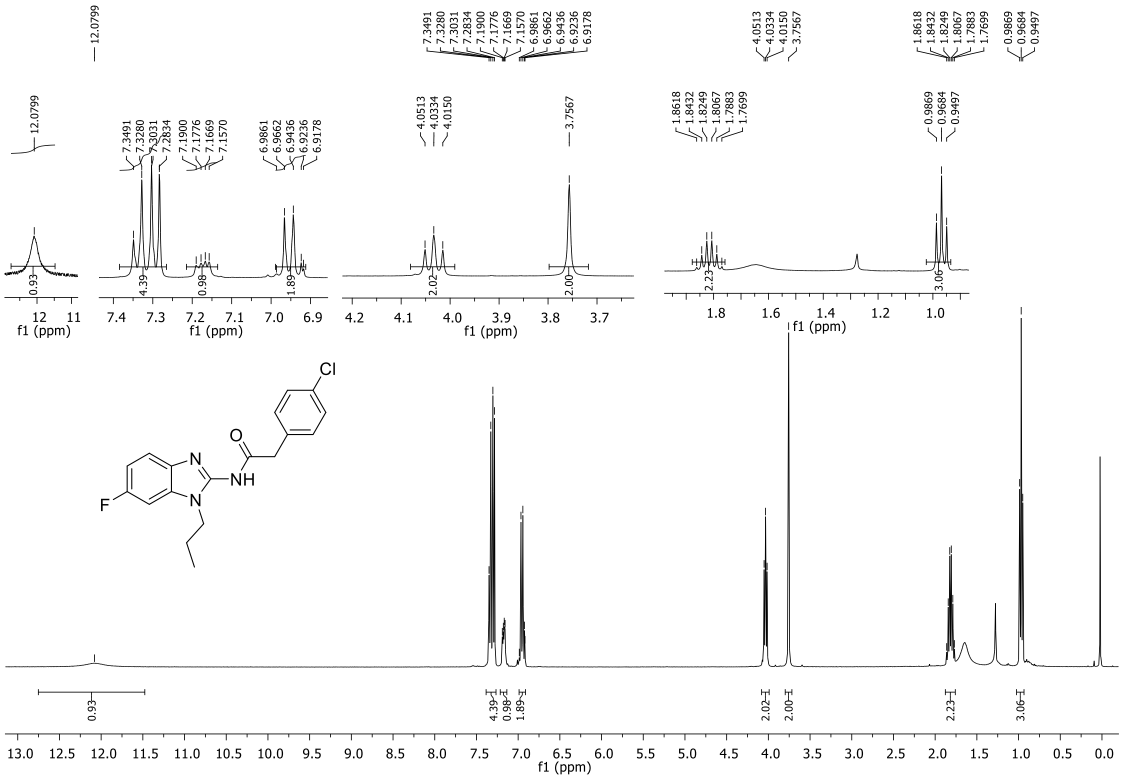

^{1}H NMR of 3 (400 MHz, $CDCl_3$)

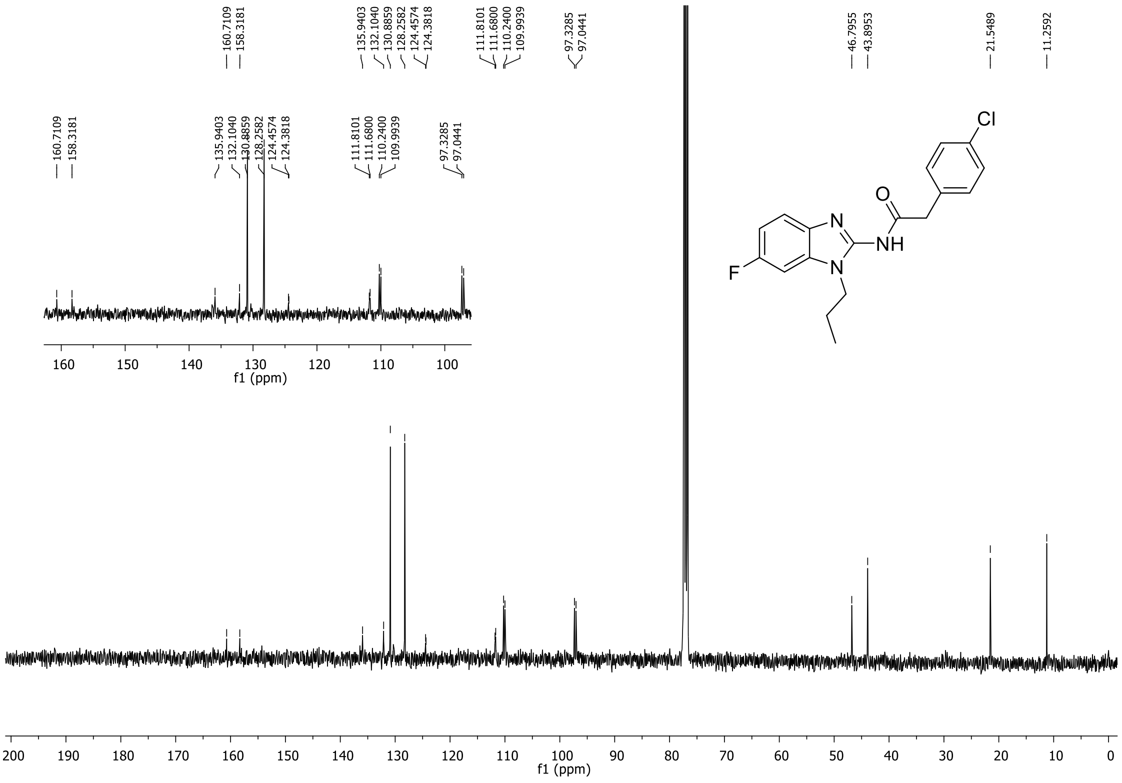

^{13}C NMR of 3 (101 MHz, $CDCl_3$)

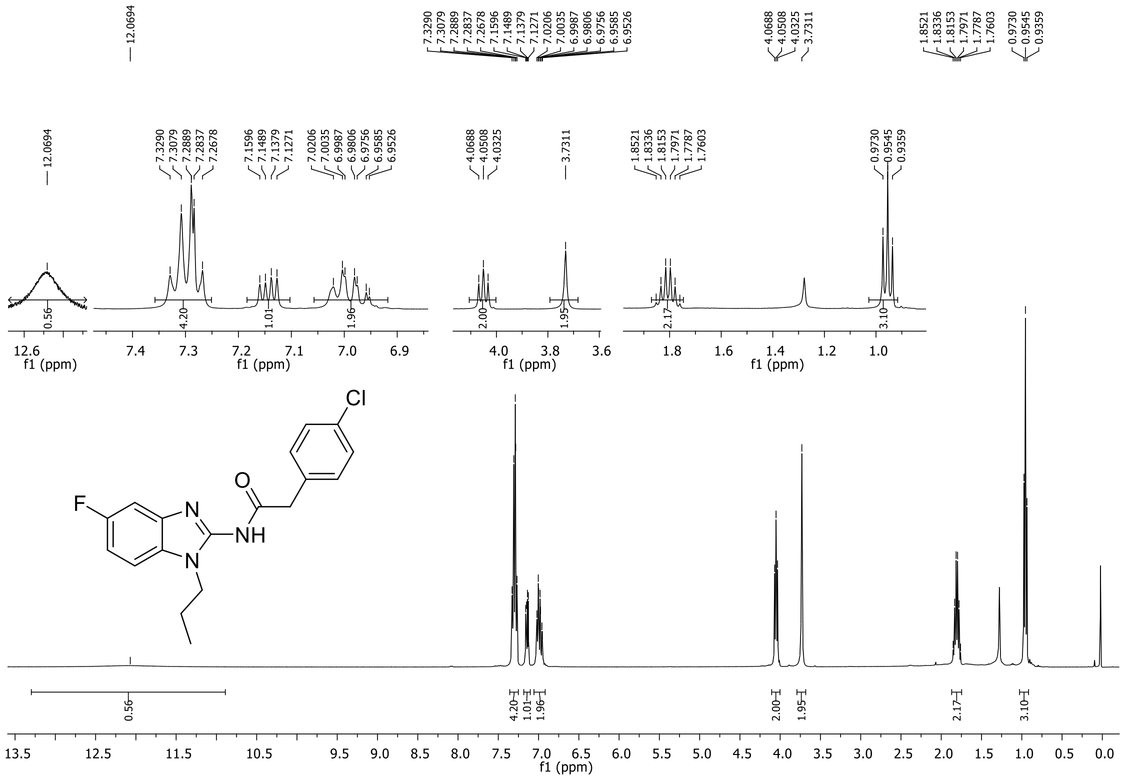

^{1}H NMR of 4 (400 MHz, $CDCl_3$)

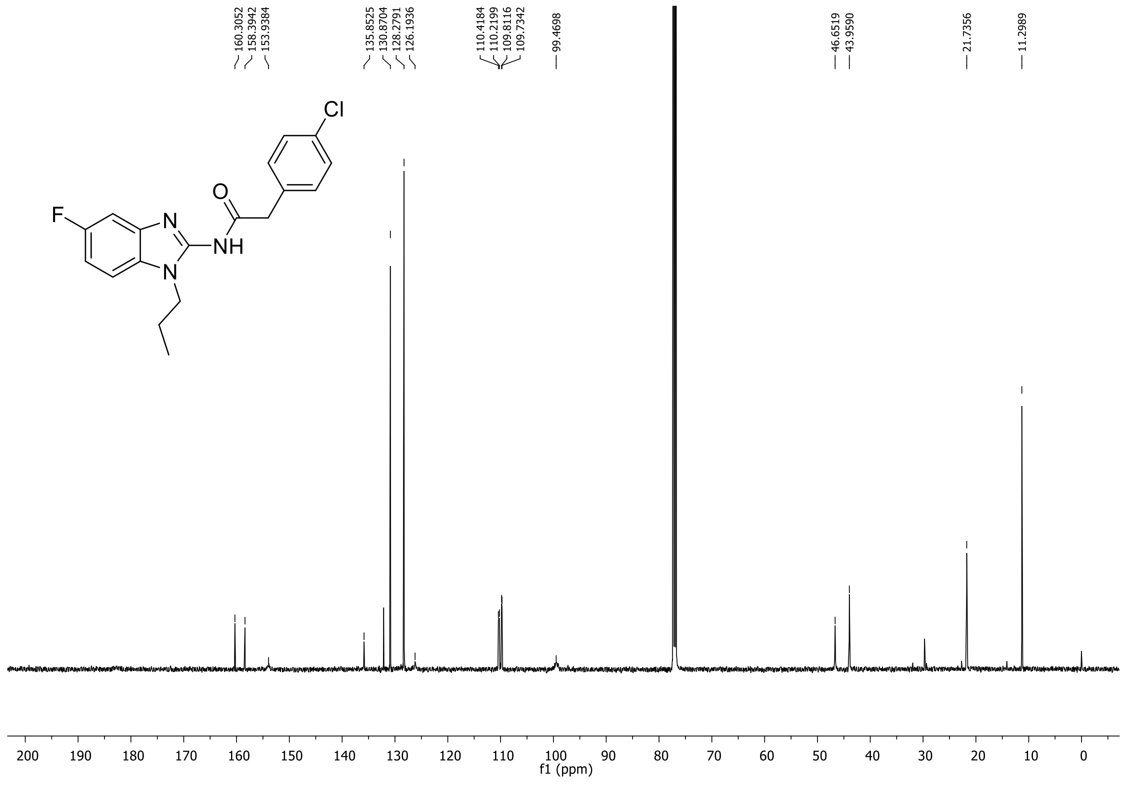

^{13}C NMR of 4 (126 MHz, $CDCl_3$)

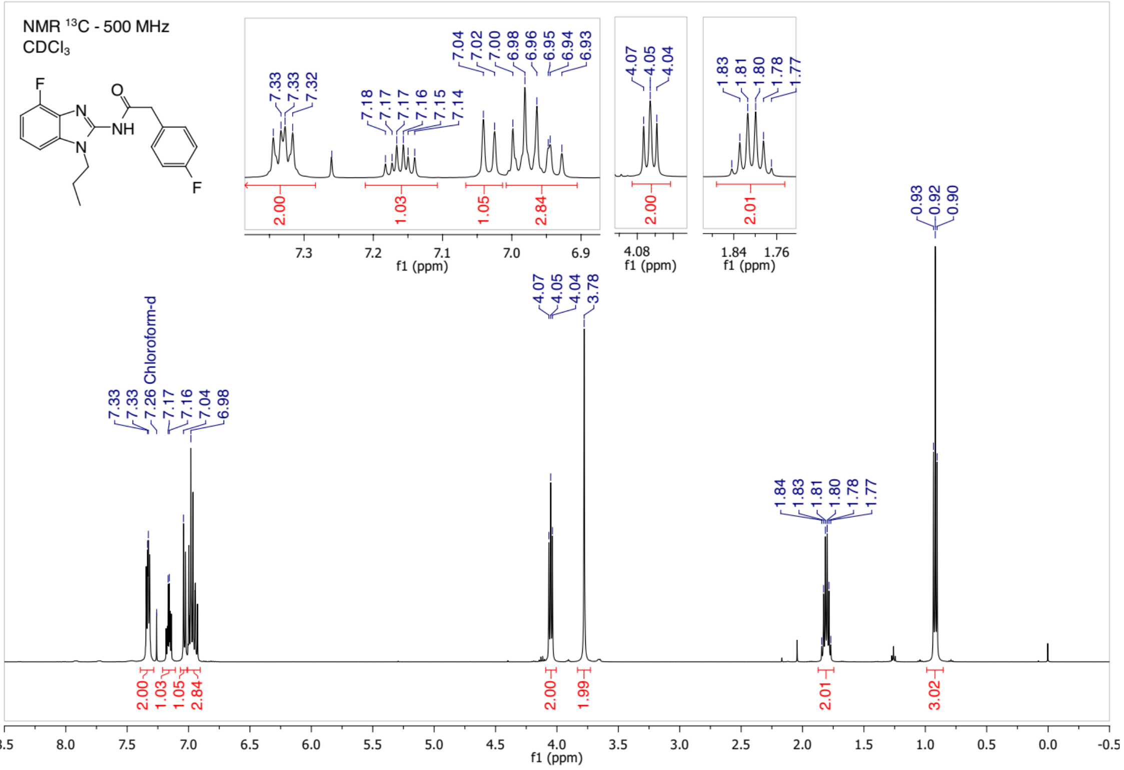

^{1}H NMR of 5 (500 MHz, CDCl$_3$)

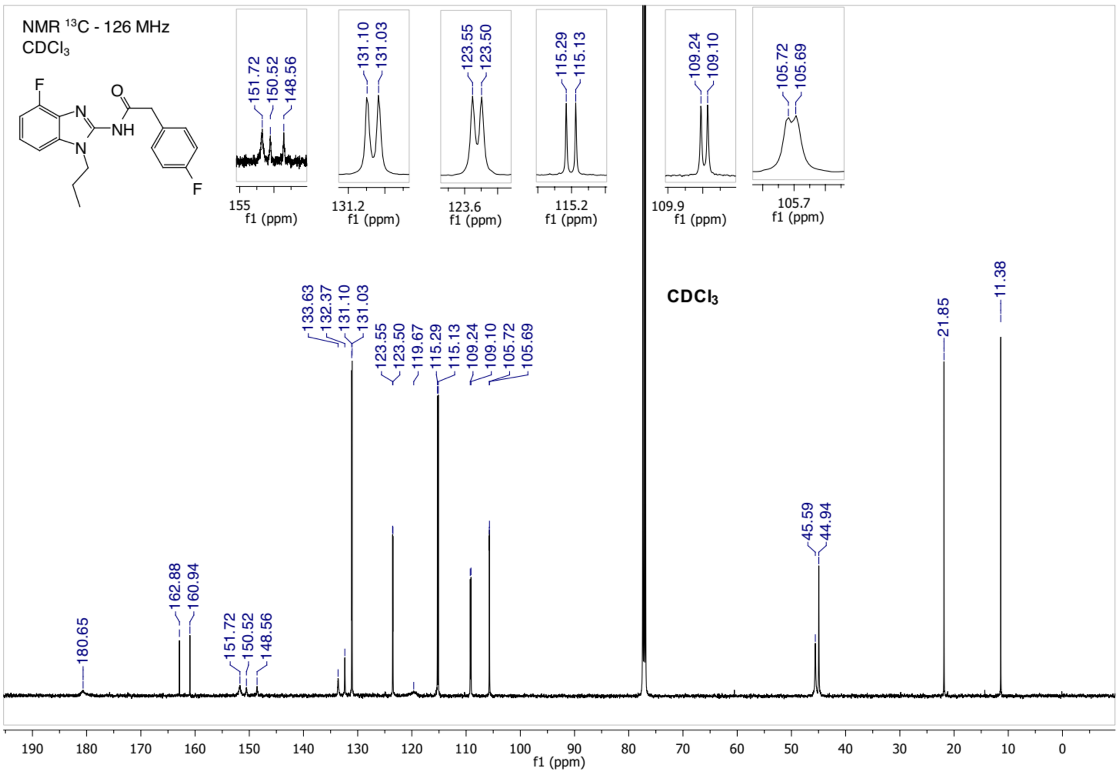

^{13}C NMR of 5 (126 MHz, CDCl$_3$)

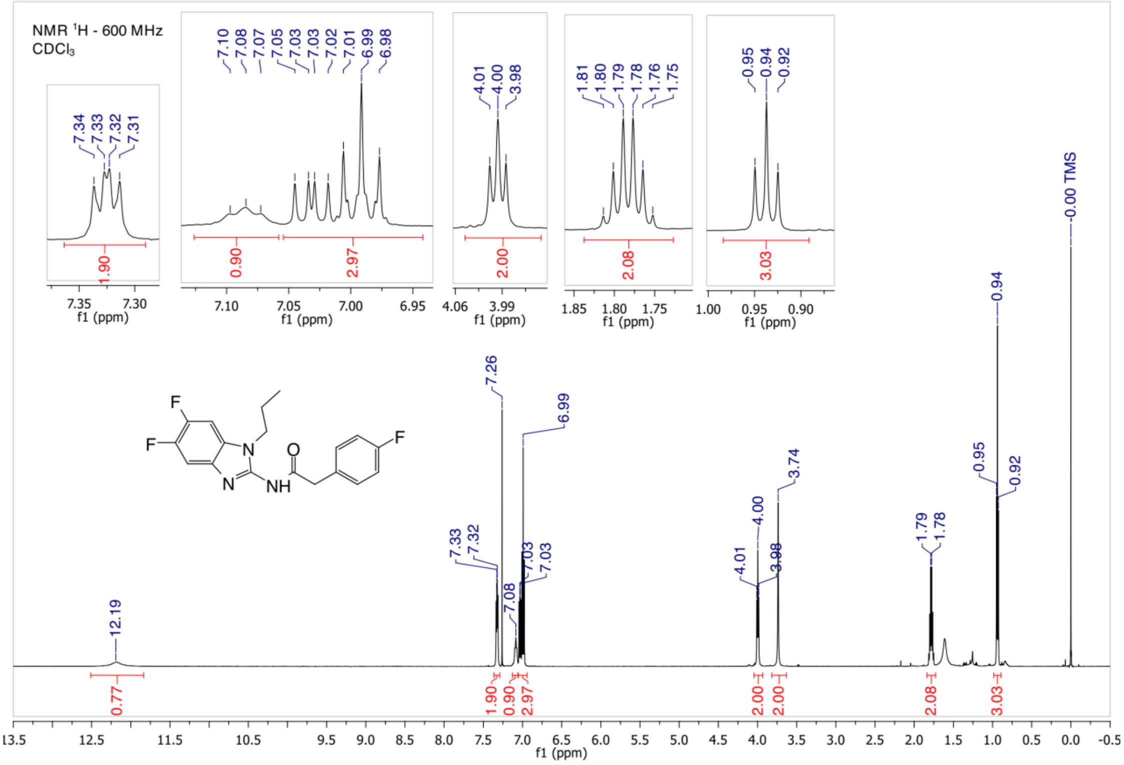

^{1}H NMR of 6 (600 MHz, $CDCl_3$)

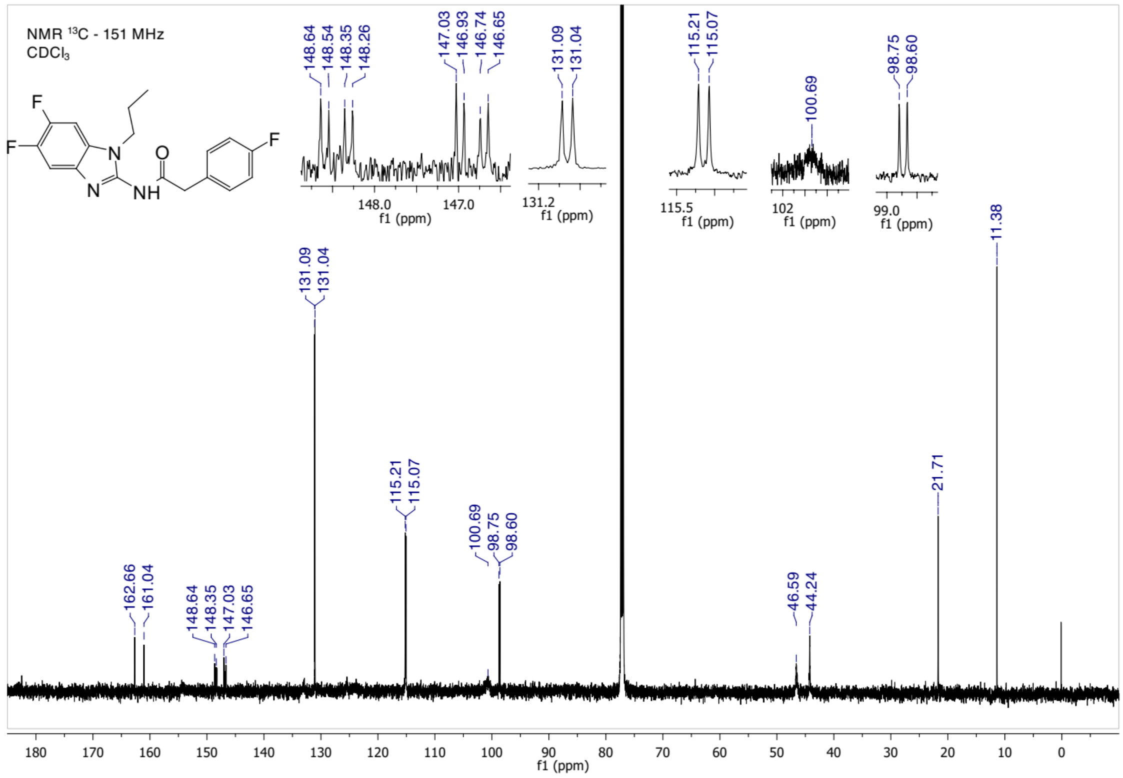

^{13}C NMR of 6 (151 MHz, $CDCl_3$)

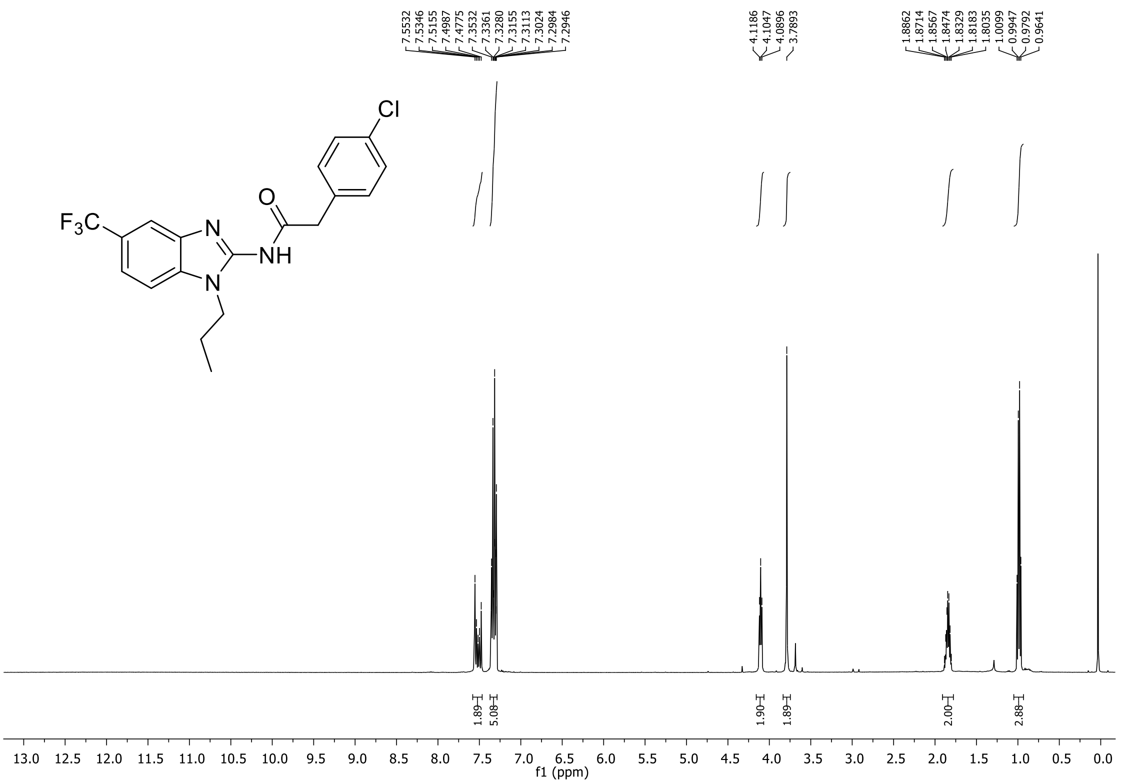

^{1}H NMR of 7 (500 MHz, $CDCl_3$)

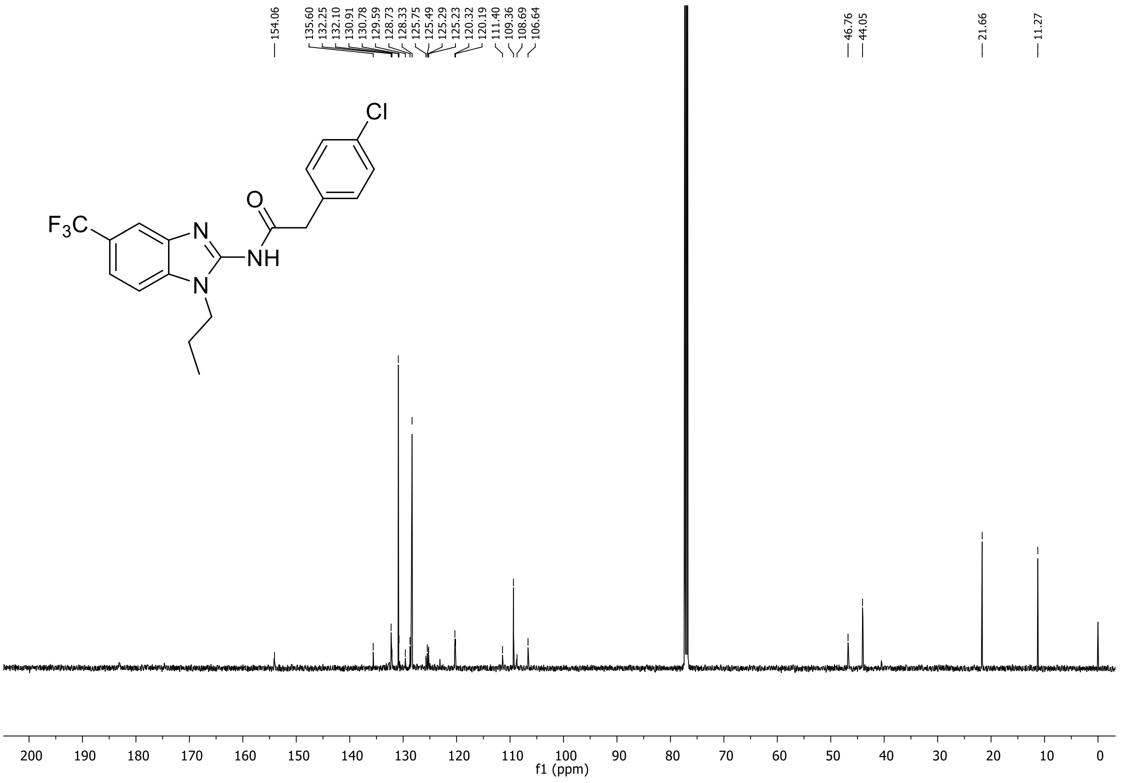

^{13}C NMR of 7 (126 MHz, $CDCl_3$)

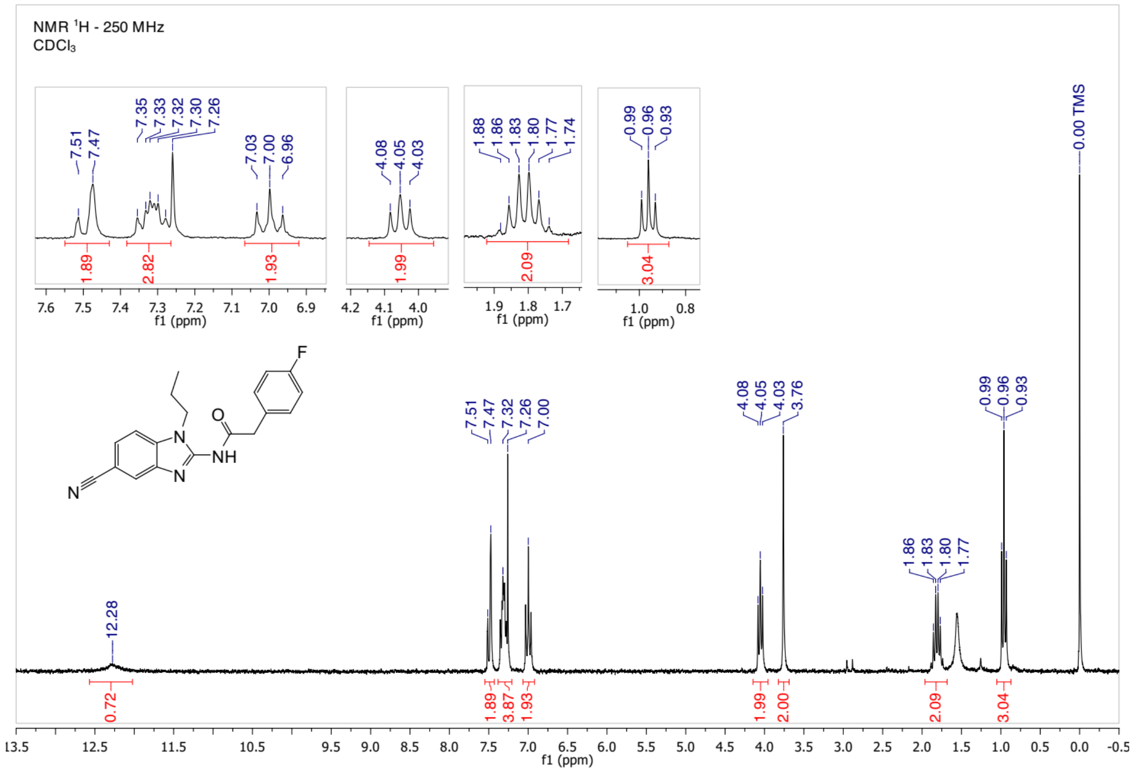

^{1}H NMR of 8 (250 MHz, $CDCl_3$)

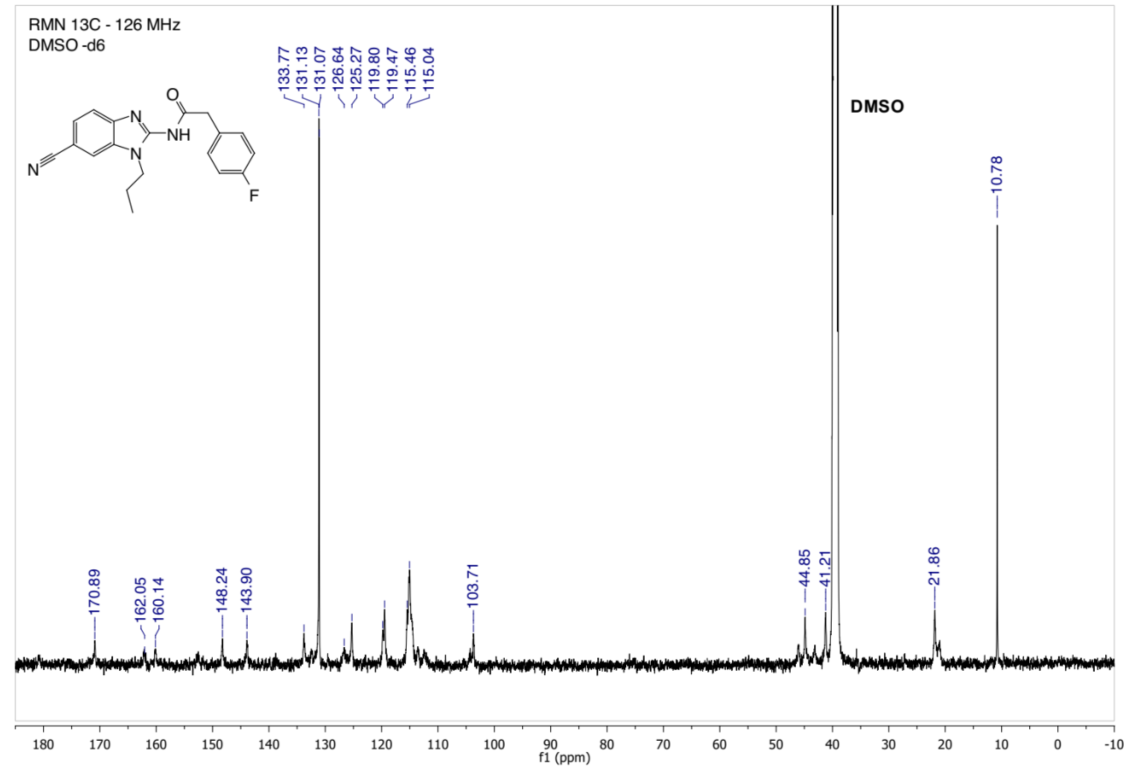

^{13}C NMR of 8 (126 MHz, DMSO-d_6)

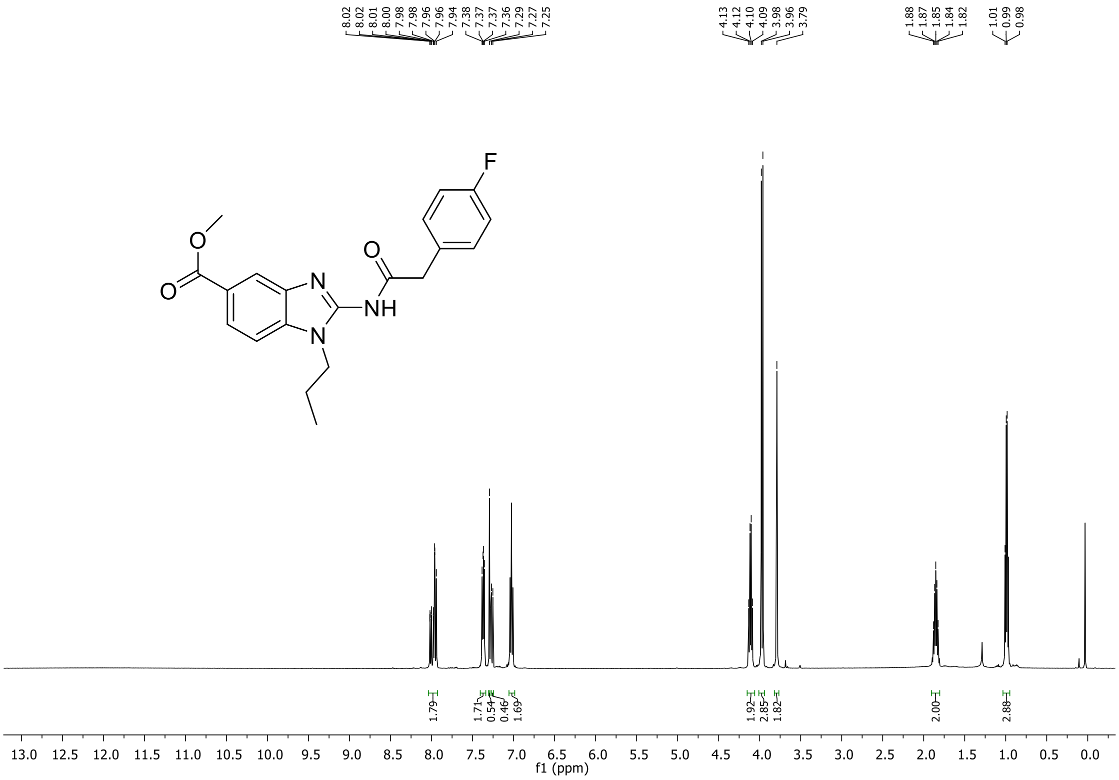

^{1}H NMR of 9 (500 MHz, $CDCl_3$)

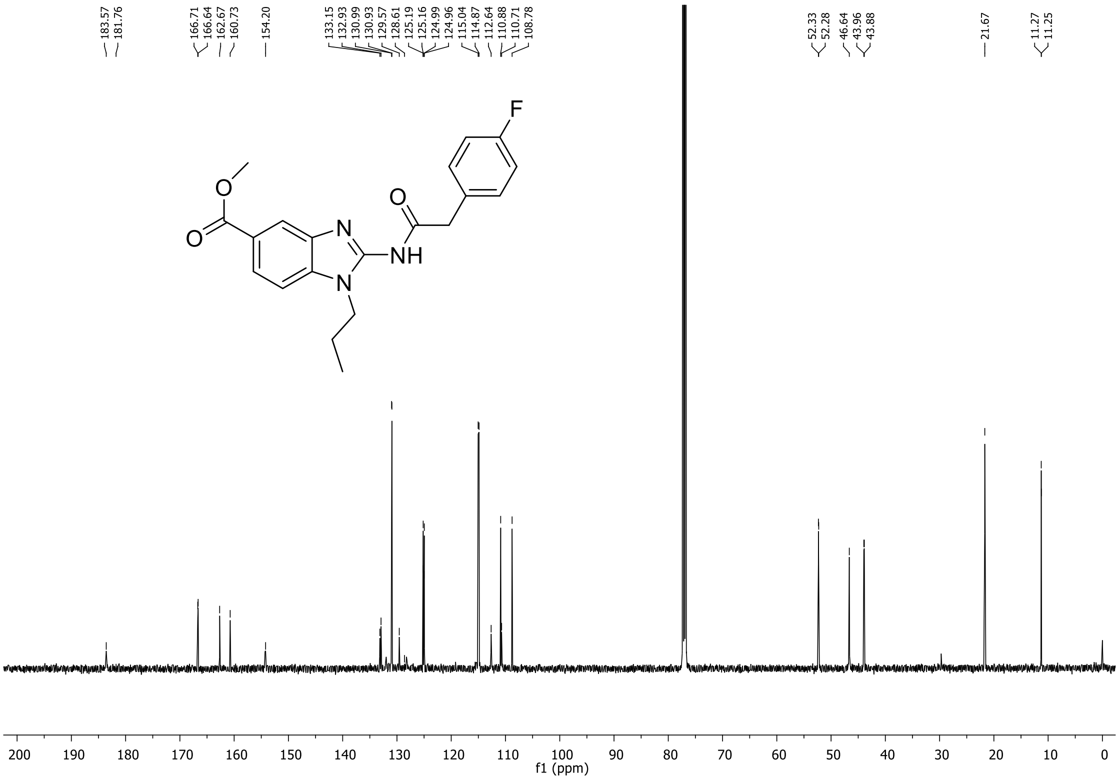

^{13}C NMR of 9 (126 MHz, $CDCl_3$)

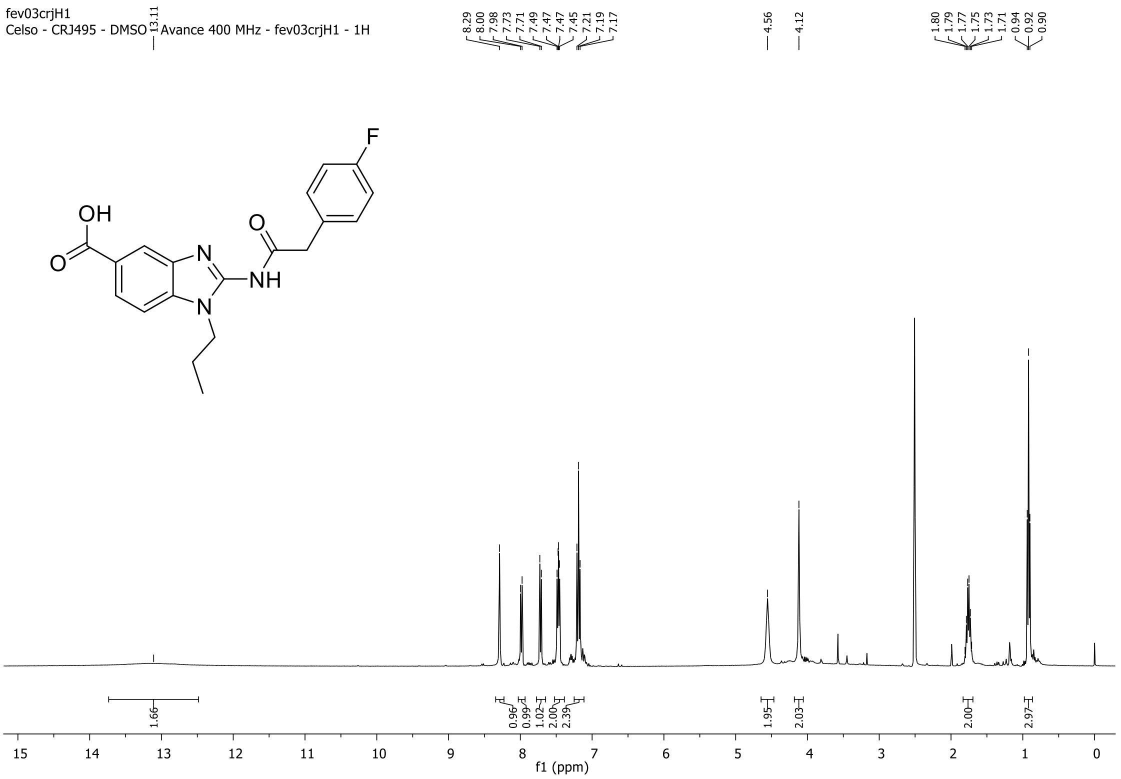

^{1}H NMR of 10 (400 MHz, DMSO)

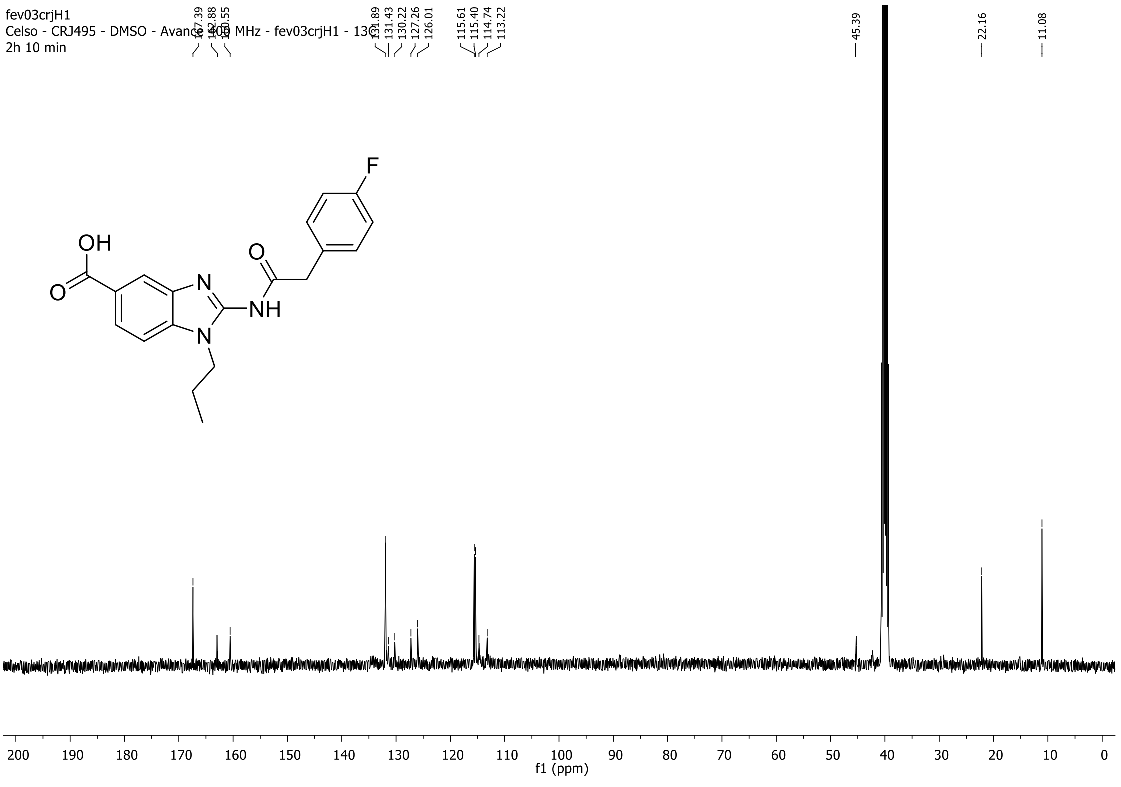

^{13}C NMR of 10 (101 MHz, DMSO)

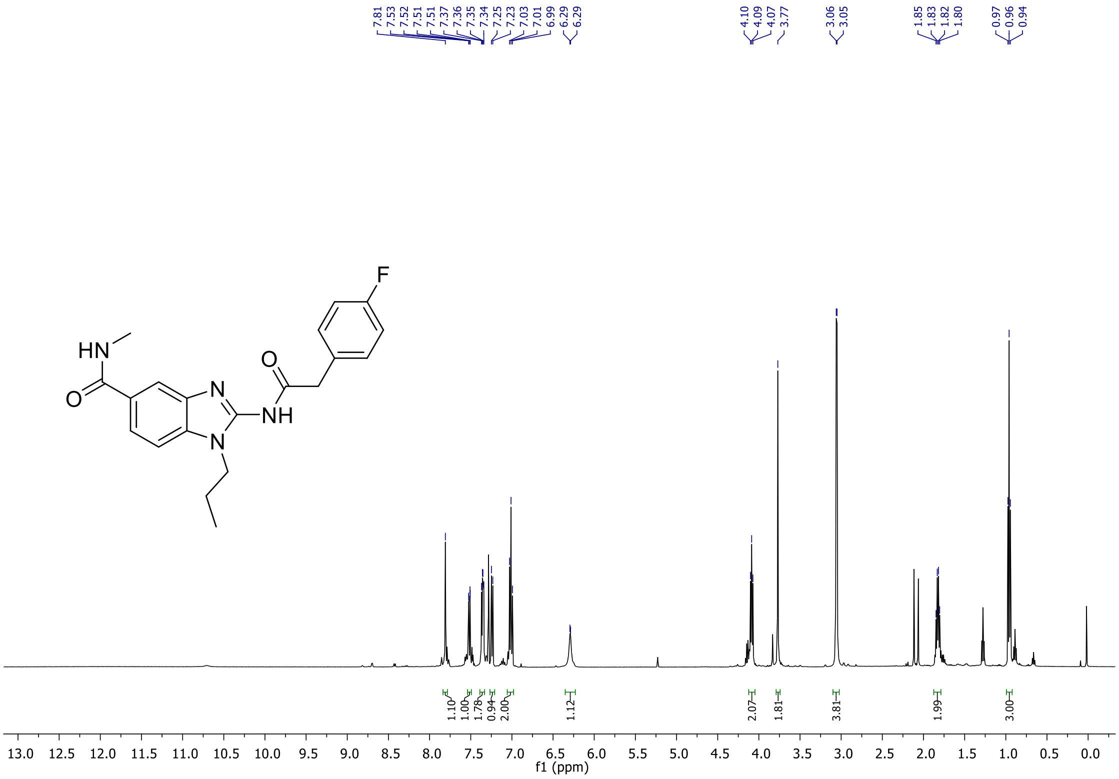

^{1}H NMR of 11 (500 MHz, CDCl$_3$)

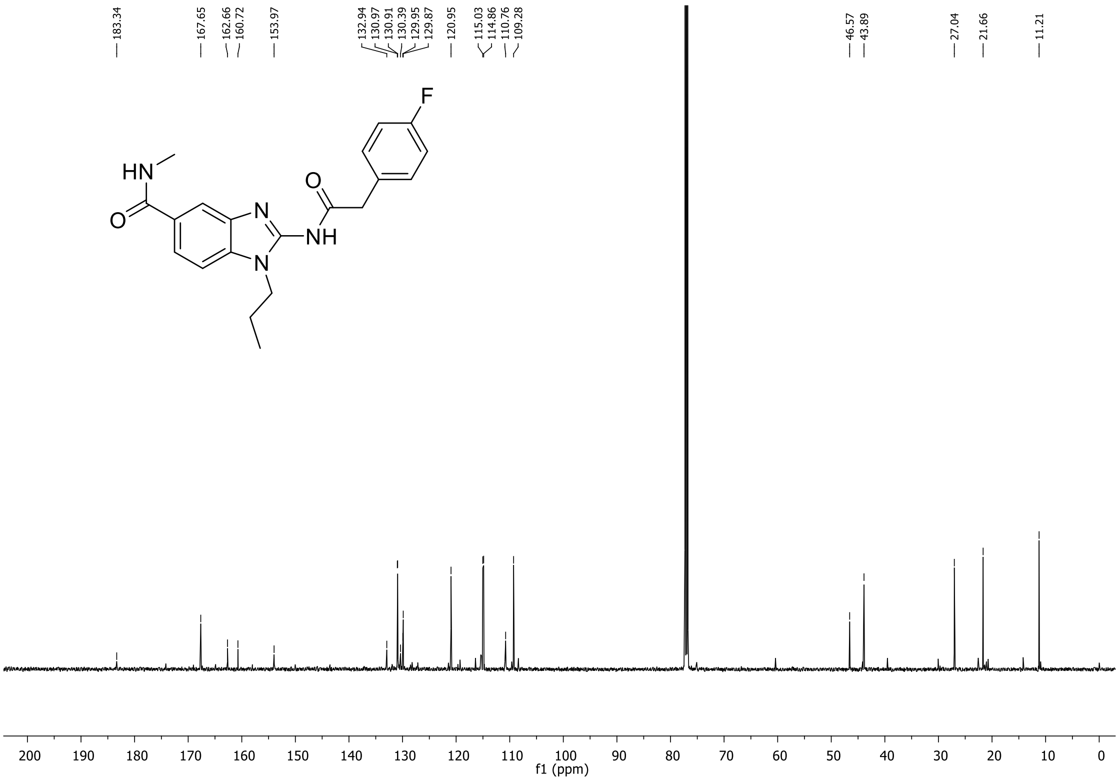

^{13}C NMR of 11 (126 MHz, CDCl$_3$)

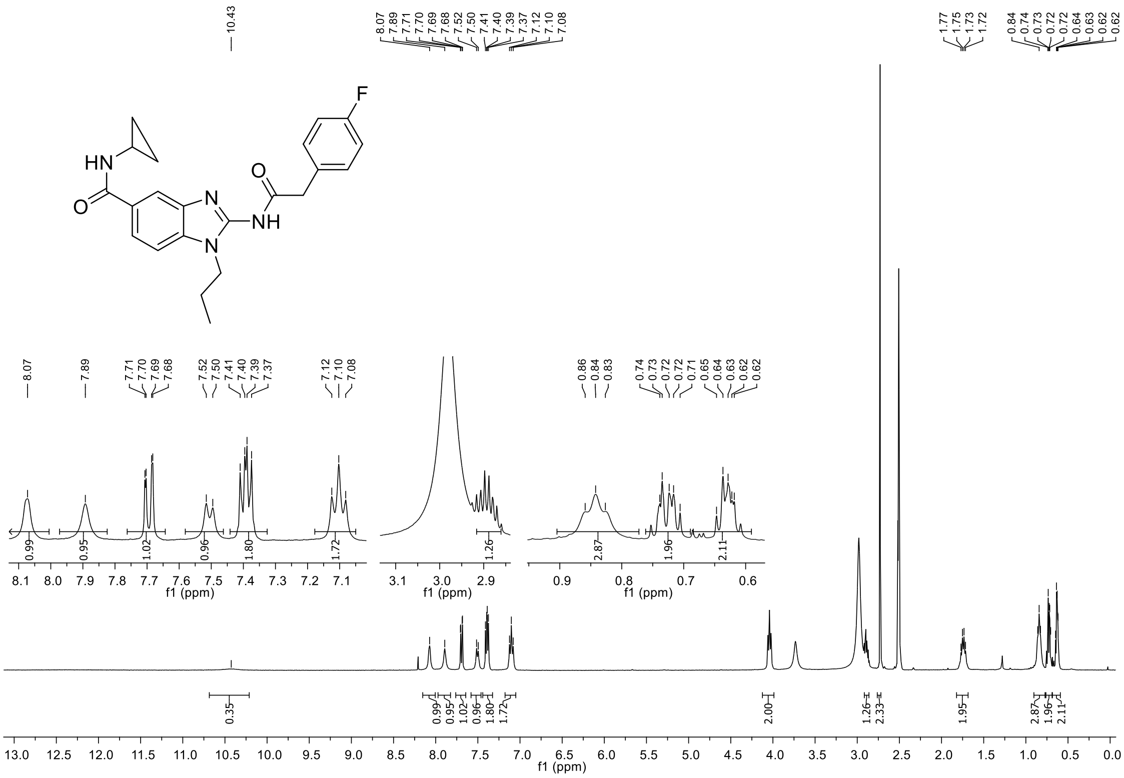

^{1}H NMR of 12 (500 MHz, DMSO)

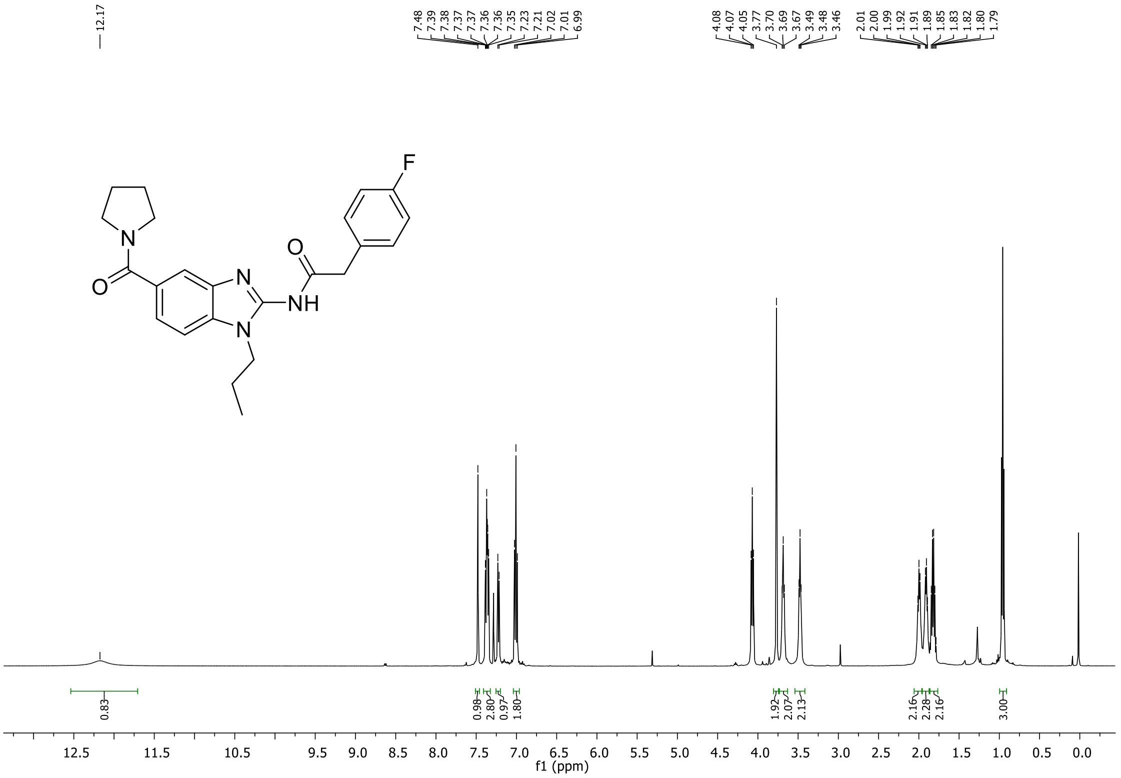

^{1}H NMR of 13 (500 MHz, $CDCl_3$)

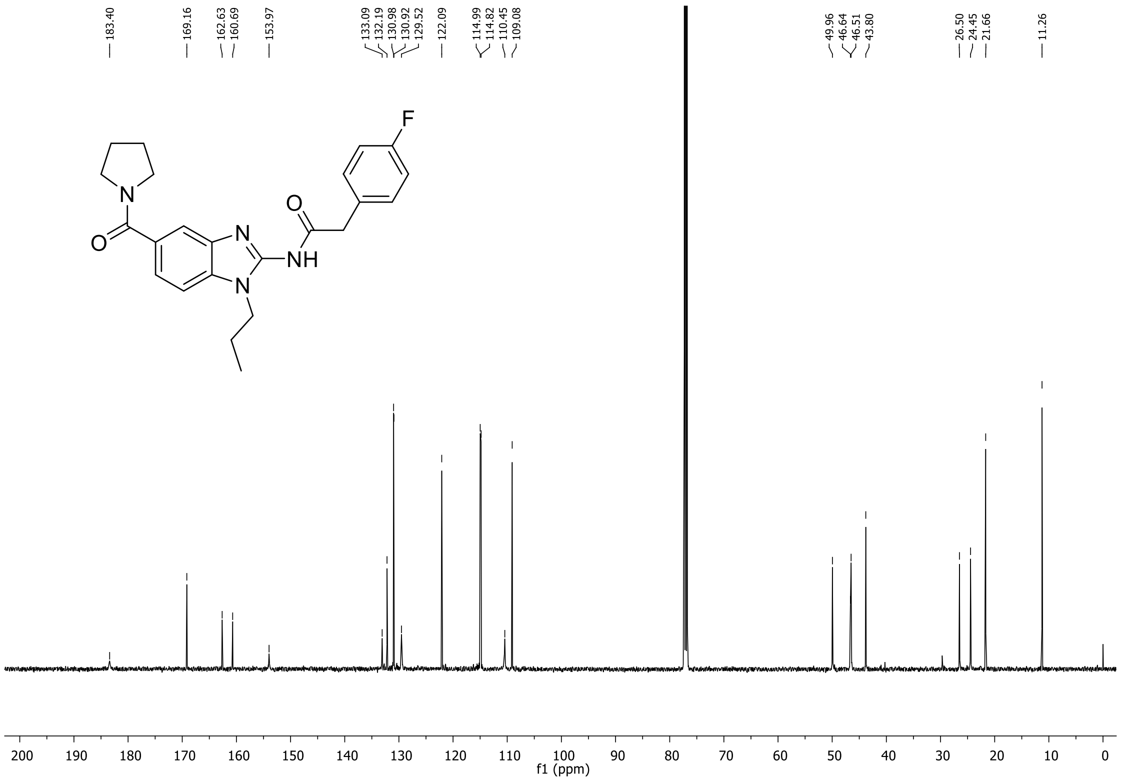

^{13}C NMR of 13 (126 MHz, CDCl$_3$)

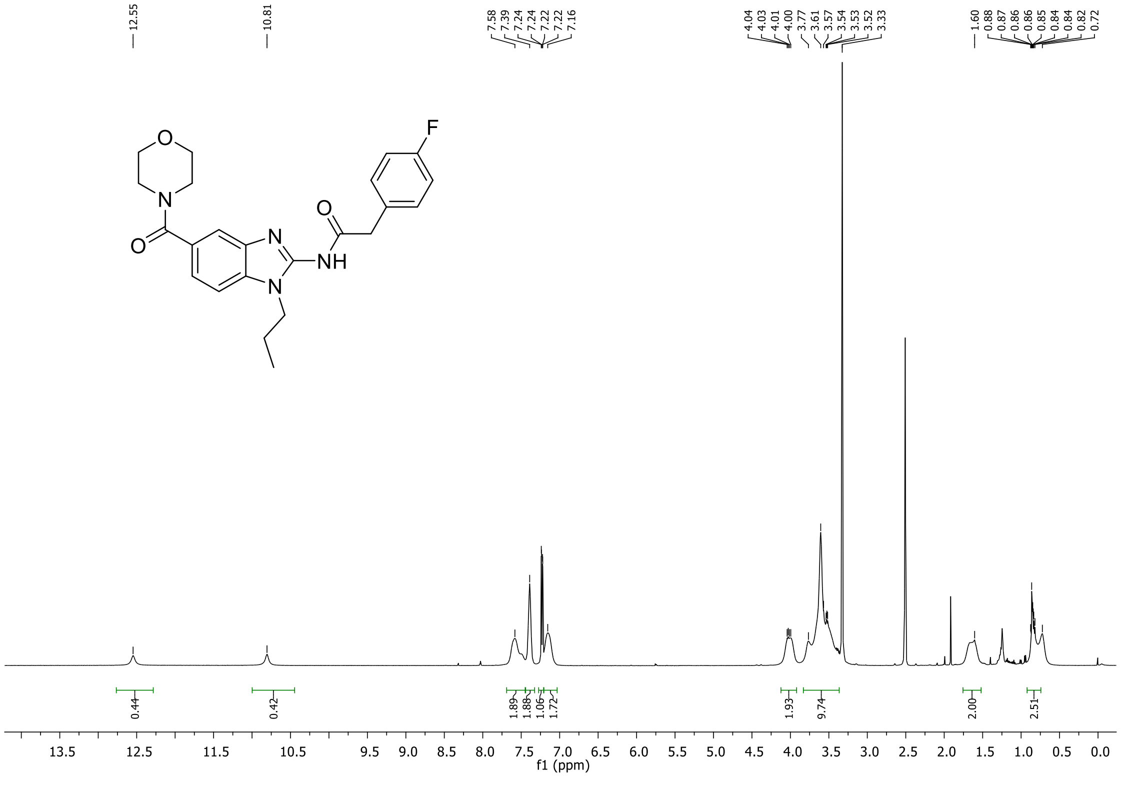

^{1}H NMR of 14 (500 MHz, DMSO)

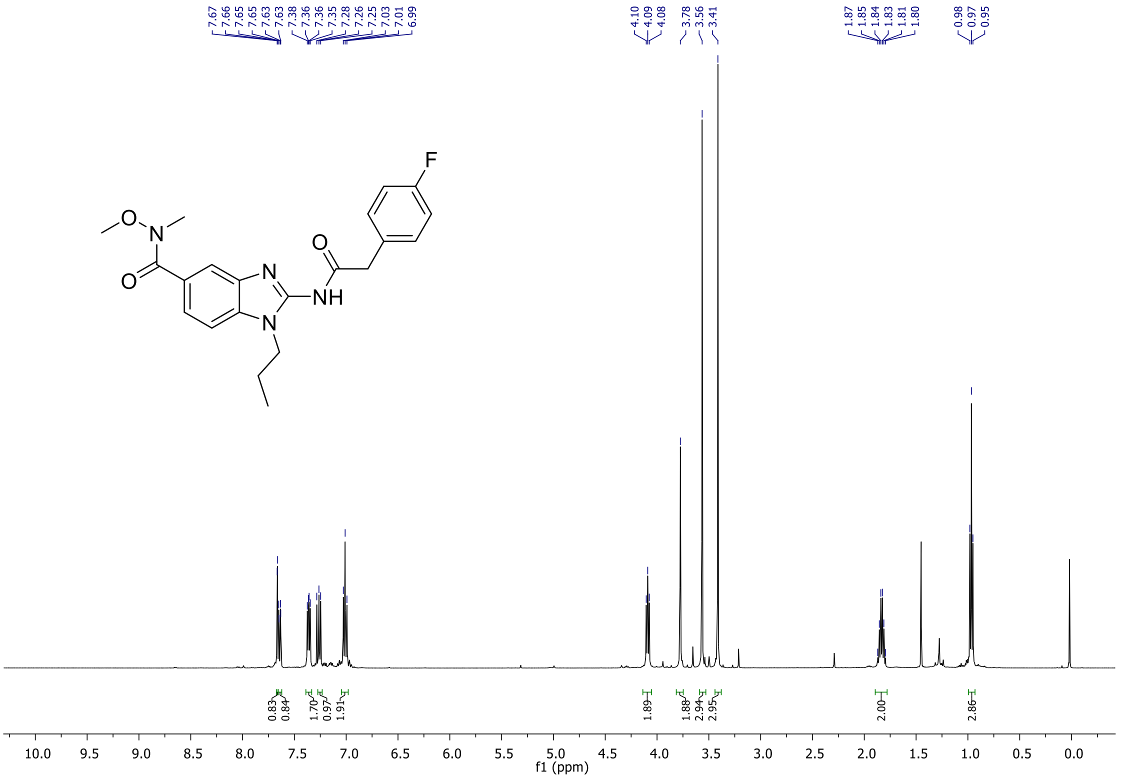

^{1}H NMR of 15 (500 MHz, CDCl₃)

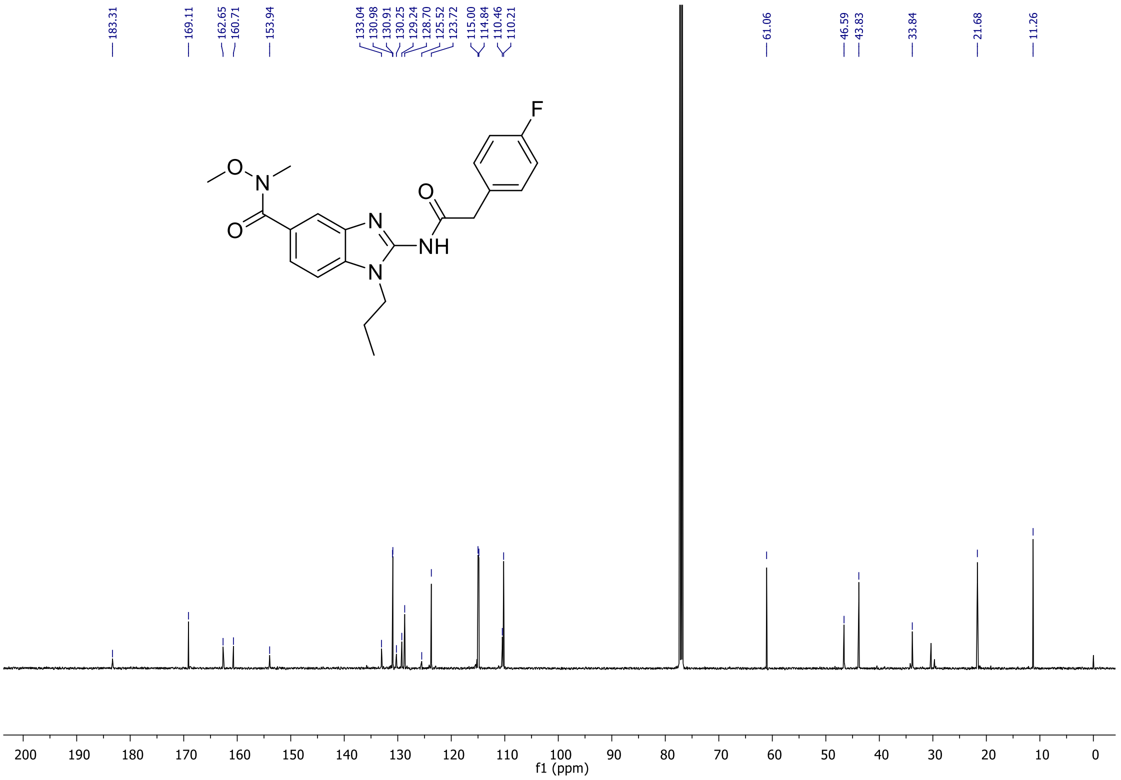

^{13}C NMR of 15 (126 MHz, CDCl₃)

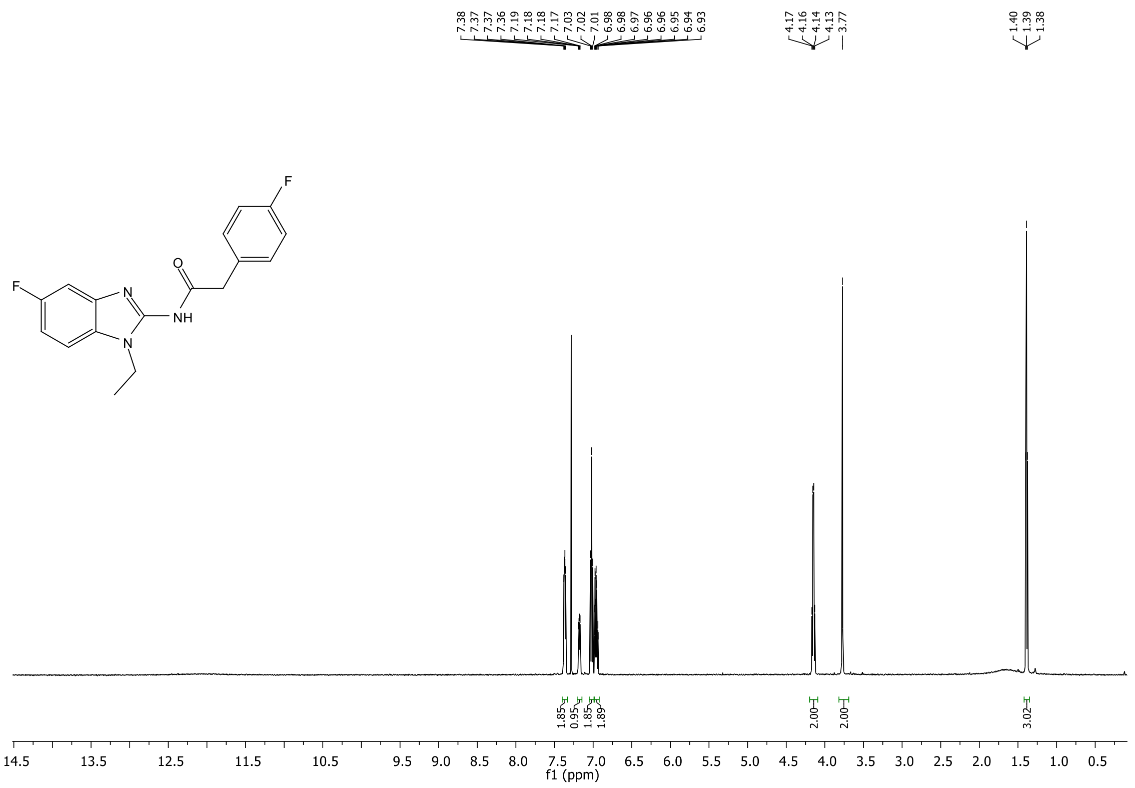

^{1}H NMR of 16 (600 MHz, CDCl$_3$)

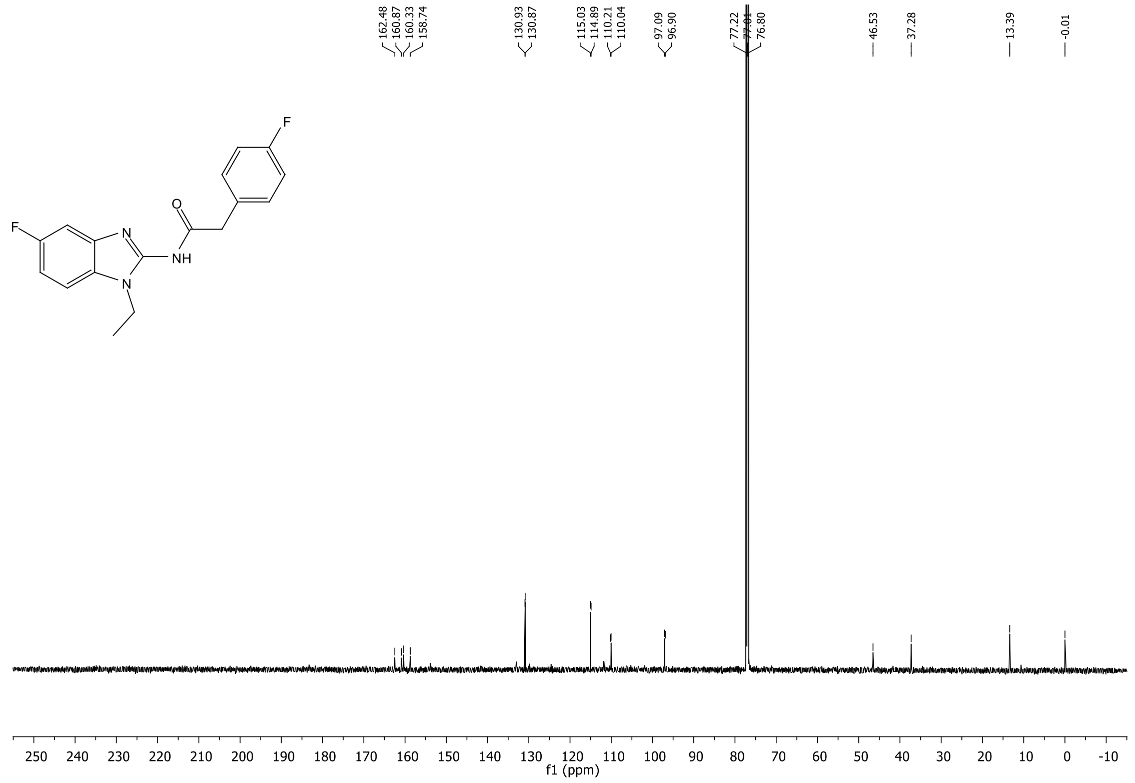

^{13}C NMR of 16 (150 MHz, CDCl$_3$)

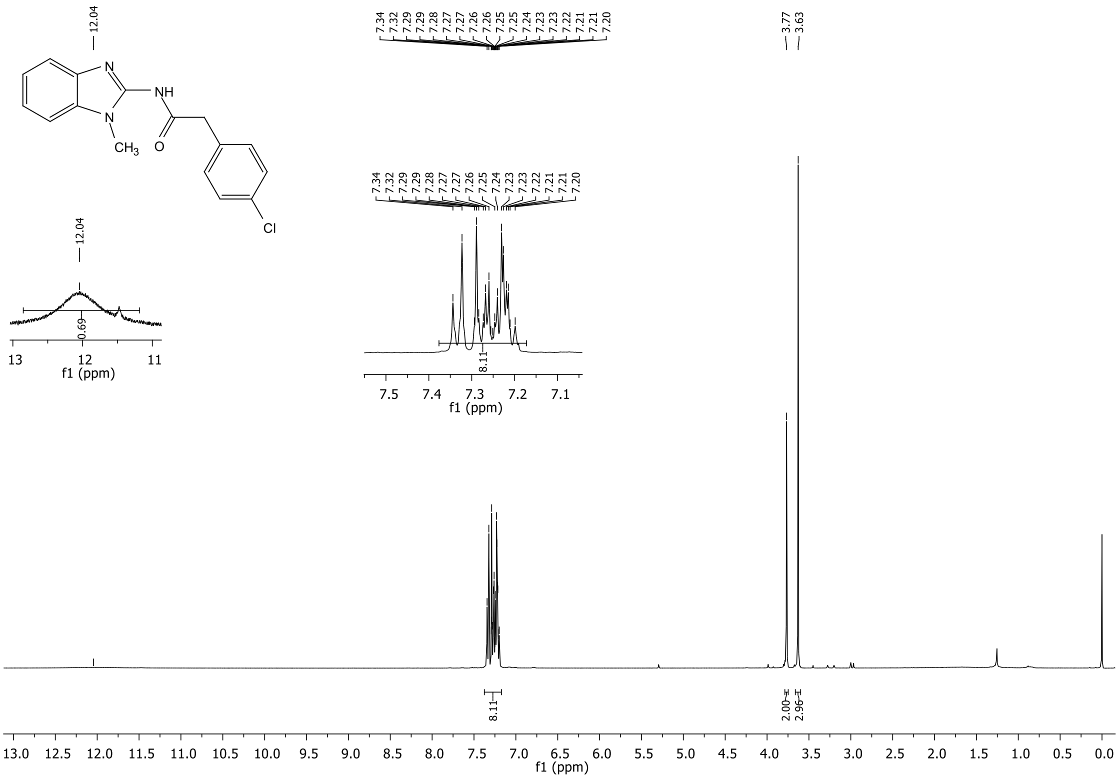

^{1}H NMR of 17 (400 MHz, $CDCl_3$)

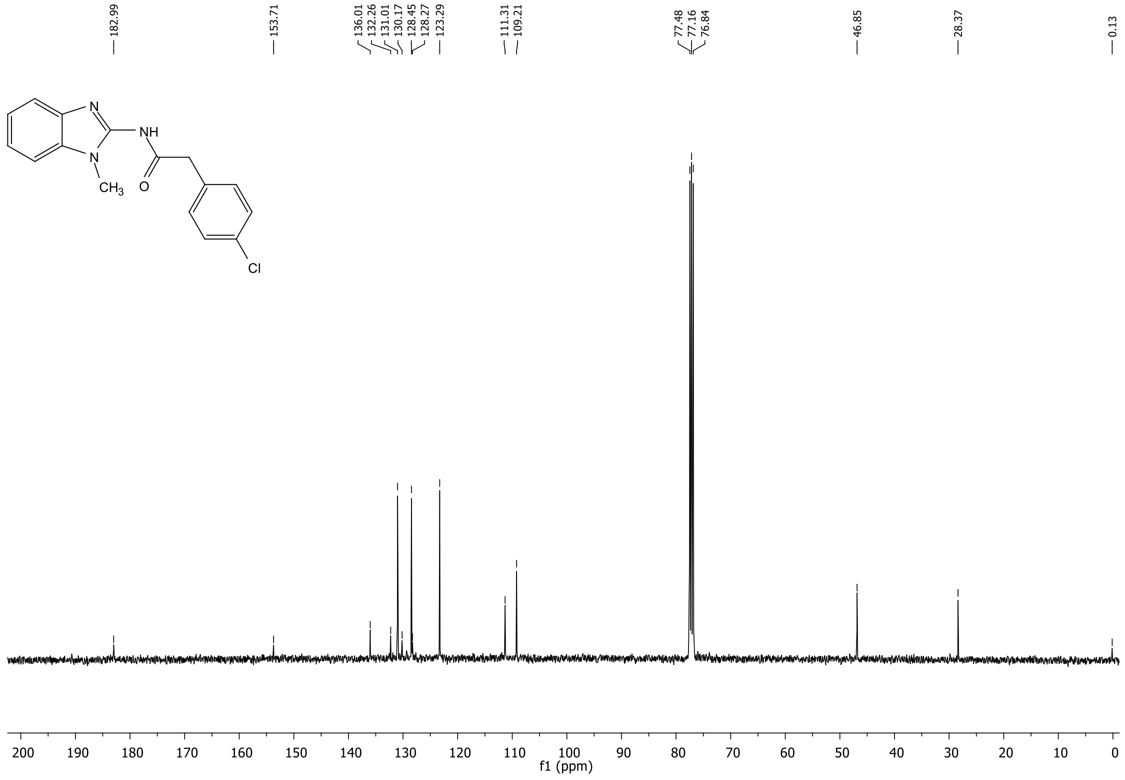

^{13}C NMR of 17 (101 MHz, $CDCl_3$)

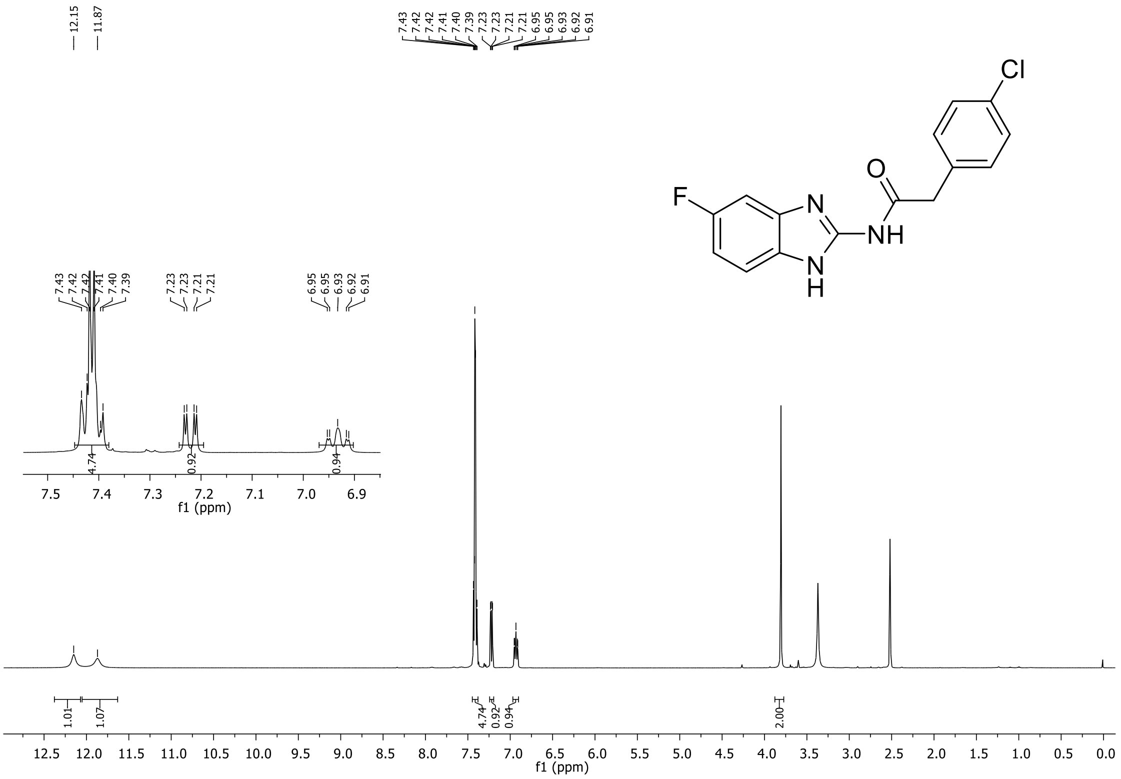

^{1}H NMR of 18 (500 MHz, DMSO)

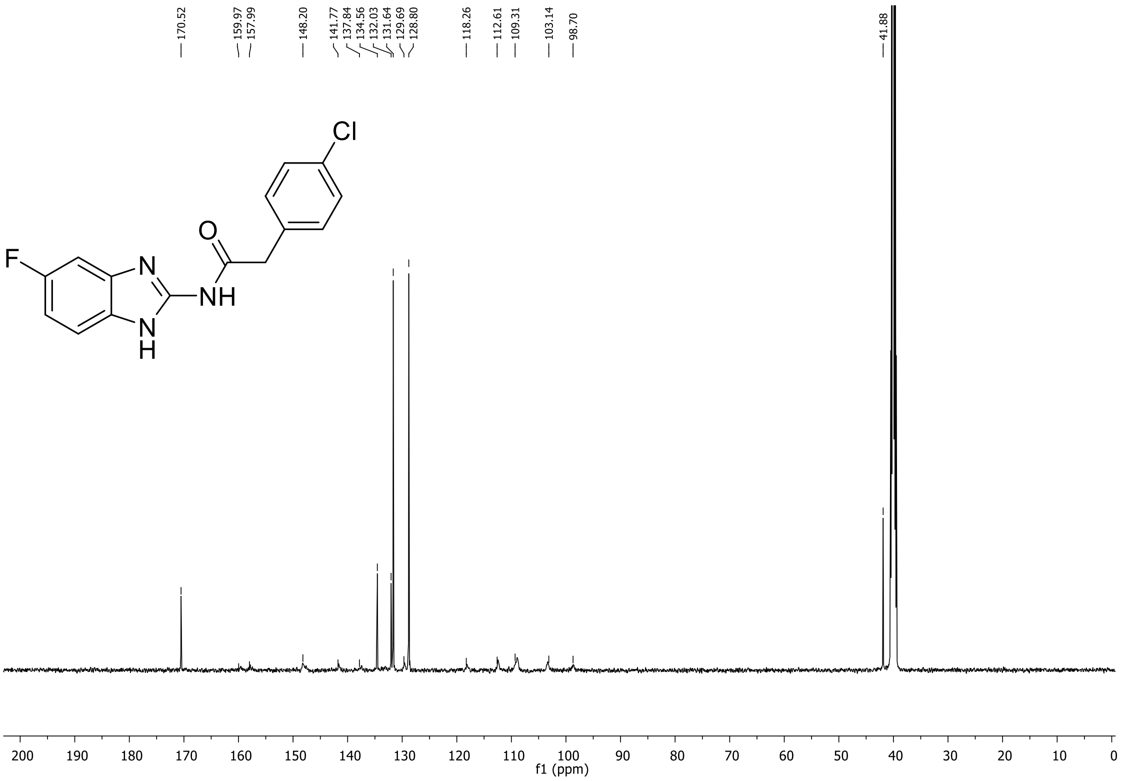

^{13}C NMR of 18 (126 MHz, DMSO)

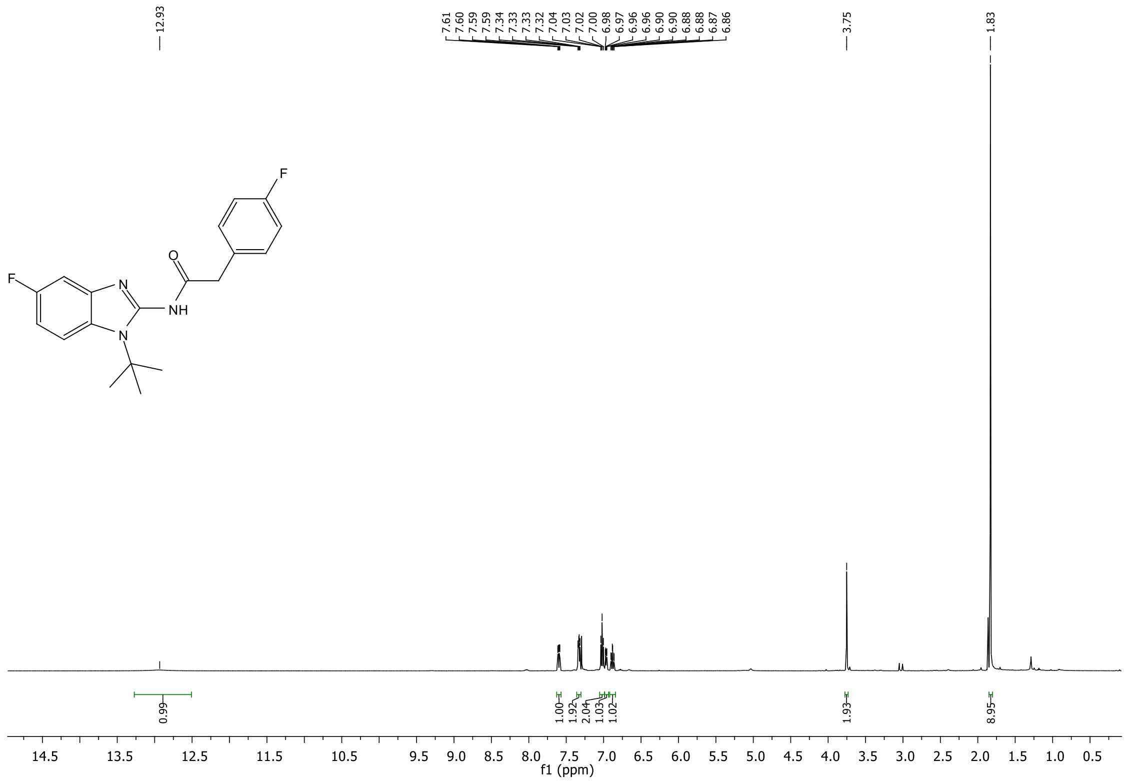

^{1}H NMR of 19 (500 MHz, $CDCl_3$)

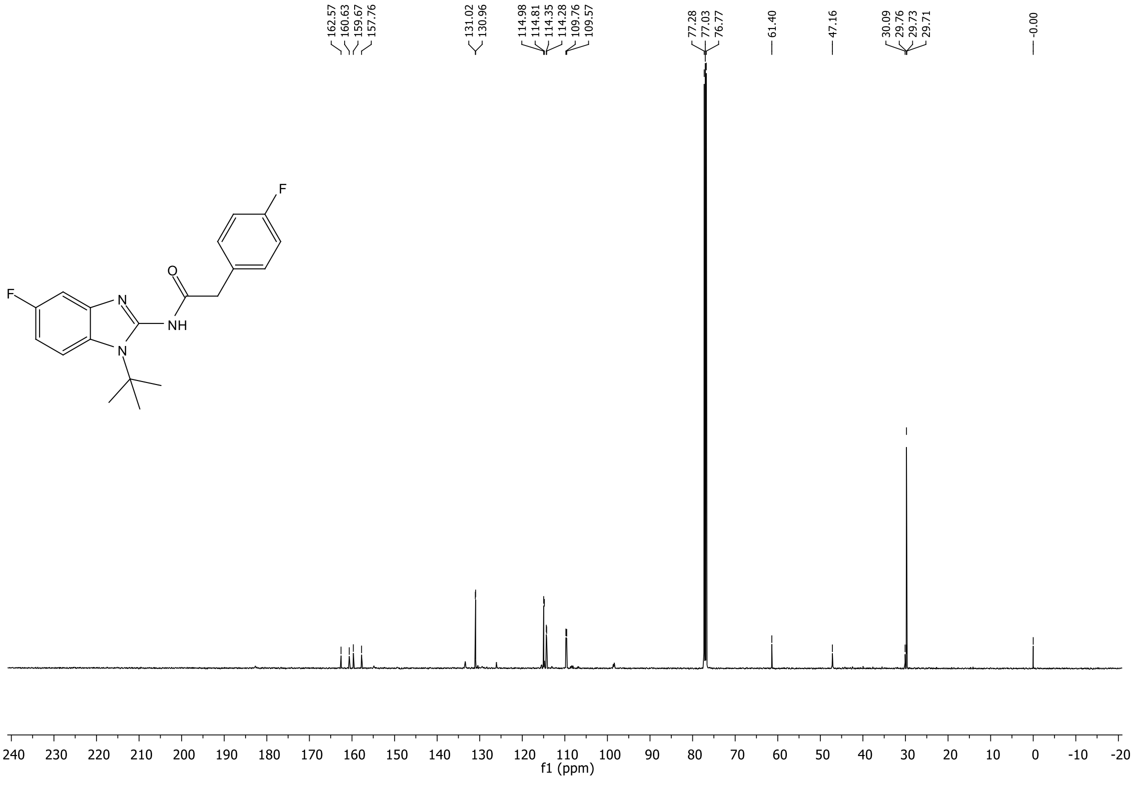

^{13}C NMR of 19 (126 MHz, $CDCl_3$)

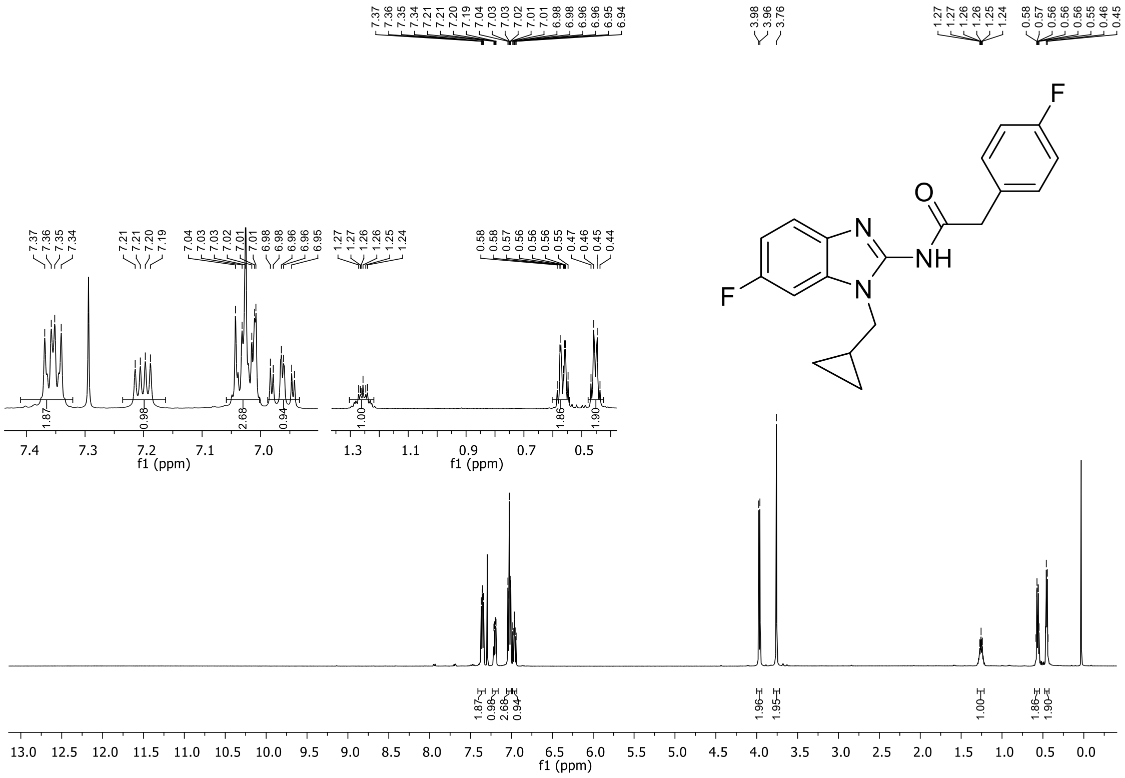

¹H NMR of 21 (500 MHz, $CDCl_3$)

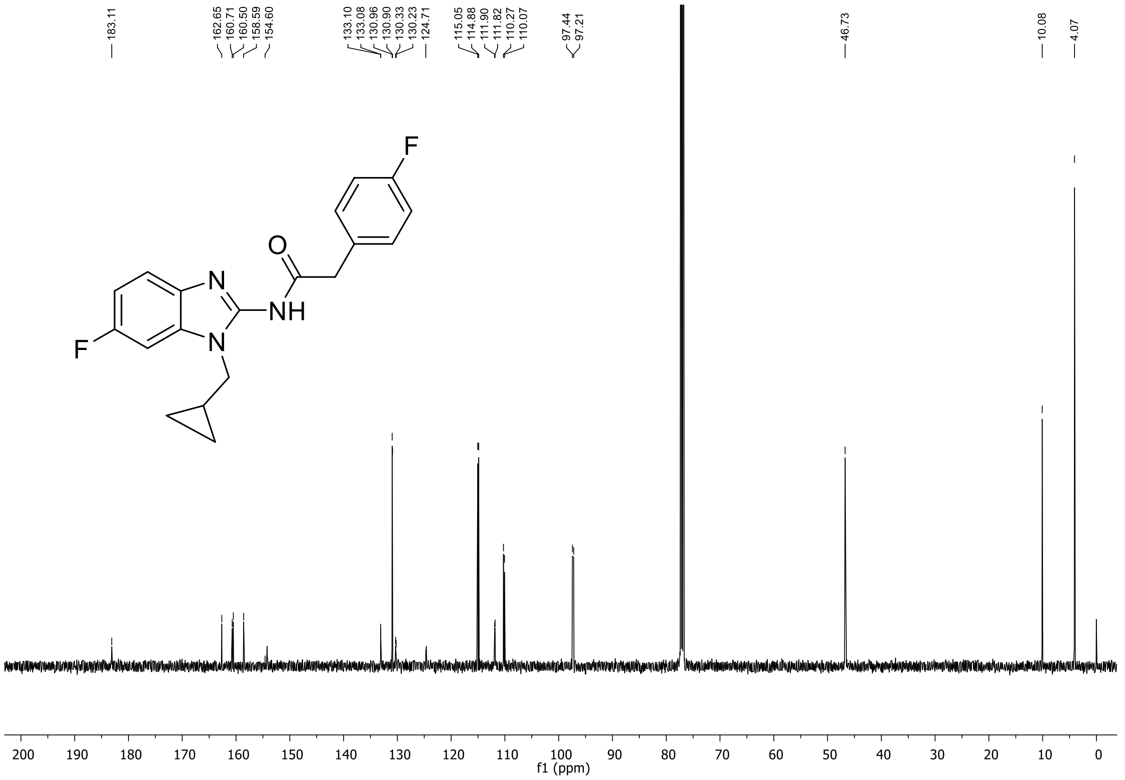

^{13}C NMR of 21 (126 MHz, $CDCl_3$)

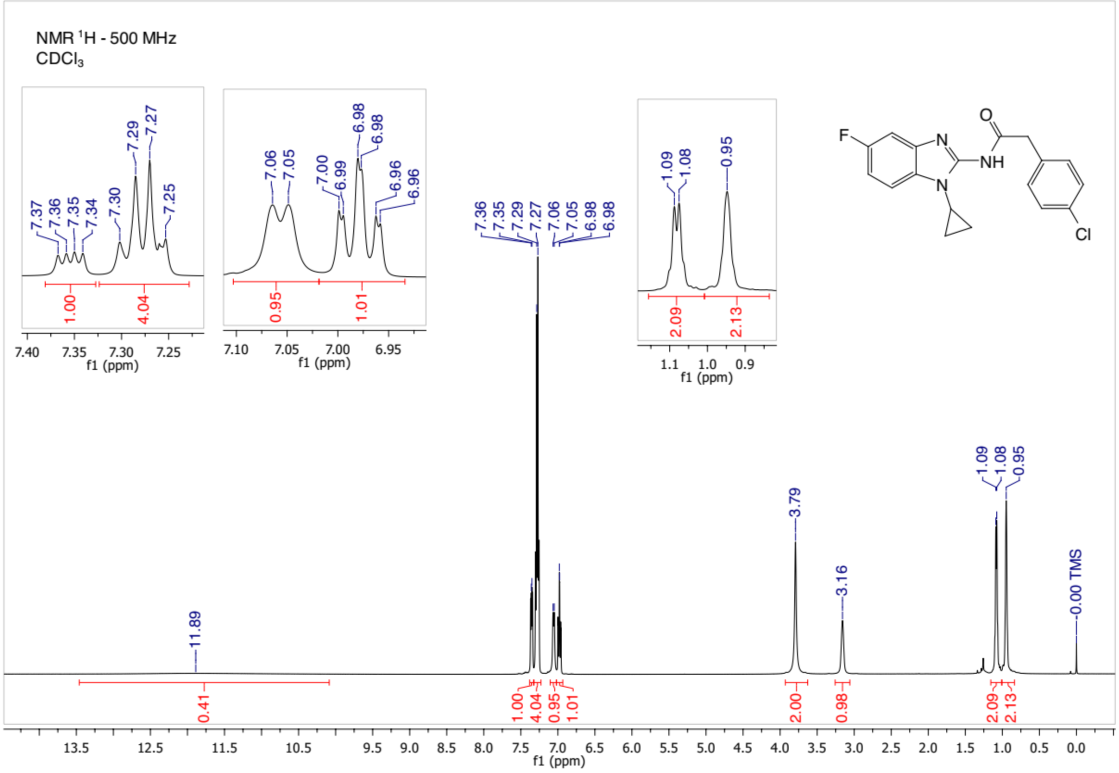

^{1}H NMR of 22 (500 MHz, $CDCl_3$)

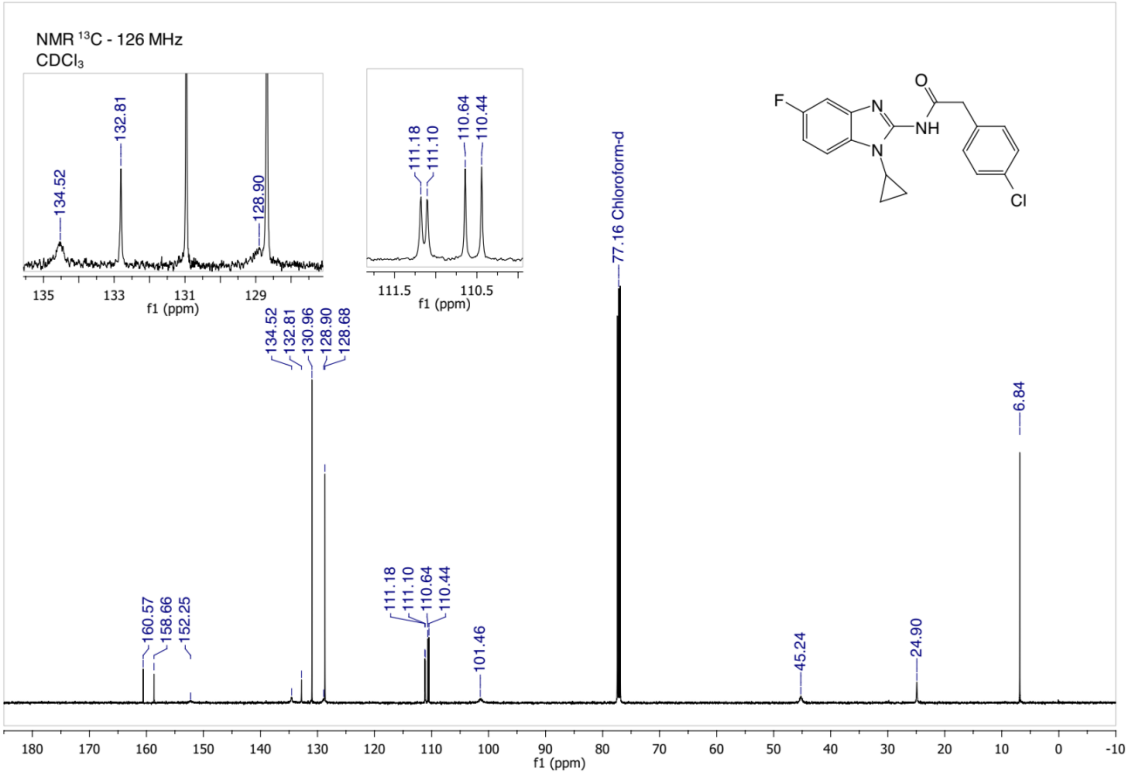

^{13}C NMR of 22 (126 MHz, $CDCl_3$)

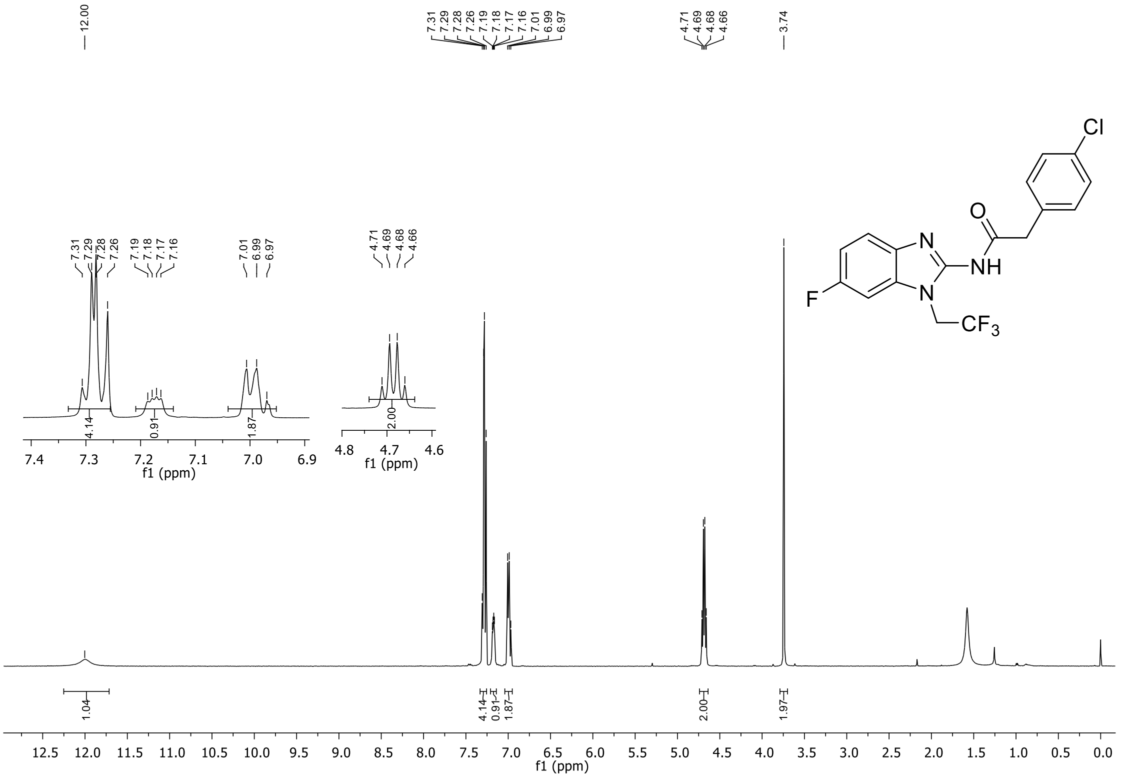

^{1}H NMR of 23 (500 MHz, $CDCl_3$)

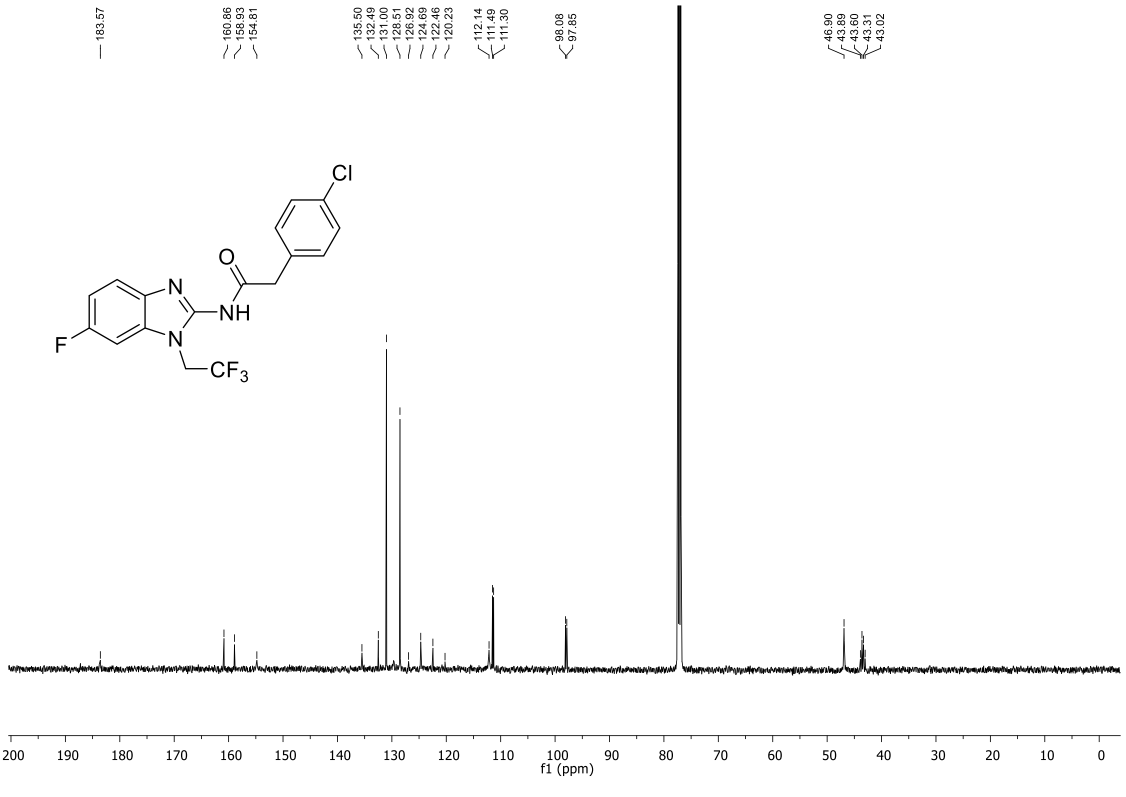

^{13}C NMR of 23 (126 MHz, $CDCl_3$)

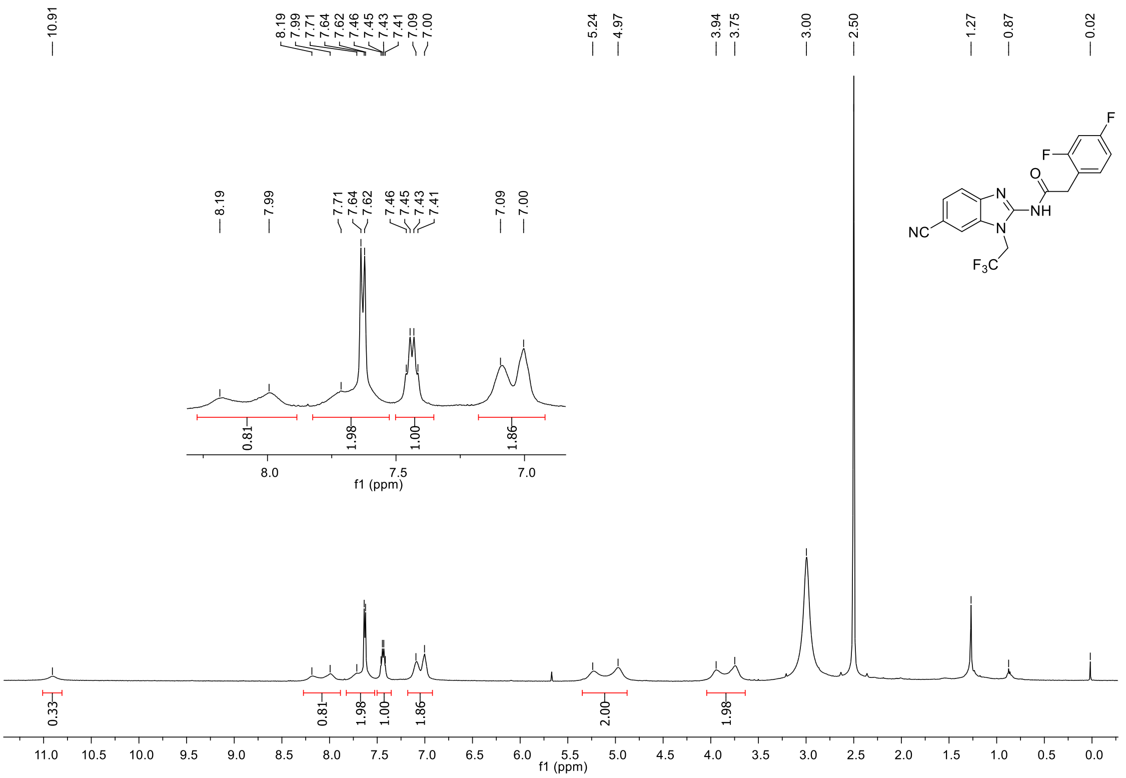

^{1}H NMR of 24 (500 MHz, 90°C, DMSO)

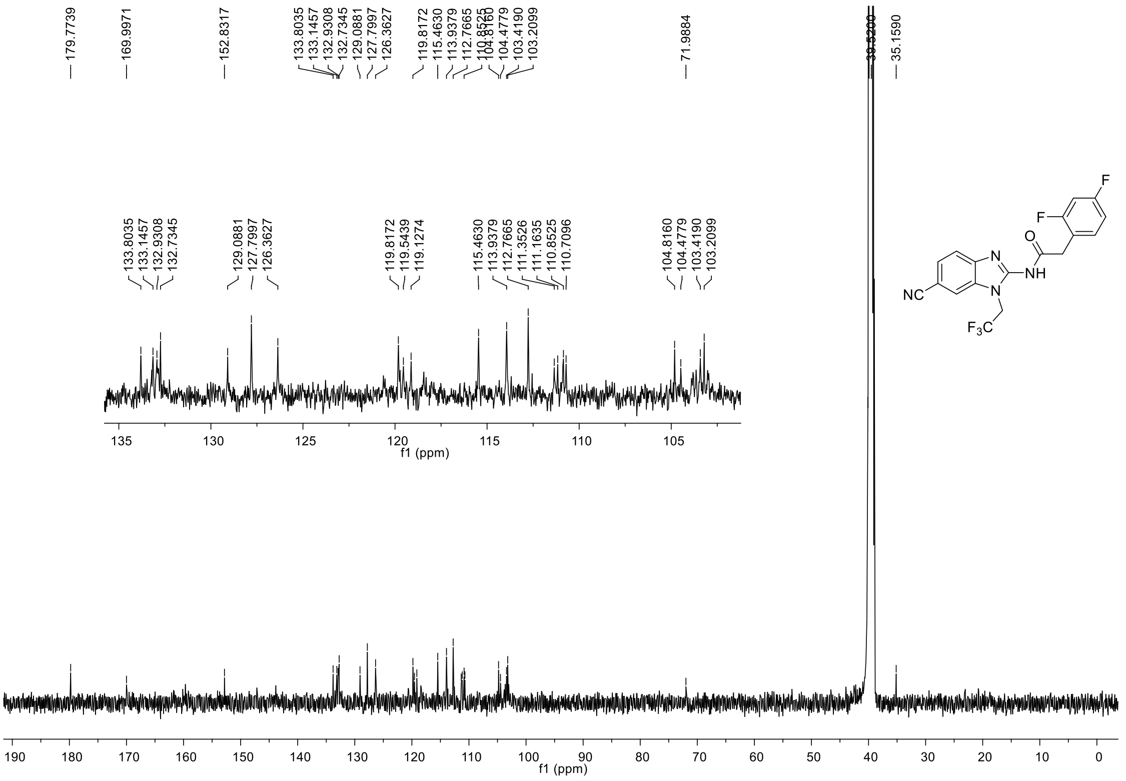

^{13}C NMR of 24 (126 MHz, DMSO)

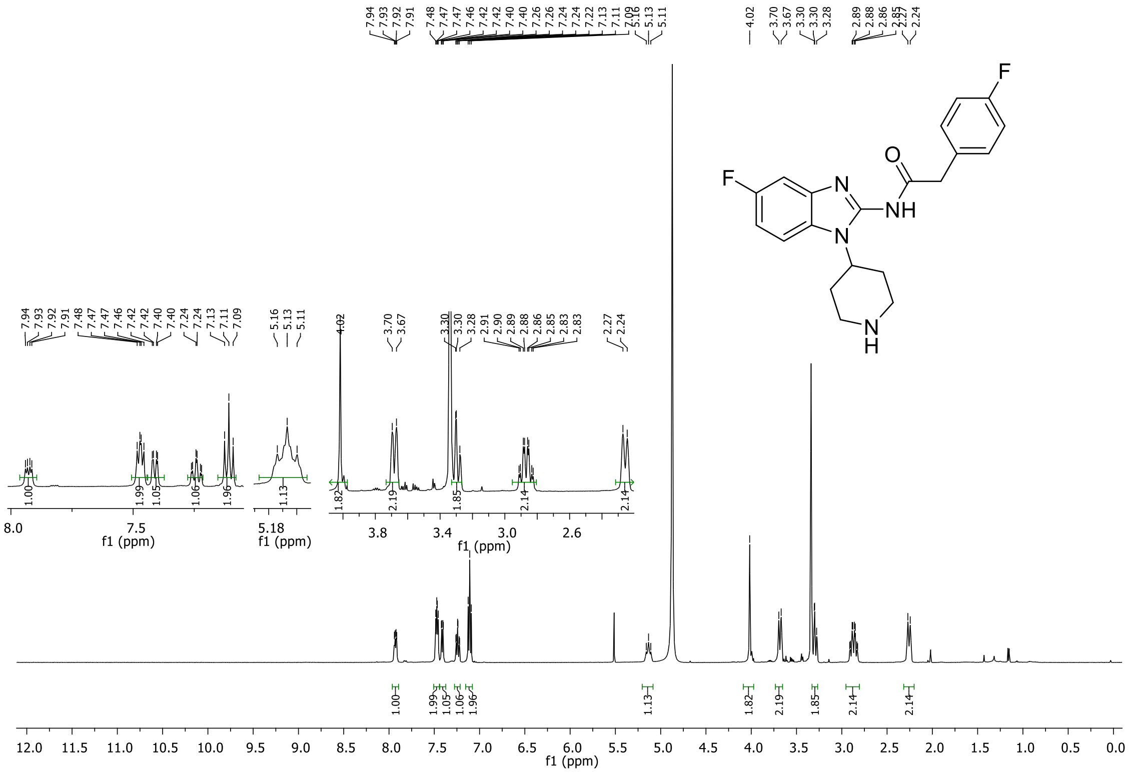

^{1}H NMR of 25 (500 MHz, MeOD)

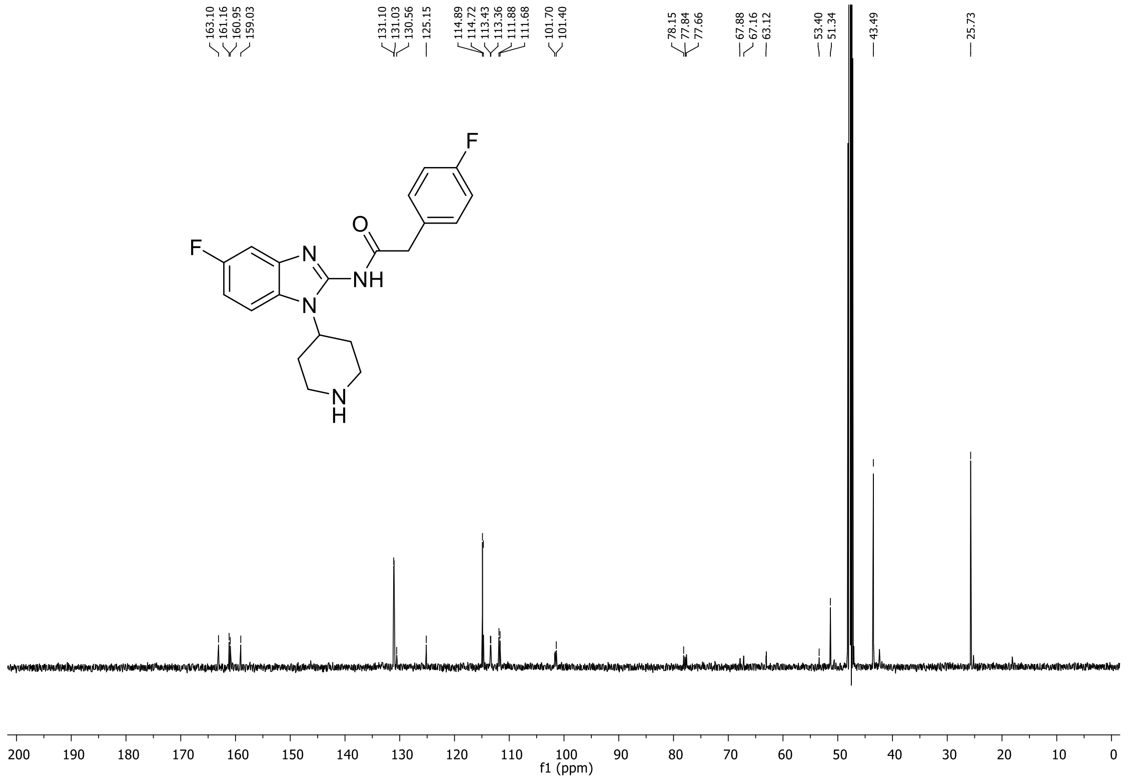

^{13}C NMR of 25 (126 MHz, MeOD)

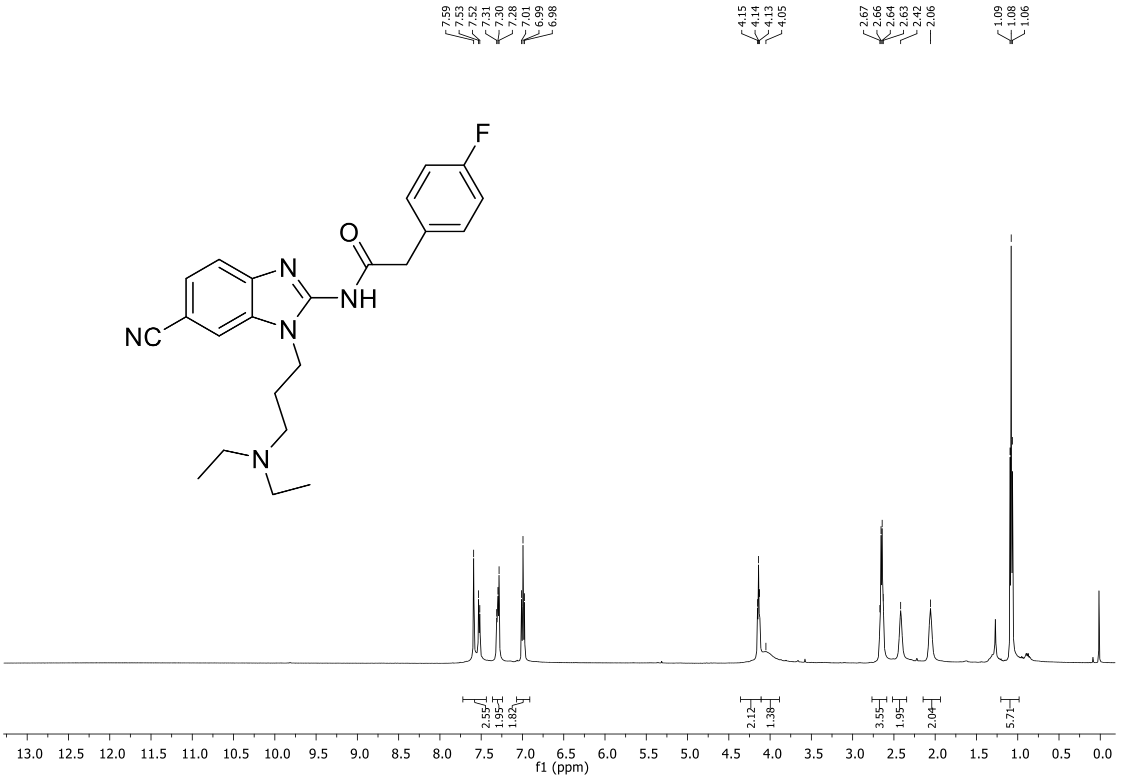

^{1}H NMR of 26 (500 MHz, CDCl$_3$)

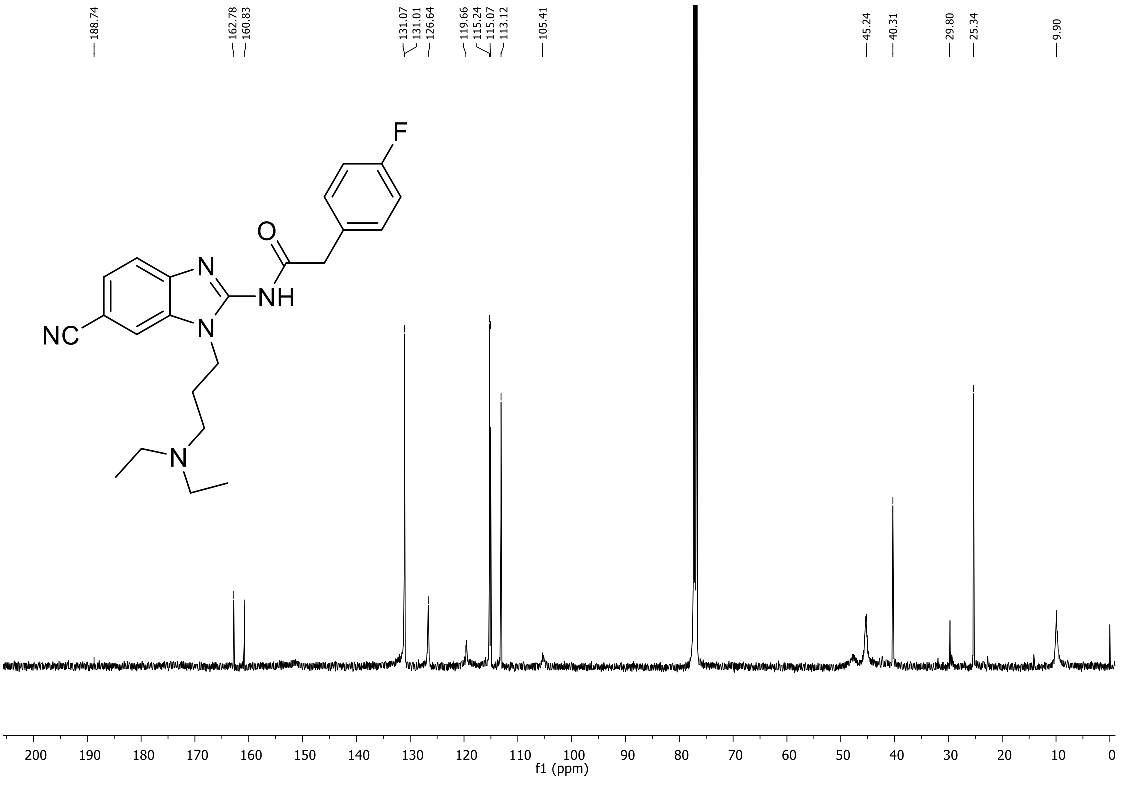

^{13}C NMR of 26 (126 MHz, CDCl$_3$)

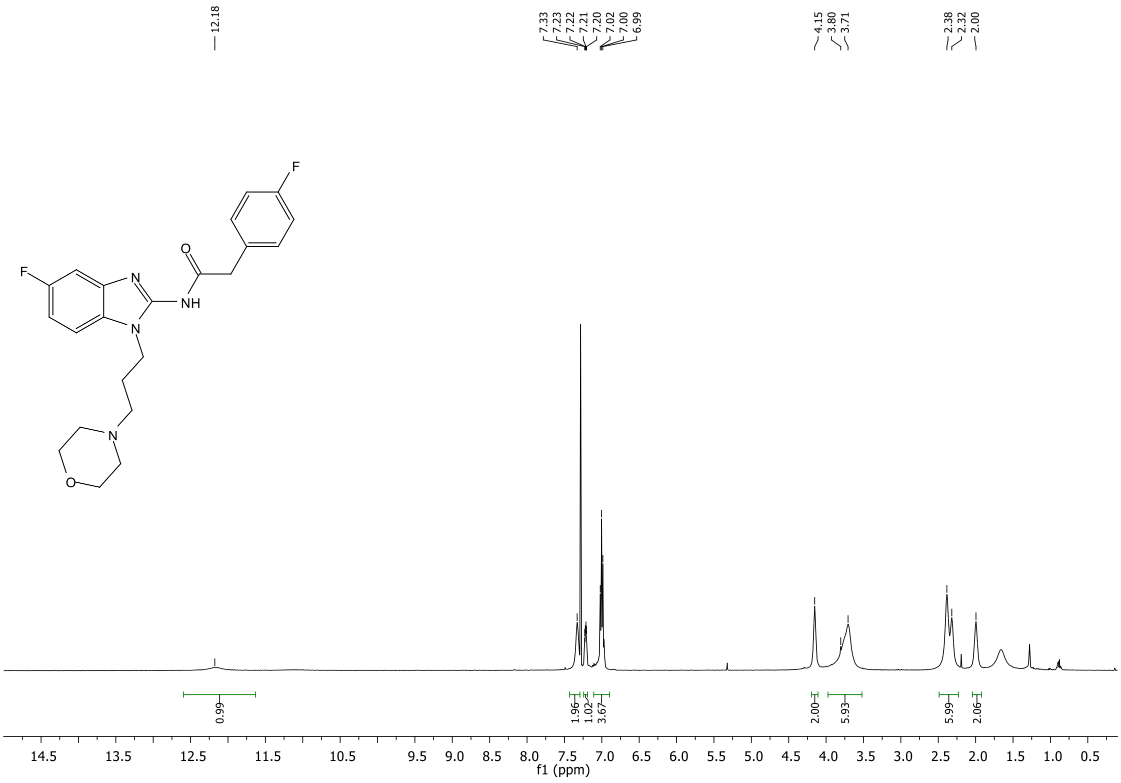

^{1}H NMR of 27 (500 MHz, $CDCl_3$)

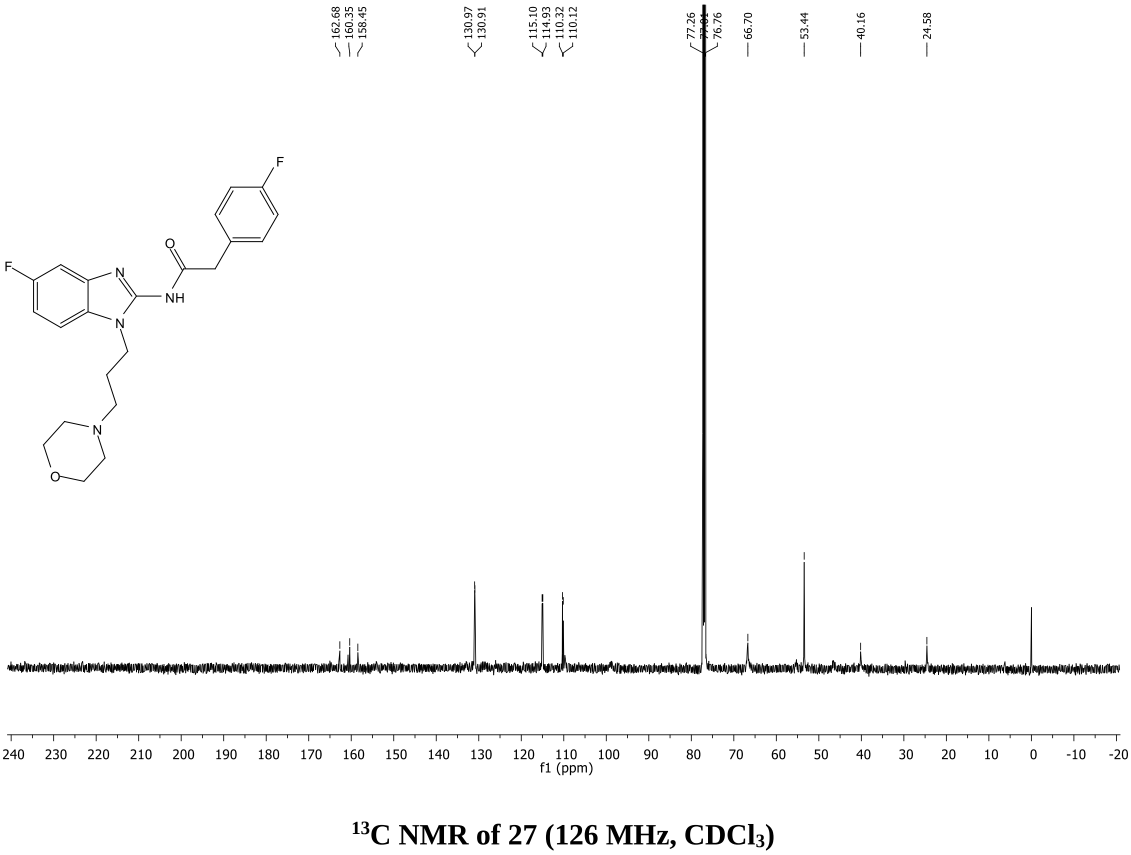

^{13}C NMR of 27 (126 MHz, $CDCl_3$)

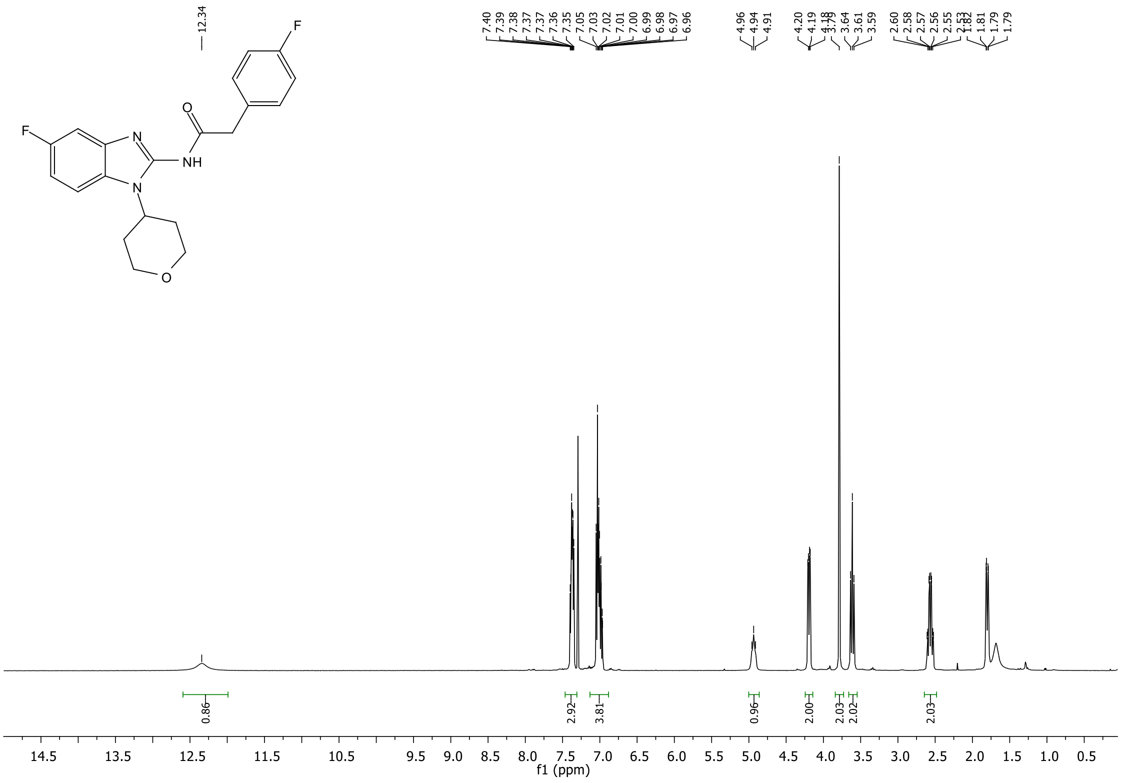

^{1}H NMR of 28 (500 MHz, $CDCl_3$)

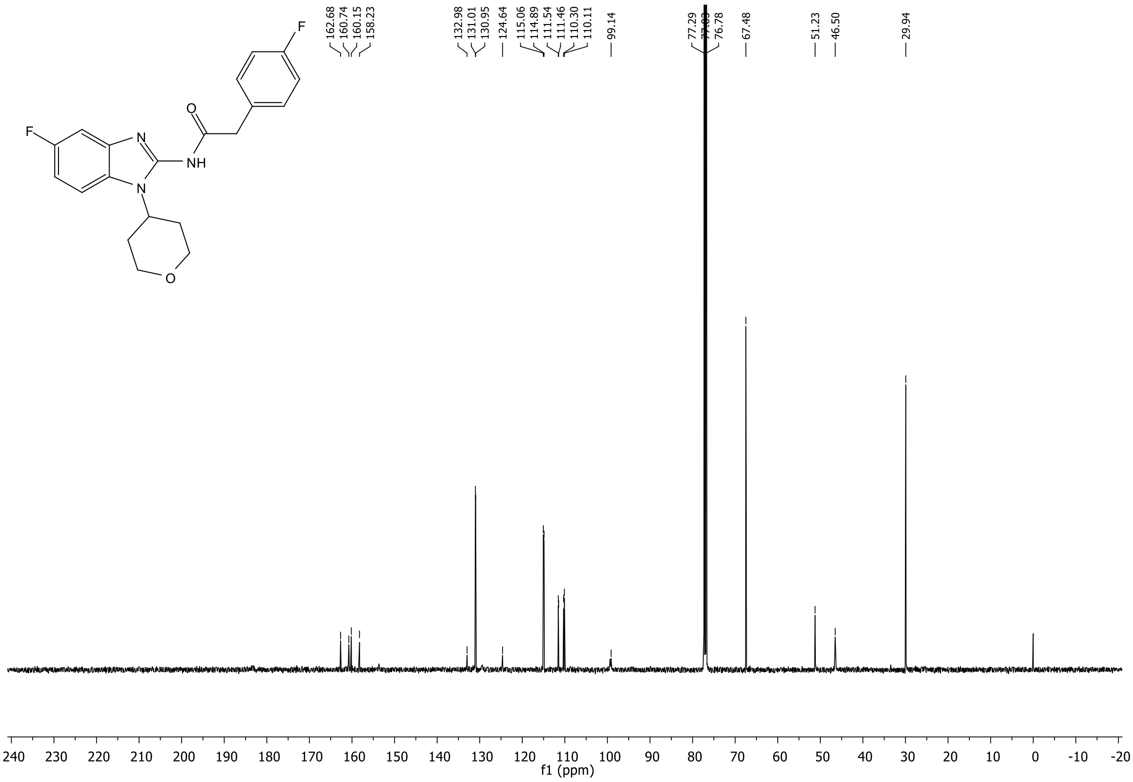

^{13}C NMR of 28 (126 MHz, $CDCl_3$)

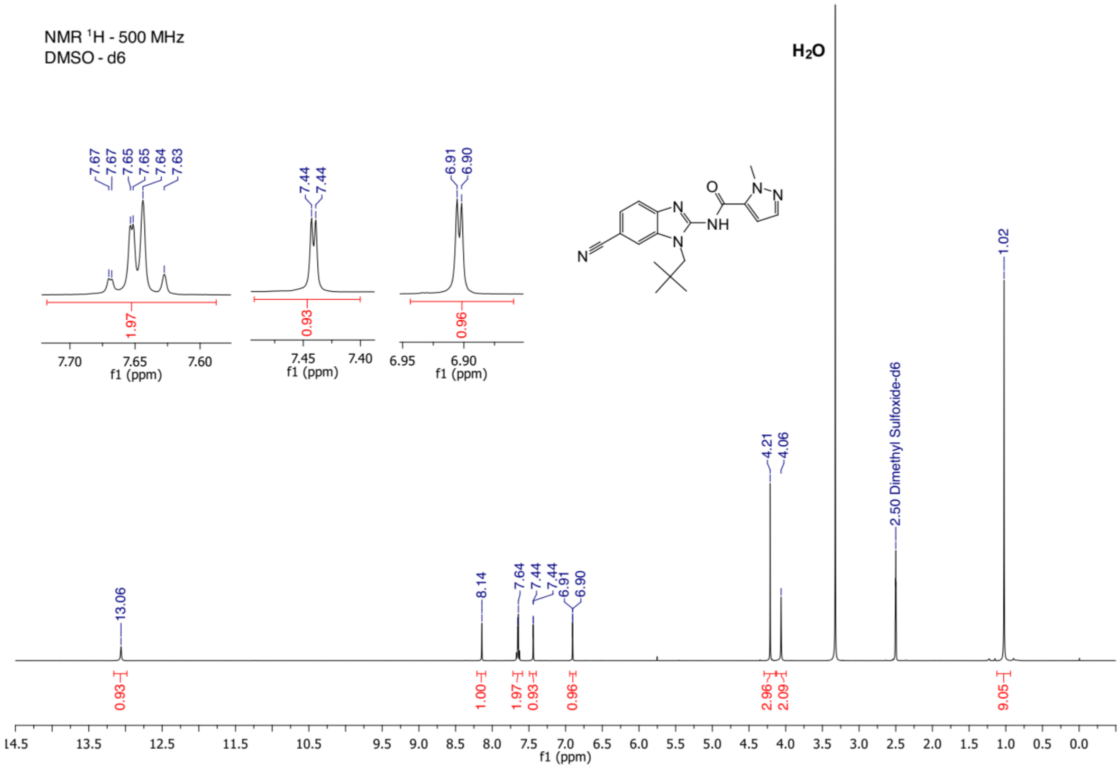

^{1}H NMR of 29 (500 MHz, DMSO)

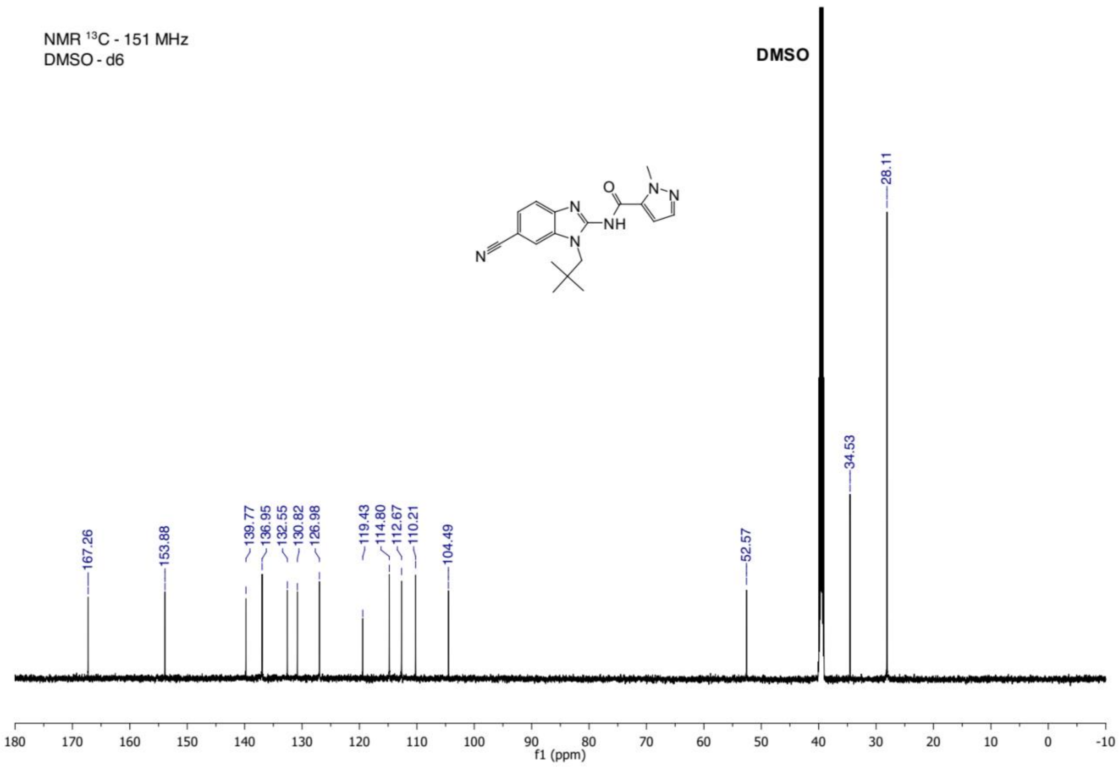

^{13}C NMR of 29 (126 MHz, DMSO)

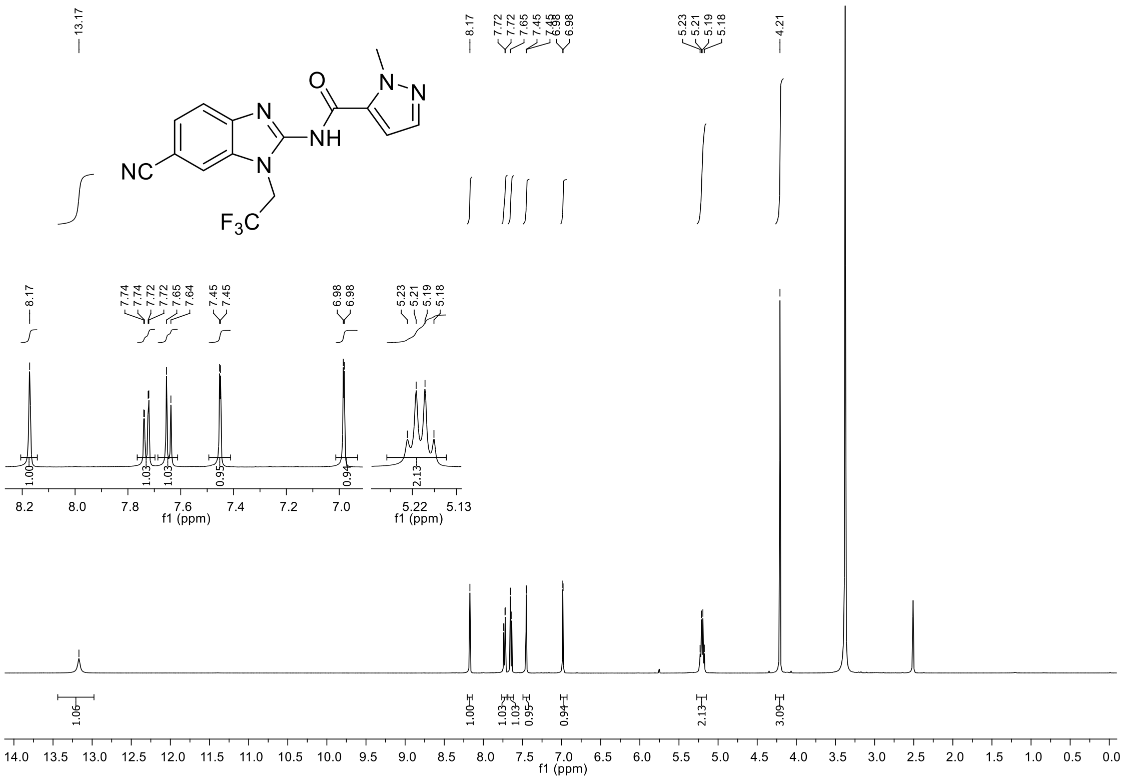

^{1}H NMR of 30 (500 MHz, DMSO)

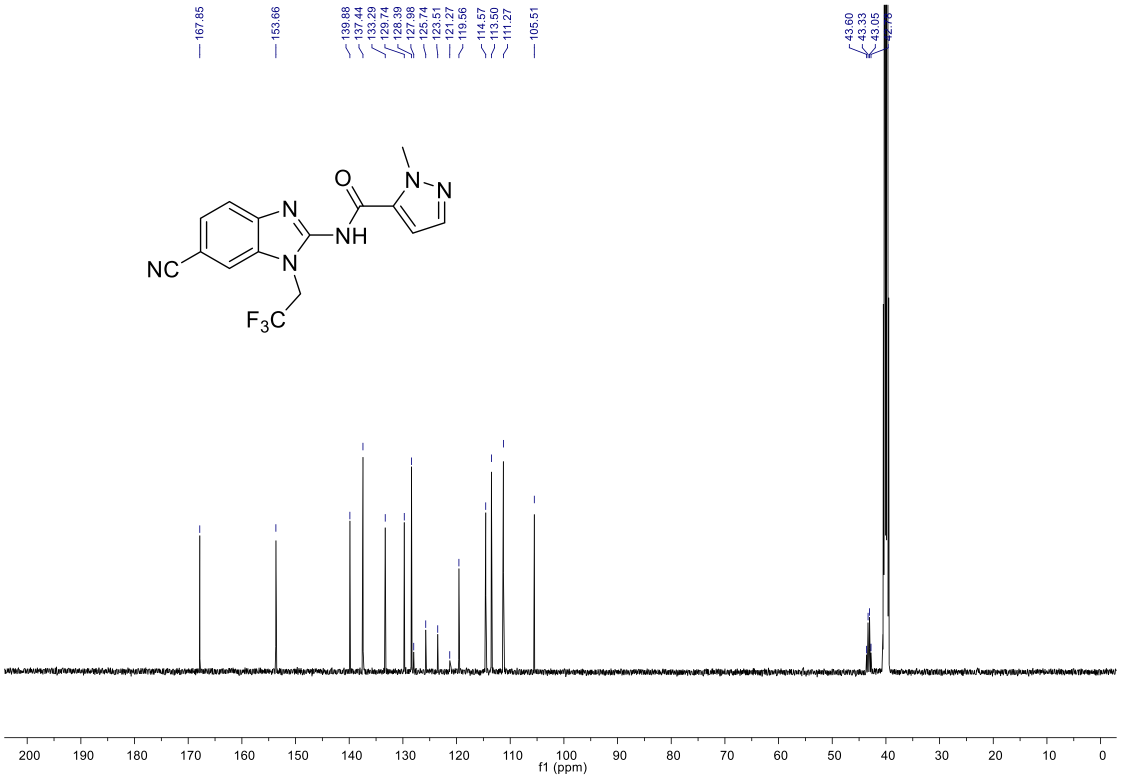

^{13}C NMR of 30 (126 MHz, DMSO)

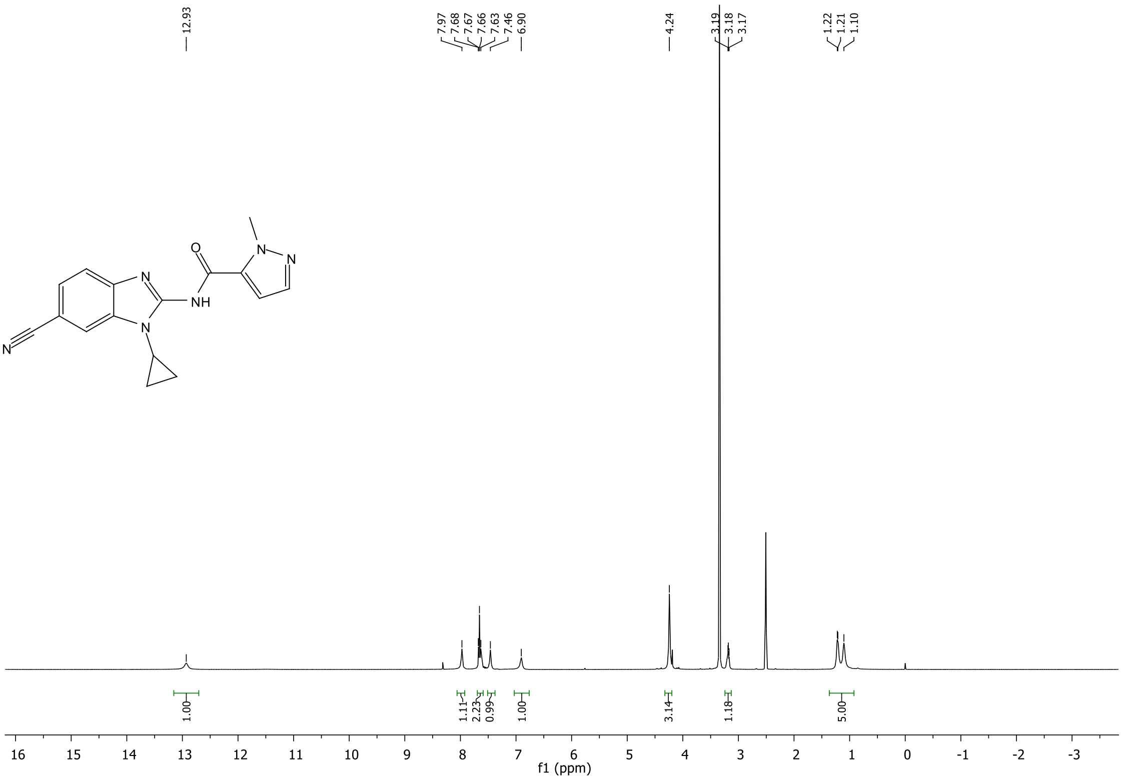

^{1}H NMR of 31 (400 MHz, DMSO)

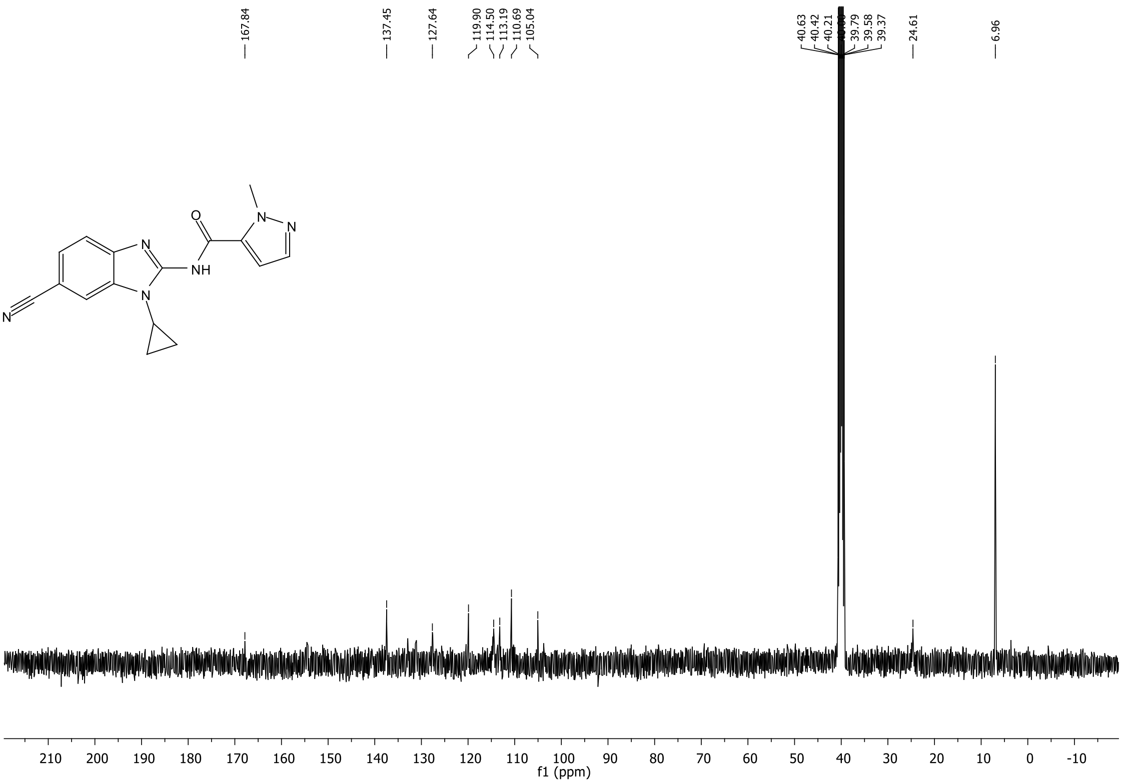

^{13}C NMR of 31 (101 MHz, DMSO)

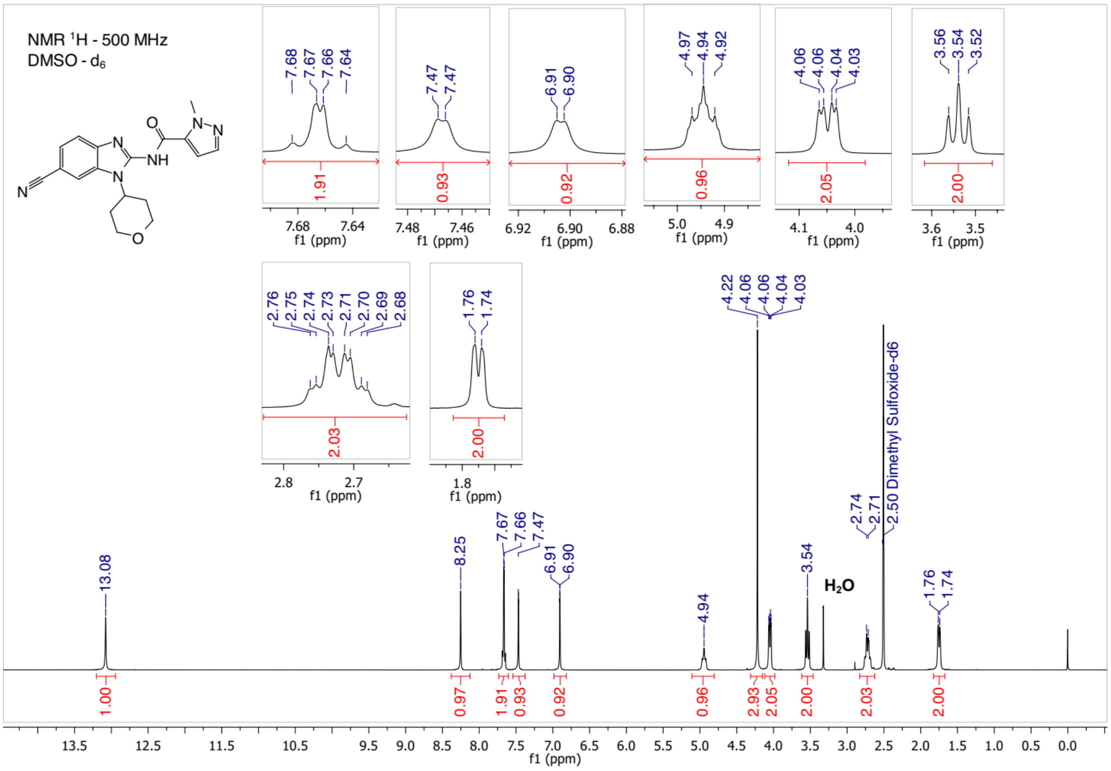

^{1}H NMR of 32 (500 MHz, DMSO)

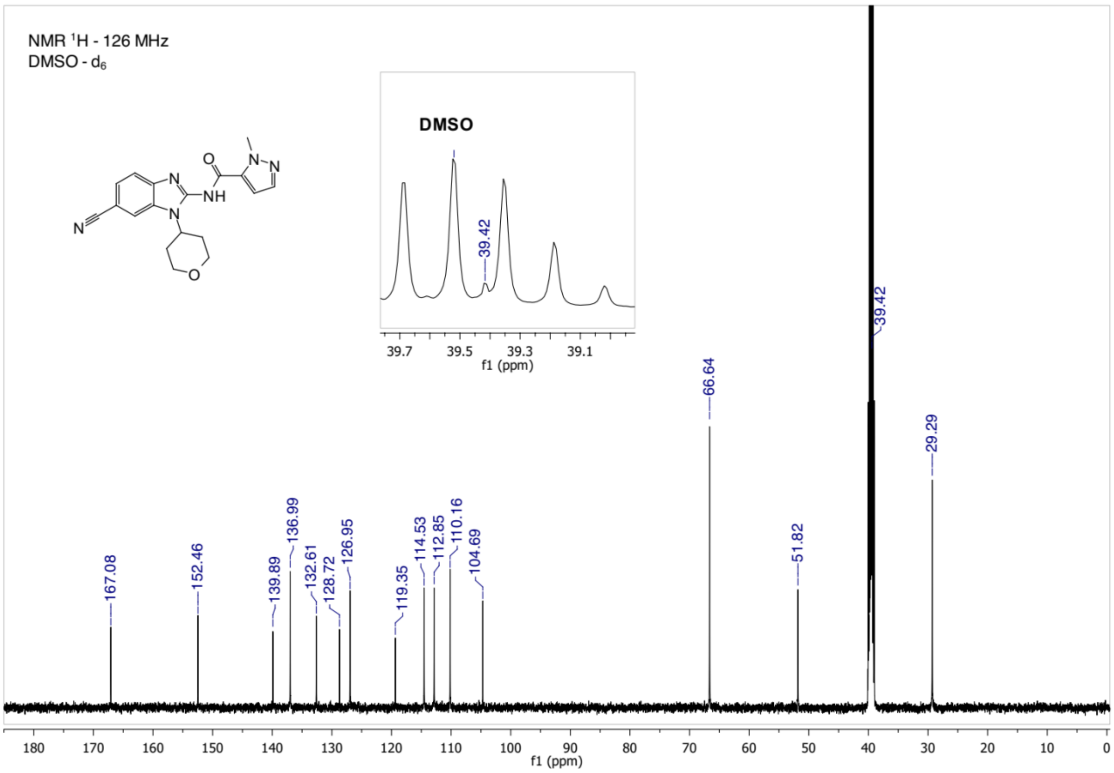

^{13}C NMR of 32 (126 MHz, DMSO)

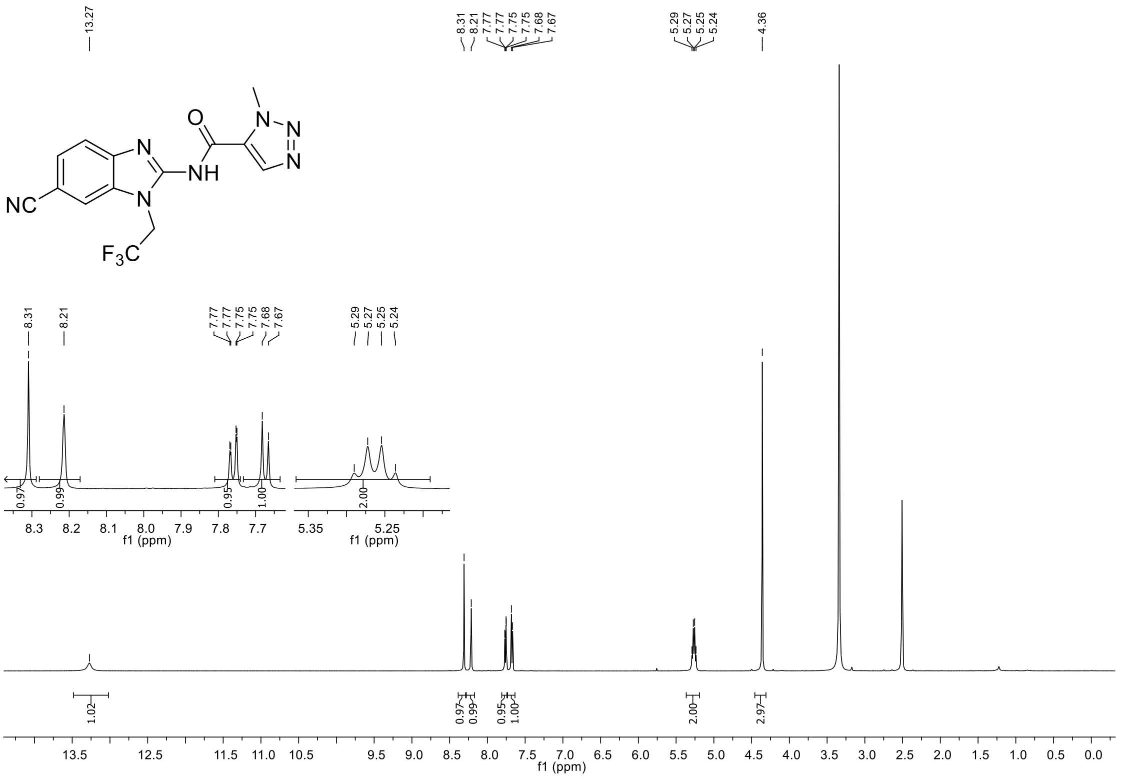

^{1}H NMR of 33 (500 MHz, DMSO)

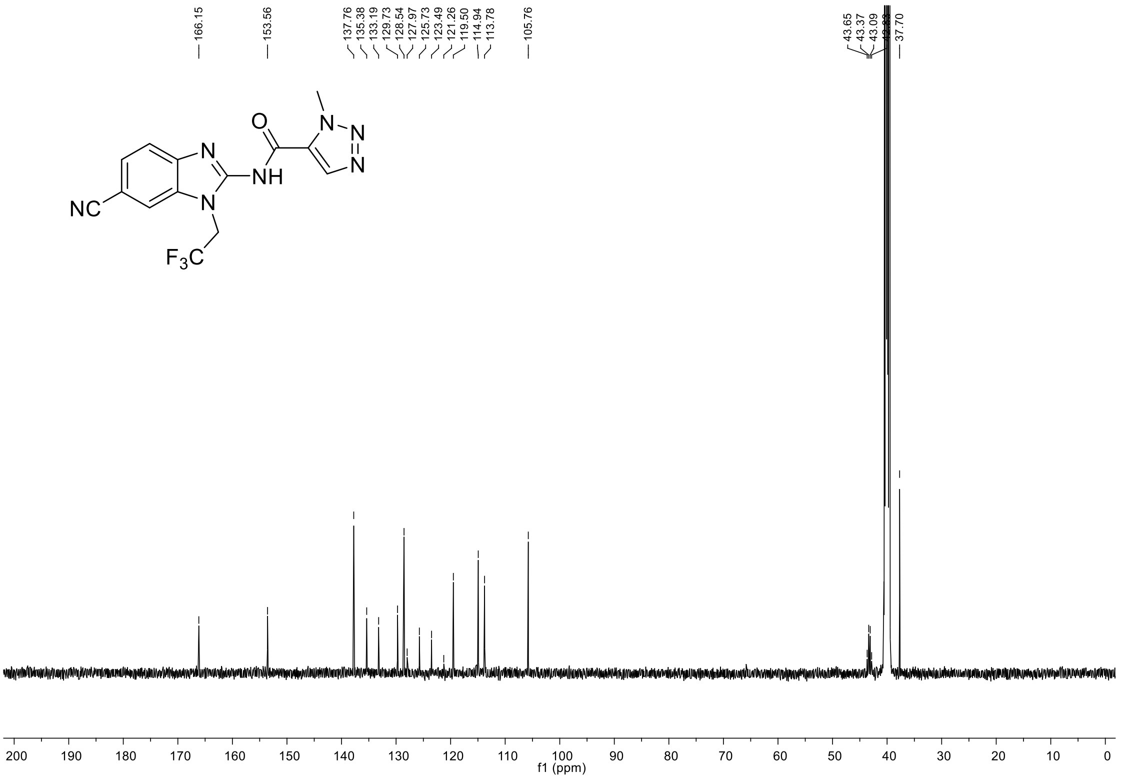

^{13}C NMR of 33 (126 MHz, DMSO)

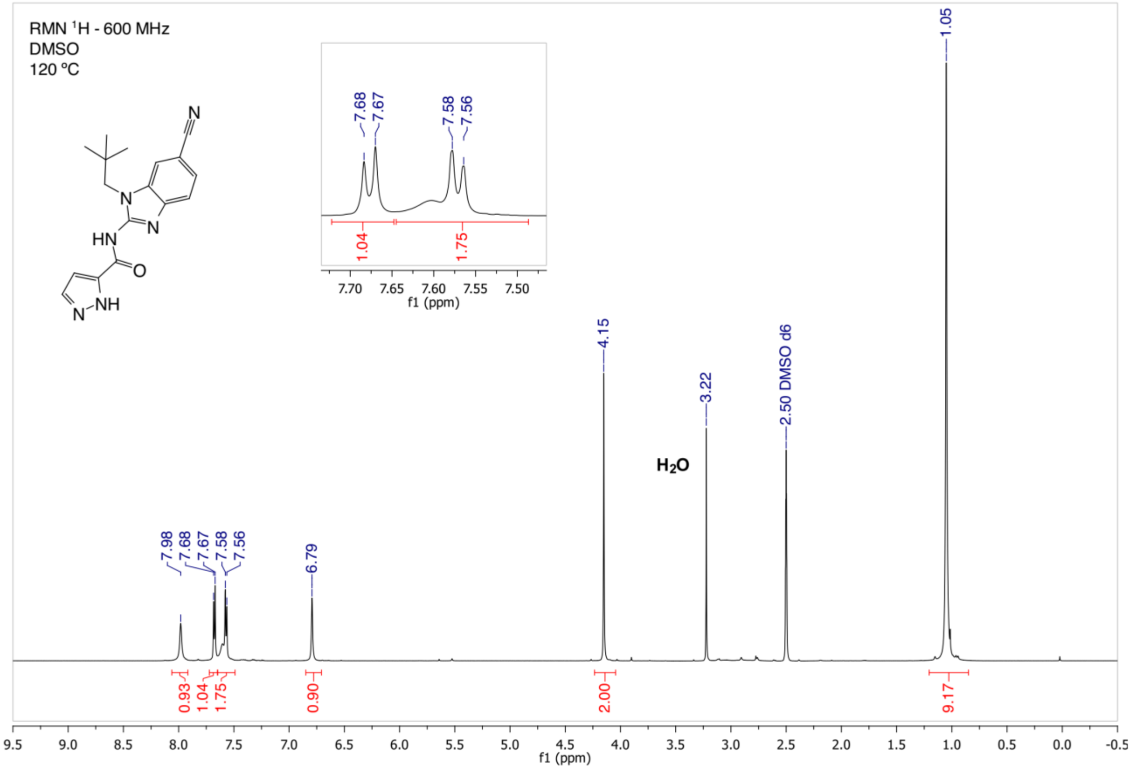

^{1}H NMR of 34 (600 MHz, 120°C, DMSO)

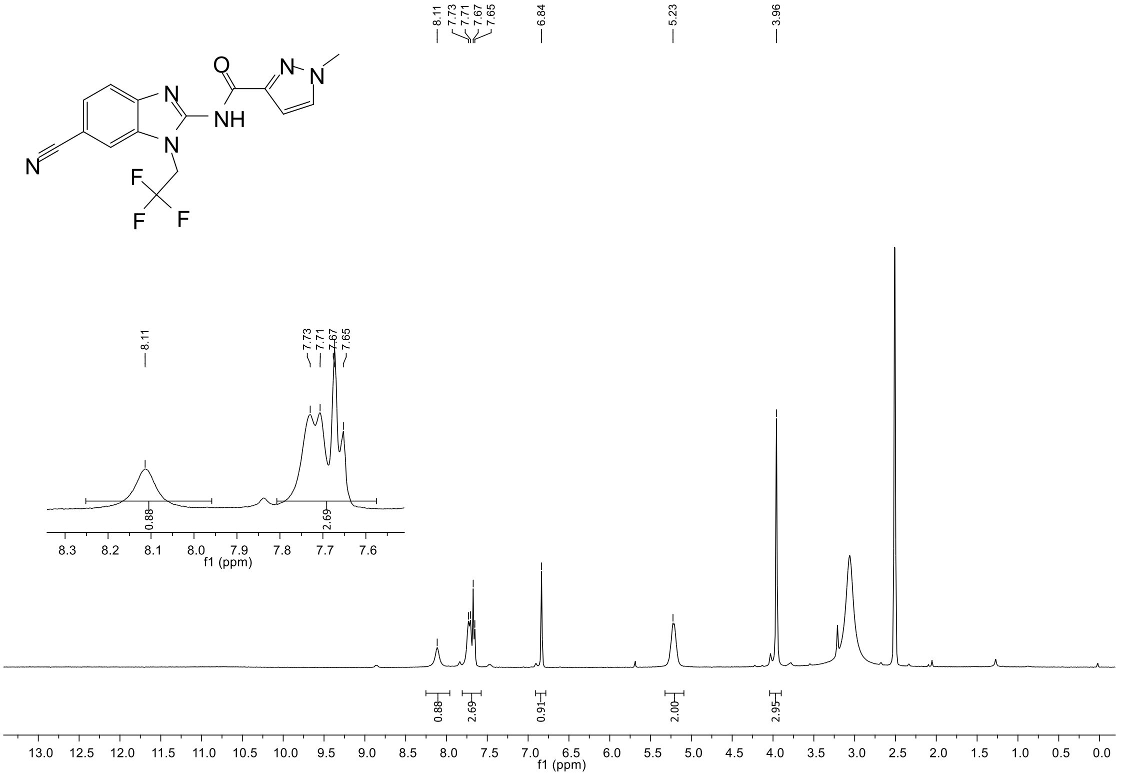

^{1}H NMR of 35 (400 MHz, DMSO)

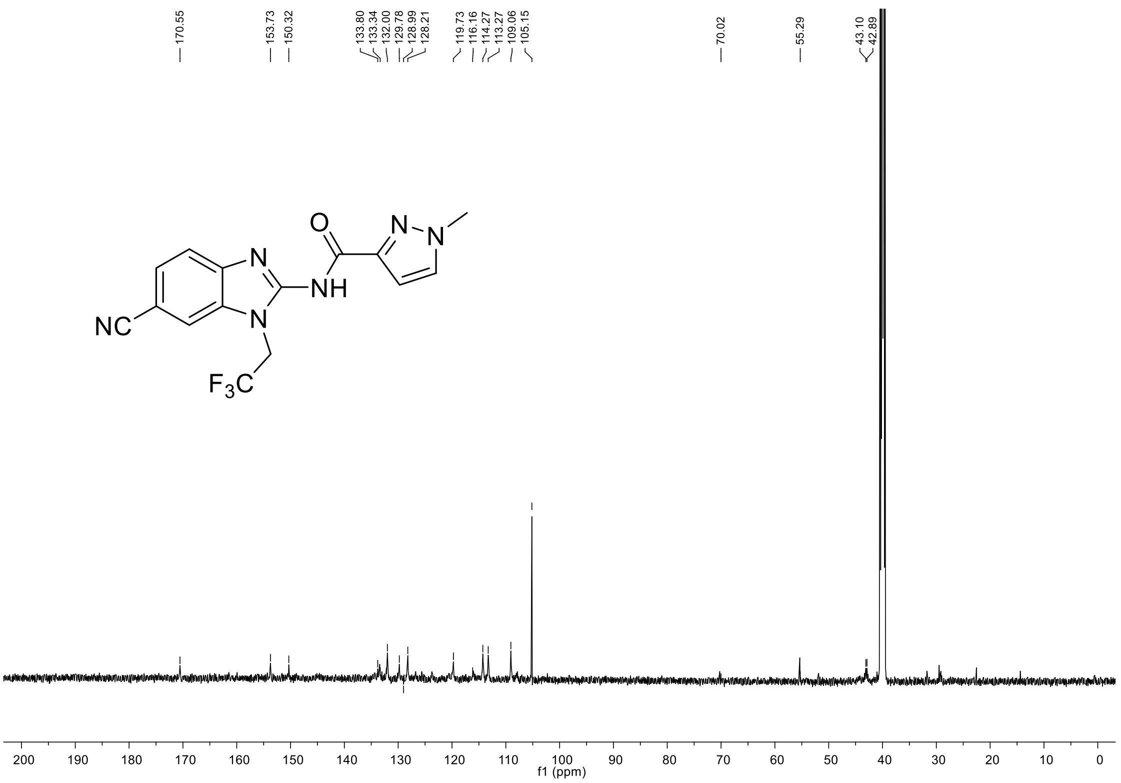

^{13}C NMR of 35 (151 MHz, DMSO)

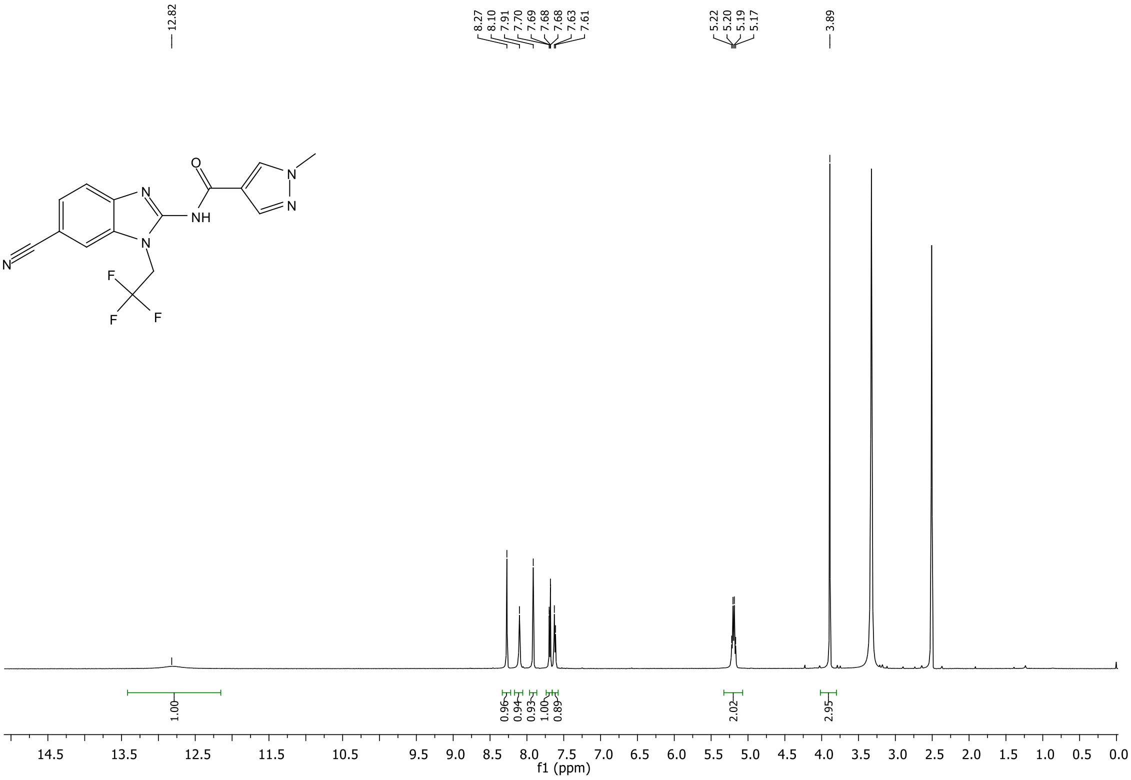

^{1}H NMR of 36 (500 MHz, DMSO)

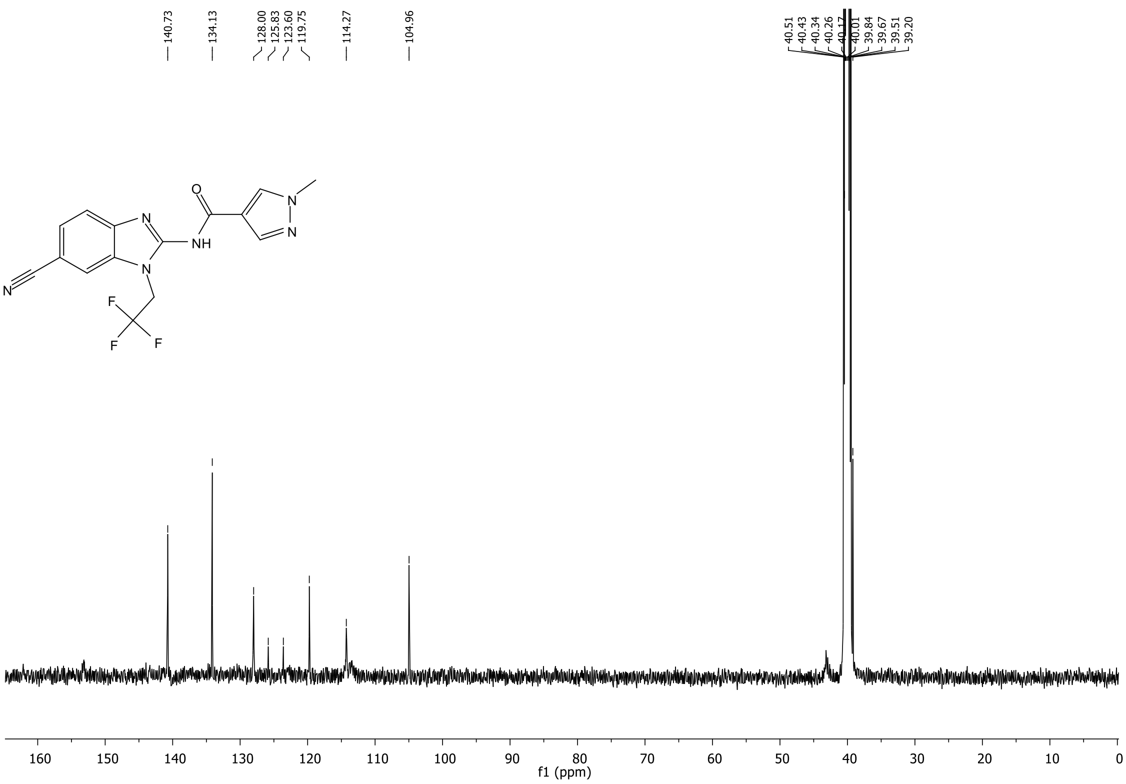

^{13}C NMR of 36 (126 MHz, DMSO)

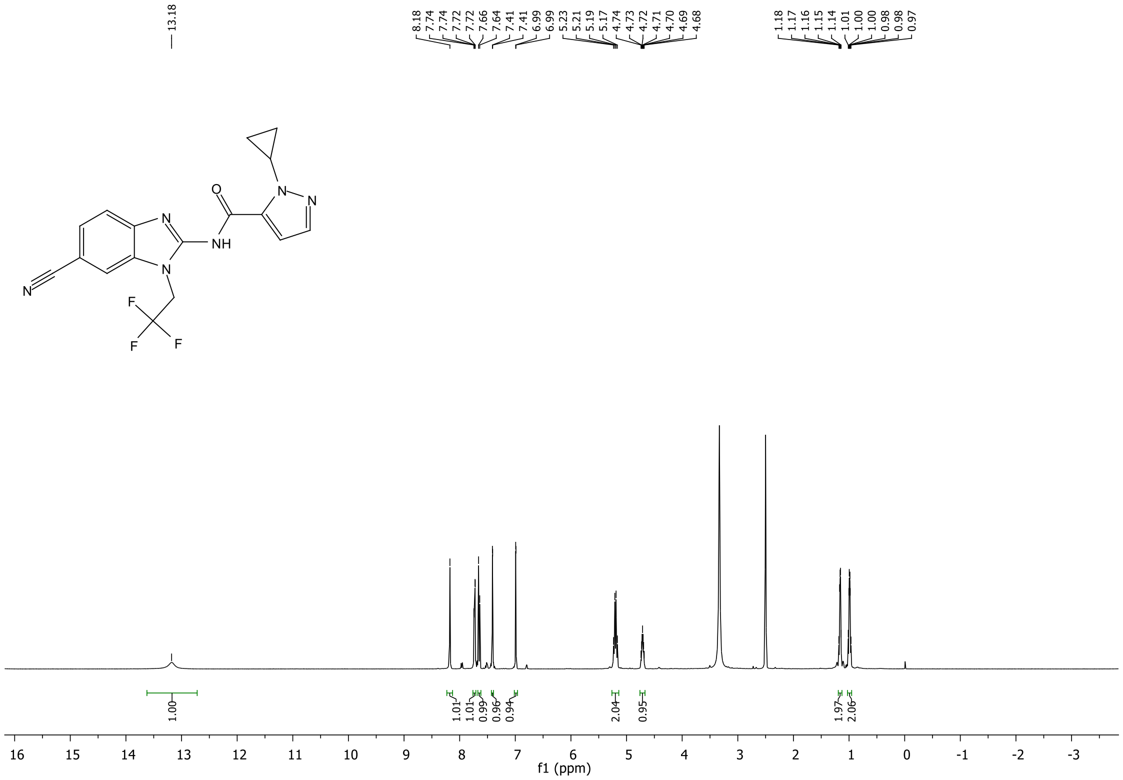

^{1}H NMR of 37 (400 MHz, DMSO)

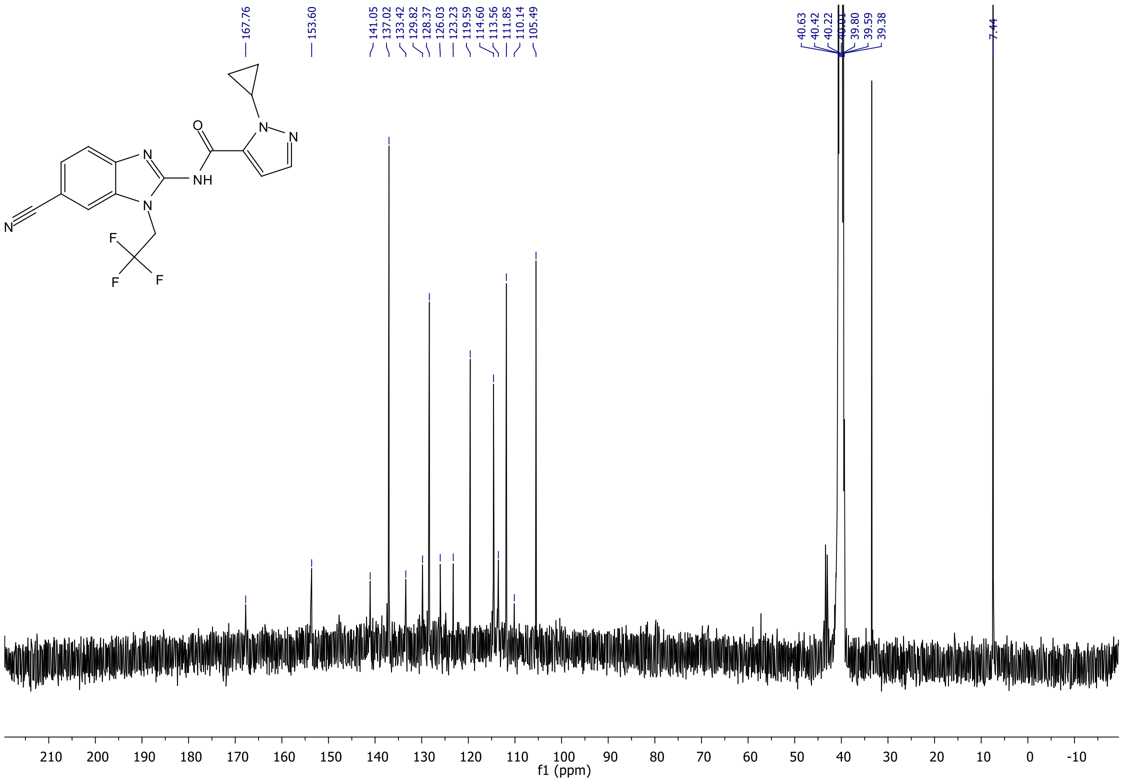

^{13}C NMR of 37 (101 MHz, DMSO)

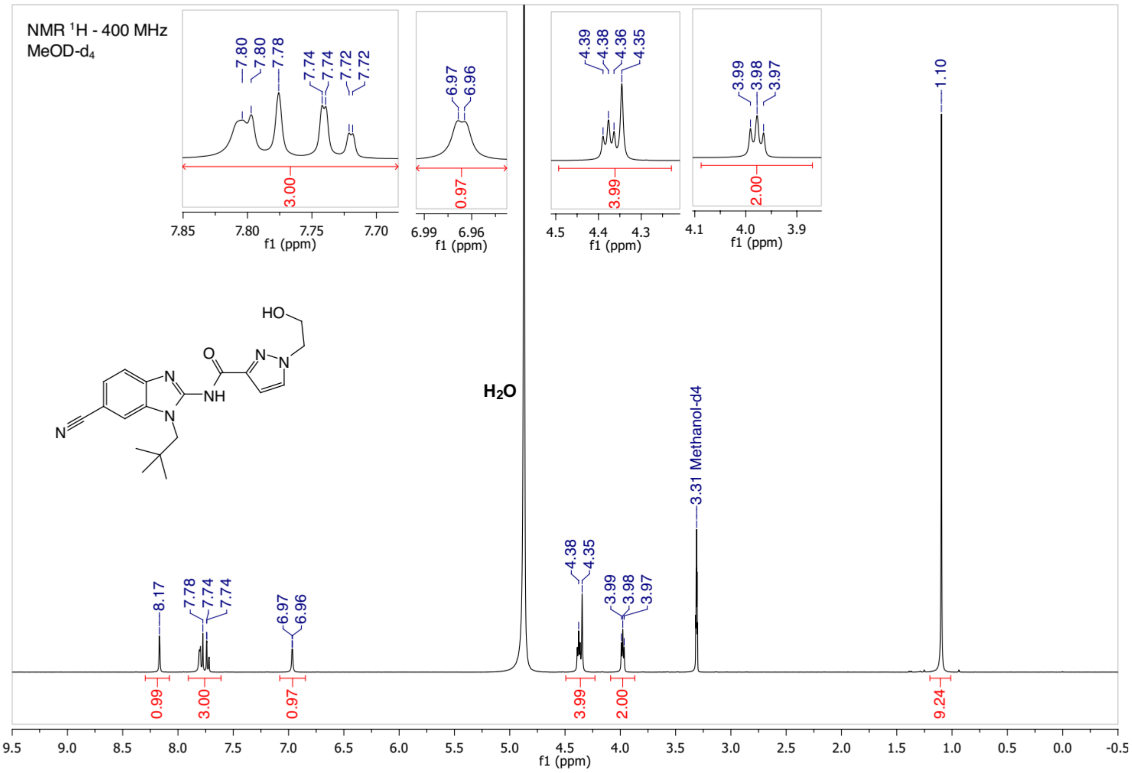

^{1}H NMR of 38 (400 MHz, CD_3OD)

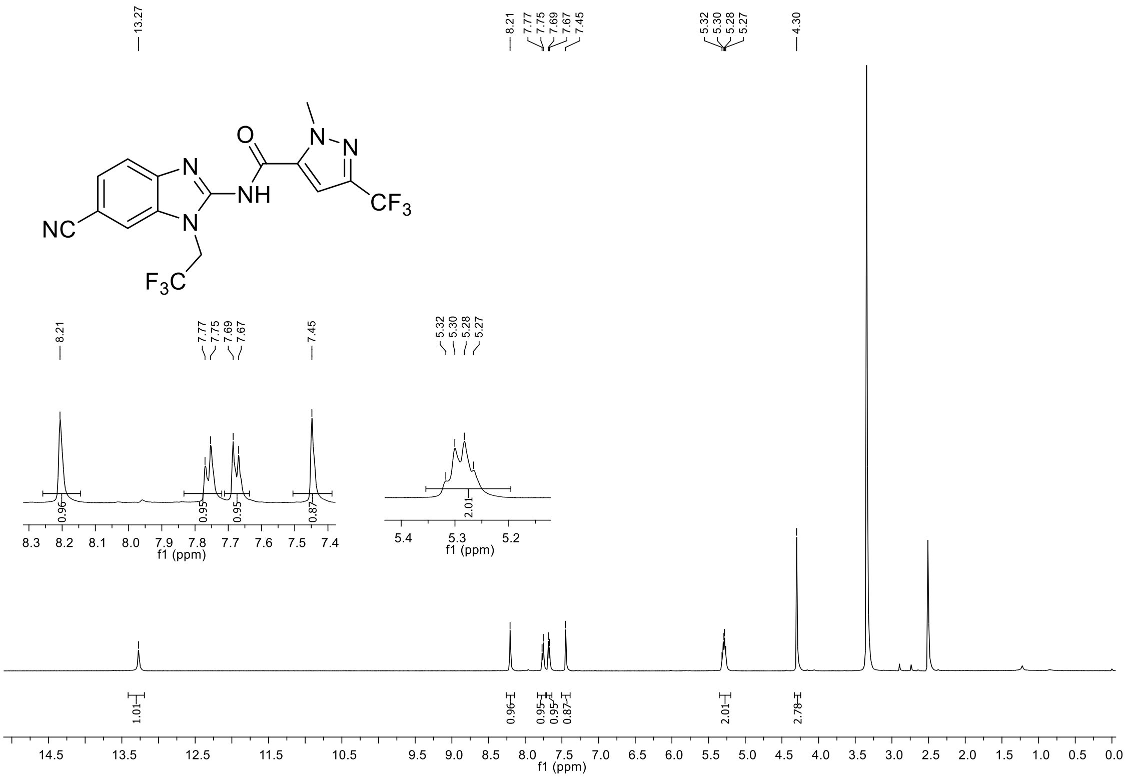

^{1}H NMR of 39 (400 MHz, DMSO)

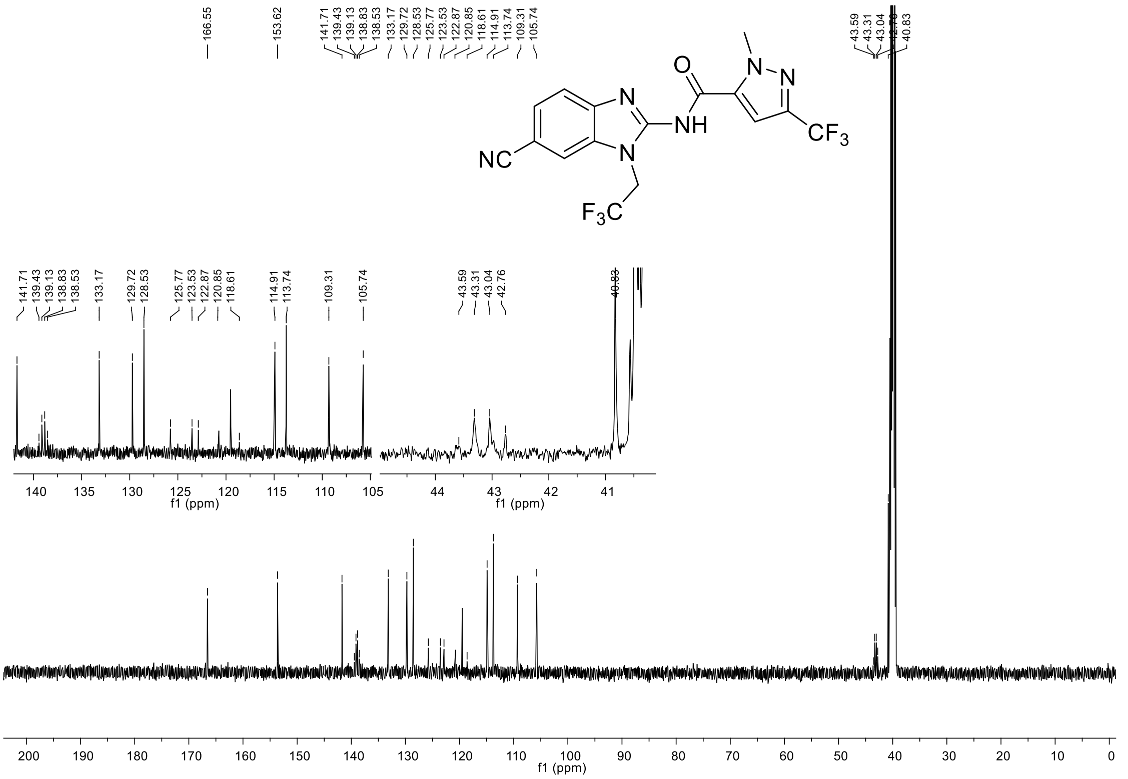

^{13}C NMR of 39 (126 MHz, DMSO)

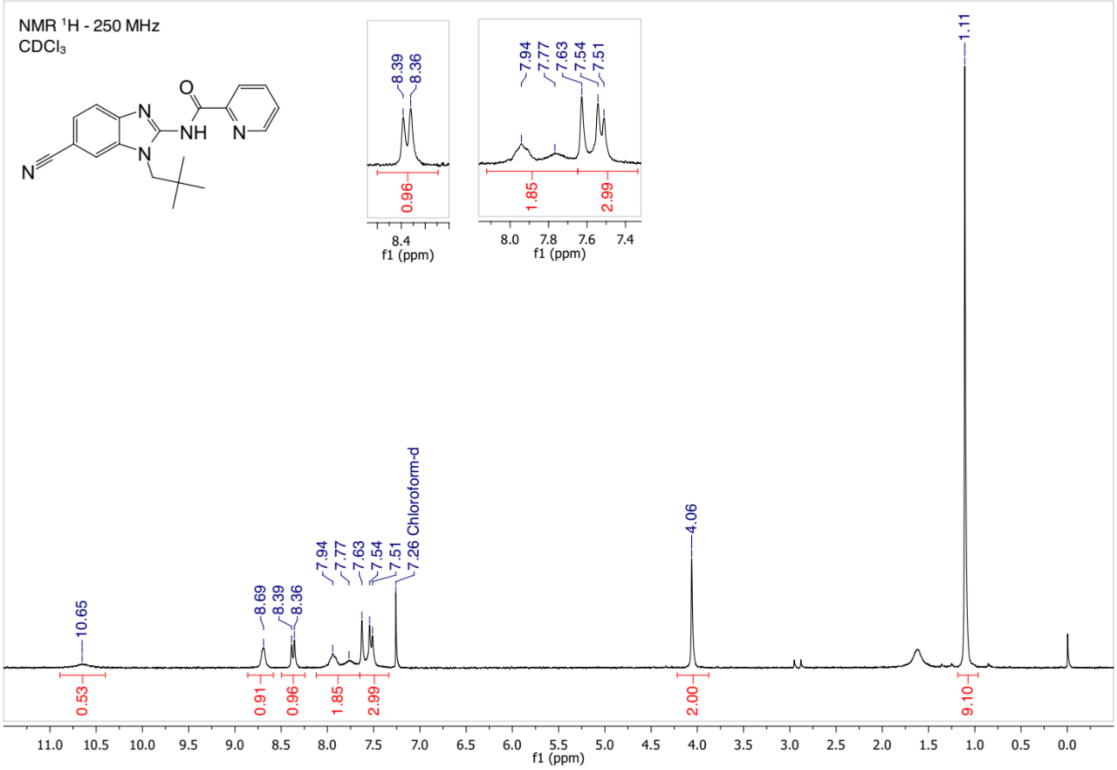

^{1}H NMR of 40 (250MHz, $CDCl_3$)

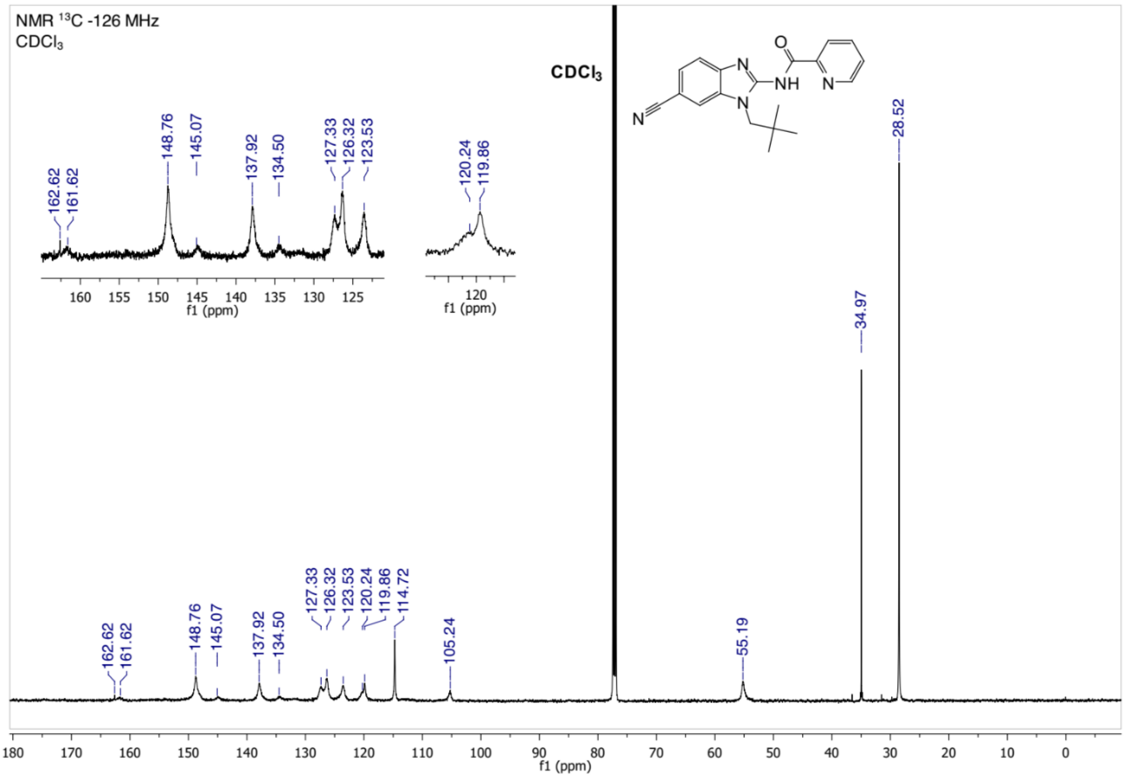

^{13}C NMR of 40 (126 MHz, $CDCl_3$)

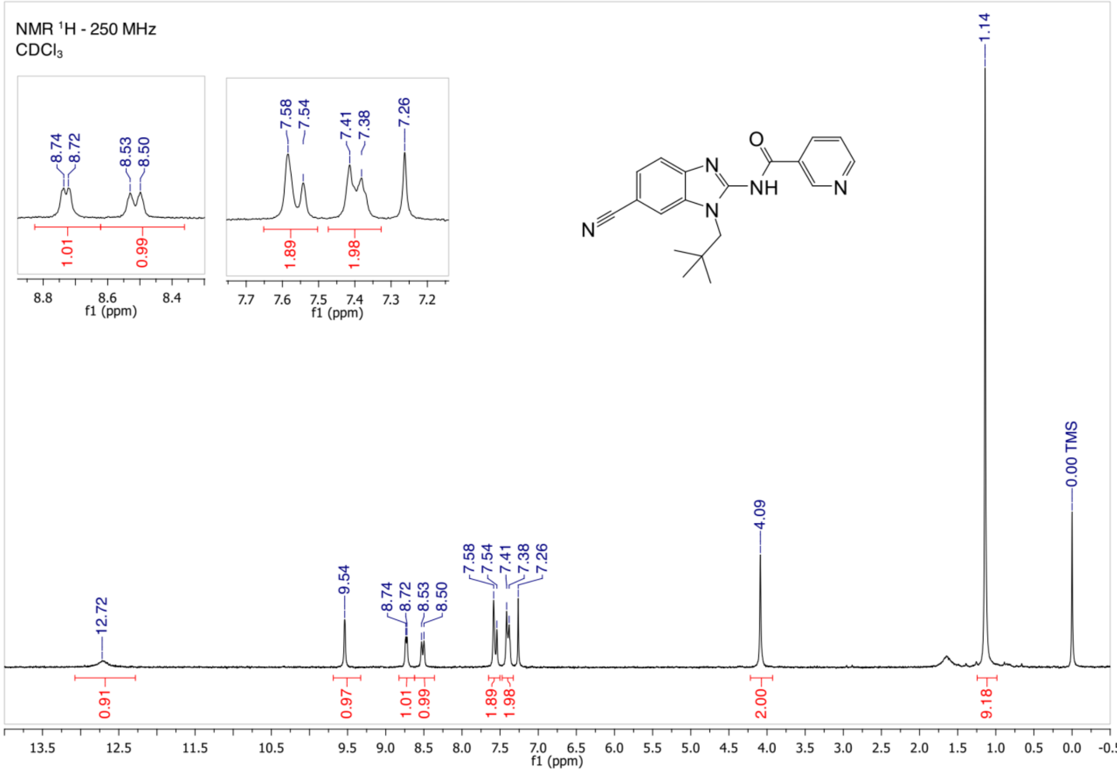

^{1}H NMR of 41 (250MHz, $CDCl_3$)

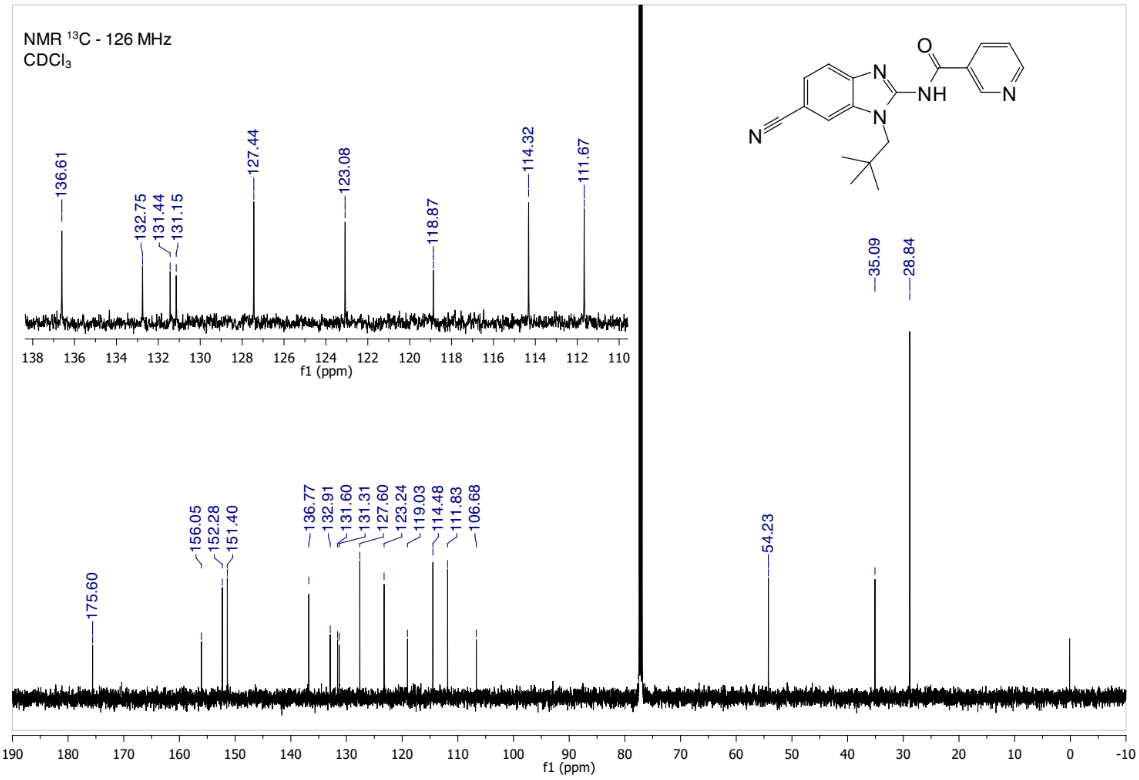

^{13}C NMR of 41 (126 MHz, $CDCl_3$)

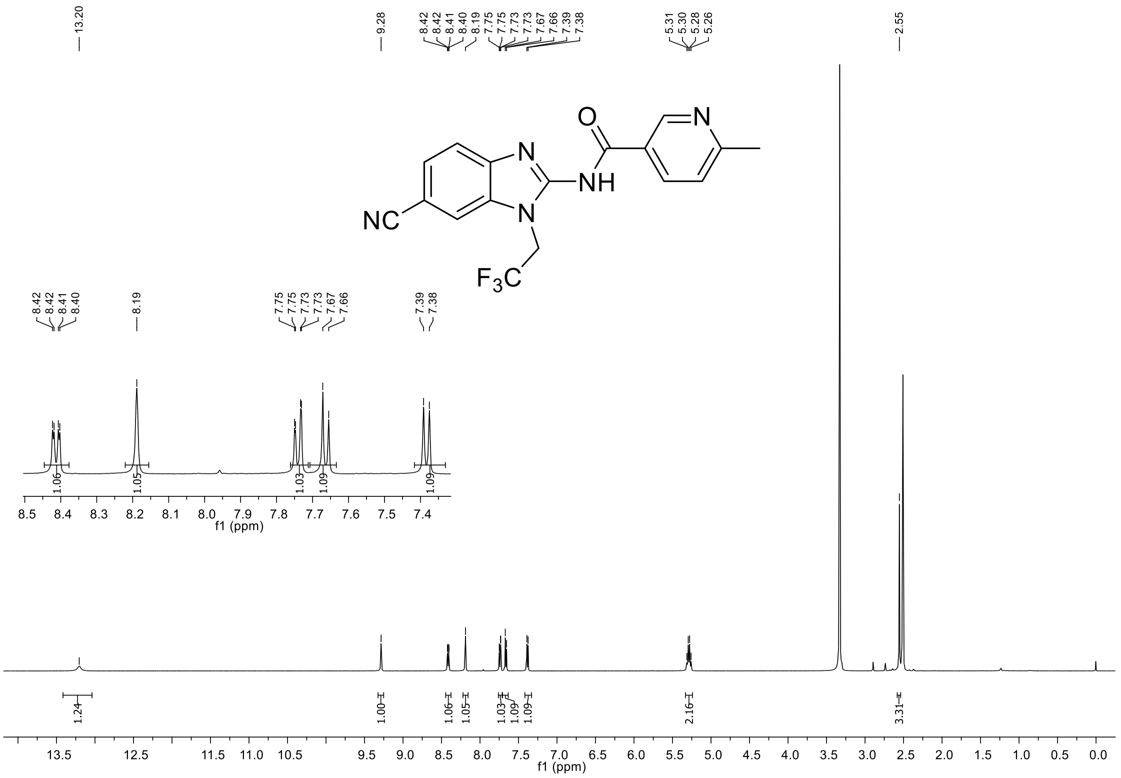

^{1}H NMR of 42 (500 MHz, DMSO)

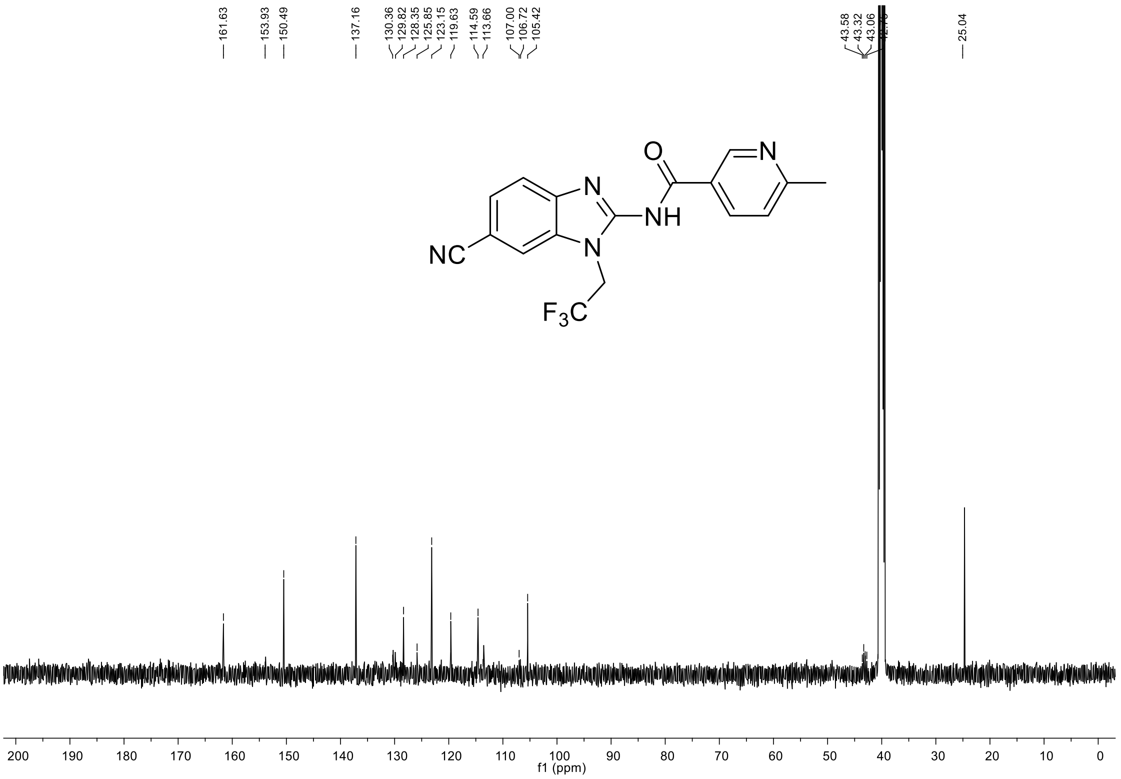

^{13}C NMR of 42 (126 MHz, DMSO)

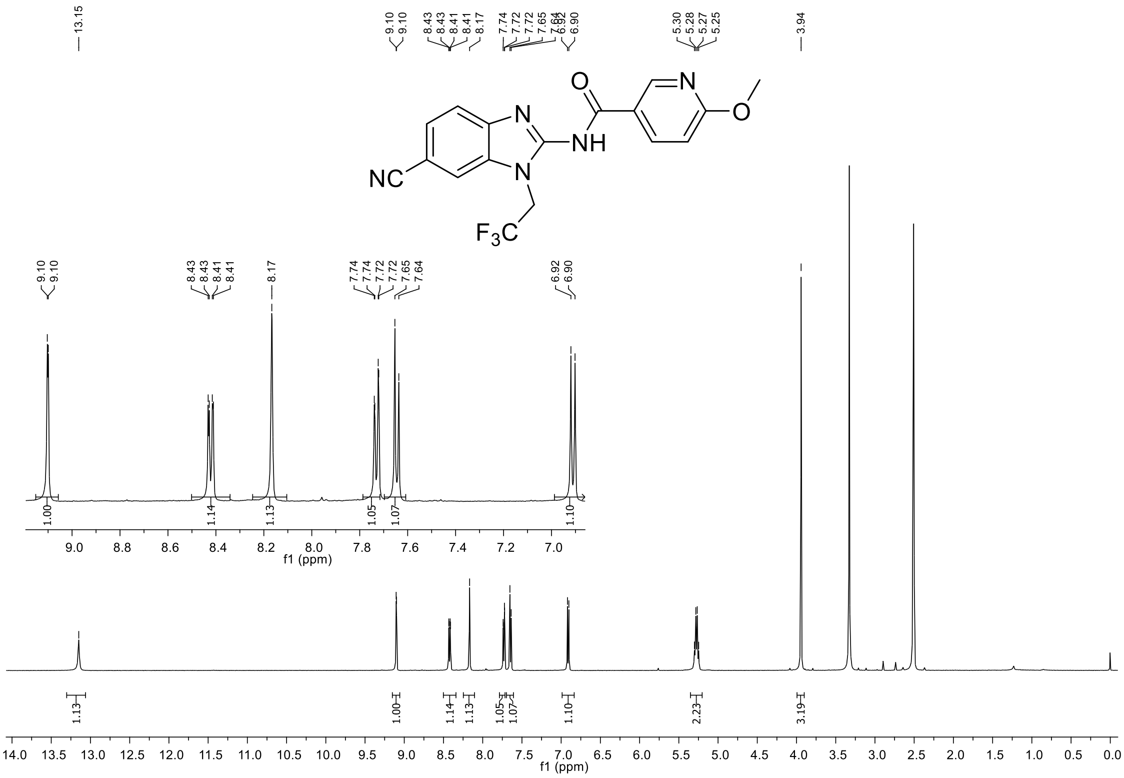

^{1}H NMR of 43 (500 MHz, DMSO)

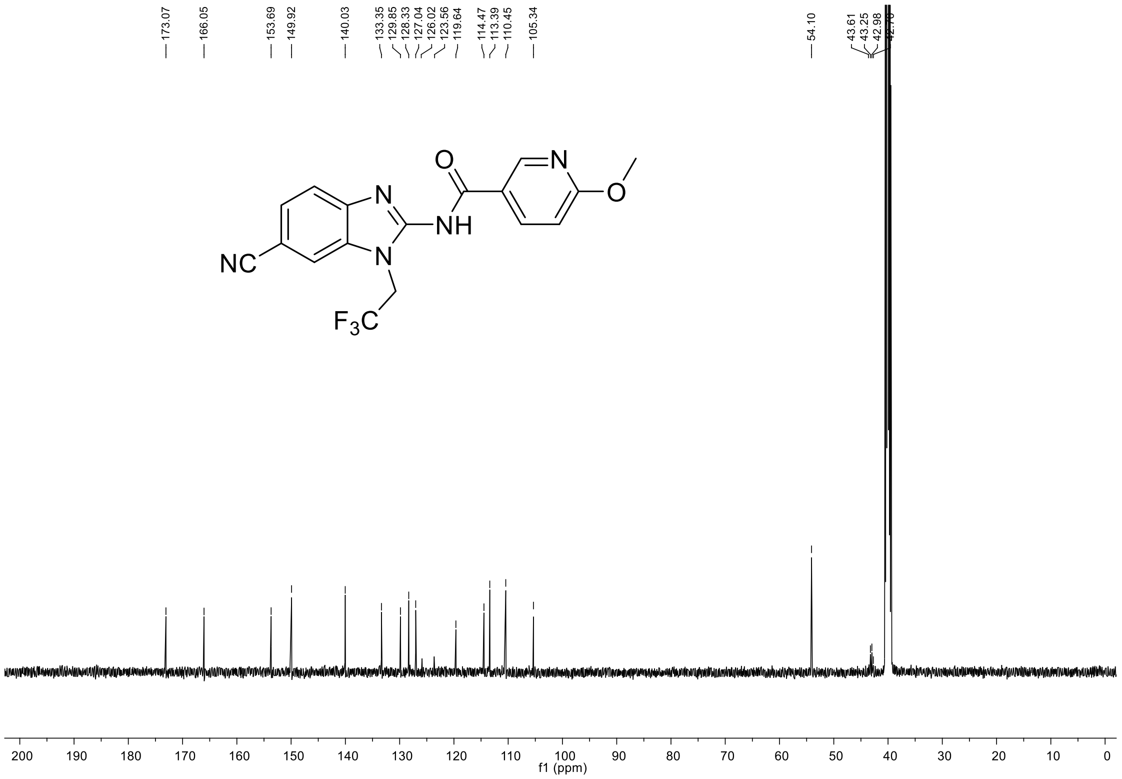

^{13}C NMR of 43 (126 MHz, DMSO)

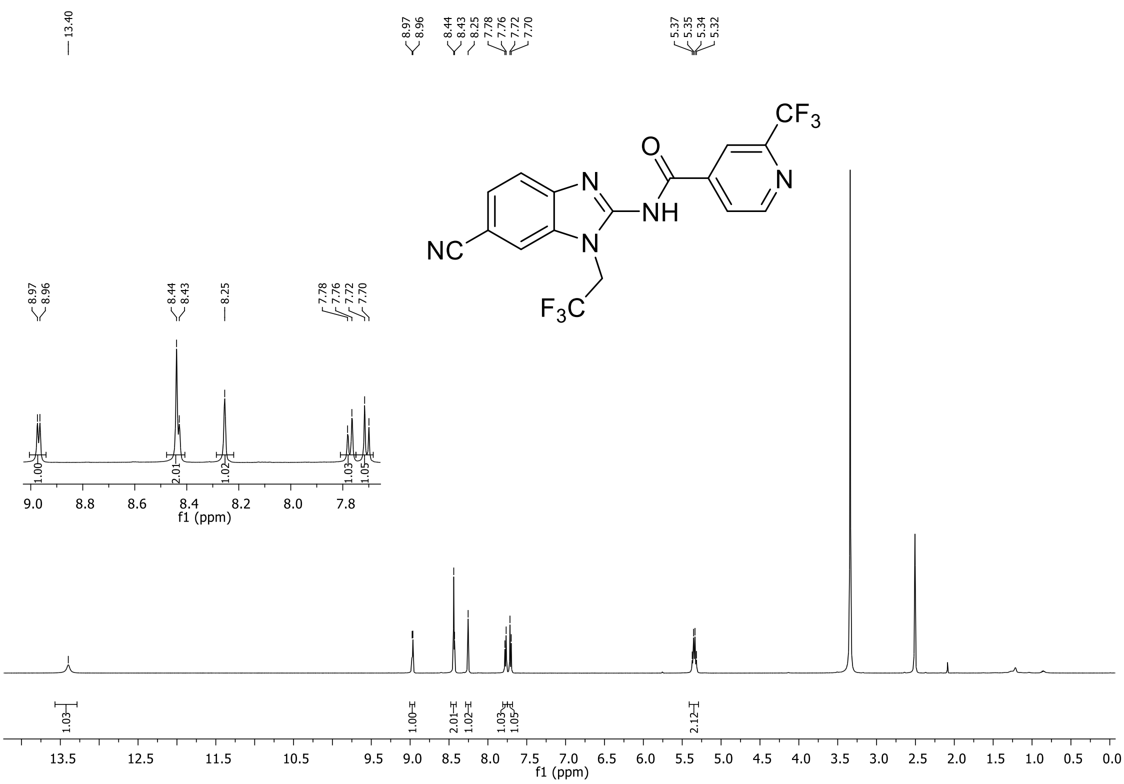

^1H NMR of 44 (500 MHz, DMSO)

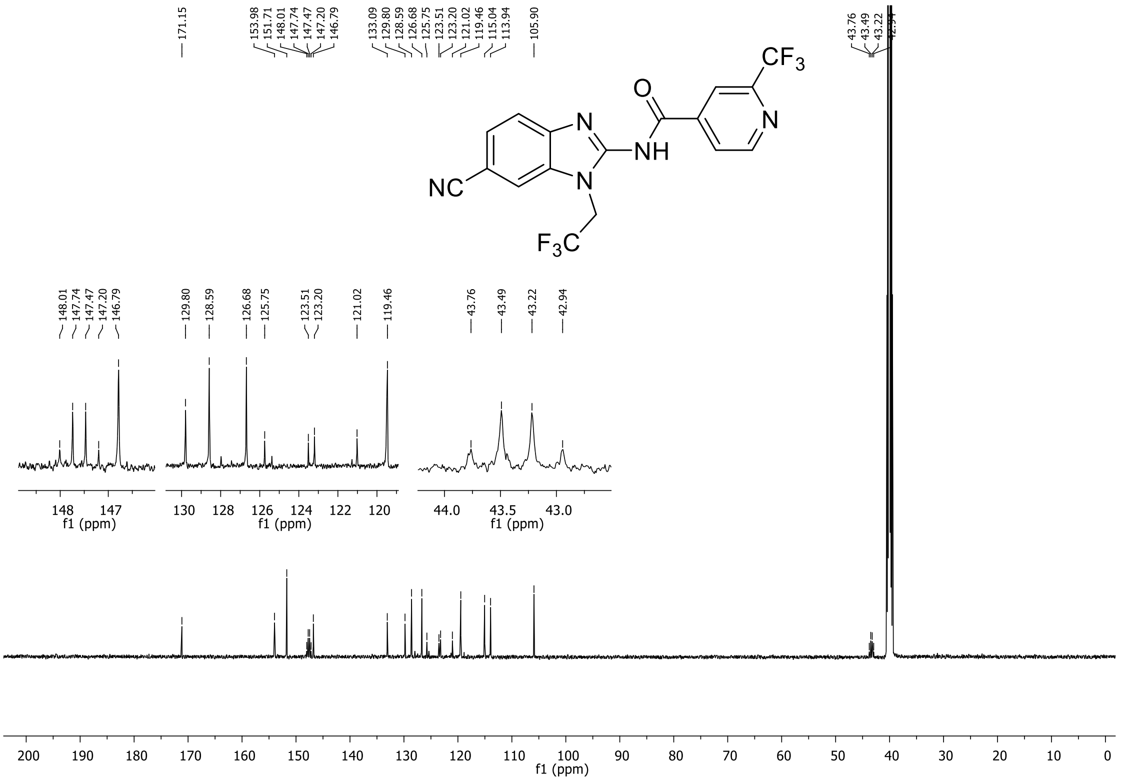

^{13}C NMR of 44 (126 MHz, DMSO)

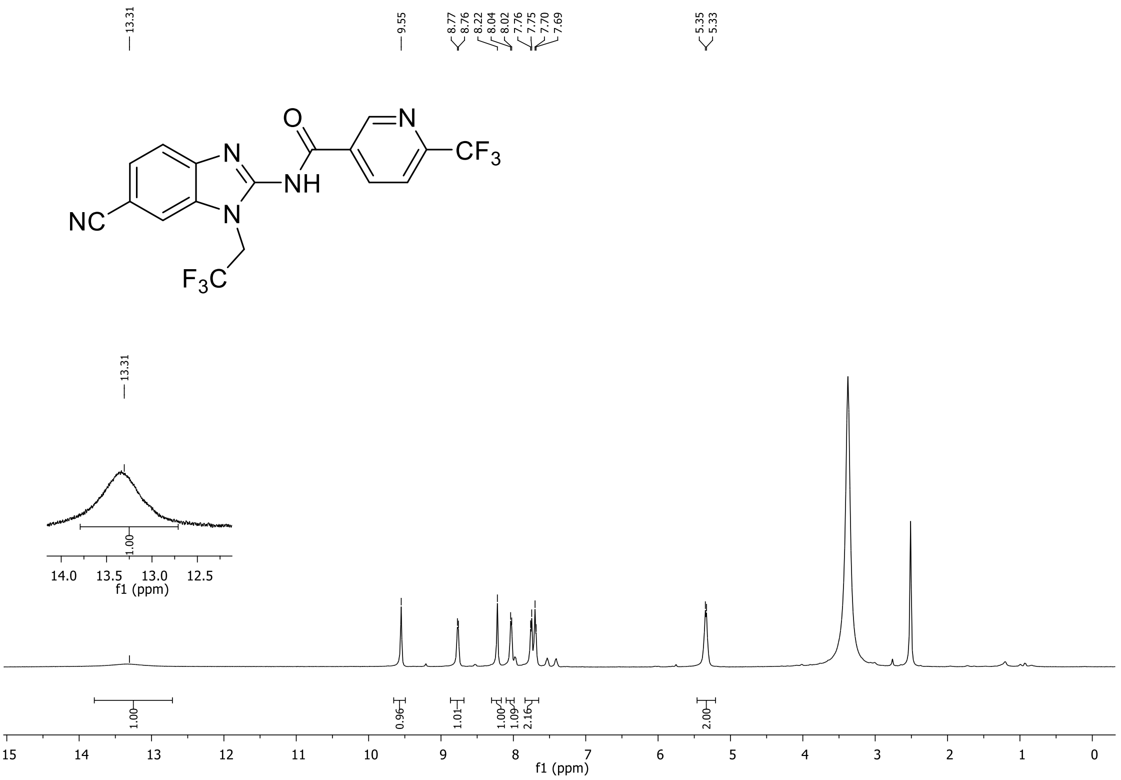

^{1}H NMR of 45 (500 MHz, DMSO)

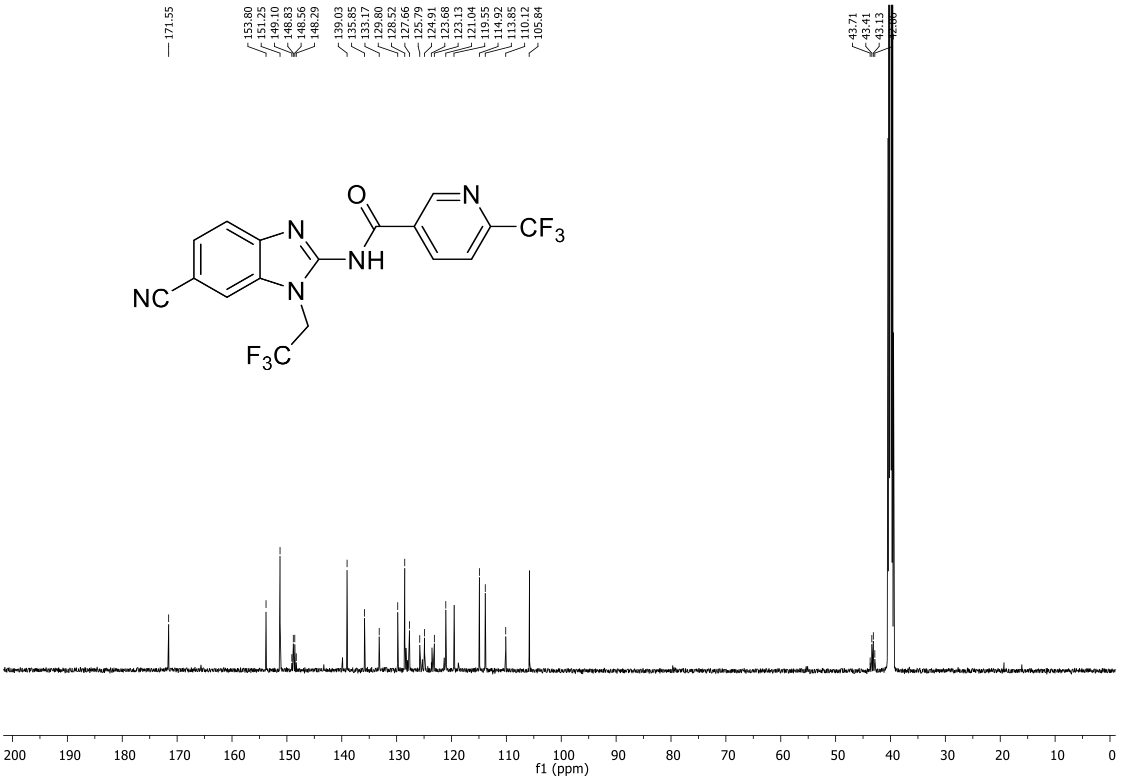

^{13}C NMR of 45 (126 MHz, DMSO)

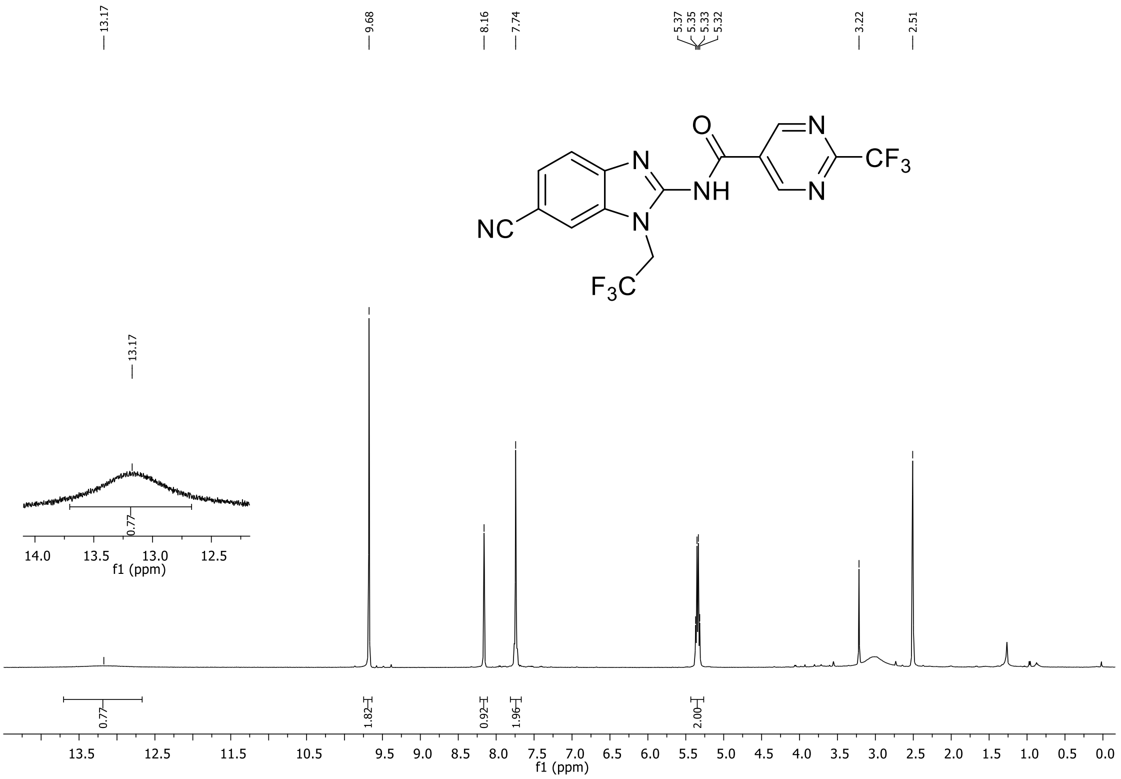

^{1}H NMR of 46 (500 MHz, DMSO)

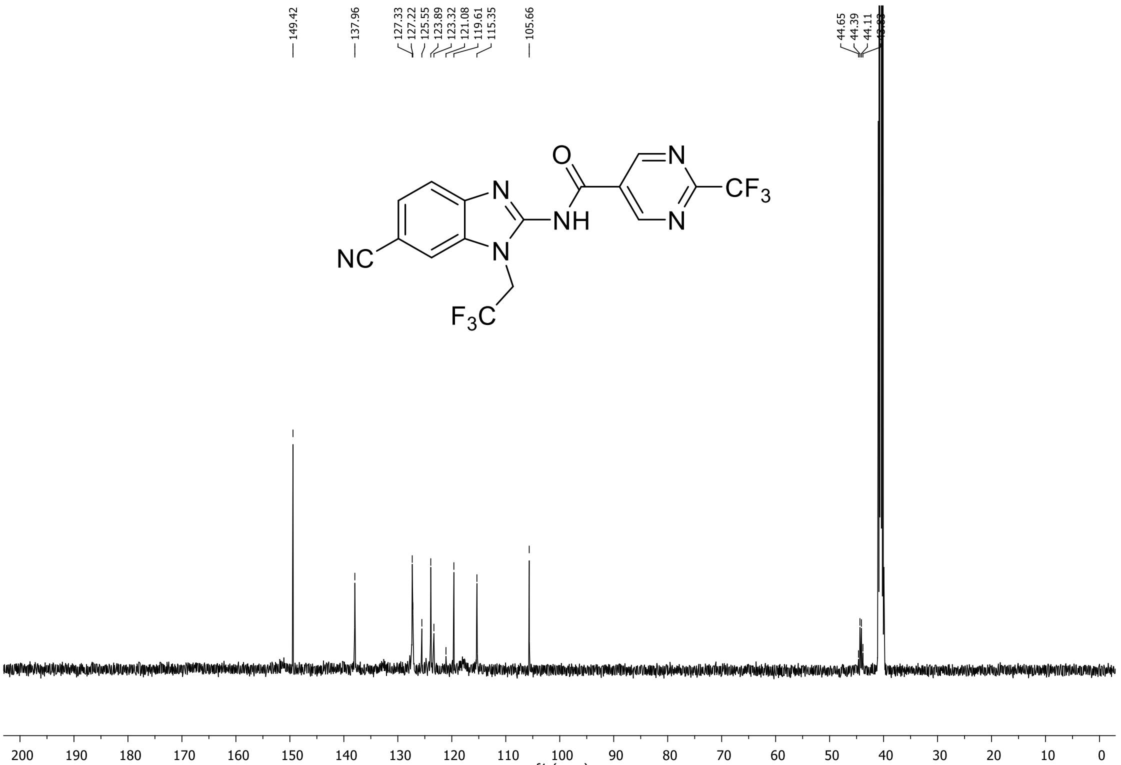

^{13}C NMR of 46 (126 MHz, DMSO)

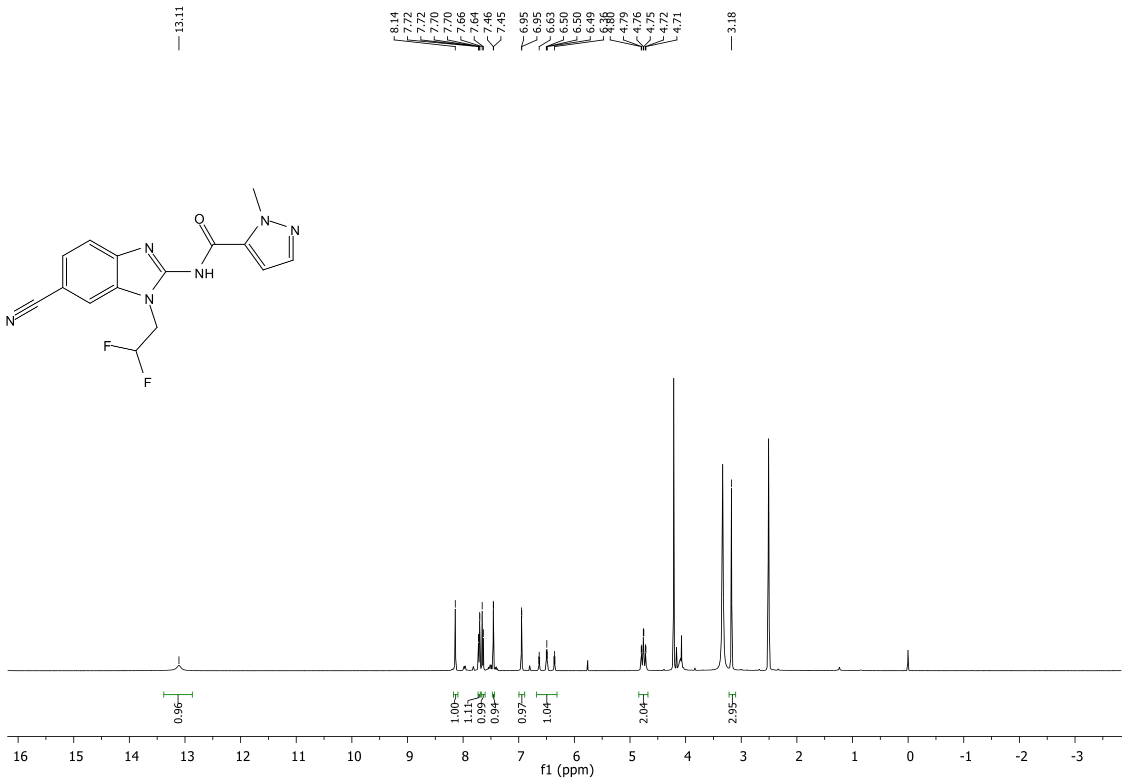

^{1}H NMR of 47 (400 MHz, DMSO)

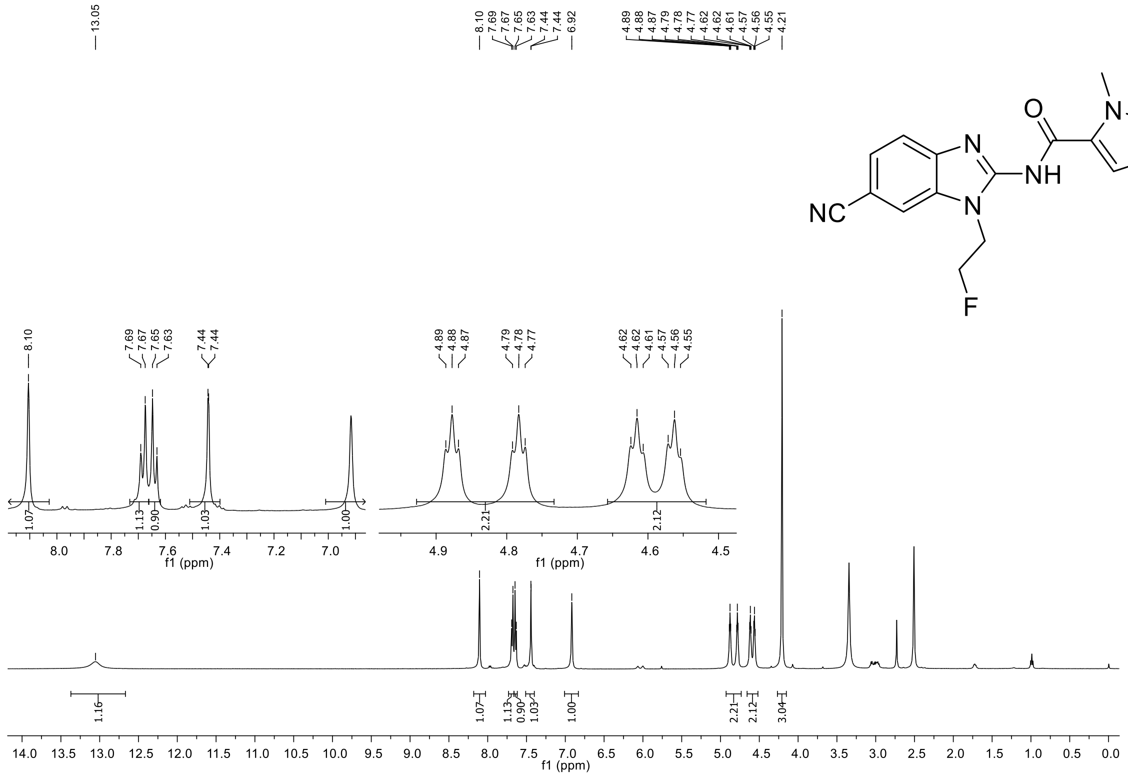

^{1}H NMR of 48 (500 MHz, DMSO)

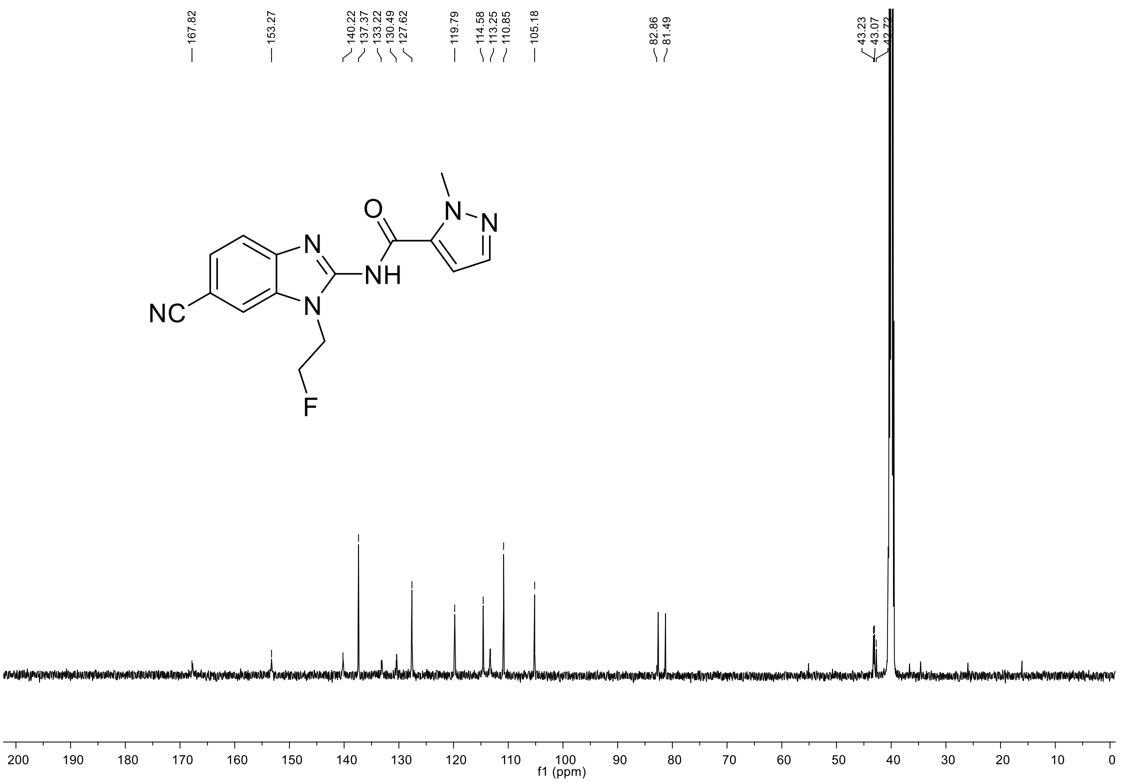

^{13}C NMR of 48 (126 MHz, DMSO)

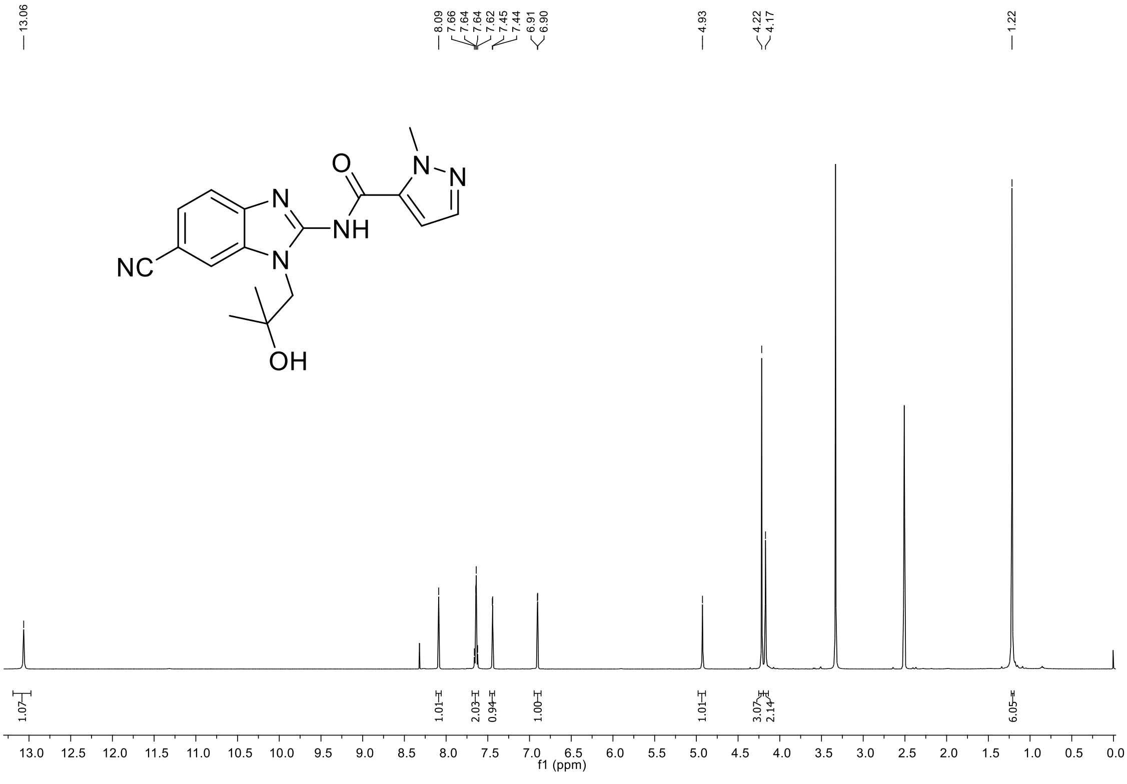

^{1}H NMR of 49 (500 MHz, DMSO)

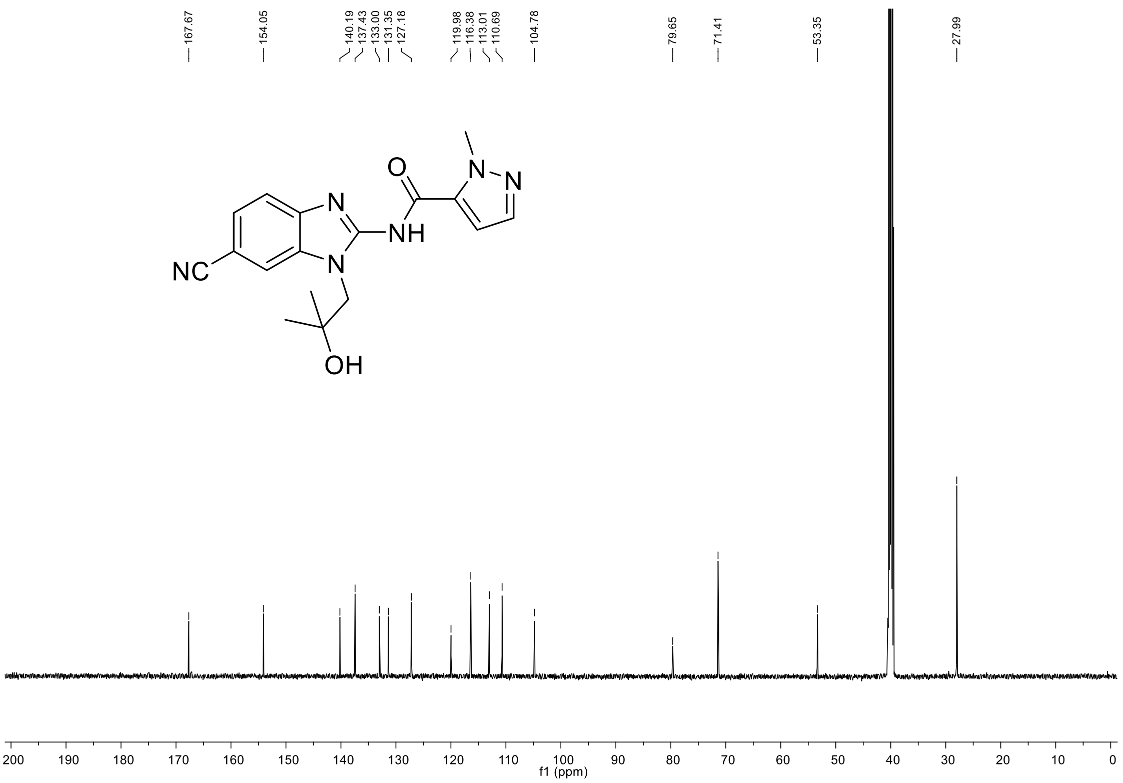

^{13}C NMR of 49 (126 MHz, DMSO)

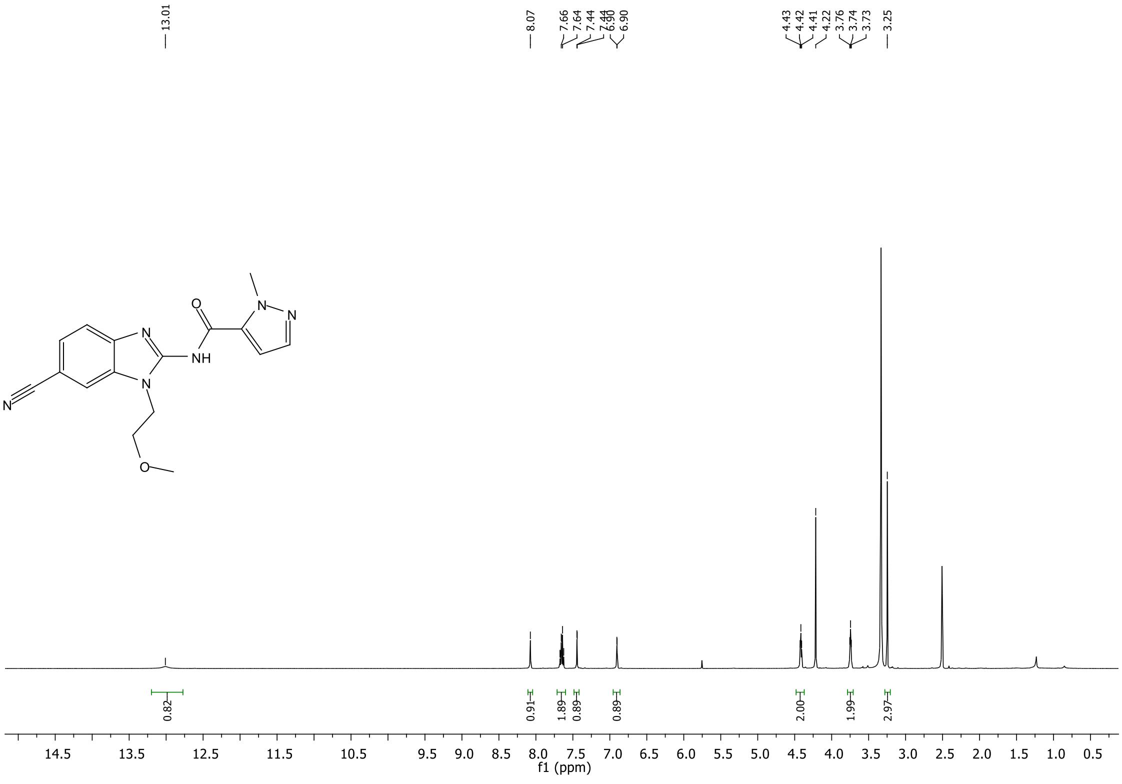

^{1}H NMR of 50 (500 MHz, DMSO)

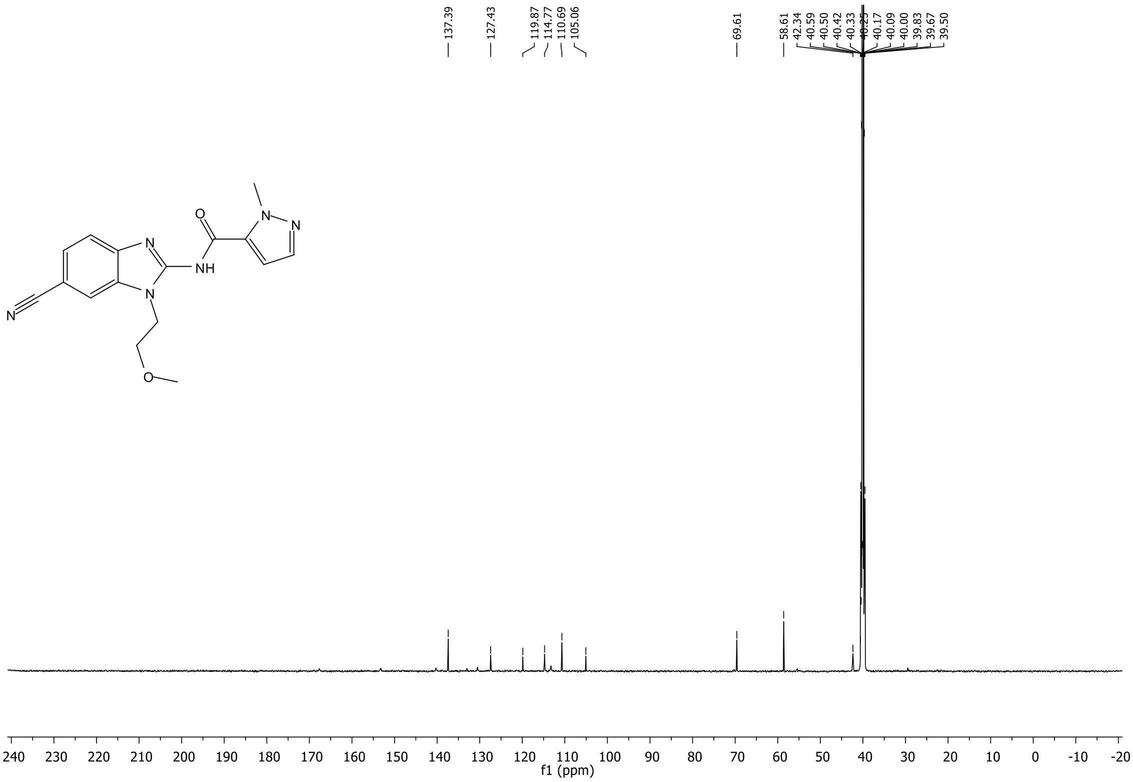

^{13}C NMR of 50 (126 MHz, DMSO)

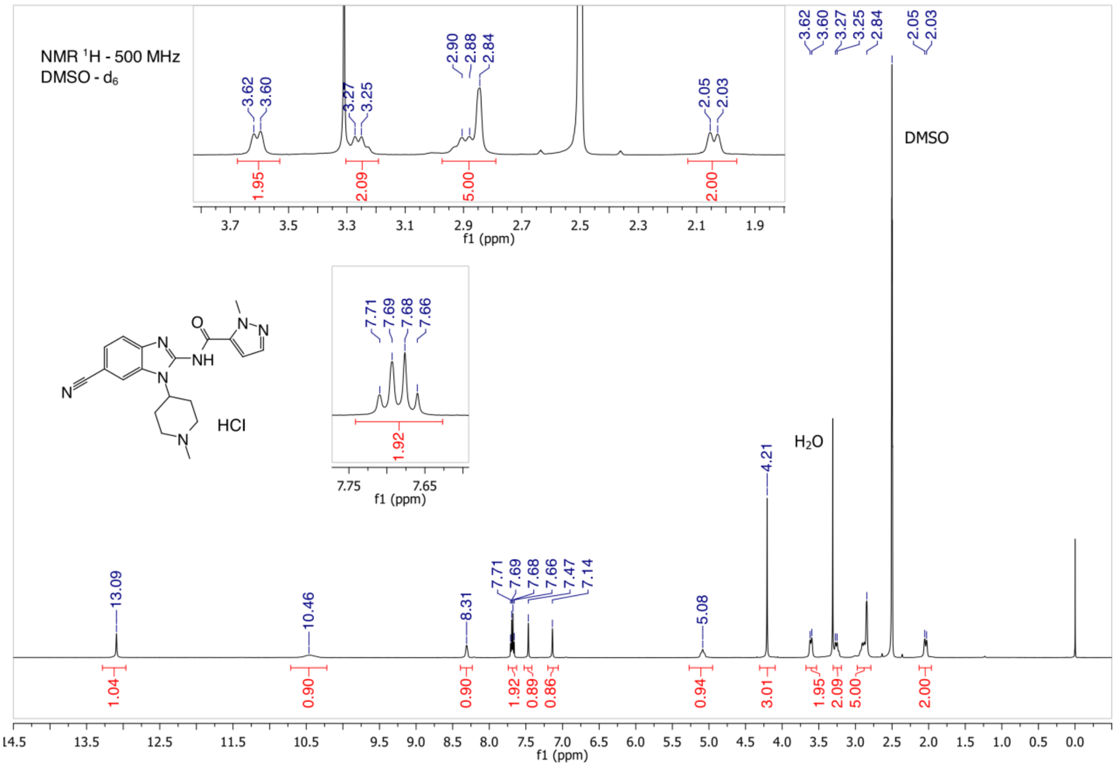

^{1}H NMR of 51 (500 MHz, DMSO)

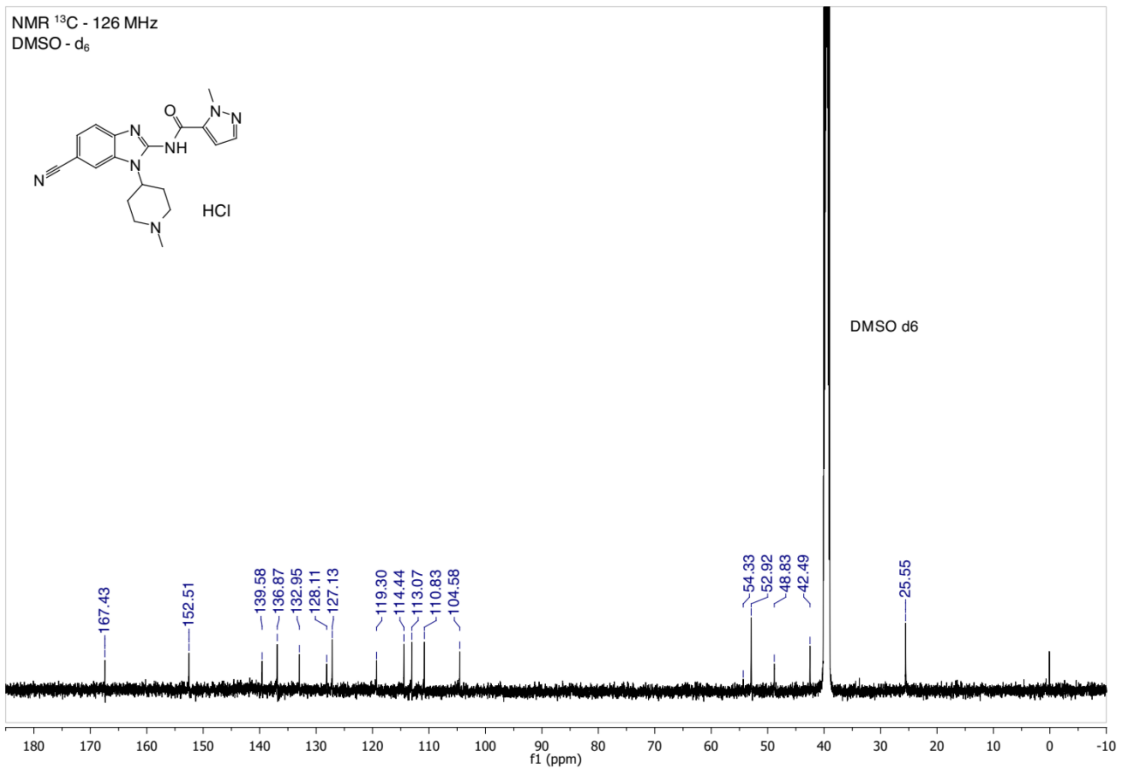

^{13}C NMR of 51 (126 MHz, DMSO)

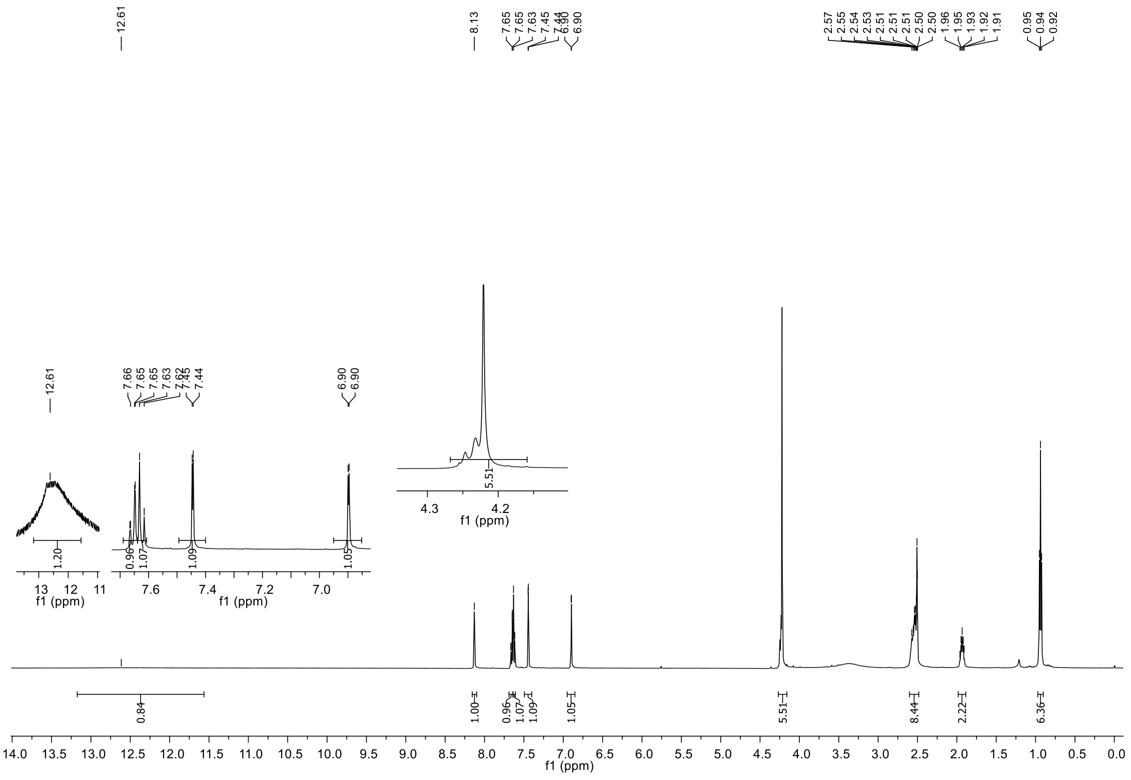

^{1}H NMR of 52 (500 MHz, DMSO)

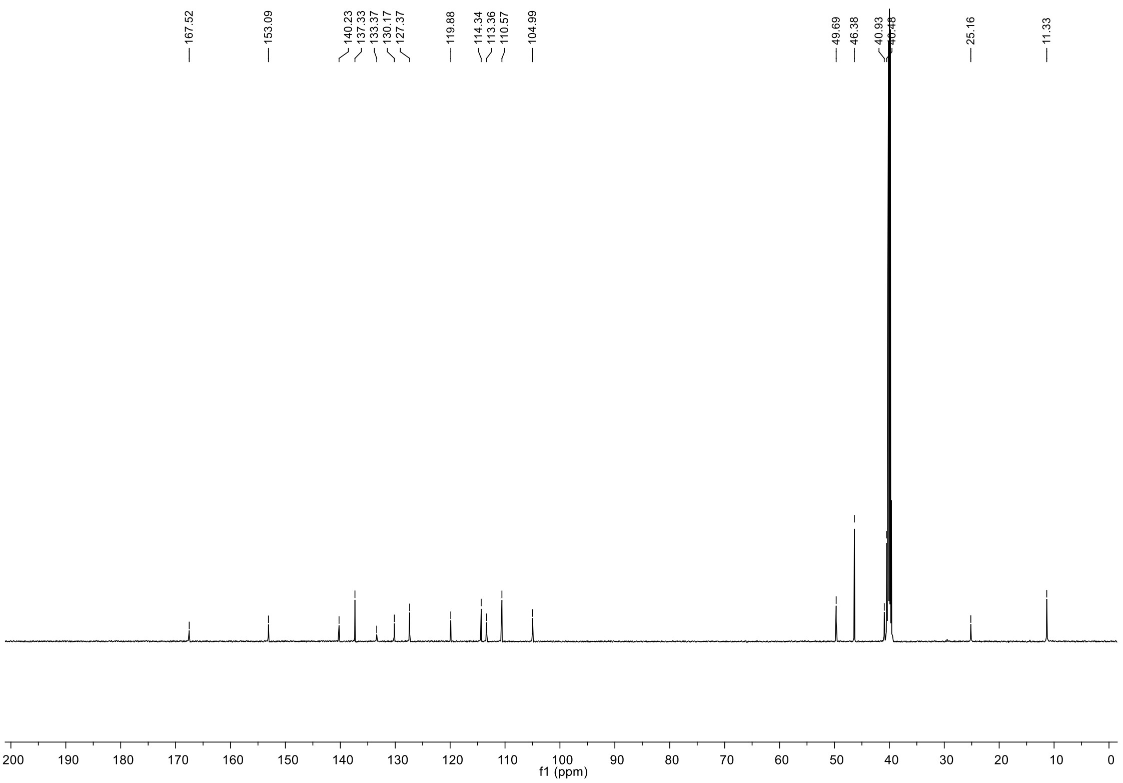

^{13}C NMR 0f 52 (126 MHz, DMSO)

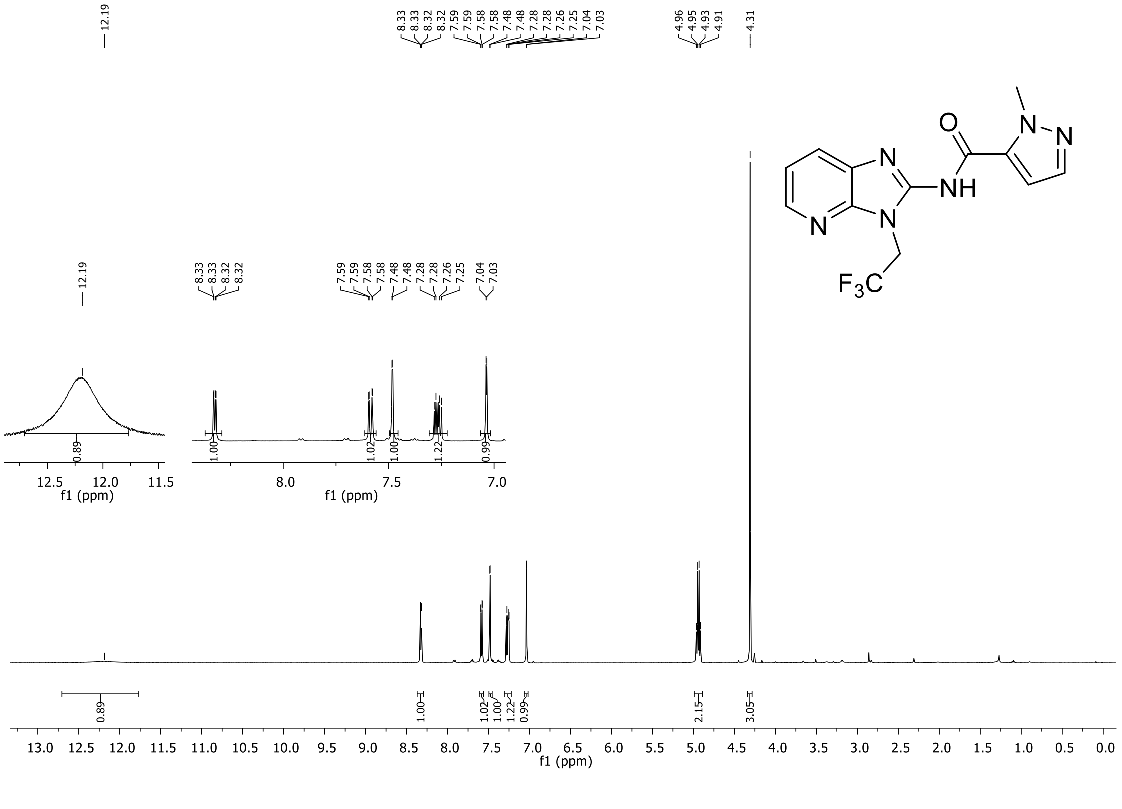

^{1}H NMR of 53 (500 MHz, $CDCl_3$)

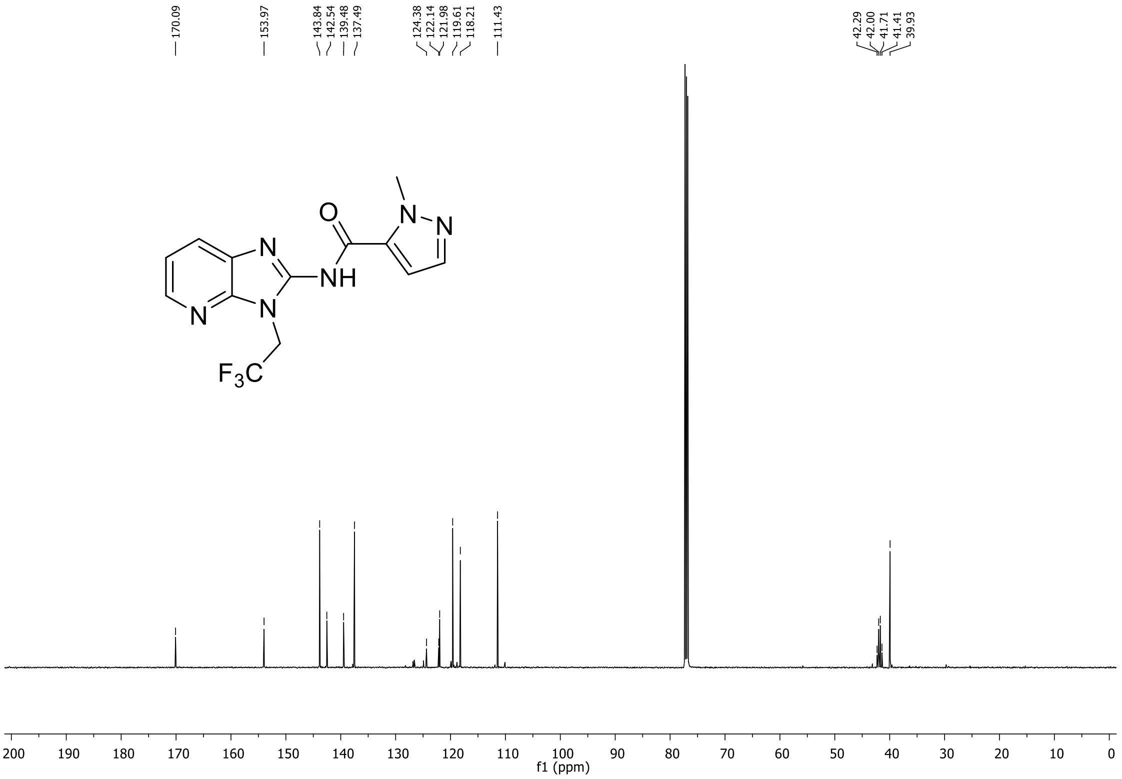

^{13}C NMR of 53 (126 MHz, $CDCl_3$)

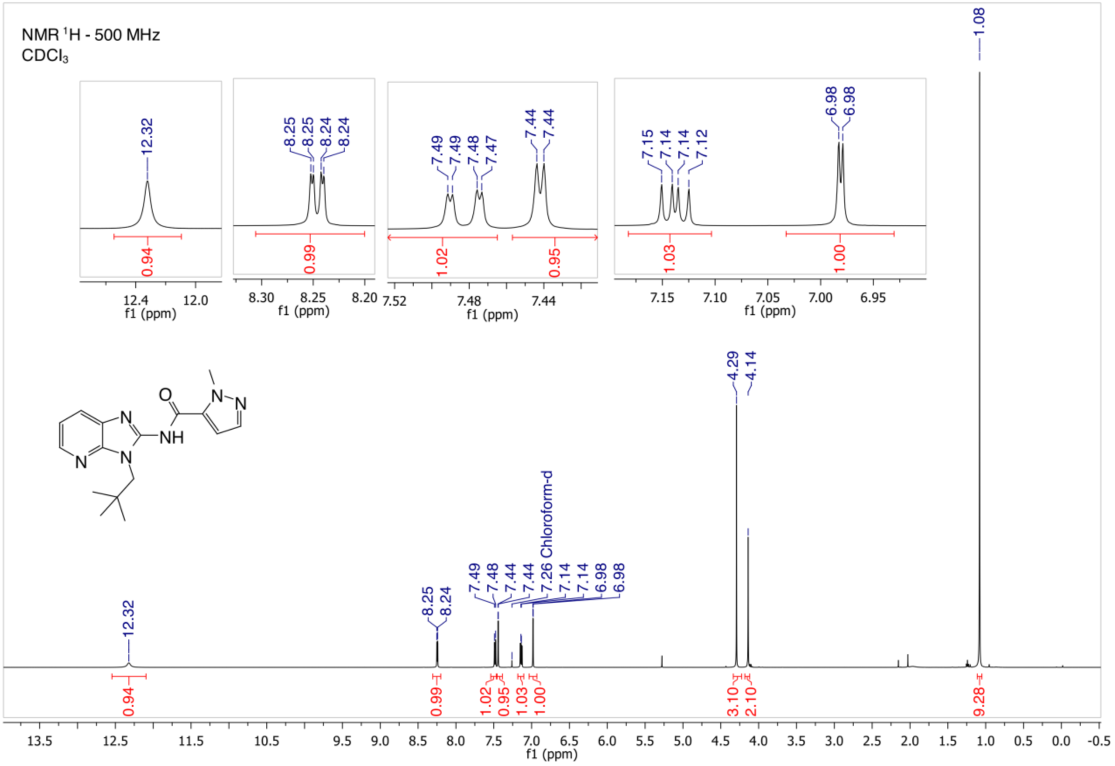

^{1}H NMR of 54 (500 MHz, $CDCl_3$)

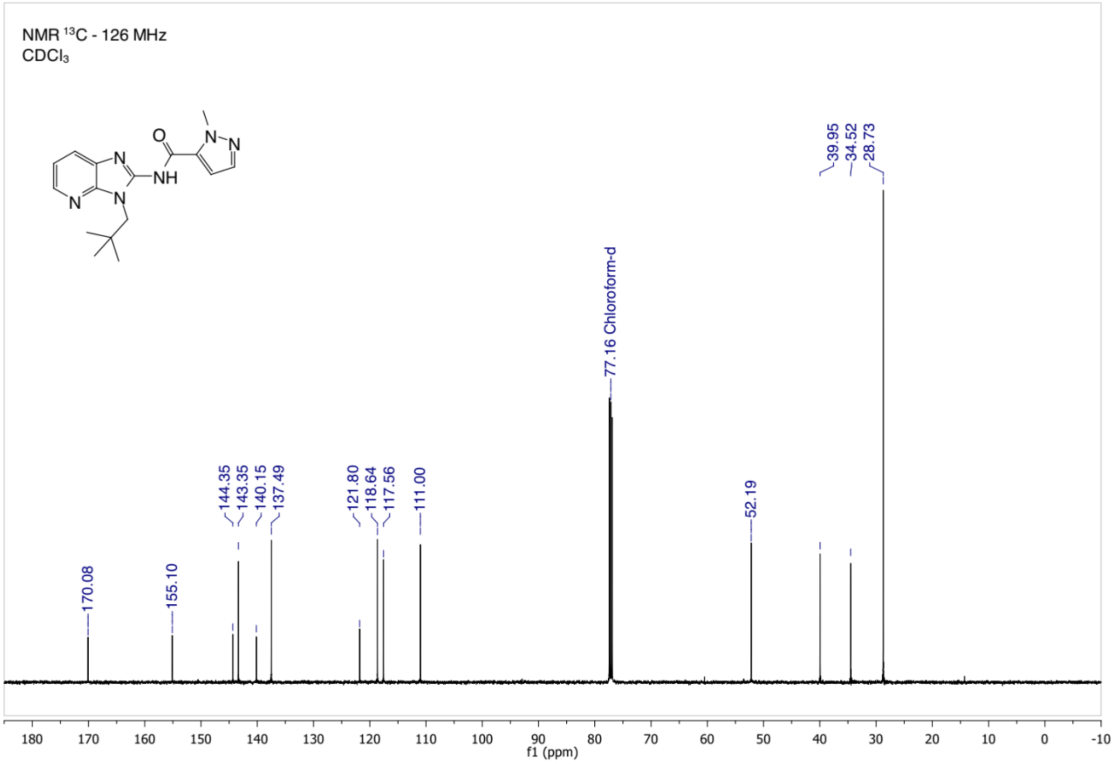

^{13}C NMR of 54 (126 MHz, CDCl$_3$)

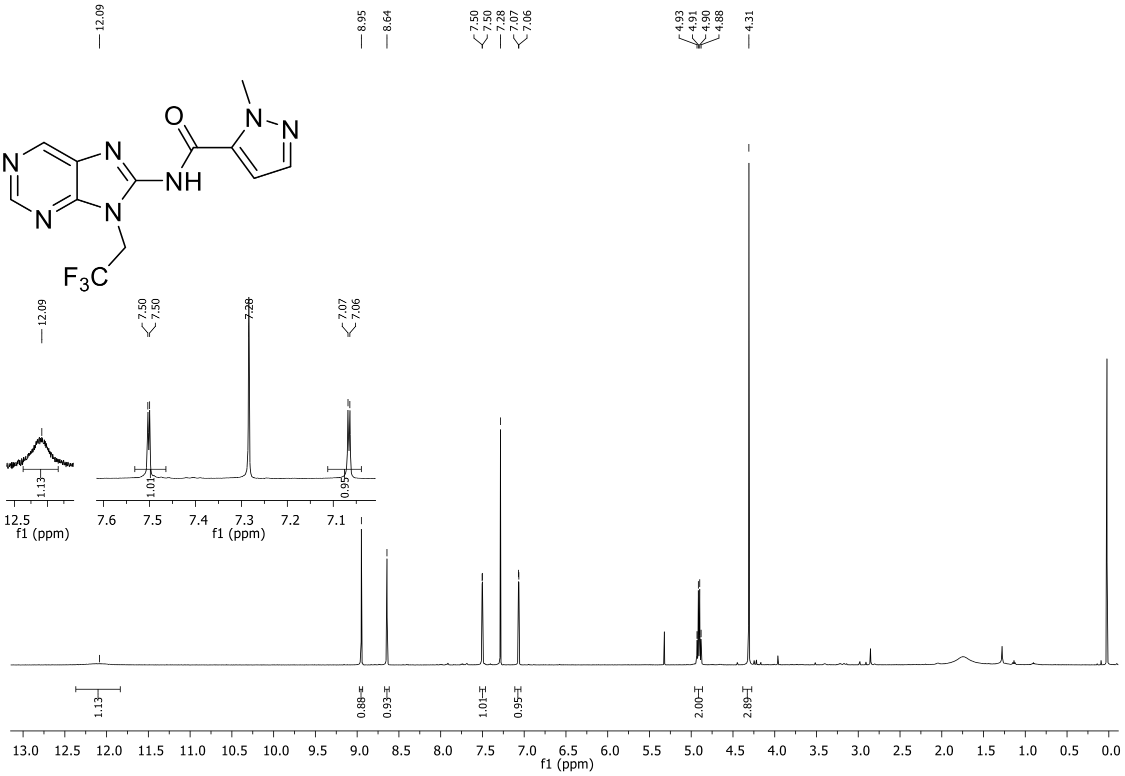

^{1}H NMR of 55 (500 MHz, CDCl$_3$)
